# Body Biofluids for Minimally‐Invasive Diagnostics: Insights, Challenges, Emerging Technologies, and Clinical Potential

**DOI:** 10.1002/adhm.202503096

**Published:** 2025-10-10

**Authors:** Lanka Tata Rao, Chandan Kumar Mandal, Fernando Patolsky

**Affiliations:** ^1^ School of Chemistry Faculty of Exact Sciences Tel Aviv University Tel Aviv‐Yafo 6997801 Israel; ^2^ Center for Nanoscience and Nanotechnology Tel Aviv University Tel Aviv‐Yafo 6997801 Israel; ^3^ Department of Materials Science and Engineering Faculty of Engineering Tel Aviv University Tel Aviv 6997801 Israel

**Keywords:** body biofluids, biomarkers, clinical healthcare, medical diagnosis, minimal‐invasive, non‐invasive, wearable sensing platforms

## Abstract

Recent advances in diagnostics have accelerated the development of miniaturized wearable technologies for the continuous monitoring of diseases. This paradigm is shifting healthcare away from invasive, centralized blood tests toward decentralized monitoring, using alternative body biofluids. Biofluids such as sweat, saliva, urine, and interstitial fluid (ISF) emerged as promising candidates in this context, due to their accessibility and potential ability to reflect physiological states. This review examines recent progress in non‐ and minimally‐invasive diagnostics, with focus on sweat and ISF as potentially suitable biofluids. For biofluids to achieve clinical utility, they must contain quantifiable and disease‐specific biomarker levels, supported by standardized collection and analysis protocols. However, sweat presents inherent limitations for diagnostics, such as intra‐ and interpersonal variability, biomarker concentration fluctuations, etc., and thus only suitable for non‐clinical applications. In contrast, ISF, a robust plasma‐like biofluid potentially fulfills all requirements posed by clinical applications, being readily accessed by minimally‐invasive microneedle‐based platforms. These developing platforms may advance this field by eliminating the need for biofluid extraction, enabling continuous sensing in clinical diagnostics. Future integration of these platforms with AI/ML‐driven algorithms may lead to powerful technologies for real‐time, predictive personalized diagnostics. These technological innovations establish a strong foundation for next‐generation diagnostics and transformative healthcare solutions.

## Graphical abstract

Recent advances in diagnostics have accelerated the development of miniaturized wearable technologies for the continuous monitoring of diseases. This paradigm is shifting healthcare away from invasive, centralized blood tests toward decentralized monitoring, using alternative body biofluids. This review examines recent progress in non‐ and minimally‐invasive diagnostics, with focus on sweat and ISF as potentially suitable biofluids for these applications.

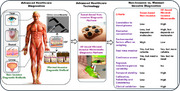

## 1 Introduction

Advanced diagnostic technologies have ushered in a new era of personalized and preventive healthcare and serve to improve treatment efficacy, while marking a shift from invasive blood tests in hospitals to point‐of‐care testing (POCT) involving the noninvasive monitoring of biofluids using portable and wearable devices.^[^ ^1^, ^2^, ^3^ ^]^ Regular and real‐time monitoring is essential for reliable management of chronic illnesses such as cardiovascular disease, diabetes, and neurological disorders. The World Health Organization (WHO) has noted that these diseases account for 75% of global deaths and pose significant economic challenges. The field of POCT has greatly evolved since its inception in 1962, with the development of a method for the rapid analysis (blood sugar levels, infectious diseases, etc.), and further expansion in 1977 with the pregnancy test.^[^ ^4^, ^5^ ^]^ The use of portable diagnostic devices for electrolyte analysis gained traction in emergency settings in the early 1990s.^[^ ^6^ ^]^ POCT provides a rapid turnaround of test results at or near the patients’ location, which facilitates decentralized diagnosis and personalized medicine. The global market for POCT diagnostics is rapidly growing; it was estimated at $45.4 billion in 2022 and is poised to reach $75.5 billion by 2027.^[^ ^7^ ^]^ The development of robust, rapid, and cost‐effective POCT devices is essential for supplementing standard laboratory‐based diagnostic tools and reducing the burden on hospitals.^[^ ^8^, ^9^ ^]^ The shift in the focus of healthcare toward disease prevention and monitoring has prompted a growing demand for painless/no‐discomfort, patient‐centered technologies.^[^ ^10^, ^11^ ^]^ Miniaturized diagnostic platforms have proven effective for the diagnosis and management of health conditions (diabetes, hypertension, etc.). At the same time, wearable sensors have advanced from tracking general biomarkers to specialized applications such as diabetes management.^[^ ^12^ ^]^ Noninvasive and minimally invasive methods of diagnosis have transformed clinical practice, enabling the detection and monitoring of physiological and pathological conditions with minimal or no discomfort.^[^ ^13^, ^14^, ^15^, ^16^ ^]^ These technologies provide alternatives to the conventional invasive methods of diagnosis and offer the advantages of accessibility, real‐time monitoring, and patient compliance. Among the most promising approaches are biofluid‐based diagnostics, which involve non‐ or minimally invasive sampling of easily accessible biofluids such as sweat, saliva, tears, urine, and interstitial fluid (ISF). Serum and plasma have long been considered the gold standard for definitive diagnosis; however, sampling blood via invasive finger pricking causes pain, bruising, and possible infection. The application of biofluid‐based diagnostics is expected to advance blood‐based clinical care. Each biofluid offers a unique diagnostic potential; however, significant differences exist in their reliability, biomarker diversity, and systemic correlation. Among these biofluids, ISF is formed by the filtration of blood through the walls of capillaries and has been recognized as the most potent alternative to blood owing to the similarity of its composition with that of plasma and the fact that it represents the most abundant component (75–80%) of extracellular fluid.^[^ ^9^ ^]^ Therefore, ISF is the most suitable for comprehensive real‐time clinical applications.^[^ ^17^, ^18^ ^]^

Furthermore, true non‐invasive methods, such as those based on sweat, saliva, tears, and urine analysis, do not penetrate the skin and offer user‐friendly, painless sampling. However, these methods often face challenges, including low biomarker concentrations, environmental contamination, and intra‐ and inter‐personal variability in secretion rates, etc., which detrimentally affect diagnostic accuracy. In contrast, microneedle‐based platforms are considered minimally‐invasive because they only penetrate the stratum corneum (dermis layer of the skin), without reaching blood vessels or causing significant pain, and offer ease of use. This approach allows for direct access to ISF through the tiny microneedle elements. It can integrate indwelling sensors to provide quick and continuous biomarker analysis from ISF, which closely resembles blood plasma in terms of biomarker composition and concentration, thus making it more suitable for continuous clinical diagnostics.^[^ ^19^ ^]^

The convenience and integration of noninvasive diagnostic methods based on biofluids such as sweat, saliva, tears, and urine into wearable technologies have been extensively investigated.^[^ ^20^, ^21^ ^]^ Sweat, produced by eccrine and apocrine glands, is a versatile biofluid employed for monitoring hydration status and the levels of electrolytes and stress. Its easy collection and compatibility with wearable sensors make it ideal for monitoring fitness and stress.^[^ ^22^ ^]^ However, sweat faces several limitations, including the instability of biomarker levels, variability in analyte concentrations among individuals and within the same individual, and susceptibility to environmental or physiological changes, compromising the reliability of analytical measurements.^[^ ^23^, ^24^ ^]^ Numerous reports have been published on biofluid analysis using various noninvasive sensing technologies. While numerous biomarkers exist for virtually every disease, not all biomarkers are detectable by wearable devices. The biomarkers can be detected either from serum/plasma or other biofluids such as ISF.^[^ ^25^, ^26^ ^]^ However, the accuracy of diagnostics based on sweat is often hindered by factors such as environmental variability, differences in the activities of region‐specific glands, and intra‐ and interpersonal inconsistencies.^[^ ^27^ ^]^ These factors limit the application of sweat in systemic diagnostics, particularly for conditions requiring high biomarker precision. Saliva is rich in hormones, enzymes, and antibodies, providing a convenient and noninvasive medium for diagnosing oral and systemic health conditions.,^[^ ^28^, ^29^ ^]^ It is frequently used for monitoring the levels of stress hormones such as cortisol, detecting infections, and assessing salivary gland disorders.^[^ ^30^, ^31^ ^]^ Despite its diagnostic potential, saliva is prone to rapid degradation and contamination, reducing its reliability for systemic health assessments.^[^ ^32^, ^33^ ^]^ Tears have been investigated mostly in the context of eye health and to help identify conditions such as dry eye syndrome and conjunctivitis;^[^ ^34^ ^]^ however, their small volume and susceptibility to evaporation limit their wider diagnostic applications.^[^ ^35^ ^]^ Urine plays a key role in metabolic and renal diagnostics, allowing the effective detection of substances such as creatinine and electrolytes.^[^ ^36^ ^]^ The high biomarker concentration in urine makes the biofluid valuable for routine clinical use, but it reflects overall health rather than real‐time conditions.^[^ ^37^, ^38^, ^39^ ^]^ These significant limitations of the noninvasive biofluids render them less attractive for use in modern clinical diagnostics.

In contrast to the abovementioned noninvasive biofluids, ISF emerges as a superior option owing to its plasma‐like composition and systemic representation of biomarkers (see Table 3); it contains a rich array of systemic biomarkers, including glucose, electrolytes, proteins, cytokines, and hormones, providing direct insight into the physiological condition of the individual.^[^ ^40^, ^41^, ^42^ ^]^ Unlike sweat or saliva, ISF offers a stronger and more consistent correlation with blood plasma, enabling the accurate monitoring of systemic conditions. For instance, ISF is highly effective for monitoring glucose for diabetes management, as the glucose levels in ISF closely mirror those in the blood, eliminating the inconsistencies often seen with sweat‐based glucose measurements.^[^ ^18^, ^43^, ^44^ ^]^ Furthermore, ISF enables continuous and real‐time monitoring of inflammatory markers, electrolyte imbalances, and levels of therapeutic drugs, making it a versatile tool for managing chronic diseases.

The development of minimally invasive technologies, particularly microneedle array platforms, has propelled ISF‐based diagnostics into mainstream clinical applications without the need for laboratory resources.^[^ ^45^, ^46^, ^47^ ^]^ Microneedles penetrate the skin's stratum corneum without causing considerable discomfort, allowing the localized, painless, and controlled sampling of ISF.^[^ ^48^, ^49^ ^]^ This technique employs arrays of micron‐sized needles that effectively penetrate the stratum corneum and facilitate the direct delivery of therapeutic agents into the ISF, enhancing absorption and bioavailability.^[^ ^48^, ^50^ ^]^ Recent studies emphasize that microneedles can considerably reduce pain and discomfort compared to conventional hypodermic injections, leading to improved patient compliance,^[^ ^51^, ^52^ ^]^ particularly among populations such as children and those with needle phobia. Advances in microneedle fabrication methods, including the development of dissolving microneedles and the use of biocompatible materials, have paved the way for self‐administered delivery systems for vaccines and treatments for chronic diseases, such as insulin for the treatment of Type I diabetes. Additionally, microneedle arrays have shown promise in new applications, including gene therapy, biosensors for disease diagnostics, and even rapid pain relief.^[^ ^53^, ^54^ ^]^ The versatility and efficacy of the microneedle technology position it as a transformative solution in drug delivery systems, enhancing therapeutic outcomes while minimizing the discomfort typically associated with injections. Integrated with wearable sensors, these technologies enable continuous monitoring of biomarkers and effectively address the limitations of conventional invasive techniques such as blood draws.^[^ ^55^ ^]^ Unlike sweat, saliva, or tears, the samples of ISF obtained using minimally invasive microneedle arrays offer consistency, higher concentrations of biomarkers, stronger systemic relevance, and greater reliability, making them highly suitable for clinical diagnostics and monitoring of therapeutic drugs. The clinical applications of ISF‐based diagnostics span a wide range of conditions.^[^ ^56^ ^]^ The ISF sensors enable continuous monitoring of glucose levels with unparalleled accuracy, reducing the burden of frequent blood sampling associated with diabetes management. For monitoring electrolyte balance and hydration, ISF‐based diagnostics provide real‐time insights critical for managing conditions such as hyponatremia or dehydration.^[^ ^57^ ^]^ ISF‐based diagnostic tools are also involved in therapeutic drug monitoring, ensuring precise medication dosages for patients undergoing chemotherapy or treatments for managing chronic diseases. Additionally, the application of ISF‐based diagnostics is being explored to monitor the levels of inflammatory cytokines and neuropeptides in autoimmune disorders and neurodegenerative conditions.

Our group has recently reported groundbreaking advancements in the development of minimally invasive diagnostic and therapeutic technologies utilizing microneedle‐based platforms. The Clinic‐on‐a‐Needle system integrates silicon nanowire field‐effect transistors (SiNWs‐FETs) with microneedle arrays to enable continuous monitoring of biomarkers in the ISF and related drug delivery.^[^ ^56^ ^]^ Designed for applications such as a closed‐loop artificial pancreas, this innovative system provides a transformative solution for the management of diabetes. It ensures painless dermal insertion, rapid recovery of skin integrity, and high target specificity with resistance to chemical interferences, making it suitable for continuously monitoring glucose and lactate levels. Furthermore, the integration of insulin delivery systems within the same platform supports real‐time therapeutic interventions. The system was validated via in vivo experiments and initial human trials. It enabled accurate detection of glucose levels and precise drug administration, offering a patient‐friendly alternative to the conventional methods. In parallel, nanopillar‐embedded microneedle arrays leverage vertically aligned silicon nanopillars (SiNPs) for fluorescence‐based detection of protein biomarkers directly from intradermal samples.^[^ ^58^ ^]^ This technology supports multiplexed detection of biomarkers, allowing the simultaneous analysis of specific targets such as prostate‐specific antigen (PSA) with high sensitivity and specificity. The nanopillars' architecture significantly enhances the sensing surface area, facilitating rapid and accurate detection while requiring minimal sample volumes. These microneedle platforms eliminate the need for invasive blood draws, making them ideal for POCT, particularly under resource‐limited settings. The scalable fabrication process ensures cost‐effectiveness and robustness, expanding the potential of these systems for widespread clinical adoption.

The development of both these approaches represents milestones in minimally invasive diagnostics and addresses key challenges in healthcare, such as patient discomfort, the need for real‐time monitoring, and the limitations of centralized laboratory testing. The Clinic‐on‐a‐Needle system focuses on continuous monitoring and therapeutic applications, particularly for chronic diseases such as diabetes, and the nanopillar‐embedded microneedles, in multiplexed detection of biomarkers for early diagnosis and precision medicine. Together, these technologies promise to transform clinical practices by improving patient compliance, enhancing the accuracy of diagnosis, and enabling personalized healthcare solutions. Their integration into wearable devices further emphasizes their potential to revolutionize healthcare delivery, particularly by increasing the accessibility, scalability, and efficiency of diagnostics and therapy for chronic and acute‐care management practices. From a scalability perspective, the mass production of advanced microneedle‐based biosensing platforms requires cost‐effective, reproducible, and high‐quality manufacturing processes to meet clinical demands. Additionally, obtaining regulatory approval necessitates rigorous validation of analytical performance, clinical accuracy, and evidence of patient benefits. This highlights the importance of standardized sample collection protocols and comprehensive clinical studies. Furthermore, feasibility challenges arise concerning long‐term biocompatibility, user safety, and the consistent performance of integrated devices in real‐world conditions.

The suitability of biofluids for diagnostic applications is highly specific to their intended use. It depends on factors such as biochemical profiles, biomarker concentrations, physiological origins, and compliance with regulatory standards. Non‐invasive fluids, like sweat and saliva, are ideal for low‐risk, continuous monitoring of glucose levels and hydration, stress, fitness, and metabolic trends because they are easy to collect and encourage user compliance. However, their diagnostic value is often limited by factors such as low biomarker concentrations, potential external contamination, physiological variability, environmental variability, etc. On the other hand, ISF, which can be obtained through minimally invasive microneedle‐based devices, provides a rich source of clinically relevant biomarkers with a strong correlation to blood. This makes ISF a promising biofluid candidate for precision diagnostics.^[^ ^60^, ^61^ ^]^

As diagnostics progress toward clinical integration, regulatory approval strategies are crucial in determining which biofluids can be widely used. Biofluids intended for clinical‐grade diagnostics must meet stringent standards for analytical validity, clinical accuracy, and reproducibility, as specified by agencies like the FDA and EMA. Platforms based on ISF, especially those that utilize minimally invasive microneedle‐based devices, with integrated indwell biosensors, are gaining more attention because they can fulfill all these requirements. To facilitate clinical translation, it is essential to establish standardized protocols for low‐volume biofluid collection, easy processing, and biomarker quantification, along with thorough clinical validation studies. These steps are vital for obtaining regulatory approval and ensuring safe and scalable implementation in real‐world healthcare environments.^[^ ^43^, ^61^, ^62^ ^]^

This comprehensive review compares various diagnostic biofluids to demonstrate the clear superiority of ISF over the others owing to its stable composition, systemic correlation, and ability to provide real‐time insights. The noninvasive technologies utilizing biofluids such as sweat and saliva remain invaluable for specific applications such as the monitoring of hydration, fitness, stress, or oral health; however, the minimally invasive microneedle technologies that leverage ISF are uniquely positioned for addressing the demands of systemic diagnostics and offer unmatched accuracy for the management of chronic diseases and therapeutic drug monitoring. With ongoing research and technological advancements, minimally invasive ISF‐based diagnostics are poised to redefine the future of personalized and preventive medicine. The integration of noninvasive and minimally invasive diagnostic platforms, notably those based on ISF, with wearable technologies marks a significant step forward in personalized and preventive medicine; in this context, ISF further enhances its clinical utility and paves the way for more accurate, accessible, and patient‐friendly diagnostic solutions. With the ongoing evolution of these technologies, addressing challenges with respect to their scalability, reliability, and affordability will be critical for broadening their application in global healthcare systems and advancing their clinical utility.

## 2 Sweat as a Biofluid for Diagnostic Applications

### 2.1 Physiology of Sweat

**Figure 1:**
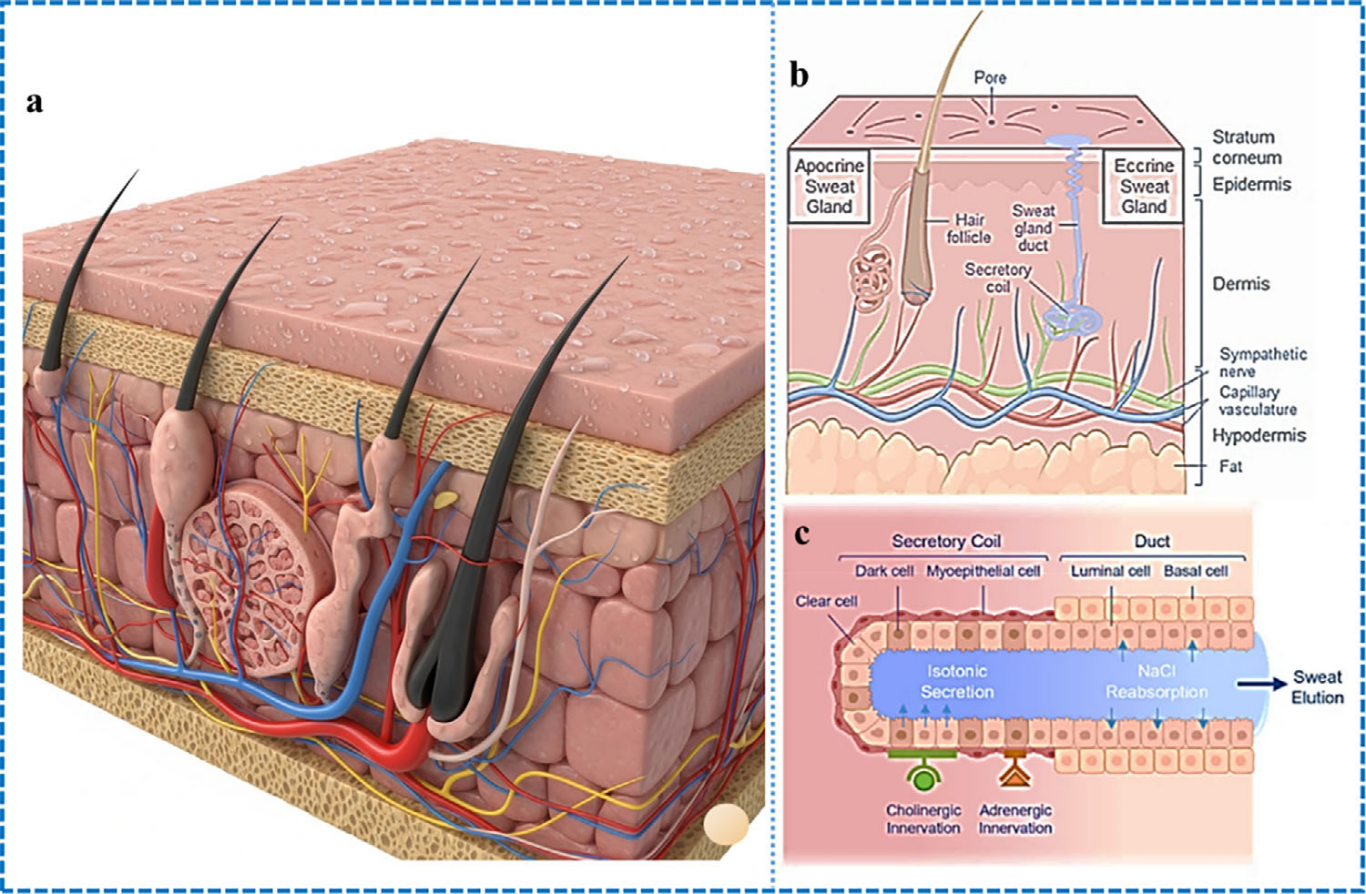
a) Illustration of the structure and function of sweat glands, with a focus on eccrine sweat secretion. b) Overview of skin anatomy, highlighting glands, including eccrine and apocrine glands. c) The eccrine gland consists of two key segments: a deep secretory coil responsible for generating an isotonic fluid, and a connecting duct where ion reabsorption occurs, resulting in the release of hypotonic sweat. Eccrine sweat production is regulated through β‐adrenergic and muscarinic receptors, which activate chloride channels via cAMP and calcium signaling pathways, respectively. Nicotinic receptors may further enhance sweat output through a sudomotor axon reflex, expanding the response beyond the initial stimulus zone. Reproduced with permission.^[^ ^74^ ^]^ Copyright 2023 ACS.

Sweat glands are found deep within the skin, which is the largest organ in the body in terms of surface area. The skin is composed of different layers, including the dermis, epidermis, stratum corneum, and hypodermis. The dermis is an important layer that contains nerve endings, blood vessels, hair follicles, sebaceous glands, and sweat glands (**Figure** **1**). The average density of eccrine sweat glands is ≈200 cm^−2^; however, the density can vary between individuals as well as different parts of the body, with the maximum density found on the palms and soles (≈ 400 cm^−2^).^[^ ^63^ ^]^ The total number of eccrine sweat glands in an individual ranges from 1.6 to 5 million.^[^ ^63^ ^]^ Sweat plays a key role in thermoregulation, which is essential for optimal health and the maintenance of the internal temperature of the body. Without thermoregulatory mechanisms, the body's internal temperature can rise above 40°C, which increases the risk of protein denaturation, cell death, and eventual organ failure.^[^ ^63^ ^]^ In addition to the regulation of body temperature, sweating also contributes to skin homeostasis. Lactate and urea are two moisturizing substances found in sweat and help maintain the integrity of the barrier as well as the flexibility of the outer layer of the skin (stratum corneum). In addition, antimicrobial agents such as dermicidin, lactoferrin, lysozyme, and IgE antibodies are secreted in sweat and help protect the skin from infection.^[^ ^64^ ^]^ Several injuries to the human body can cause the destruction of sweat glands, as is the case with burn injuries. Healing the wounds and damage caused by burn injuries presents several challenges, and further research on wound healing, as well as sweat gland physiology, is required for addressing these.

Electrolytes, metabolites, and molecules (carbohydrates, lipids, nucleic acids, and proteins) are present in the highly filtered aqueous fluid secreted by the eccrine sweat glands. By contrast, the apocrine sweat glands secrete a viscous fluid that includes lipids, proteins, steroids, and ions into the gland's coil via exocytosis.^[^ ^65^ ^]^ The apocrine secretions contain volatile chemical molecules that function as pheromones.^[^ ^63^ ^]^ Different stimuli trigger the apocrine and eccrine sweat glands. The apocrine sweat glands respond intensely to emotions and sympathomimetic medications with adrenergic innervation. However, unlike the eccrine sweat glands, they are not responsive to cholinergic or heat stimulation.^[^ ^64^, ^66^ ^]^ Apoeccrine sweat glands share properties of both eccrine and apocrine glands. They may arise from existing eccrine glands in the underarm area during puberty. This gland has an eccrine‐like sweat duct but a secretory duct similar to that of apocrine glands. The sweat produced by apoeccrine glands is similar to the watery eccrine sweat and is produced upon moderate stimulation.^[^ ^67^ ^]^

Particularly, eccrine sweat glands, which are widely distributed across human skin, play a crucial role in sweat production and serve as an important interface for sweat‐based diagnostics. These glands consist of a secretory coil and a ductal system, with distinct cellular subtypes responsible for secretion and reabsorption. Clear cells secrete water and electrolytes, while dark cells contribute proteins, glycoproteins, and other biomolecules to the sweat fluid. As the primary secretion travels through the duct, selective reabsorption of sodium and chloride ions alters the final composition of sweat before it is released onto the skin surface.^[^ ^68^ ^]^ This physiological process directly influences the diagnostic potential of sweat.^[^ ^69^ ^]^ Sweat contains a wide range of clinically relevant constituents, including electrolytes, metabolites, proteins, hormones, and external compounds such as drugs and alcohol. Factors such as secretion rate, gland density, body location, and the mechanism of stimulation (exercise, thermal, or pharmacological) significantly impact the concentration and stability of these analytes. High secretion rates may dilute biomarker levels, whereas slow or localized secretion can increase the risk of contamination and surface degradation. Moreover, variability in sweat gland activity among individuals adds complexity to establishing standardized reference ranges, presenting a challenge for reproducibility and clinical application. Clinical practice highlights the diagnostic significance of sweat physiology. For example, sweat chloride testing is the gold standard for diagnosing cystic fibrosis, as impaired ductal ion reabsorption leads to elevated chloride levels.^[^ ^70^ ^]^ Similarly, sweat glucose monitoring has been explored as a non‐invasive method for managing diabetes; however, its low concentrations and physiological variability limit its ability to correlate quantitatively with blood glucose levels. Sweat lactate can indicate exercise intensity and muscle metabolism, while ethanol in sweat has been effectively used for continuous alcohol monitoring. These indicate that a comprehensive understanding of glandular physiology is essential not only for identifying relevant biomarkers but also for interpreting them, considering the dynamics of secretion. Recent advancements in wearable microfluidic,^[^ ^71^ ^]^ electrochemical sensing technologies, standardized collection strategies, and calibration algorithms aim to address these physiological challenges.^[^ ^72^, ^73^ ^]^

### 2.2 Sweat Glands and Their Diagnostic Relevance

The physiological diversity of sweat gland types and their secretion mechanisms plays a significant role in understanding the variability in sweat and the diagnostic reliability of sweat‐based platforms. Eccrine, apocrine, and apoeccrine glands contribute differently to sweat volume and biochemical composition, thereby influencing the quality and interpretability of analytes. Notably, their diagnostic outputs are strongly affected by intra‐ and inter‐personal variability, environmental conditions (temperature, humidity, physical activity), and biological factors such as age, gender, and hormonal status. These variables collectively impact gland activity, sweat rate, and biomarker concentration, leading to fluctuations in diagnostic accuracy. Hence, a comprehensive understanding of gland types, secretion mechanisms, and variability factors is crucial for developing standardized calibration strategies that ensure consistent, reproducible, and clinically significant diagnostics.

**Figure 2:**
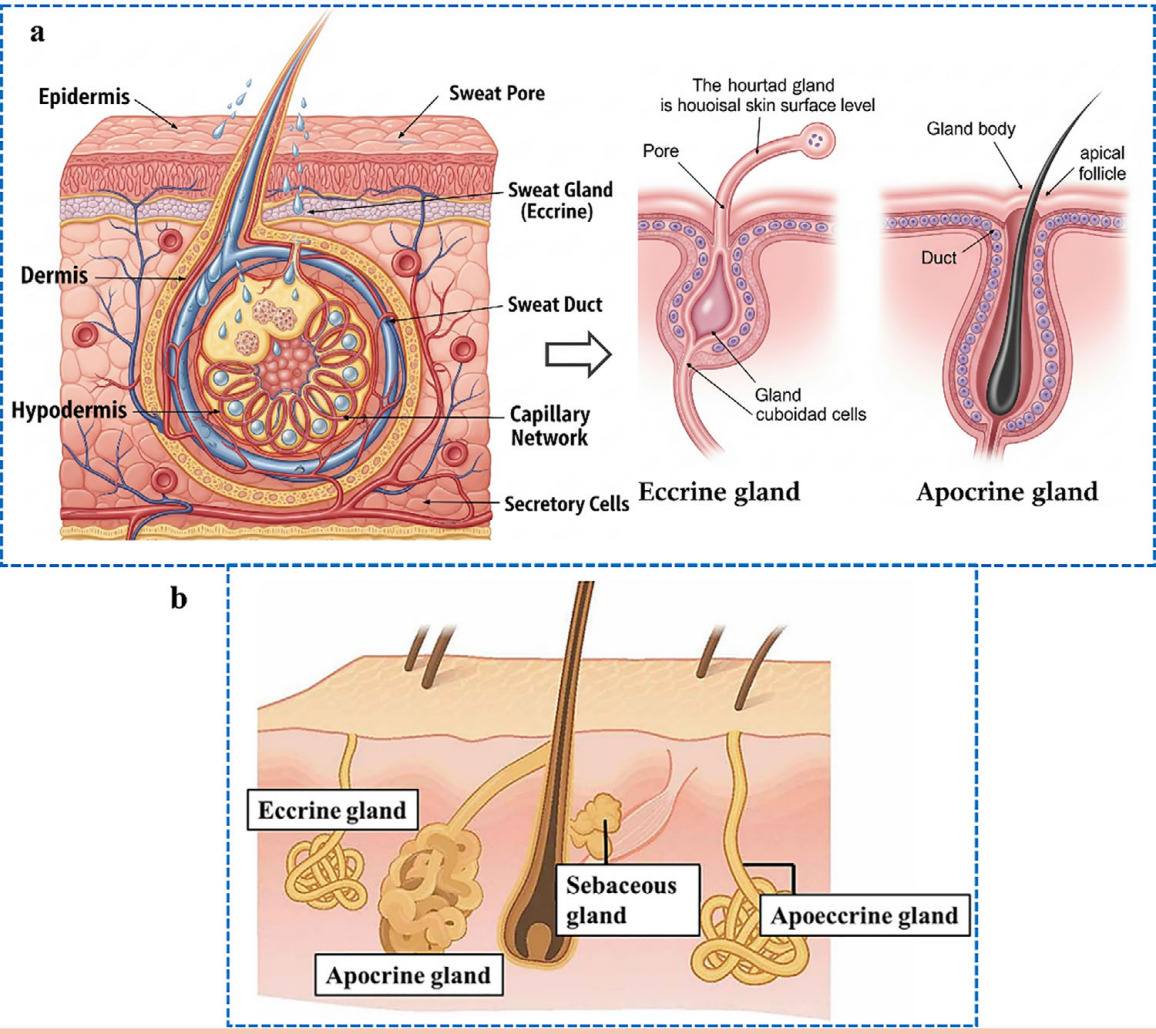
a). The detailed description of skin layers, sweat mechanism, and their gland types. b). A comparison of the apocrine, eccrine, and apoeccrine glands in the axilla, highlighting their distinct characteristics, locations, secretions, and functions within the skin of the underarm region. Reproduced with permission.^[^ ^81^ ^]^ Copyright 2019 Temperature.

Human skin contains three primary types of sweat glands: eccrine, apocrine, and apoeccrine, as shown in **Figure** **2**. Eccrine sweat glands are the most abundant and widely distributed, producing most of the sweat across the body. In contrast, apocrine and apoeccrine glands are localized to specific regions and contribute less to the overall sweat volume. However, they significantly influence the biochemical profile of sweat on the skin surface, which is highly relevant for diagnostic applications.

Eccrine glands are small, numerous, and widespread, with ≈2 to 4 million glands throughout the body. They are found on both glabrous skin (palms and soles, where their density ranges from 250 to 550 glands cm^−^^2^) and nonglabrous skin (face, trunk, and limbs, where their density is lower, but they cover a larger area).^[^ ^72^, ^75^, ^76^ ^]^ Eccrine glands become functional by the age of 2 to 3 years, and their density decreases as body surface area increases. Their primary function is thermoregulation through the secretion of electrolyte‐rich fluid. From a diagnostic perspective, eccrine sweat is highly valuable since it provides a reproducible source of biomarkers such as electrolytes, glucose, lactate, cortisol, and stress‐related metabolites. This makes eccrine glands the primary target in most sweat‐based sensing technologies.^[^ ^77^, ^78^, ^79^ ^]^

Apocrine glands are larger and are localized to areas such as the armpits, scalp, breasts, face, and perineum, as shown in Figure 2a.^[^ ^80^ ^]^ They become active only after puberty. Their secretions, which are delivered into hair follicles, are thick, lipid‐ and protein‐rich, and contain metabolites like ammonia and sugars. Although their role in thermoregulation is minimal, apocrine secretions are diagnostically important due to their biochemical complexity, which reflects metabolic and microbiome‐related processes. These secretions also affect odor generation, presenting new opportunities for volatile biomarker analysis in disease and stress diagnostics.

Apoeccrine glands develop from eccrine precursors between the ages of 8 and 14, and by late adolescence (Figure 2b), they can represent up to 45% of the glands found in the axillary area. These glands share characteristics of both eccrine and apocrine glands: like eccrine glands, they connect directly to the skin surface and produce high‐volume, salt‐rich secretions; like apocrine glands, they contribute proteins and lipids. While they play a minimal role in thermoregulation, apoeccrine sweat is diagnostically relevant because it is dominant in the axilla, where its mixed composition influences local biomarker concentrations. This makes apoeccrine glands significant for studies examining regional variability in sweat analytes, especially in the context of stress, hormonal changes, or metabolic diagnostics.^[^ ^67^ ^]^ The diversity of sweat gland types presents both opportunities and challenges for sweat‐based diagnostics. Eccrine glands serve as the foundation for systemic, wearable biosensing platforms; apocrine glands offer rich molecular content for specialized diagnostic applications; and apoeccrine glands remain an underexplored hybrid source.

### 2.3 Impact of Gland Heterogeneity on Biomarker Reliability and Wearable Sensors

Gland heterogeneity presents a significant challenge to the reliability of sweat‐based biomarker measurements. Variations in gland type, density, and secretory mechanisms directly affect the biochemical composition of sweat. Eccrine, apocrine, and apoeccrine glands differentiate in their cellular structures, innervation patterns, and secretion modes, leading to distinct fluid compositions, ion concentrations, and biomolecule profiles. Even among the same gland type, inter‐individual differences in gland size, metabolic activity, and responsiveness to stimuli (thermal stress, physical exertion, or pharmacological agents like pilocarpine) can result in significant variability in biomarker concentrations. Moreover, this biological variability introduces considerable noise in measurement, making it challenging to differentiate between genuine systemic changes and local gland‐specific fluctuations. The concentration of a specific biomarker may vary significantly between different anatomical sites due to variations in gland density, local metabolic processes, and environmental exposure, potentially leading to inconsistent diagnostic interpretations. Additionally, temporal variability, hydration status, hormonal cycles, circadian rhythms, or prior stimulation can impact intra‐individual reproducibility, further undermining analytical consistency. In clinical diagnostics, heterogeneity can lead to false positives, false negatives, or misleading trends if gland‐specific influences are not carefully controlled. Therefore, standardizing collection sites, implementing calibration strategies against reference fluids, and validating biomarkers in a site‐ and gland‐specific manner are essential steps to enhance the accuracy, reproducibility, and overall diagnostic validity of sweat‐based assays.^[^ ^82^, ^83^ ^]^

Moreover, this gland heterogeneity is a significant source of variability in biomarker analyses for wearable sensors. Even when physiological conditions are the same, differences in gland characteristics can lead to considerable variations in biomarker concentrations between different sensor sites. Additionally, individual factors such as age, gender, ethnicity, and the functional capacity of glands can further increase this variability. In addition, transient physiological states, such as changes in hydration, thermal stress, and physical activity, also affect gland patterns and secretion profiles. All these factors contribute to temporal variability, which can undermine the reproducibility, comparability across individuals, and long‐term reliability of biomarker measurements by wearable sensors. To address these challenges, effective strategies must be implemented, which might include optimizing sensor placement, using calibration algorithms, or employing multi‐site sensing methods. Such approaches are essential to ensure that the output from wearable sensors achieves the accuracy and clinical relevance necessary for diagnostic purposes or continuous monitoring applications.^[^ ^84^ ^]^

### 2.4 Structure of Sweat Glands and Mechanism

The eccrine sweat tubule is 4–8 mm in length and acts as a conduit for the exchange of sweat and electrolytes. The secretory coil at the base is 500–700 µm in size, with inner and outer diameters of 30‐40 µm and 60‐120 µm, respectively.^[^ ^63^ ^]^ The secretory coil is associated with capillaries for exchange with blood and sudomotor nerve fibers for regulation by the autonomic nervous system.^[^ ^85^, ^86^ ^]^ The secretory tube then straightens into the dermal duct, which has inner and outer diameters of 10–20 and 50–80 µm, respectively, and is composed of 2‐3 layers of epithelial cells.^[^ ^63^ ^]^ The sweat duct passes directly from the dermis to the epidermis before changing into an epidermal helical structure that ends at the stratum corneum. The pitch angle remains constant across sweat glands, while the number of turns of the helical duct ranges from four to six and fluctuates according to the thickness of the stratum corneum.^[^ ^87^ ^]^ The helical structure of the sweat duct endows it with resonance properties, allowing it to function as a helical antenna. The resonant frequency and interaction of the skin with terahertz waves are influenced by the dimensions, density, and distribution of sweat ducts, as well as the dielectric properties of the stratum corneum. The length of the duct varies from 150 to 600 µm, and in accordance with the thickness of the stratum corneum.^[^ ^87^ ^]^ The lumen has a diameter ranging from 20‐60 µm and may contain cornified cells.^[^ ^63^ ^]^

Human sweat glands chiefly develop during the first two trimesters of pregnancy, and nearly the entire repertoire is present at birth, which explains the higher duct density in children than in adults.^[^ ^67^ ^]^ The sweat gland originates from a group of multipotent K14^+^ progenitor cells, which are derived from epidermal stem cells. It extends downward as a straight duct, whose lower half contains stratified proliferative K14^low^/K18^+^ suprabasal progenitors. These K14^low^/K18^+^ suprabasal progenitors develop into luminal cells, while the rest of the K14^+^ progenitors differentiate into myoepithelial cells.^[^ ^88^ ^]^ The turnover and proliferation capacities of the sweat glands are limited; however, regeneration potential is exhibited by these glands to a certain extent. The expansion and positioning of stem cells associated with the secretory luminal and myoepithelial cells at the wound site have been found to aid in the regeneration of the epidermis and sweat glands.^[^ ^89^ ^]^ Moreover, earlier studies have reported the utilization of 3D bioprinting matrices for investigating tissue‐level self‐organization based on the formation of sweat glands.^[^ ^90^ ^]^ The secretory coil and duct play key roles in isotonic secretion and salt reabsorption, respectively, with these representing the two main processes in the production of sweat (Figure 1c). The process of transepithelial reabsorption is facilitated by the ductal cells and assisted by the mitochondria‐rich basal cells. The secretory coil consists of the basal myoepithelial, dark, and luminal clear cells, which can be recognized based on their appearance when stained with eosin, toluidine blue, and methylene blue, respectively.^[^ ^63^ ^]^ The myoepithelial cells support the structure of the secretory coil and generate a microenvironment that supports the differentiation of the stem cells in the sweat glands.^[^ ^91^ ^]^ Dark cells are small, compact, filled with large vesicles, and contain particulate substances. They play an important role in the secretion of proteins, including the Periodic Acid Schiff (PAS), positive diastase‐resistant glycoproteins, dermicidin, and sialomucin.^[^ ^91^ ^]^ Further research is needed to elucidate the interdependent association between clear and dark cells. Sweat secretion is triggered by adrenergic and cholinergic innervation, as shown in Figure 1c. Ouabain and metabolic inhibitors reduce the sudomotor response, which involves various adenosine triphosphate (ATP) dependent steps.^[^ ^85^ ^]^ A signaling cascade initiated upon the stimulation of the secretory cells utilizes either Ca^2+^ or cAMP as the second messenger, which, in turn, triggers the efflux of chloride ions (Cl^−^) into the lumen of the secretory coil. Sodium ions (Na^+^) are circulated at the basolateral membrane, which subsequently releases their electrochemical gradient (Na^+^) into the lumen. This release of electrolytes into the lumen makes it hypertonic compared to the cytosol; this osmotic gradient triggers the outflow of the sweat precursor from the cells to the lumen of the secretory coil, as shown in Figure 1c. As this fluid moves along the eccrine sweat duct, the luminal cells reabsorb ions, resulting in a hypotonic sweat solution.

### 2.5 Stimulation of the Sweat Glands and Release of Sweat

Thermoreceptors located in the preoptic area of the anterior hypothalamus send signals that trigger an autonomic response known as thermoregulatory sweating. When the core body temperature is enhanced, the thermoreceptors transmit signals via efferent neurons to postganglionic sympathetic neurons in the dermis.^[^ ^85^ ^]^ The cholinergic nerve fibers surrounding the secretory coil release acetylcholine, which simulates muscarinic G‐protein coupled receptors (GPCRs) on the membrane of the eccrine secretory cells. This stimulation enhances the levels of intracellular inositol trisphosphate, which interacts with receptors on the membrane of the endoplasmic reticulum to affect the release of Ca^2+^ into the cytosol. The protein stromal interaction molecule 1 (STIM1) monitors the levels of Ca^2+^ in the intracellular reservoir. When the Ca^2+^ stores are low, STIM1 stimulates the store‐operated entry of Ca^2+^ by attaching to and triggering the Orai Ca^2+^ channel on the plasma membrane.^[^ ^92^ ^]^ Sweat secretion is caused by the electrolyte exchange mediated by this influx of Ca^2+^ ions.

Adrenergic innervation also triggers sweat during the “fight or flight” response. The palms, soles, and axillary area are the regions where the physical manifestations of tension, worry, fear, and pain are primarily felt. This reaction may have the selective and advantageous effect of increasing palmoplantar friction during the flight response.^[^ ^63^ ^]^ The limbic system regulates “emotional” sweating and sends signals to adrenergic nerve fibers in the secretory coil of sweat glands. The α‐ and β‐adrenergic receptors of the secretory cells are stimulated by the release of adrenaline and norepinephrine during such signaling. Isoproterenol, a synthetic sympathomimetic drug that selectively stimulates β‐adrenoreceptors, has been utilized for further distinguishing between these two routes; these investigations established β‐adrenergic activation as the predominant route in emotional sweating. Additionally, sweat secretion rates indicated that sweat is secreted in response to β‐adrenergic and α‐adrenergic stimulation, as per the ratio 4:2.^[^ ^93^ ^]^ Ca^2+^ influx occurs upon α‐adrenergic stimulation in a manner akin to that observed upon cholinergic stimulation; the intracellular concentration of cAMP is increased, and adenylyl cyclase is activated by β‐adrenergic receptors of the GPCR family. The activation of the cystic fibrosis transmembrane conductance regulator (CFTR) results in the cAMP‐mediated activation of protein kinase A (PKA) and the subsequent secretion of Cl^−^ ions.^[^ ^94^, ^95^ ^]^ β‐adrenergic stimulation occurs when CFTR is either absent or defective, which leads to the restriction of Cl^−^ reabsorption and inhibition of CFTR‐dependent secretion of Cl^−^ ions, as witnessed in cystic fibrosis. The functional activity of CFTR may be evaluated utilizing a “ratiometric” sweat secretion test that compares the rates of sweat production in response to adrenergic and cholinergic stimulation.^[^ ^96^ ^]^ The sudomotor axon reflex allows the production of sweat near the edge of an area that has been activated (Figure 1). Axonal antidromic conduction toward a branch point and subsequent orthograde conduction along the branching fibers result from the interaction between nicotinic agonists and receptors on postganglionic sudomotor terminals at the base of the sweat gland. Subsequently, acetylcholine is produced at the nerve terminals and attaches itself to muscarinic receptors on the eccrine sweat gland, resulting in sweat secretion in a manner akin to that witnessed with the direct iontophoretic response.^[^ ^97^ ^]^ This sweating may spread several millimeters beyond the circumstance of the activated area.^[^ ^98^ ^]^ The sudomotor axon reflex may be employed for evaluating disorders of the autonomic nervous system, including diabetic neuropathy,^[^ ^99^ ^]^ and identifying sweat‐sampling areas that are not subjected to the influence of drug‐induced sweat stimulation, helping to prevent cross‐contamination.^[^ ^97^ ^]^ The latency of the sudomotor axon response is ≈5 s greater than that of the direct cholinergic response due to the time required for axonal conduction and neuroglandular transmission. The presence of nicotinic agonists results in the production of similar sweat volumes via the direct and sudomotor axon responses. However, the direct stimulation of sweat response can last for over an hour following the termination of the stimulation, while the sudomotor axon response returns to baseline levels within 3–5 min.^[^ ^100^ ^]^ The sweat rate is partially regulated via localized and uneven activation of the sweat glands. The sweat generated by the surrounding localized stimulation sweat glands varies significantly during mental stress.^[^ ^101^ ^]^ In healthy individuals with an average sweat gland density of 200 cm^−2^, the sweat rate varies over the range 0.2‐1 µL cm^−2^ min^−1^,^[^ ^102^, ^103^ ^]^ which corresponds to 1–5 nL gland^−1^ min^−1^. The sweat rate is also influenced by local skin temperature.^[^ ^63^ ^]^ Additionally, the pharmacological stimulation of sweating may increase the sweat rate to ≈10 nL gland^−1^ min^−1^.^[^ ^104^ ^]^ The decrease in the sweat rate and eventual termination of sweating correspond to the elimination of sweat stimulants such as acetylcholinesterase from the subcutaneous tissue.^[^ ^105^ ^]^ Variation in sweat rates among individuals is likely due to differences in the functionality and responses of sweat glands.^[^ ^106^ ^]^ Several factors can influence the sweat response, including gender, physical fitness, menstrual cycle, and circadian rhythm.^[^ ^63^ ^]^ Variations in the density and distribution of sweat glands may contribute to intra‐individual regional variations in the measured sweat rate. For instance, the forehead has the highest density of sweat glands and supports the highest sweat rate under conditions of active as well as passive thermal sweating.^[^ ^106^ ^]^

While sweat produced during exercise may possibly be evaluated using sweat sensors, continuous monitoring of the secreted sweat may not be conceivable under all circumstances. Therefore, alternative techniques are required to obtain sweat samples. Iontophoresis is a highly effective noninvasive technique for inducing sweat secretion in particular regions of the body, such as the wrist^[^ ^26^ ^]^ and involves the application of a mild electric current to the skin for the localized stimulation of sweat production.^[^ ^107^ ^]^ A small current is administered between pilogels, which are hydrogels containing the sweat‐stimulating drug pilocarpine; this drives the drug beneath the skin surface, causing sweat production from the surrounding sweat glands. Wearable sensors then collect this sweat for analysis. This approach can provide new insights into sweat secretion and broaden the scope for sweat‐based sensing applications, including in health monitoring systems and POCT.^[^ ^108^ ^]^

#### 2.5.1 Sweat Generation under Natural Conditions and in Response to Heat and Exercise

Besides natural secretion, sweat is generated in response to various conditions, including heat stimulation, physical activity, and iontophoresis. Thermal activation involves exposure to a hot atmosphere (sauna), resulting in an increase in skin temperature to 40‐41°C; thermal sweat is produced in response throughout the surface of the body at a rate of 0.6‐1 kg h^−1^.^[^ ^109^, ^110^ ^]^ Numerous studies have shown that sweating during exercise is common, but the rate of sweating can vary depending on the type and intensity of the exercise. Typical activities, such as treadmill and stationary bike workouts, allow for controlled and measured exercise intensity. The challenges associated with controlling thermal conditions and exercise during testing have prompted increasing interest in using naturally occurring sweat for downstream sensing.^[^ ^111^, ^112^, ^113^, ^114^ ^]^ The spontaneously generated sweat, also known as “background sweat,” occurs during day‐to‐day activity with a comparatively modest sweat rate, roughly 10 times lower than exercise‐induced sweat.^[^ ^113^ ^]^

#### 2.5.2 Iontophoresis‐Induced Sweat

Pilocarpine, a muscarinic cholinergic agonist, is commonly used in iontophoresis protocols to stimulate the production of eccrine sweat glands. It primarily acts by activating the M3 muscarinic acetylcholine receptors found in the secretory coil cells of sweat glands. When pilocarpine binds to these receptors, it initiates a G‐protein‐coupled signaling cascade that activates phospholipase C, generates inositol trisphosphate (IP_3_), and leads to the release of intracellular calcium (Ca^2^⁺). This process enhances secretory activity by promoting the transport of fluid and electrolytes across the glandular epithelium.

Although this direct biochemical mechanism effectively induces sweating in individuals with low or absent spontaneous sweat rates, it also introduces biochemical changes that can affect the natural composition of sweat. The stimulation of sweat glands modifies the kinetics of ion reabsorption in the ducts, often resulting in higher sodium and chloride concentrations than sweat produced under physiological conditions. Additionally, the concentrations of metabolites such as lactate, urea, and glucose may be altered due to changes in glandular metabolism and enzymatic activity. In addition, pilocarpine‐induced stimulation bypasses the thermoregulatory neural pathways that usually regulate sweat secretion, which may lead to a secretion profile that does not accurately reflect systemic physiological conditions. These changes can be worsened by the localized effects of iontophoresis, including temporary alterations in skin barrier permeability and the potential mobilization of surface contaminants into the collected sweat sample. As a result, biomarkers obtained from sweat stimulated by pilocarpine should be interpreted with caution. The cholinergic activation can distort baseline analyte levels, complicate systemic correlations, and require thorough calibration and validation to ensure diagnostic reliability.^[^ ^115^ ^]^

**Figure 3:**
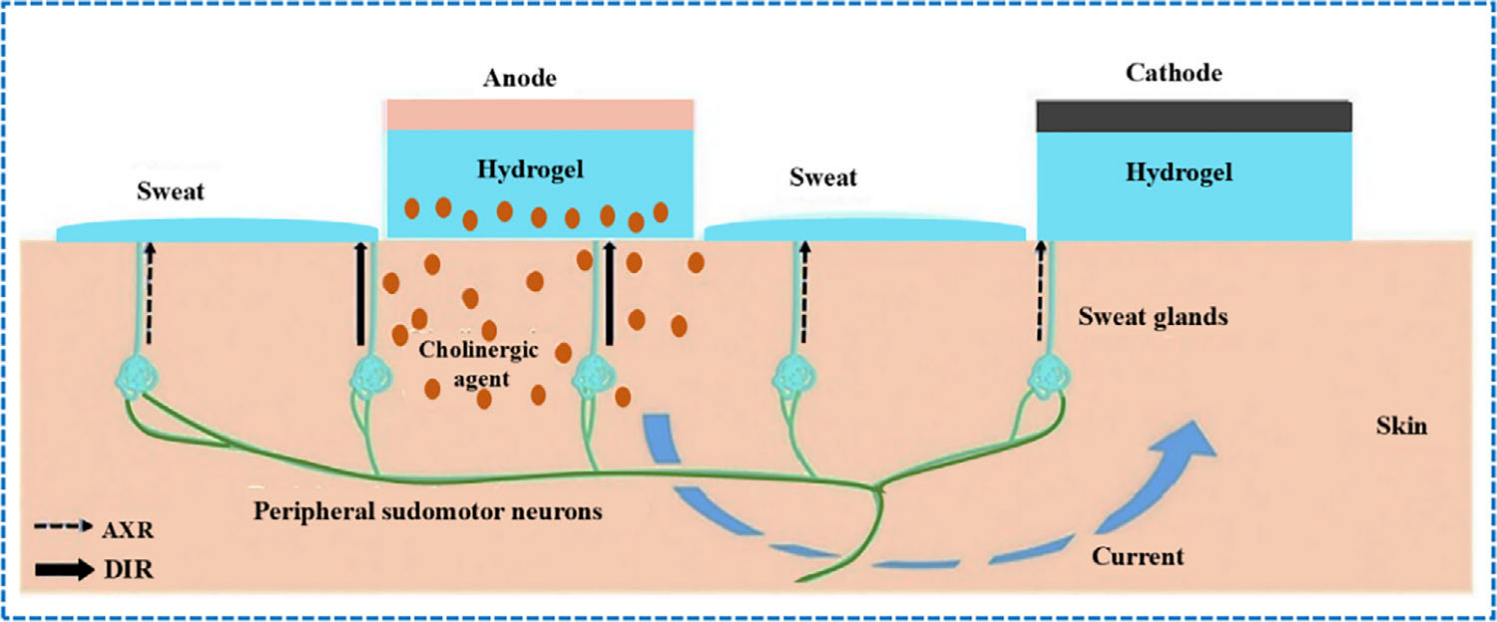
Schematic representation of sweat stimulation using iontophoresis. Sweating occurs through two primary mechanisms: AXR (axon reflex‐mediated) and DIR (direct stimulation of sweat glands). Reproduced with permission.^[^ ^74^ ^]^ Copyright 2023 ACS.

Iontophoresis is a technique in which a mild electric current is used for administering a cholinergic drug carried within a hydrogel directly into the skin, as shown in **Figure** **3**. This process involves placing two hydrogel patches on the skin. The cathode and anode hydrogels contain electrolytes and the cholinergic drug, respectively, to facilitate electrical conductivity (Figure 3). The cholinergic agent's activation of the muscarinic 3 (M3) sensor in sweat glands results in a direct sweat response. Moreover, the drug's specific targeting of nicotinic receptors can induce sweating via the peripheral sudomotor axon reflex.^[^ ^97^ ^]^

#### 2.5.3 A Wearable Device for Iontophoresis‐Based Induction of Sweating

**Figure 4:**
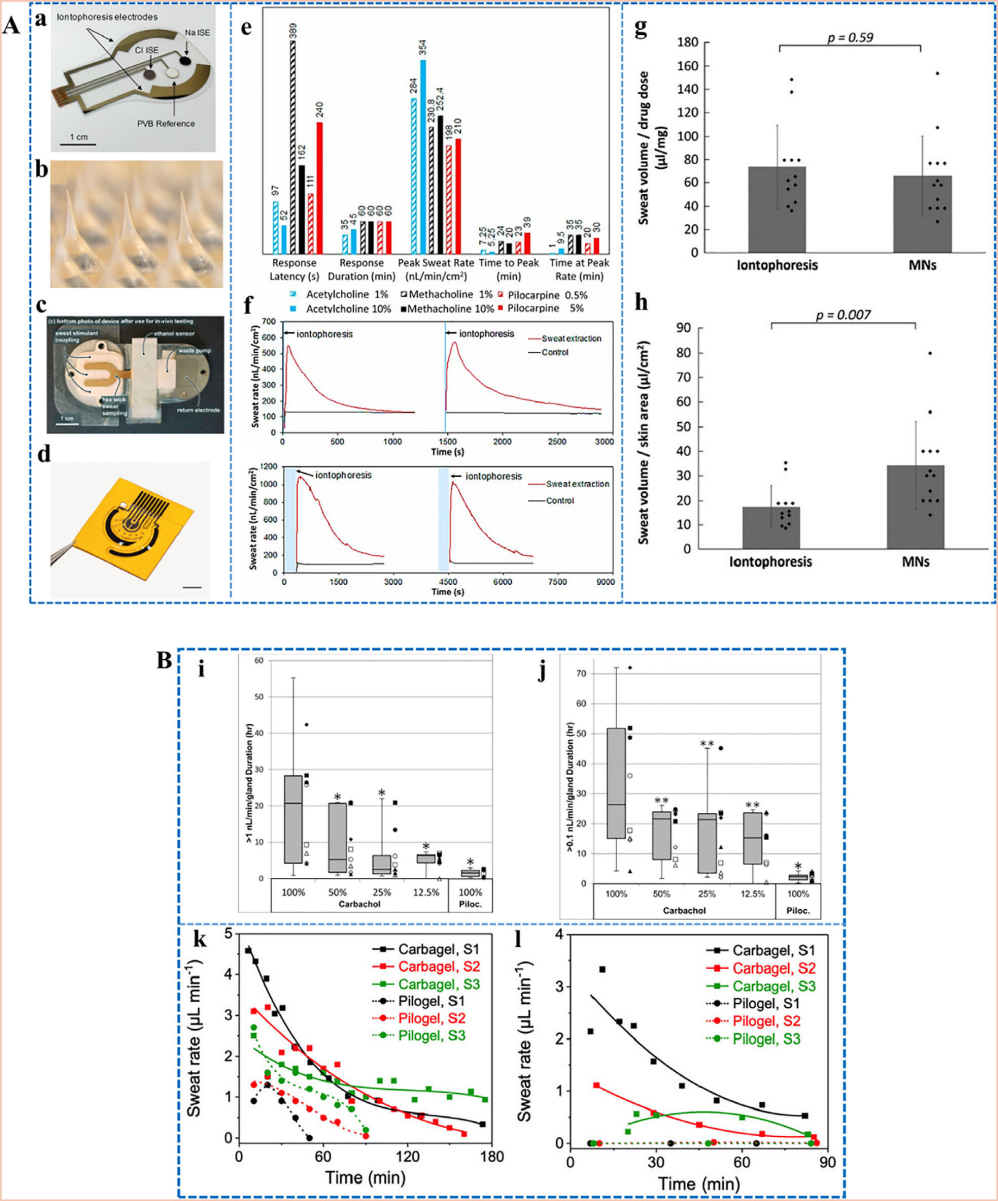
Illustration of iontophoresis‐driven sweat stimulation approaches. **A**. a) Image of an iontophoresis patch used for sweat induction. b) Design of a microneedle‐enabled iontophoresis platform, c) A carbachol‐based sweat‐sensing system utilizing iontophoresis. d) A soft, flexible sensing patch fabricated via laser engraving. e,f) Sweat secretion profiles following the application of different cholinergic stimulants. g,h) Microneedle‐enabled iontophoresis platform and its sweat‐inducing performance in vivo. **B**. i,j) Comparative durations of sweat generation under high and low conditions and secretion rates following iontophoresis with carbachol and pilocarpine, respectively. k,l) Measurement of localized sweat output from the directly stimulated site (k) and adjacent skin area (l) after 5 min of pilocarpine and carbachol iontophoretic activation. Scale bar represents 5 mm. Reproduced with permission.^[^ ^74^ ^]^ Copyright 2023 ACS.

Several wearable devices based on iontophoresis have been developed for inducing sweat over the past decade. The Food and Drug Administration of the United States of America (USA) has approved the commercial use of the Macroduct system, which uses pilocarpine as a cholinergic agonist, for iontophoresis‐based induction of sweating for the diagnosis of cystic fibrosis. After an initial increase in the current, a steady current is applied via pilocarpine gel disks attached to the arm. The current is gradually reduced, and the device turns off after 5 min. While it offers adjustable straps for stimulating various parts of the limbs and accommodates different age groups from infants to adults, the system remains too bulky for everyday use. Similar setups consisting of an iontophoresis device paired with custom‐made hydrogels that contain specific cholinergic agonists can be constructed for prototype testing.^[^ ^104^ ^]^ A flexible wristband with electrodes for iontophoresis and an integrated flexible printed circuit board (FPCB) was designed to enhance wearability, as shown in **Figure** **4**a.^[^ ^108^ ^]^ The sweat response to iontophoresis gels infused with various cholinergic agonists such as acetylcholine, methacholine, and pilocarpine was analyzed by evaluating factors such as response time, duration of sweating, peak sweat rate, time taken to attain the peak rate, and duration of maintenance of the peak rate, as shown in Figure 4e. Additionally, periodic iontophoresis using various concentrations of acetylcholine showed that the gel containing 10% acetylcholine elicited a higher sweat rate and extended duration of sweating compared to the gel containing 1% (Figure 4f). In addition to the gel‐based wearable systems,^[^ ^116^ ^]^ a microneedle patch loaded with pilocarpine was recently developed for sweat testing.^[^ ^117^ ^]^ The iontophoresis patch containing an array of 100 microneedles (each measuring 600 µm in length; Figure 4b) is significantly smaller and thinner than a standard commercial gel‐based iontophoresis electrode. A study employing horses revealed that the microneedle patch generated more sweat per area than the pilocarpine‐containing hydrogel (Figure 4g,h). Previous studies focused on localized sweating have been reported; however, this research employed carbachol for the first time to extract sweat using a band‐aid‐shaped system connected with an external iontophoresis device (Figure 4c).^[^ ^118^, ^119^ ^]^ Additionally, sweating was monitored over an extended duration (>10 h) in the aforementioned study, and the sweat response to custom‐made carbachol gels was compared to that obtained with pilocarpine gels at both high (Figure 4i) and low (Figure 4j) sweat rates. A compact, flexible iontophoresis patch was recently employed for fabricating laser‐engraved graphene electrodes containing tiny carbachol gels. The patch and an integrated FPCB enabled on‐demand sweat induction (Figure 4d).^[^ ^120^ ^]^ Sweat rates at the activated area and adjacent skin were measured after 5 min of iontophoresis with pilocarpine and carbachol (Figure 4k,l). Variations in the current profile and innovative form factors were investigated to increase sweat volume. A current profile consisting of sinusoidal pulses was suggested for patients with sweat issues.^[^ ^121^ ^]^

However, this technique holds promise for non‐invasive drug delivery and biomarker extraction, but it faces several real‐world usability challenges that limit widespread adoption. Prolonged application of low‐level currents (0.5‐5 mA) can cause skin irritation, erythema, or mild burns due to local pH shifts and electrochemical reactions at the electrode‐skin interface. This raises safety and comfort concerns, especially for sensitive patients. Additionally, these devices are energy‐intensive, requiring sustained current that quickly depletes small batteries. This makes portable or wearable designs less practical without bulky power sources or the need for frequent recharging. Practical issues such as electrode misalignment, poor adhesion on sweaty or hairy skin, and user discomfort from tingling sensations further reduce compliance in home‐care settings. Moreover, the small volumes of analytes extracted (glucose or lactate) often fall below reliable detection thresholds, undermining diagnostic accuracy. For instance, while clinical glucose monitoring via iontophoresis has shown promising results in controlled trials, it remains inconsistent in daily use due to variability in skin properties and extraction efficiency. Therefore, despite their technical potential, iontophoresis devices require significant improvements in biocompatibility, power management, and standardization before they can achieve reliable, user‐friendly clinical applications.^[^ ^26^, ^122^ ^]^

## 3 Analysis of Variability in Sweat Production

### 3.1 Intrapersonal and Interpersonal Variations in Sweat

Significant variations are observed in the composition and production of sweat within the same individual (intrapersonal) and among different individuals (interpersonal). Intrapersonal variations are influenced by factors such as hydration status, physical activity, diet, environmental factors, and daily rhythms, which lead to changes in sweat rate and biomarker concentrations. The interpersonal variations are attributable to age, gender, fitness level, genetic predisposition, and sweat gland density. For instance, males generally produce higher sweat volumes, resulting in a dilution of biomarkers, whereas females often produce sweat with a higher concentration of analytes. These variations complicate the standardization and reliability of sweat as a diagnostic biofluid, especially for systemic applications.

#### 3.1.1 Regional Versus Whole‐Body Sweating

**Figure 5:**
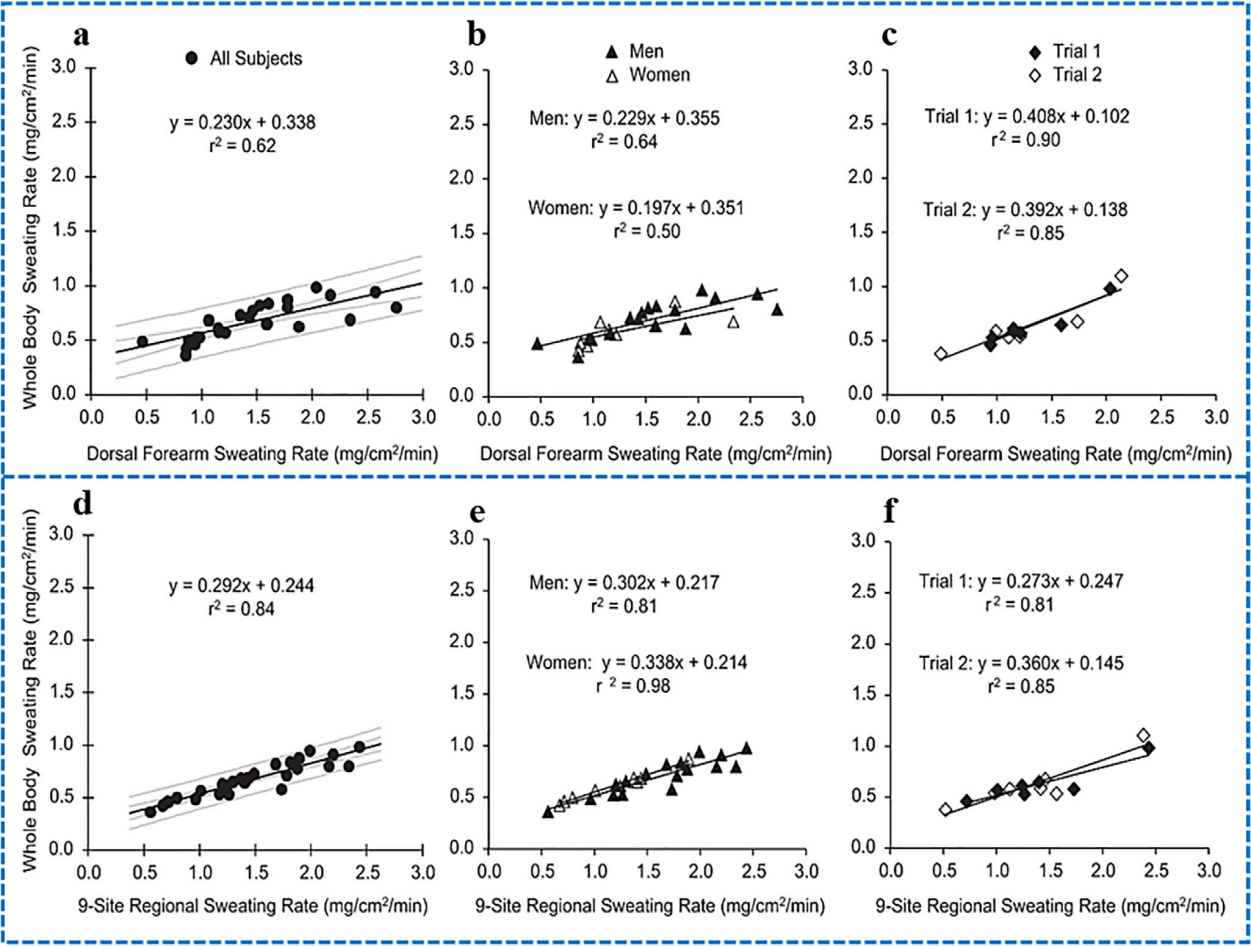
Linear regression plots illustrate the relationship between regional and whole‐body sweating rates for the dorsal forearm a–c) and a combined 9‐site measurement d–f). a and d display regression lines derived from the full participant group (n =26). Panels b and e show separate regression analyses for male (n = 17) and female (n = 9) participants. c and f shows regression results from trial 1 and trial 2 in the repeatability assessment (subset of n = 7). Black lines denote the regression lines, while the outer and inner gray lines represent the observed and mean 95% confidence intervals, respectively, for a and d. Reproduced with permission.^[^ ^123^ ^]^ Copyright 2018 American Chemical Society.

Baker et al. reported a strong correlation between regional (at various specific sites) and whole‐body sweat rates (*P* < 0.01). However, the regional sweat rates at the dorsal and ventral forearms, lower back, scapula, and forehead led to significant overestimation of the whole‐body sweat rate with varying levels of statistical significance (*P* < 0.05 to *P* < 0.0001). While the average values for regional sweat rate at other sites were typically higher than the whole‐body sweat rate, these differences were not statistically significant (P ≥ 0.05). Every regression model for predicting the whole‐body sweat rate from regional sweat rates exhibited slopes and intercepts that notably differed from 1 and 0, respectively. The R^2^ values ranged from 0.34 for the lower back to 0.84 for the aggregate values from nine sites. **Figure** **5** presents the linear regression analysis for regional versus whole‐body sweat rates at the dorsal forearm and the aggregate values across the nine sites. These locations are highlighted because the dorsal forearm is one of the most frequently used sites for field sweat testing, while the nine‐site aggregate provides a comprehensive overview of all regional sites. A comparison between regional and whole‐body sweat rates is presented in **Table** **1**.^[^ ^123^ ^]^

**Table 1:** Detailed comparison between regional and whole‐body sweat rates.

S.No	Area	Ratio of RSR To WBSR	Mean Sweat Rate, mg∙cm^−2^∙min^−1^	Pearson's Correlation Coefficient (r value) between RSR and WBSR
1	Whole body	‐	0.677 ± 0.167	‐
2	Triceps	1.639 ± 0.519	1.150 ± 0.555	0.83*
3	Lower back	2.549 ± 0.732	1.715 ± 0.635*	0.58*
4	Dorsal forearm	2.147 ± 0.532	1.476 ± 0.571*	0.79*
5	Scapula	2.686 ± 0.719	1.853 ± 0.746*	0.77*
6	Forehead	6.622 ± 3.594	4.720 ± 3.036*	0.66*
7	Chest	1.984 ± 0.579	1.401 ± 0.598	0.74*
8	Ventral thigh	1.437 ± 0.370	0.973 ± 0.320	0.74*
9	Calf	1.097 ± 0.345	0.753 ± 0.339	0.70*
10	Nine‐site	2.137 ± 0.385	1.480 ± 0.524*	0.92*
11	Ventral forearm	2.188 ± 0.502	1.478 ± 0.488*	0.72*

Values represent means ± standard deviation (SD); n = 26 subjects for all sites except chest, for which n = 25; * denotes *P* < 0.05; regional sweat rate (RSR) vs. whole‐body sweat rate (WBSR).

#### 3.1.2 Flow Rate of Sweat in Regional Body Parts

**Figure 6:**
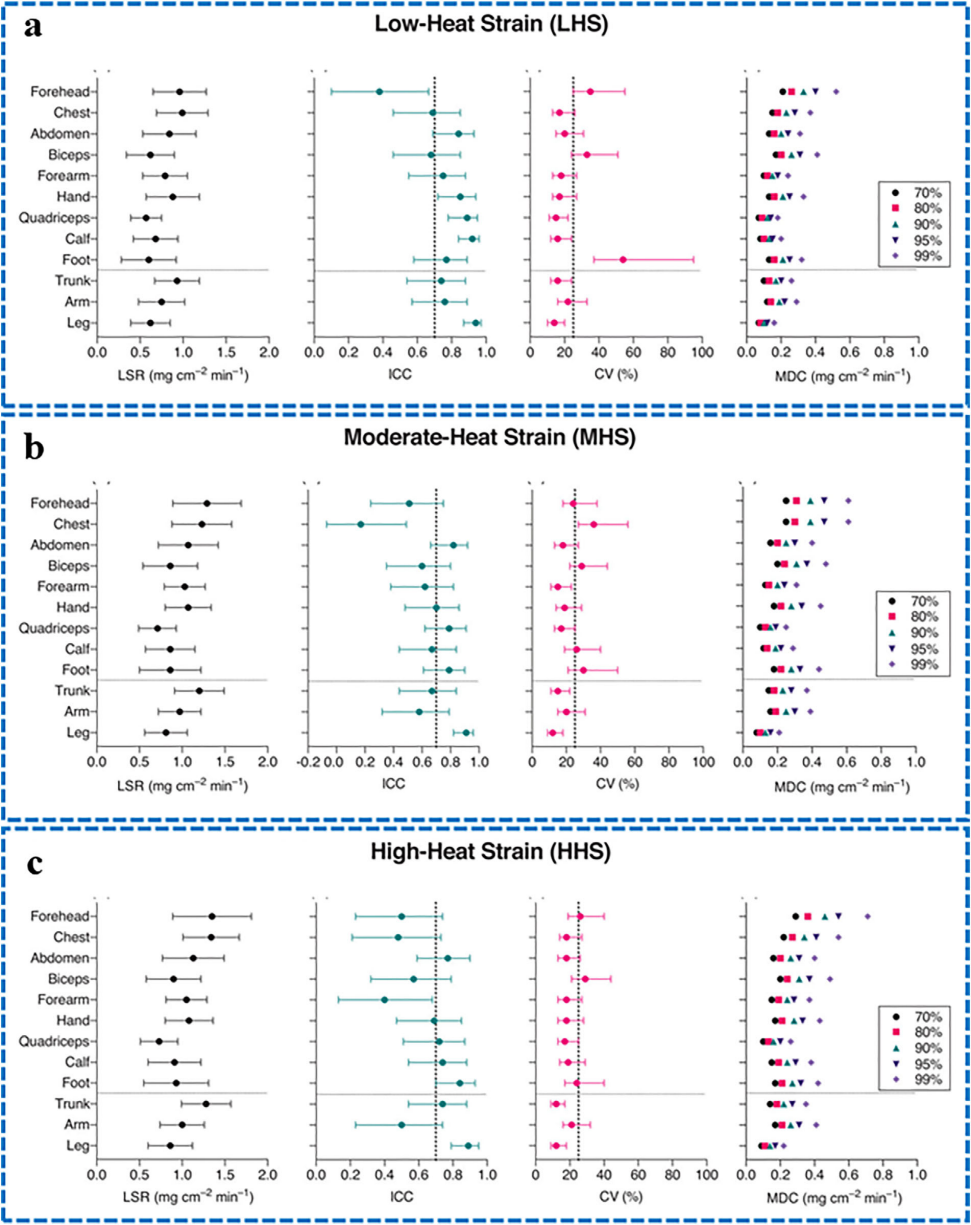
Overview of local sweat rate (LSR) and its reliability across body sites during passive heating at different heat strains. Low‐heat strain a), Moderate‐heat strain b), and High‐heat strain c). Mean LSR, intra‐class correlation coefficient (ICC), coefficient of variation (CV), and minimum detectable change (MDC) are shown for arm, leg, and trunk regions. LSR is reported as mean (SD); reliability metrics include mean and 95% CI. Dotted lines mark thresholds for acceptable reliability (ICC ≥ 0.70, CV < 25%). The regions include the arm (biceps, forearm, and hand), the leg (quadriceps, calf, and foot), and the trunk (forehead, abdomen, and chest). Reproduced with permission.^[^ ^124^ ^]^ Copyright 2020 Wiley.

The individual sites of the body were categorized into three regions, including trunk (abdomen, chest, and forehead), arm (biceps, forearm, and hand), and leg (quadriceps, calf, and foot), as shown in **Figure** **6**. This was done to determine whether reliability can be improved by obtaining the averages for the relevant sites without weighting. The absolute reliability exhibited by each region was considerable (14‐22%), although one site in each region failed to meet this threshold on its own (Figure 6a). Furthermore, relative reliability for the trunk and leg was improved at low, medium, and high‐heat settings, while absolute reliability increased across all regions at medium and high‐heat settings (Figure 6b,c).

**Figure 7:**
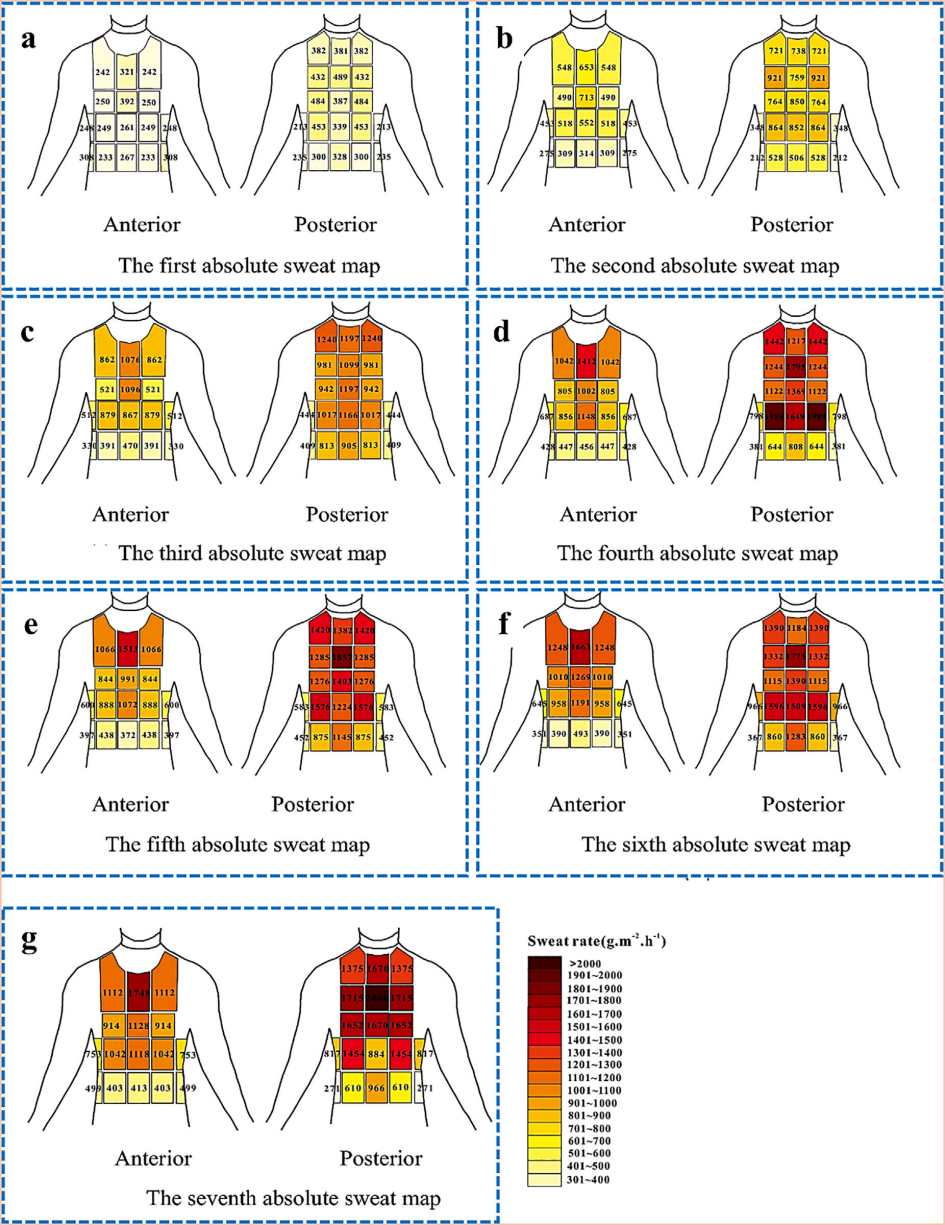
Absolute RSR of participants wearing FCPE. The first a), second b), third c), fourth d), fifth e), sixth f), and seventh g) absolute sweat maps were drawn based on the quantities of sweat collected from the upper body over the durations 0‐–5, 9‐–14, 18‐–23, 27‐–32, 36‐–41, 45‐–50, and 54‐–59 min, respectively. Reproduced with permission.^[^ ^125^ ^]^ Copyright 2022 Nature.

**Figure** **7** shows that the sweat rate in the posterior region is higher than in the anterior region. The maximum sweat rate among the anterior zones was found in the medial upper chest. Additionally, the medial and upper areas of the lower back showed greater sweat rates, which aligns with observations made in previous studies that the central upper or lower back exhibited the highest regional sweat rates (RSR). In addition, the waist and buttock areas showed the lowest sweat rate. Previous studies also reported a decrease in sweat rate from the center to the side areas of the front upper body. In the back region, however, the side areas had a higher sweat rate than the central regions; these differences may be attributable to differences in the experimental methodology, the sizes of the sweat‐sampling area, or errors in sweat collection due to the concave spinal curvature. The sweat rates from the third sweat map (corresponding to a heart rate of 121–148 beats per minute or bpm) were compared with those observed by Smith and Havenith (target heart rate of 125–135 bpm). Similarly, sweat rates from the seventh sweat map (corresponding to heart rates of 144–169 bpm) were compared to those obtained by Smith and Havenith for the second‐intensity level (target heart rate set at 150–160 bpm).

### 3.2 Sweating Behavior under Different Environmental Conditions

Environmental conditions such as humidity, wind speed, and air pressure influence the sweat secretion rate. These factors may cause the evaporation of sweat from the skin and also promote sweat generation at a specific average body temperature.^[^ ^126^ ^]^ The whole‐body and regional (in specific regions: forehead, hands, etc.) sweat rates increase in response to heightened environmental heat stress, including higher air temperatures, greater solar radiation, and lower air velocity, under a constant workload; these conditions also lead to changes in sweat composition. Prolonged exposure to humid air can suppress sweating, leading to reduced sweat generation. Additionally, an increase in the ambient temperature results in elevated Na+ and Cl− ions concentrations in the sweat.^[^ ^127^ ^]^ Hidromeiosis affects sweat production once the skin is fully saturated with sweat, and the ensuing reduction in sweat rate is proportional to the sweat rate at the beginning of the process. Seasonal adaptations, including acclimatization to heat in the summer and cold in the winter, impact the threshold for hidromeiosis. Importantly, hidromeiosis appears to cause cessation of sweating under humid rather than dry conditions.^[^ ^126^ ^]^

### 3.3 Sweating Behavior at Different Body Temperatures

**Figure 8:**
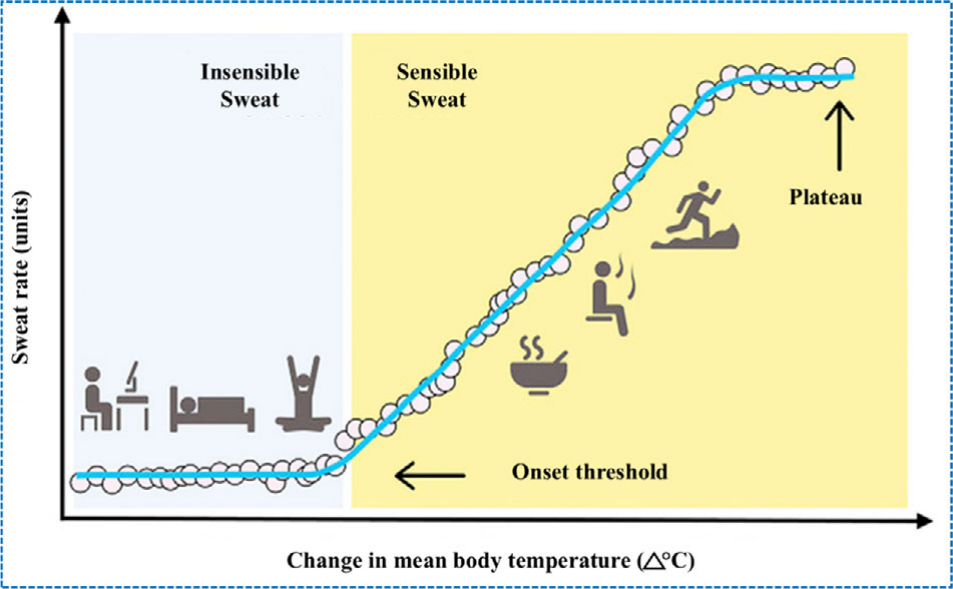
Association between change in mean body temperature and sweat rate for achieving thermoregulation. Insensible sweat is the main type generated within the onset threshold (during activities such as office work, sleeping, or yoga), where a relatively flat line characterizes the association. Beyond the onset threshold (consumption of hot food, exposure to sauna, or running), sensible sweat begins to be secreted. It becomes the predominant form of sweat, characterized by a linear association. Ultimately, sensible sweat rate reaches a maximum level, leading to a plateau despite mounting mean body temperature. Reproduced with permission.^[^ ^132^ ^]^ Copyright 2017 Wiley.

The regulation of sweating behavior is essential for thermoregulation, which stably maintains the body's core temperature within a narrow range of 36–38°C under conditions of rest and 41°C during intense physical activity. This regulation is essential for maintaining normal physiological functions and ensuring organ systems operate efficiently under varying thermal conditions.^[^ ^128^ ^]^ The evaporation of sweat is crucial for regulating body temperature as it helps in heat loss. Thermoregulation is achieved by balancing the core body temperature with the skin surface, which constitute the overall body temperature.^[^ ^129^ ^]^ Under conditions of extremely mild activity, such as resting, sleeping, or engaging in low‐intensity activities, the body primarily loses heat through radiation, relying on a consistent level of insensible sweat to keep the body temperature stable. However, when the body is subjected to higher thermal stress that surpasses a certain threshold, it starts producing sensible sweat that is secreted onto the skin surface. The evaporation of sweat aids in cooling, as the latent heat from insensible sweat alone is insufficient for regulating body temperature. **Figure** **8** briefly explains the association between the change in mean body temperature and sweat rate during this process.^[^ ^81^, ^129^, ^130^ ^]^ Heat‐related stressors such as intense physical activity, exposure to passive heat, and consuming hot and spicy foods can trigger the sensible sweat response.^[^ ^63^, ^131^ ^]^ Heat balance is achieved as the secretion rate of sensible perspiration increases linearly with body temperature. In this instance, water evaporation from sensible sweat provides the latent heat necessary for efficiently cooling the body.^[^ ^81^, ^129^ ^]^

### 3.4 Differences in Sweating Behavior with Age

The variations in regional sweat rates between the right and left sides of the body range from 11 to 54 g m^−2^ h^−1^ during various sports. In a group of older individuals (> 50), the right posterior lower arm showed a substantially greater regional sweat rate than that of the left during both rest (*P* < 0.05) and exercise (*P* < 0.05). In the group of young individuals at rest, higher regional sweat rates were observed at the right shoulder (*P* < 0.05), right side (*P* < 0.05), and outer upper leg (*P* < 0.05) compared to the regions on the left side. During exercise, this difference was also noted on the front (*P* < 0.05) and back sides of the lower arm (*P* < 0.05) as well as the outer lower leg (*P* < 0.05). Following Bonferroni corrections, the posterior lower arm was the only region from the group of young individuals where right‐to‐left variations were noticeable during the exercise session. Given that these variations only occurred in a minor proportion of all the areas examined, classifying right and left regional sweat rates was deemed suitable for analyzing data across age groups. In keeping with the observations of Smith and Havenith's study, this also minimized the number of comparisons across areas.^[^ ^133^, ^134^ ^]^

**Figure 9:**
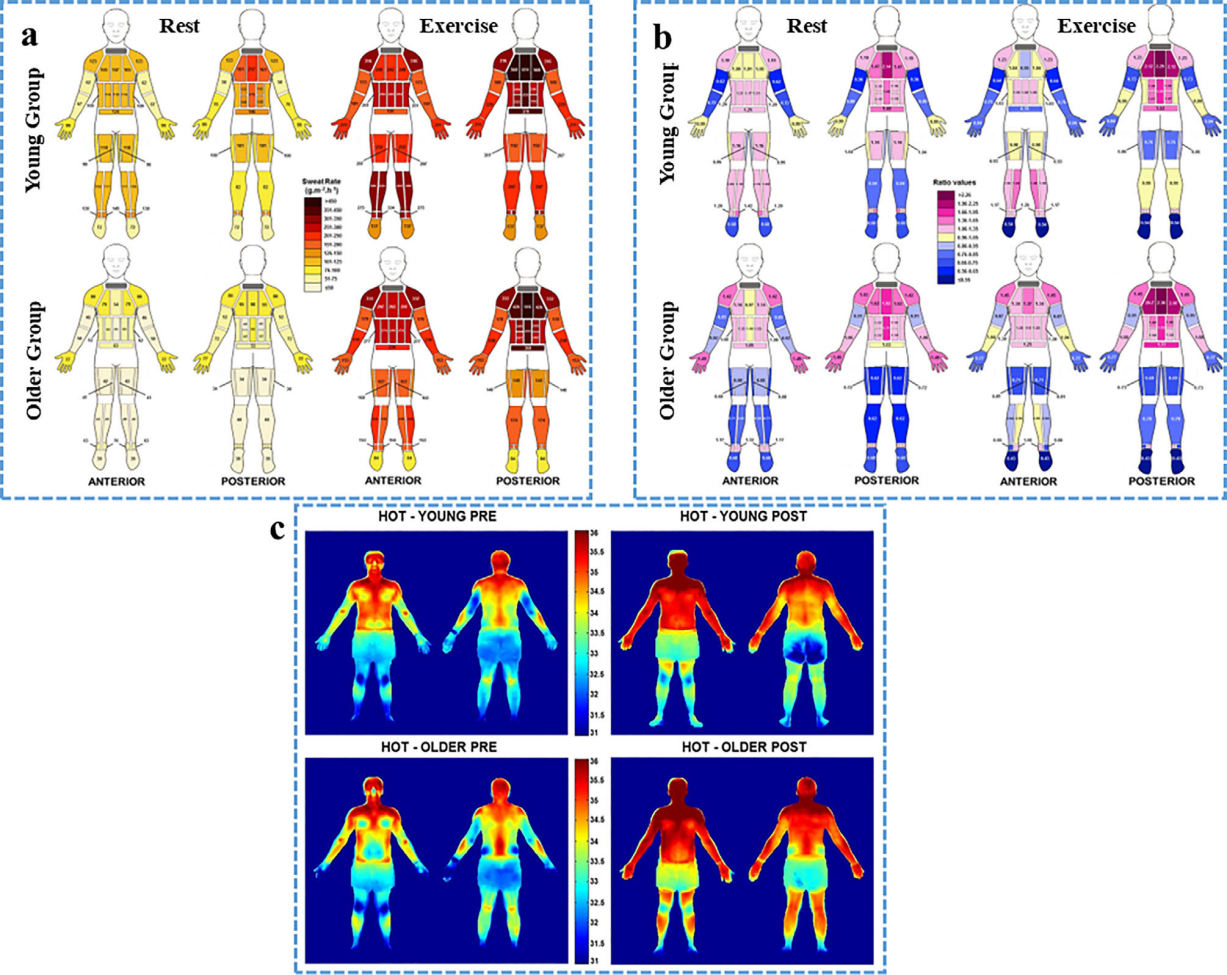
a) Whole‐body sweat maps of absolute regional median sweat rates (g m^−2^ h^−1^) during periods of rest and exercise (32°C and 50% RH) in young (18–30 years) and older (60‐80 years) individuals. b) Body maps illustrating normalized regional median sweat rates (RSR) under the same conditions and age groups. Normalization was done by dividing local sweat rates by the surface area‐weighted whole‐body sweat rate for everyone. Values greater than 1 indicate above‐average sweating, 1 represents the average, and values below 1 indicate below‐average sweating. c. Group‐averaged body maps of absolute skin temperature (°C) before and after the rest period at 32 °C and 50% RH for young (n = 10) and older (n = 10) participants. The temperature scale is centered around the mean (≈34 °C), with a ±4 °C range. Reproduced with permission.^[^ ^137^ ^]^ Copyright 2021 Springer Nature.

The whole‐body sweat maps depict the average data for the two age groups during rest and exercise, as shown in **Figure** **9**. Descriptive statistics of regional sweat rates during both periods can be found online. Under resting conditions (passive heating), the younger participants showed higher regional sweat rates across all body regions. However, these differences were statistically significant in only 20 of 28 areas, including the torso, legs, and feet (*P* < 0.05). After applying Bonferroni corrections, the younger participants exhibited significantly greater regional sweat rates only in the lower body regions (10 areas, including the legs, ankles, and feet). During exercise, the regional sweat rates were notably higher in 11 of 28 regions, including the hands (*P* < 0.05) and all areas of the lower body (*P* < 0.05), in younger individuals compared to those in the older group. However, significant differences were found only at the lateral ankle and feet (*P* < 0.01) after the application of Bonferroni corrections. The regional sweat rates during exercise were significantly greater than those at rest across all body regions in both age groups (P ≤ 0.001), which was maintained even after applying Bonferroni corrections.^[^ ^75^, ^77^, ^78^, ^135^, ^136^ ^]^

### 3.5 Gender‐Based Differences in Sweat Rates

The sweat rates for men and women are presented in **Table** **2**. Significant differences in sweat rates can be observed based on the region (*P* < 0.001), gender (*P* < 0.001), and region gender interaction (*P* < 0.001). Tukey's posthoc analysis revealed significant differences in sweat rates between men and women only at the forehead area (5.931 ± 3.005 vs. 2.433 ± 1.319 mg cm^−^^2^ min^−1^, *P* < 0.0001) and in the ratio of forehead and whole‐body sweat rates (WSBR) (8.068 ± 3.522 vs. 3.892 ± 1.650, *P* < 0.0001). Strong correlations were observed between sweat rates in specific areas of the body and the overall sweat rate for both men and women (r‐values of 0.63–0.99, *P* < 0.05), except for the lower back in men (r = 0.09). The regression model employed for predicting whole‐body sweat rate from RSR did not reveal significant effects of gender (P > 0.05) or the interactions between gender and regional sweat rates (P > 0.05) across any of the measured body sites.^[^ ^123^ ^]^

**Table 2:** Whole‐body and regional sweat rates in men and women.

S.No	Area	Sweat rates in women [mg cm^−2^ min^−1^]	Sweat rates in men [mg cm^−2^ min^−1^]	Ratio of RSR to WBSR in women	Ratio of RSR to WBSR in men	P‐values for the Influence of Region–gender Interactions on the Regression Model for Predicting WBSR Based on RSR
1	Whole body	0.610 ± 0.139	0.712 ± 0.173	‐	‐	‐
2	Forehead	2.433 ± 1.319	5.931 ± 3.005	3.89 ± 1.65	8.07 ± 3.52	Gender: 0.63 Sex‐by‐RSR: 0.35
3	Ventral forearm	1.240 ± 0.357	1.603 ± 0.510	2.03 ± 0.34	1.96 ± 0.79	Gender: 0.53 Sex‐by‐RSR: 0.57
4	Dorsal forearm	1.313 ± 0.497	1.562 ± 0.603	2.13 ± 0.53	2.15 ± 0.55	Gender: 0.98 Sex‐by‐RSR: 0.72
5	Lower back	1.556 ± 0.775	1.800 ± 0.556	2.46 ± 0.68	2.60 ± 0.77	Gender: 0.49 Sex‐by‐RSR: 0.76
6	Triceps	0.916 ± 0.306	1.273 ± 0.623	1.50 ± 0.41	1.71 ± 0.57	Gender: 0.64 Sex‐by‐RSR: 0.71
7	Scapula	1.509 ± 0.590	2.036 ± 0.771	2.41 ± 0.53	2.83 ± 0.78	Gender: 0.44 Sex‐by‐RSR: 0.47
8	Ventral thigh	0.866 ± 0.374	1.030 ± 0.282	1.38 ± 0.43	1.47 ± 0.35	Gender: 0.66 Sex‐by‐RSR: 0.45
9	Nine‐site	1.171 ± 0.405	1.644 ± 0.515	1.87 ± 0.26	2.28 ± 0.37	Gender: 0.98 Sex‐by‐RSR: 0.59
10	Chest	1.127 ± 0.425	1.555 ± 0.636	1.82 ± 0.48	2.27 ± 0.56	Gender: 0.38 Sex‐by‐RSR: 0.57
11	Calf	0.731 ± 0.349	0.765 ± 0.344	1.14 ± 0.31	1.07 ± 0.37	Gender: 0.25 Sex‐by‐RSR: 0.65

RSR, regional sweat rate;

WBSR, whole‐body sweat rate.

### 3.6 Analysis and Relevance of Correlation Between Biofluid Biomarkers and Systemic Health Parameters

The analysis of the correlation between biofluid biomarkers and systemic health parameters is crucial for establishing the reliability of the noninvasive methods of diagnosis. The effective clinical application of the biofluids sweat and ISF is dependent on obtaining an in‐depth understanding of how the levels of their biomarkers reflect systemic physiological states, such as blood glucose or electrolyte balance. A strong correlation enhances the predictive power of these biofluids, enabling accurate monitoring of chronic conditions, metabolic states, and therapeutic responses. Sweat biomarkers often exhibit weaker and variable correlations with plasma levels due to external influences, whereas ISF shows strong and consistent correlations owing to its plasma‐like composition (see **Table** **3**), making it a more reliable biofluid for systemic diagnostic applications.

**Table 3:** Biomolecule concentration in ISF to clinical relevance compared with sweat and blood.^[^ ^248^ ^]^

S.No	Biomolecule	Sweat [µM]	ISF [µM]	Blood [µM]	Clinical relevance
1	Glucose	10–1000	3900–6900	3900–6900	Diabetes, metabolic disorders
2	Sodium	10–90 × 10^3^	135–145 × 10^3^	135–145 × 10^3^	Hydration, electrolyte balance
3	Chloride	10–70 × 10^3^	98–107 × 10^3^	98–107 × 10^3^	Cystic fibrosis
4	α‐Amylase	714 × 10^−5^–714 × 10^−3^	714 × 10^−5^–714 × 10^−3^	2.86 × 10^−6^–1.0 × 10^−5^	Pancreatic disorders, stress
5	Urea	2–10 × 10^3^	2.5–7.1 × 10^3^	2.5–7.1 × 10^3^	Kidney diseases
6	Albumin	1.50–75.19	300.75–451.13	526.32–751.88	Liver/kidney diseases, Nutritional
7	Creatinine	50–200	60–100	60–100	Kidney diseases
8	Zinc	0.4–1.2	10–18	10–18	Deficiency (immune/metabolic disorders)
9	Copper	0.3–1	11–22	11–22	Wilson's disease, an antioxidant deficiency
10	Iron	0.1–1	10–30	10–30	Anemia, hemochromatosis
11	Bicarbonate	1–40 × 10^3^	22–29 × 10^3^	22–29 × 10^3^	Acid‐base balance, metabolic disorders
12	Phosphate	0.1–1 × 10^3^	0.8–1.4 × 10^3^	0.8–1.4 × 10^3^	Bone/renal disorders
13	Amino acids	1–10 × 10^3^	2–5 × 10^3^	2–5 × 10^3^	Metabolic disorders, malnutrition
14	Cholesterol	0.01–0.1 × 10^3^	3.9–5.2 × 10^3^	3.9–5.2 × 10^3^	Cardiovascular disease
15	Triglycerides	0.01–0.1 × 10^3^	0.4–1.8 × 10^3^	0.4–1.8 × 10^3^	Cardiovascular disease
16	Testosterone	0.0347–3.47	10.4–34.68	10.4–34.68	Hypogonadism, infertility
17	Estradiol	3.67 × 10^−6^–3.67 × 10^−4^	3.67 × 10^−5^–1.84 × 10^−4^	3.67 × 10^−5^–1.84 × 10^−4^	Hormonal imbalances
18	Progesterone	0.318–3.18	0.636–63.6	0.636–63.6	luteal function, pregnancy monitoring
19	C‐reactive protein	0.00087–0.0870	0.00087–0.0870	0.00087–0.0870	Inflammation, infection
20	Interleukin‐6	4.76 × 10^−9^–4.76 × 10^−7^	0–4.76 × 10^−7^	0–4.76 × 10^−7^	Inflammation
21	Tumor necrosis factor‐α	5.88 × 10^−9^–5.88 × 10^−7^	0–1.18 × 10^−6^	0–1.18 × 10^−6^	Inflammation
22	Leptin	0.00625–0.625	0.0625–6.25	0.0625–6.25	Obesity, metabolic regulation
23	Adiponectin	0.00333–0.333	0.0667–1.0	0.0667–1.0	Insulin resistance, diabetes
24	Ghrelin	3.03 × 10^−6^–3.03 × 10^−5^	3.03 × 10^−5^–3.03 × 10^−4^	3.03 × 10^−5^–3.03 × 10^−4^	Appetite hormone, nutritional
25	Melatonin	4.30 × 10^−6^–4.30 × 10^−5^	4.30 × 10^−5^–2.15 × 10^−4^	4.30 × 10^−5^–2.15 × 10^−4^	Circadian rhythm
26	Serotonin	0.568–5.68	283.7–1134.9	283.7–1134.9	Neurological diseases
27	Dopamine	0.653–6.53	6.53 × 10^−5^–6.53 × 10^−4^	6.53 × 10^−5^–6.53 × 10^−4^	Parkinson's disease, psychiatric disorders
28	Epinephrine	0.0546–0.546	5.46 × 10^−5^–5.46 × 10^−4^	5.46 × 10^−5^–5.46 × 10^−4^	Cardiovascular
29	Vitamin C	5.68–56.78	22.71–113.54	22.71–113.54	Scurvy diagnosis
37	Folate	2.27–22.66	4.53–45.33	4.53–45.33	Megaloblastic anemia
38	Thyroid‐stimulating hormone	3.57 × 10^−4^–3.57 × 10^−3^	0.0143–0.143	0.0143–0.143	Thyroid disorders (hypo‐/hyperthyroidism)

**Correlation Analysis**: The regional sweat rates at specific body sites exhibited strong correlation with WBSRs, with values of Pearson's correlation coefficient (r) ranging from moderate (0.58) for the lower back to high (0.92) for the aggregate of nine sites. The regional sweat rates from certain regions, such as the dorsal forearm, led to a significant overestimation of the whole‐body sweat rate, indicating variability in how different areas contribute to overall sweating.

**Gender‐based Differences**: Strong correlations between the regional and whole‐body sweat rates across various body areas were exhibited by both men and women, with r‐values ranging from 0.63 to 0.99 (*P* < 0.05). However, the exceptions with significant variability included specific areas such as the lower back in men, which exhibited low correlation (r = 0.09).

**Age‐dependent Variations**: The regional sweat rates were higher in younger than in older individuals, with significant differences observed in the legs and feet during rest and exercise (*P* < 0.05). These differences were intensified during exercise, with the younger group exhibiting a stronger correlation between the regional and whole‐body sweat rates; these observations emphasize the age‐related physiological changes in the distribution and efficiency of the sweat response.

**Variations Dependent on Environmental and Physiological Conditions**: Environmental factors such as heat, humidity, and exercise intensity influenced the correlation between regional and whole‐body sweat rates. Increased environmental heat stress was associated with higher sweat rates and altered regional contributions.

**Reliability and Predictive Models**: The regression models that predicted whole‐body sweat rates from regional sweat rates exhibited slopes that deviated from one and intercepts that differed from 0. This suggests a need for adjustments that account for the over‐ or underestimation of sweat rates based on specific regions. The r^2^ values varied widely, ranging from 0.34 for the lower back to 0.84 for the nine‐site aggregate, reflecting different extents of predictive reliability across various regions.

### 3.7 Sweat Variability Impacts on Real‐World Device Performance

Sweat‐based diagnostic platforms face considerable limitations in real‐world applications due to the inherent variability in sweat secretion and composition. As discussed in previous sections, sweat production is influenced by multiple extrinsic and intrinsic factors, including ambient temperature, physical activity, hydration status, emotional stress, and inter‐individual physiological differences. Furthermore, the biochemical composition of sweat is highly variable across intra‐ and inter‐personal body locations and time, with analyte levels (glucose, lactate, electrolytes, cortisol) often deviating due to sweat rate‐dependent dilution effects, rather than true physiological changes. These factors result in inconsistent sweat rates and fluctuating analyte concentrations, which can significantly impair real‐world device performance's accuracy, sensitivity, and reproducibility.^[^ ^74^, ^138^, ^139^, ^140^ ^]^

Sweat glucose levels often correlate poorly with blood glucose in non‐exercising or low‐sweat conditions, limiting sweat‐based sensors' reliability for monitoring diabetes. Similarly, fluctuations in electrolyte levels due to inconsistent sweating patterns can lead to inaccurate assessments of dehydration or electrolyte imbalances in both clinical and athletic settings. These performance fluctuations are critical from a disease monitoring perspective. In chronic conditions like cystic fibrosis, where elevated chloride levels in sweat serve as a diagnostic biomarker, inconsistent sampling or environmental factors can result in false positives or negatives, potentially compromising clinical decision‐making. Furthermore, variability in sweat hormone levels may obscure important trends in metabolic or endocrine disorders unless strict calibration and normalization protocols are employed. To address these challenges, advanced sweat‐sensing devices must include real‐time sweat rate monitoring, localized microfluidic sampling, and adaptive calibration AI algorithms that can adjust to changing conditions. Until these technologies are developed, widely adopted, and clinically validated, sweat‐based diagnostics may be more suitable for non‐critical, trend‐based health tracking, rather than for precise, disease‐specific monitoring.^[^ ^68^, ^141^, ^142^ ^]^

## 4 Sweat‐based Diagnostics and their Applications

Wearable sweat‐based sensors designed to detect analytes associated with human illnesses and ailments have recently been the focus of intense research. A few studies also focused on the development of wearable systems that incorporate multi‐analyte sensors and circuitry for in situ analysis and calibration with blood. The fundamental connection between sweat glucose and blood glucose levels dynamics is a critical factor (see Table 3) to consider when assessing the reliability of sweat‐based glucose monitoring. Blood glucose levels are tightly regulated and reflect systemic metabolic states, while sweat glucose concentrations are significantly lower, typically in the micromolar range, and are influenced by a complex interplay of local physiological and environmental factors. Unlike blood, where glucose levels can be directly measured and remain stable within physiological ranges, glucose in sweat is derived from a secondary transport process involving diffusion through epithelial layers. This can initiate time lags and result in concentration attenuation. In addition, factors such as sweat secretion rate, gland‐specific metabolism, variable skin permeability, and surface contamination can all influence the glucose concentration found in sweat. Furthermore, glucose can be locally utilized by the skin‐resident microbiome or enzymatically degraded, which alters its concentration independently of blood glucose levels. These conditions contribute to a non‐linear and often inconsistent relationship between sweat and blood glucose levels, making real‐time correlation unfeasible or challenging without individualized calibration or compensation algorithms. This section highlights several examples of the application of wearable technology in areas such as the monitoring of health and diseases, tracking of physical activity, assessments of drug metabolism, and detection of ethanol.

### 4.1 Sweat‐Based Wearable Bioelectronics

#### 4.1.1 Monitoring Health and Diagnosing Diseases

**Figure 10:**
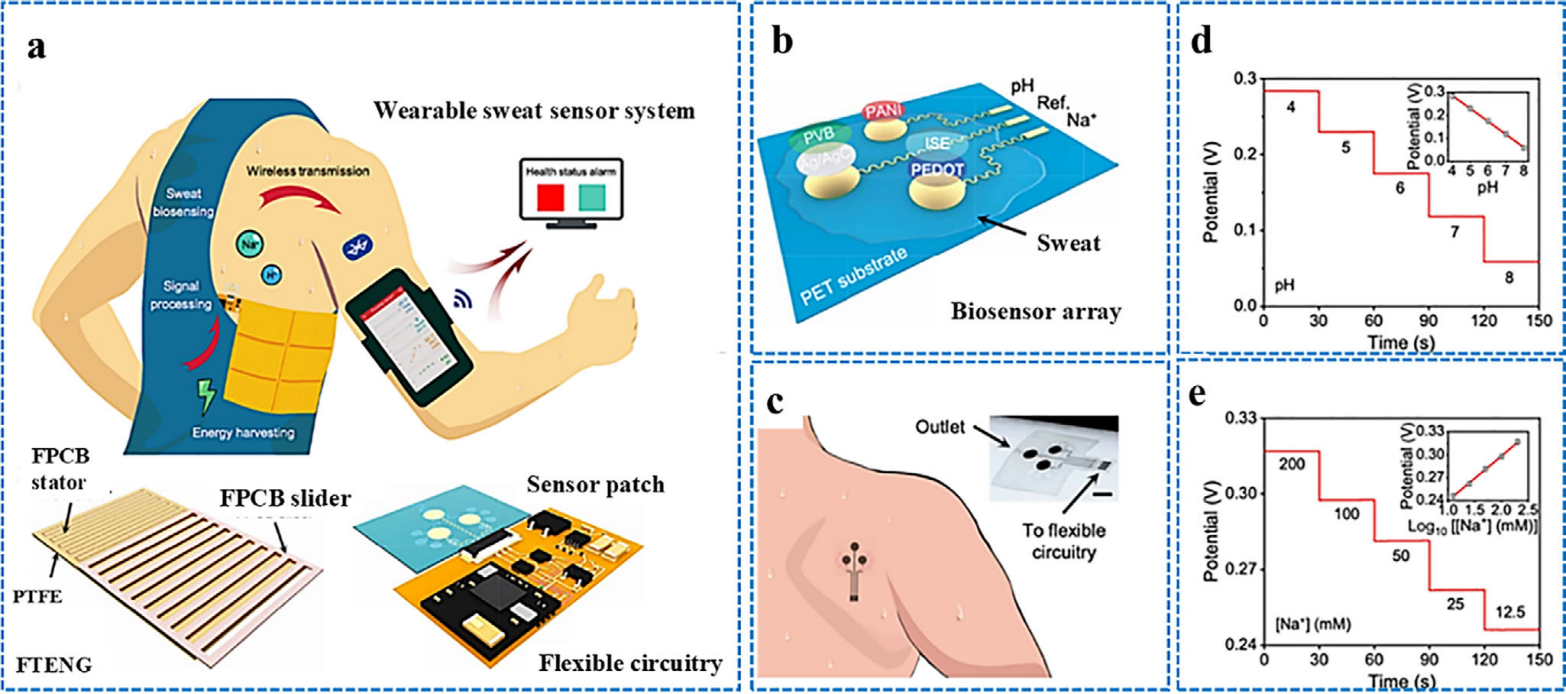
Sweat‐based sensors for continuous monitoring of health. a) Diagrammatic representation of the device, its functioning, and of the flexible TENG that powers it. b, c, Schematic representations of the sensor array b) and microfluidic sensor patch c). d, e, Open‐circuit potential responses of the pH sensor in standard Mcllvaine buffer d) and Na^+^‐ion sensor in sodium chloride solution e). Reproduced with permission.^[^ ^16^ ^]^ Copyright 2021 MDPI.

Sweat‐based biosensors present a valuable option for monitoring health and diagnosing diseases. For instance, diabetes is closely correlated with the levels of glucose metabolites, which can be readily identified in sweat.^[^ ^143^, ^144^ ^]^ A glucose monitoring system based on sweat was developed utilizing an electrochemical monolithic glucose sensor, which includes features for correcting pH and temperature.^[^ ^145^ ^]^ The bioelectronic device can monitor glucose levels in sweat in real time. Additionally, it incorporates a microneedle‐based drug delivery system to control blood glucose. While devices that allow continuous monitoring of blood glucose are currently available, the sweat‐based glucose monitors offer the potential to minimize the size of current devices and provide a pain‐free method for managing diabetes. **Figure** **10**a illustrates a wearable health monitoring device developed by a team of researchers at the California Institute of Technology, USA; the device operates on energy generated from human movement.^[^ ^146^ ^]^ The bioelectronic device is driven by a flexible triboelectric nanogenerator (TENG) and utilizes potentiometric sensing to measure Na^+^ levels and pH in sweat. It processes the signals and sends the data via Bluetooth to a mobile app for real‐time health monitoring.^[^ ^147^, ^148^, ^149^, ^150^, ^151^ ^]^ Figure 10b presents a schematic of the biosensor array, including the sensors for pH and Na^+^ ions, arranged on a malleable polyethylene terephthalate (PET) substrate.^[^ ^71^ ^]^ The entire design is wearable by adding it onto an FPCB that can be placed on the arm or side of the torso, as illustrated in Figure 10c. Self‐powering is enabled via the seamless inclusion of a flexible TENG. The TENG operates on the principle of contact electrification, generating a potential difference between the copper and polytetrafluoroethylene plates through relative sliding motion, allowing the device to capture biomechanical energy from human movement. The TENG in the device produces the highest power yield of 0.94 mW for a 4.7 MΩ load, making it ideal for wearable applications, as shown in Figures 10d and 10e. These aspects demonstrate the efficacy of these devices for monitoring pH and the levels of Na^+^ ions.^[^ ^152^, ^153^ ^]^

Libu et al. briefly addressed the fundamental disconnection between sweat and blood glucose dynamics, which is critical for assessing the translational potential of energy‐autonomous wearable systems. While sweat glucose ultimately derives from blood glucose, it is present only at micromolar concentrations. It is subject to attenuation, temporal lag, and variability influenced by sweat rate, glandular activity, skin permeability, and environmental factors. These discrepancies result in a non‐linear and often inconsistent relationship between sweat and blood glucose levels, complicating direct clinical interpretation. Recent advances, including the application of pharmacokinetic modeling and personalized algorithmic calibration, have demonstrated significantly improved correlations (Pearson's r ≈ 0.98 with RMSE ≈12 %), highlighting the importance of integrating computational approaches to bridge this physiological gap.^[^ ^154^, ^155^ ^]^

**Figure 11:**
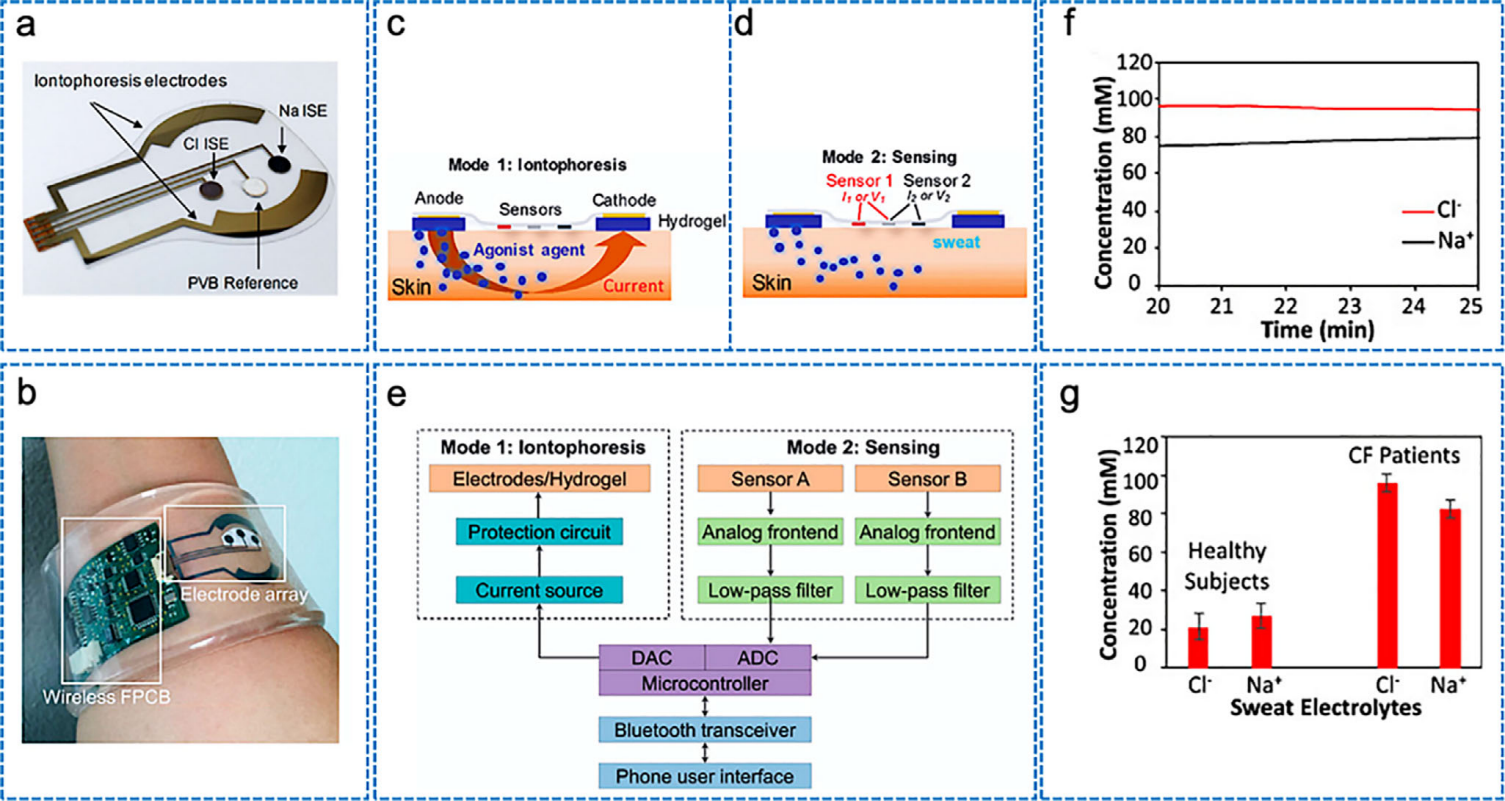
Overview of the monitoring device for cystic fibrosis. a) Electrodes are employed for iontophoresis and sensing. b) A flexible wearable device worn on the wrist of the user. c,d) Description of the working modes of the system, including modes 1 (iontophoresis; c) and 2 (sensing; d). e) Block diagram of the monitoring system. f) Real‐time on‐body measurements of Na+ and Cl− ions concentrations in the iontophoresis‐induced sweat of a patient with cystic fibrosis. g) Comparison of sweat electrolyte levels between six healthy subjects and three patients with cystic fibrosis. Reproduced with permission.^[^ ^16^ ^]^ Copyright 2021 MDPI.

Sweat‐based sensing technology provides an affordable means for the diagnosis and detection of various diseases. Various devices have been developed to diagnose cystic fibrosis,^[^ ^108^ ^]^ a genetic disorder that profoundly affects the lungs, digestive system, and other organs of humans. It impacts the functioning of cells that produce mucus, sweat, and digestive fluids, causing these secretions to thicken and become sticky. Instead of acting as lubricants, the thickened secretions block tubes and passageways, particularly in the lungs and pancreas, leading to significant harm. This can result in symptoms such as airway damage, chronic infections, and, in severe cases, respiratory failure. Reports from the Cystic Fibrosis Foundation Patient Registry show that over 70,000 individuals worldwide are afflicted with the disease, underscoring the need for continuous monitoring of this serious condition. The system design developed for monitoring cystic fibrosis is illustrated in **Figure** **11**a,b. The operation of the device involves the stimulation of sweat secretion via iontophoresis to generate different secretion profiles, as shown in Mode 1. The front‐end electronics analyze these in real time, as depicted in Mode 2 (Figure 11c,d). The processed signal is transmitted to the communication module of the circuit, which subsequently transmits the data (concentration of Na^+^ and Cl^−^ ions) to a phone. The data are displayed in an easy‐to‐understand format for the user. The signal processing workflow is shown in Figure 11e. This system allows the real‐time measurement of concentrations of Na^+^ and Cl^−^ ions in the iontophoresis‐induced sweat of patients with cystic fibrosis, as shown in Figure 11f. Figure 11g compares electrolyte levels in the sweat of six healthy individuals and three patients with cystic fibrosis.

#### 4.1.2 Monitoring of Physical Activity

**Figure 12:**
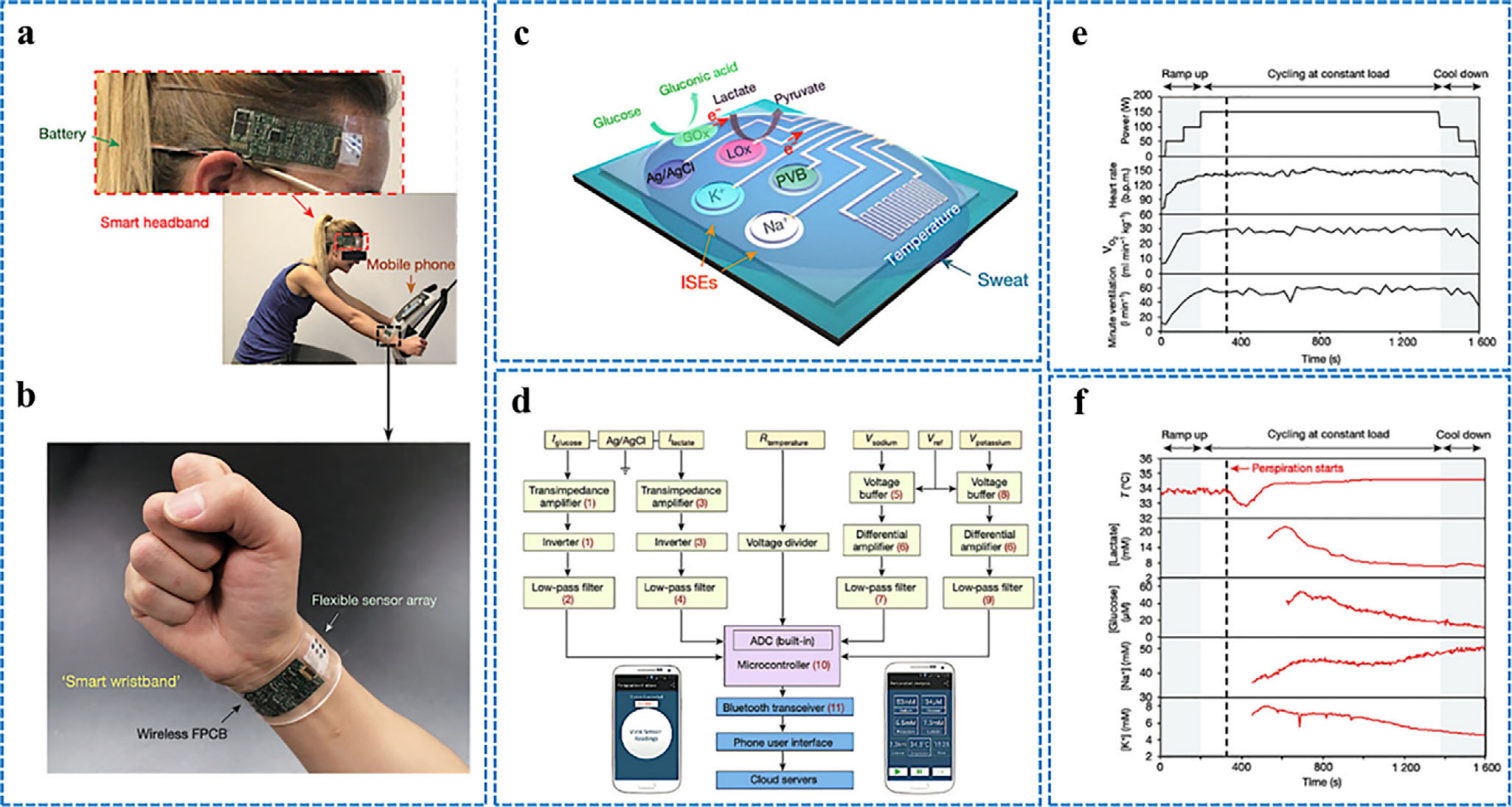
Sweat‐based wearable sensors for monitoring physical activity. a) A participant performing stationary exercise while wearing sensors on the forehead and wrist. b) A flexible integrated sensor array (FISA) is positioned on the wrist, connected to a wireless flexible printed circuit board (FPCB). c) A diagram illustrating the structure of the sensor array. d) System block diagram showing data processing and transmission flow. e) Real‐time sweat analysis from the forehead‐mounted FISA. f) Physiological responses during constant‐load exercise at 150 W, including power output, heart rate, oxygen uptake, and ventilation, recorded using external monitoring equipment. Reproduced with permission.^[^ ^16^ ^]^ Copyright 2021 MDPI.

Wearable sweat biosensors are primarily used for monitoring exercise. Gao et al. developed a wearable sensor array that simultaneously monitors multiple analytes. This sensor utilized a flexible integrated sensing array (FISA), and the signal was processed by an FPCB.^[^ ^144^ ^]^ Images of the FISA and FPCB sensors placed on the head and wrist of the subject are shown in **Figure** **12**a,b. These sensors are built to endure the strains of everyday use and physical activity; for instance, bending of the FPCB at radii of 1.5 and 3 cm exerted minimal impact on the response of FISA.

The design, mechanism, and functions of the sensor are described in Figure 12c. The amperometric sensors for glucose and lactate function using glucose oxidase and lactate oxidase, respectively, which are immobilized within a permeable membrane. Both sensors utilize the silver chloride (Ag/AgCl) electrode as the counter and common reference electrodes. These enzymatic sensors naturally produce current signals that are proportional to the concentrations of the respective metabolites, which then move between the working and Ag/AgCl electrodes. The levels of Na⁺ and potassium (K⁺) ions were determined utilizing ion‐selective electrodes. These potentiometric devices work alongside a reference electrode coated with polyvinyl butyral (PVB) to maintain a stable potential in solutions with varying ionic strengths. Additionally, the generation of Cr/Au microwires allows for a temperature sensor based on resistance.

As shown in Figure 12d, the potentiometric data obtained are transferred via an amplifier, inverter, analog‐to‐digital converter, and Bluetooth module for final display on a smartphone. The sensor was evaluated on a subject performing a stationary cycling exercise (Figure 12a), which included 3 min of cycling at an enhanced rate, steady cycling at 150 W for 20 min, and 3 min of cooling down; the heart rate, oxygen consumption, and pulmonary ventilation were evaluated during the exercise. The measured data indicated an increase in the values of the evaluated parameters concomitantly with an increase in output power. The real‐time FISA‐based measurements of sweat on a subject's forehead are shown in Figure 12e; it demonstrates that continuous sweating caused an increase in skin temperature at ≈400 s, followed by maintenance of that temperature. This was accompanied by a progressive decrease in glucose and lactic acid concentrations in the sweat (Figure 12f), which may be attributed to the diluting effect caused by an increase in the sweat rate.

#### 4.1.3 Monitoring Drug Metabolism

**Figure 13:**
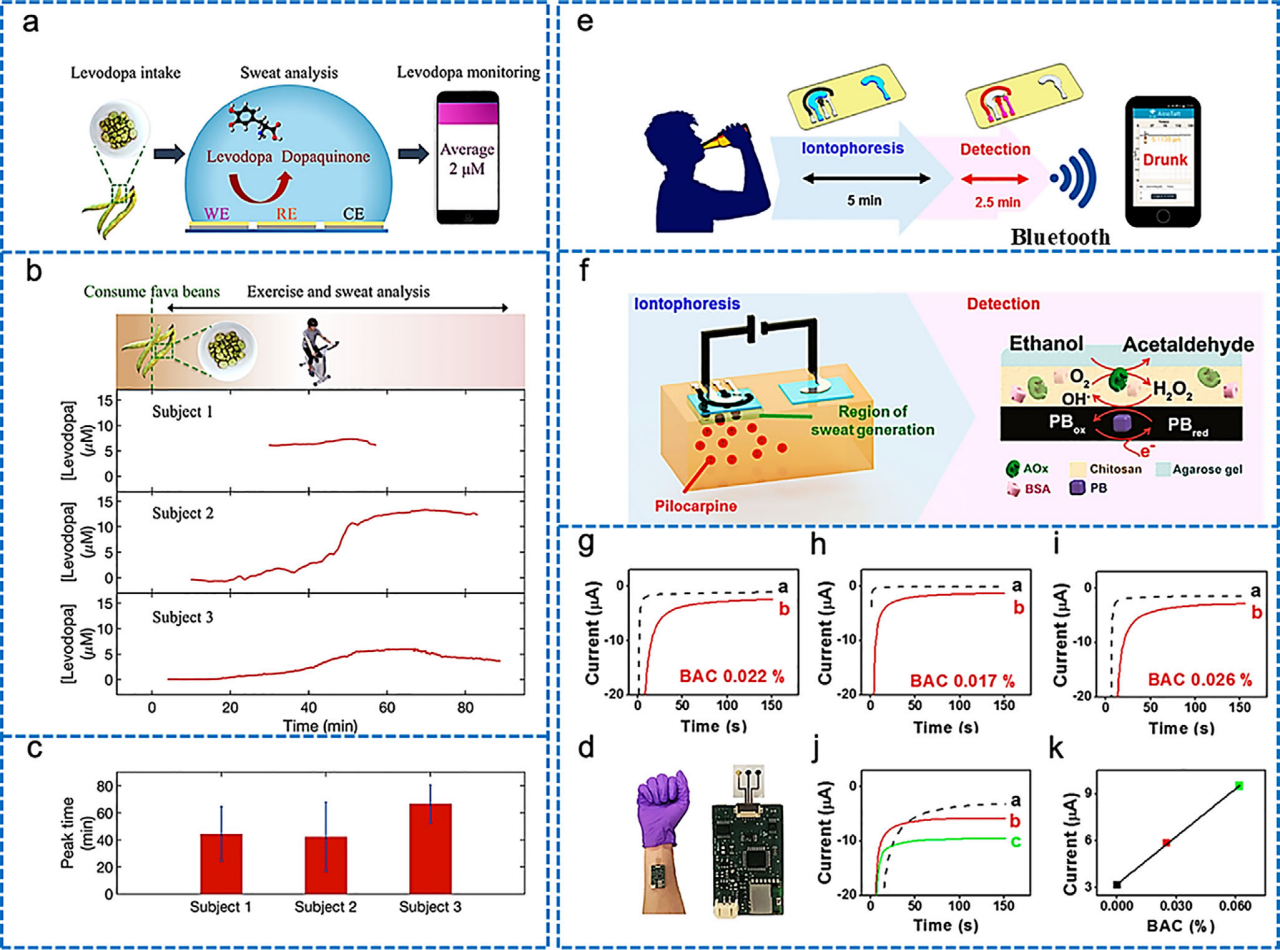
Mechanisms of monitoring drug metabolism and ethanol levels. a) Sensor‐based analysis of sweat for monitoring the levels of levodopa. b) Analysis of sweat following cycling. Representative levodopa concentrations in sweat from three different subjects following the consumption of fava beans (450 g). c) Average duration for attaining peak levodopa concentrations in the sweat of three subjects across multiple exercise trials. d) Optical image of the S‐band worn on a subject's wrist. e) Sweat‐based ethanol sensor. Alerts are sent to a smart device. f) Schematic representations of components involved in the iontophoretic system (left) and processes involved in the amperometric detection of ethanol in the reagent layer of the working electrode (right). g‐–i) Response currents (in µA) generated in three human subjects before (plot “a”) and after (plot “b”) consumption of 12 oz of beer. BAC, blood‐alcohol content. j) Chronoamperograms obtained with BAC values of 0% (a), 0.025% (b), and 0.062% (c). k) Correlation between response current and BAC values. Reproduced with permission.^[^ ^16^ ^]^ Copyright 2021 MDPI.

Wearable sensors for sweat‐based biomarkers can be used to monitor exercise intensity and drug metabolism. For instance, levodopa is commonly used for the treatment of patients with Parkinson's disease; the response of patients to this medication is influenced by numerous factors, underscoring the necessity for monitoring the concentration of the drug in blood. As the monitoring methodologies based on blood are typically invasive, the sweat‐based sensing of levodopa using a sweatband (S‐band)–integrated sensor was explored.^[^ ^156^ ^]^ The sensor employs a standard three‐electrode setup consisting of working, reference, and counter electrodes on a PET substrate (**Figure** **13**a). The test subjects included healthy individuals who had just consumed fava beans, a natural source of levodopa. This method allows for comprehensive testing of the S‐band on non‐vulnerable subjects using iontophoresis‐induced sweat. The concentration of levodopa in iontophoresis‐induced sweat was continuously monitored following the consumption of fava beans; the S‐band was employed to continuously monitor levodopa content in sweat, which closely matches the levels found in blood. The duration of iontophoresis‐induced sweating is typically short‐lived, while that caused by exercise tends to last longer. This difference can be exploited for the evaluation and validation of these sensors. Figure 13b shows the responses recorded from three subjects exercising on a stationary ergometer. Each trial involved the consumption of 450 grams of fava beans by the subjects and their participation in multiple exercise sessions. The cumulative results, illustrated in Figure 13c, show the average time at which the peak concentration of levodopa can be detected. A comparative investigation of sweat generated because of physical activity as well as iontophoresis allows the monitoring of drug metabolism, thereby optimizing drug dosages. Figure 13d shows the sensor equipment installed on a subject's wrist. Future research efforts are expected to focus on pharmacodynamics involving a comparison of various drugs, extending the duration of iontophoresis‐induced sweating, and extending electrode lifetimes. Consequently, the S‐band may be integrated into drug delivery systems, utilized to investigate the intrinsically complicated pharmacological profiles, and optimize treatment doses for individuals with Parkinson's disease.

#### 4.1.4 Measurement of Ethanol Levels

Ethanol levels in sweat can be measured using a tattoo‐like sensor designed for sweat monitoring and used for estimating blood‐alcohol concentrations.^[^ ^116^ ^]^ A diagrammatic representation of a system that uses an ethanol‐in‐sweat sensor to send an alert to a smart device is presented in Figure 13e. The patch resembles a temporary tattoo and contains an enzyme‐based amperometric sensor that detects ethanol in sweat, combined with an iontophoresis‐based delivery mechanism for the drug pilocarpine. This system communicates with smart devices via Bluetooth. A careful adjustment of the iontophoresis current (0.6 mA) balances effective drug delivery with user comfort, given that high current can easily cause skin irritation. This device offers greater accuracy than typical breath analyzers as it eliminates potential errors from environmental factors (such as humidity) or products (such as mouthwash). Additionally, it provides a more rapid means of measuring blood‐alcohol levels compared to other skin‐based devices, requiring ≈10 min instead of the 0.5 to 2 h required for the conventional detection methods. Ethanol sensors have therefore been proposed as a useful tool for detecting illegal alcohol consumption among drivers. To detect ethanol in sweat, this sensor device employs iontophoresis‐based delivery of the medication pilocarpine via the skin using a steady current. The sensor makes use of a printed Prussian blue electrode transducer and an electrode for the enzyme alcohol oxidase. The paper electrodes of the wearable temporary tattoos are readily removable from the skin and mass‐produced using screen printing. These procedures involving iontophoresis and the subsequent amperometric identification are detailed in Figure 13f. The response of the sensor device was validated by conducting experiments on three human subjects; the responses following the consumption of equal amounts of alcohol by the subjects have been illustrated using plots (Figure 13g–i), with curves “a” and “b” representing the amperometric responses before and following alcohol consumption, respectively. The variation in blood‐alcohol concentrations among the subjects is due to differences in their rates of metabolism; however, the change in the response current from the sensor device following alcohol consumption is clearly noticeable. Additionally, three control experiments were carried out to ensure the absence of false positives. Figure 13g demonstrates the lack of any changes in the sensor's response at a BAC value of zero. Additionally, it shows the sensor's reaction over a specific duration in the absence of alcohol consumption, confirming that the generation of the response current is due to alcohol intake. Figure 13h highlights the lack of response when the sensor device does not contain immobilized enzyme, proving the high specificity of the device for ethanol in sweat. Figure 13i shows the sensor's response with and without iontophoresis; it demonstrates that the method of sweat extraction does not affect the sensor's performance. These observations suggest that the sensor is highly selective for ethanol in sweat, such that it fails to exhibit any response at a BAC of 0%. Figure 13j shows the response of the sensor at various BAC levels (0%, 0.025%, and 0.062%; panels a, b, and c, respectively). Figure 13k illustrates the changes in response current at various levels of BAC. Indeed, a balanced evaluation should consider cost, accuracy, and certification when comparing sweat‐based ethanol sensors to conventional breath analyzers. Breath analyzers are mass‐produced using standardized processes, making them relatively inexpensive, typically ranging from $30 to $300. They have established regulatory approvals (FDA and DOT) that ensure their accuracy, reliability, and broad acceptance in clinical and forensic settings. In contrast, while offering the potential for continuous and non‐invasive monitoring, sweat‐based ethanol sensors are still in the early stages of research. They often rely on advanced materials and designs, which can drive up manufacturing costs. Additionally, these sensors face challenges such as calibration drifts and biofouling, which can affect their accuracy. The lack of standardized clinical validation and regulatory certification limits their applicability.^[^ ^157^, ^158^ ^]^

#### 4.1.5 Monitoring of Biomolecules

**Figure 14:**
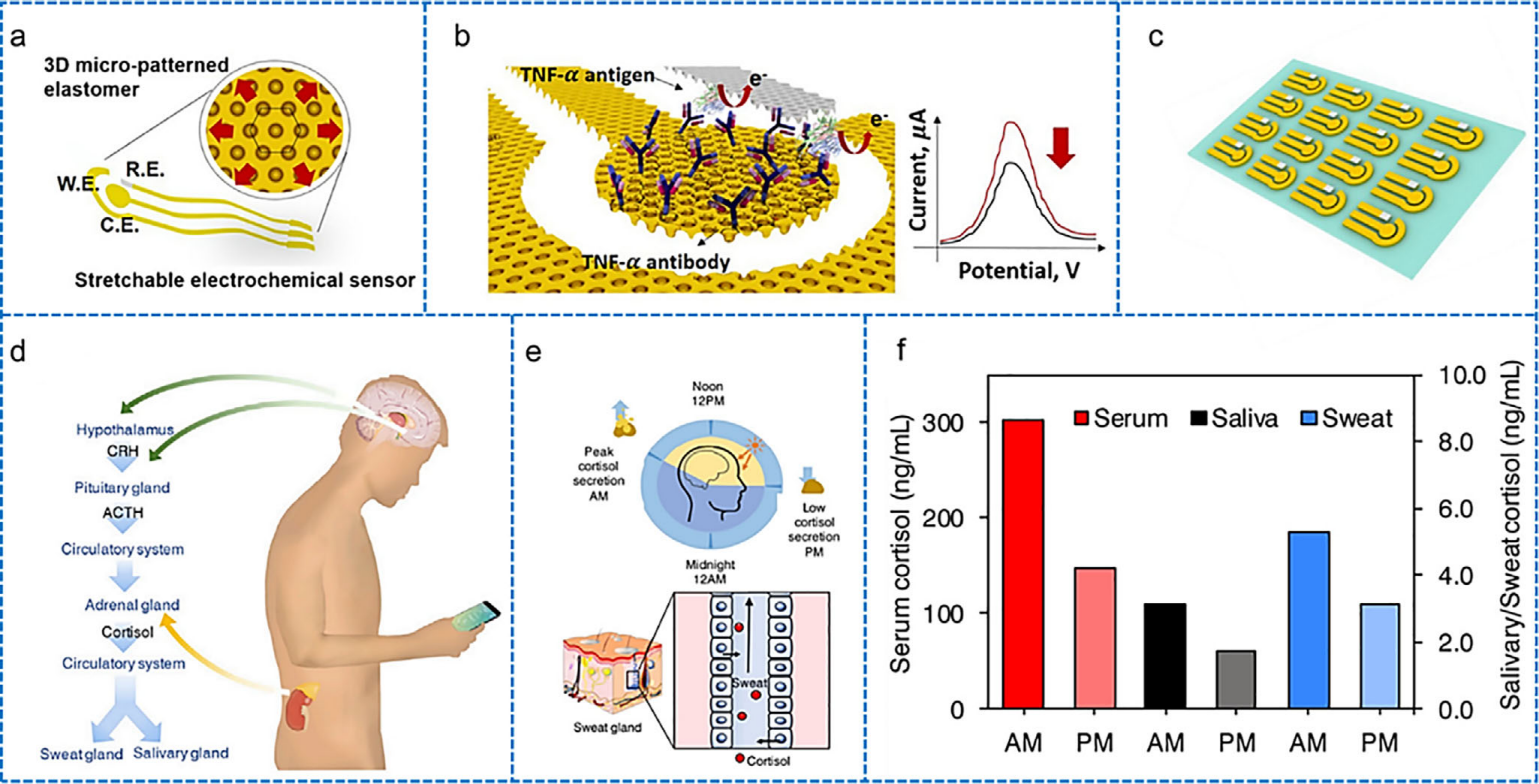
Sweat‐based sensors for monitoring biomolecules. a) Schematic representation of a stretchable chemical immunosensor for the cytokine TNF‐α. b) Schematic illustration of the TNF‐α antibody immobilized on the working electrode for electrochemical sensing. c) Image of fabricated device arrays. d) Schematic illustration of cortisol secretion in sweat and saliva. CRH, corticotropin‐releasing hormone; ACTH, adrenocorticotropic hormone. e) Schematic representation of the light–dark cycle that regulates circadian rhythm and controls cortisol secretion in sweat. f) Cortisol levels in a healthy subject's serum, saliva, and sweat are different at various times of the day. Reproduced with permission.^[^ ^16^ ^]^ Copyright 2021 MDPI.

In addition to the three previously discussed biomarkers, biomolecules such as proteins, cytokines, nucleic acids, and neuropeptides are crucial indicators of individuals' health or infection status. Although low concentrations of these molecules are consistently found in body fluids, they hold significant potential for monitoring chronic wound healing and diagnosing or managing conditions such as Parkinson's disease, depression, and wound healing. For instance, tumor necrosis factor alpha (TNF‐α) was detected using a stretchable electrochemical immunosensor that tracked the healing of wounds, as shown in **Figure** **14**a.^[^ ^159^ ^]^ The experiment involved immobilizing TNF‐α‐specific antibodies on the working electrode to detect TNF‐α using a technique called Differential Pulse Voltammetry (DPV). In the absence of TNF‐α, the faradaic current was observed at the redox potential of ferricyanide, as shown in Figure 14b. However, the presence of TNF‐α resulted in the formation of a barrier layer on the electrode surface, which hindered electron transfer and reduced current generation. The immunosensor performed well in buffer containing TNF‐α concentrations that fell within the typical clinical range (0.1 pM to 0.1 M) and human serum. Additionally, the sensor was prepared using 3D micropatterned elastomers; it is therefore adaptable to up to 30% strain, making it suitable for wearable immunosensing applications (Figure 14c). The prompt and accurate identification of stress is crucial for tracking and managing mental health. The highly subjective nature of current methods, such as questionnaires, prompted the proposal to employ a wearable chemical sensor. This sensor is part of a selective and compact mHealth device that utilizes a flexible laser‐induced graphene sensor for monitoring the levels of the stress hormone cortisol in a noninvasive manner, as illustrated in Figure 14d. Cortisol levels in sweat have been reported to respond rapidly to acute stress and are closely associated with the levels in blood. Additionally, there is evidence of a daily cycle and specific response curve to stress, which suggests that the mHealth sensor system can be employed for dynamic stress monitoring (see Figure 14e), Observed variations in cortisol levels in response to the circadian rhythm, with ratios of levels found in AM and PM ranging from 1.35 to 2.00 (see Figure 14f).

### 4.2 Paper‐Based Microfluidic Sweat Sensors for POCT

The integration of paper‐based microfluidic devices (µPADs) into wearable biosensors provides considerable advantages, including cost‐effectiveness, a high surface area–to‐volume ratio, and the use of nontoxic, flexible materials. µPADs enable sampling as well as storage of reagents on the same platform.^[^ ^160^ ^]^ The capillary‐driven flow in µPADs allows fluid movement across the device without any requirement for external power sources, making them ideal for low‐energy applications. Furthermore, precise control is achieved in the regions of biospecific recognition via chemical printing or cutting techniques to manufacture the µPADs. The 2D or 3D design of the microfluidic pattern can be determined based on the choice of vertical or horizontal paths for the movement of fluids. The wide range of materials, processing techniques, and combinations allows the creation of various custom‐designed devices for specific applications.^[^ ^161^, ^162^, ^163^ ^]^ As documented in literature, one of the main uses of wearable paper‐based devices for sweat analysis includes the monitoring of glucose levels.^[^ ^164^ ^]^ Most current systems focus on multi‐parametric analysis, allowing the measurement of multiple analytes in a single test. A common example includes the simultaneous measurement of the levels of glucose and lactate as well as pH. These systems directly integrate all the necessary reagents, including specific enzymes and substrates, on the paper platform. The recognition of the target analytes by the enzymes generates a signal (colorimetric or electrochemical), which can be measured to provide quantitative results.

Cao et al. reported a wearable electrochemical biosensor using various stacked paper layers produced using wax‐screen printing. The sequential folding of the layers in the device allows sweat analysis. One of the main challenges in sweat analysis using wearable biosensors involves ensuring the accuracy of sample‐volume measurements. A simulation of capillary flow within the device and testing using various sample volumes revealed that it takes ≈5 s for the fluid to flow through the system.^[^ ^165^ ^]^

Fiore et al. developed a wearable biosensor that employed immunoassay with electrochemical detection for measuring cortisol levels. They optimized a competitive immunoassay platform based on magnetic beads. Data from the sensor was wirelessly transmitted to a smartphone using near‐field communication (NFC) technology.^[^ ^166^ ^]^ De Brito et al. described the development of a biosensor that utilized the oxidation of ferrocyanide, a byproduct of bacterial respiration, to detect the bacterium Staphylococcus aureus instead of relying on conventional protein‐based recognition methods. The oxidation of ferrocyanide was detected using electrochemical methods, presenting a novel approach for the detection of bacteria.

Vaquer et al. reported a time‐sampling method that employed sequential measurements for effective monitoring.^[^ ^167^ ^]^ They designed valves composed of dried polymers, which dissolve upon contact with liquid, to control the volume of sweat. The valves allow the flow of the sweat sample to the functionalized region containing immobilized urease, an enzyme specific to urea. The reaction between urease and urea facilitates detection, and measurements can be performed sequentially using timed valve openings, allowing for a colorimetry‐based detection of changes in analyte concentration.

### 4.3 Wearable Biosensors Based on Highly Flexible Plastic Substrates

Ubiquitous polymers that exhibit flexibility and elasticity, including PET, polyimide (PI), polydimethylsiloxane (PDMS), polyurethane (PU), and polymethyl methacrylate (PMMA), are commonly employed in wearable biosensors. The low cost, biocompatibility, and physicomechanical properties of these materials and their ability to provide excellent electrical insulation render them ideal substrates for biosensing devices.^[^ ^26^, ^168^ ^]^

Gao et al. reported the development of a wearable biosensor capable of simultaneously detecting multiple biomarkers in sweat. The sensor included a PET substrate and FPCB technology for signal processing and the integration of circuit components. The glucose and lactate levels were measured using the enzymes glucose oxidase and lactate oxidase, which were immobilized on a polysaccharide chitosan film. The levels of Na^+^ and potassium (K^+^) ions were measured using an ion‐selective electrode paired with a PVB‐coated reference electrode.^[^ ^144^ ^]^

Park et al. evaluated PI substrates integrated into a needle for use in medical procedures; the device contained an electrodeposited reactive layer for detecting the levels of glucose and lactose. The design involved six microelectrodes placed on a polyimide (PI) membrane, with each electrode containing immobilized enzymes to detect target analytes. Additionally, metal oxide was deposited onto the electrodes to measure pH, creating a versatile sensing system for real‐time monitoring during medical applications.^[^ ^169^ ^]^

Xu et al. developed a wearable electrochemical biosensor with electrodes printed on PDMS and connected using conductive silver wires. The system utilizes NFC technology to transmit data, including the levels of glucose and the ions Na^+^ and K^+^, as well as pH, directly to smartphones. This design eliminates the need for batteries or external wiring. Sweat sampling is achieved with a porous sponge placed over the electrodes, creating a liquid environment that allows the electrodes to function effectively for accurate detection.^[^ ^170^ ^]^

### 4.4 Applications for Wearable Monitoring Systems

#### 4.4.1 Monitoring Fitness

The implementation of biometric tracking in sports and for monitoring fitness represents one of the earliest applications of wearable monitoring systems. These devices continuously obtain real‐time physiological data in a noninvasive manner, which is essential for the accurate design of treatment plans and personalized training programs; this approach helps improve athletic performance while reducing the risk of injury.^[^ ^171^, ^172^ ^]^ The commonly evaluated physiological biomarkers in fitness tracking include heart rate (HR) and core temperature, which are indicators of physiological adaptation, exercise intensity, and physical exertion. Additionally, muscle oxygen saturation is measured to optimize athletic performance.^[^ ^173^, ^174^, ^175^, ^176^ ^]^

**Figure 15:**
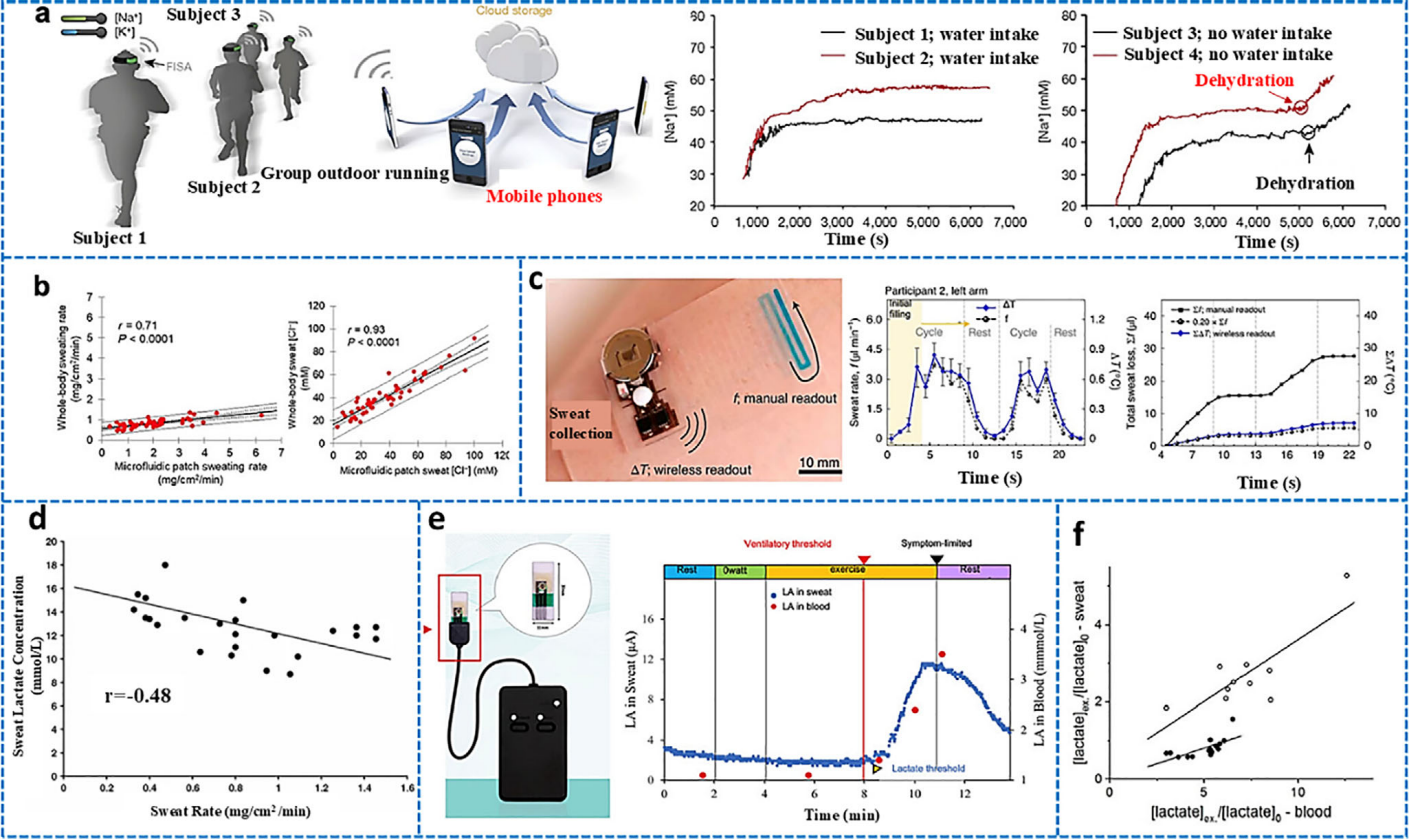
Wearable sensors for monitoring fitness and human performance. a) FISA enables multiplexed sweat analysis to identify the onset of dehydration during exercise. b) WBSR versus RSR measured with microfluidic patches (left panel), and chloride concentrations in whole‐body sweat versus regional sweat measured using microfluidic patches (right panel). c) Thermistor‐based flow sensor for wireless monitoring of sweat rates. Validation was done via visual quantification of sweat flow. d) Inverse association between sweat lactate concentration and sweat rate. e) Wearable lactate sensor that tracks sweat lactate threshold (LT1) in conjunction with ventilatory threshold (VT1). f) Correlations between lactate concentrations in sweat from the thigh (region corresponding to working muscle, open circles) or the arm (region corresponding to latent muscle, closed circles) and blood. Reproduced with permission.^[^ ^74^ ^]^ Copyright 2023 ACS.

An important application of the on‐field monitoring of biochemical profile involves the assessment of hydration status. An inadequate intake of water can result in dehydration, while excessive intake can induce hyponatremia.^[^ ^177^ ^]^ The ions Na^+^ and K^+^ are important electrolytes in sweat, and their levels can be easily measured to assess hydration status and predict muscle activity. In particular, the levels of Na^+^ ions in sweat closely correlate with WBSR and total body sodium loss. A measurement of Na^+^ concentration in regional sweat (using devices such as patches on the forearm) allows the estimation of whole‐body Na^+^ levels using appropriate models. This, in turn, helps to determine the total body sodium loss through sweat.^[^ ^178^ ^]^ In 2016, a FISA was developed to monitor sweat metabolites such as glucose and lactate, electrolytes such as Na^+^ and K^+^ ions, and skin temperature. This system was evaluated during various physical activities such as cycling and running, and exhibited the ability to monitor important physiological parameters in real‐time, as shown in **Figure** **15**a.^[^ ^144^ ^]^ The real‐time monitoring of sweat Na^+^ and K^+^ levels in study subjects (runners) engaged in prolonged outdoor activity is shown in Figure 15b; the results revealed that participants who consumed water maintained stable Na^+^ and K^+^ levels after initial fluctuations. By contrast, the subjects who did not consume water experienced significant and slight increases in Na+ and K+ ions levels, respectively, after 80 min of activity. These findings emphasize the importance of monitoring Na^+^ levels in sweat to assess dehydration during extended physical exertion.

An alternate method for evaluating salt imbalance and the extent of dehydration in athletes involves measuring the content of Cl^−^ ions in sweat.^[^ ^171^ ^]^ A comprehensive study with 312 athletes as study subjects employed a wearable microfluidic device with a smartphone image processing platform for investigating the association between sweat Cl^−^ levels and RSR during competitive sports.^[^ ^179^, ^180^ ^]^ A comparison of sweat rates measured using a microfluidic patch to whole‐body sweat rates (calculated from changes in body mass before and after exercise following adjustments for fluid intake and loss) found a strong correlation between the two, regardless of body surface area normalization. The study also revealed a strong association between the content of Cl^−^ and Na^+^ ions in whole‐body sweat (r^2^ = 0.98), reflecting the close association between these two electrolytes during sweating. A simple linear regression model was employed for estimating Cl^−^ levels in whole‐body sweat based on the values in regional sweat. The results indicated a strong correlation between the determined and estimated Cl^−^ concentrations in whole‐body sweat (r^2^ = 086), as shown in Figure 15b. Therefore, Cl^−^ concentrations in regional sweat may be a useful indicator for estimating sweat Na^+^ loss and evaluating the need for electrolyte replenishment. Additionally, a strategy involving the use of a thermal actuator and precision thermistor‐based flow sensor that wirelessly communicates data via Bluetooth to allow continuous assessments of sweat loss was also proposed, as shown in Figure 15c.^[^ ^181^ ^]^ The wireless measurements of sweat rate (ΔT) closely matched manual readings of flow rate (f) in a dye‐filled serpentine microfluidic channel during cycling and resting conditions. The wireless flow rate was found to increase during cycling to reach a steady state; however, it dropped to nearly zero under resting conditions. This observation demonstrates the strong correlation between physical activity and sweat rate, confirming the reliability of wireless monitoring for tracking changes in sweat production during the exercise and recovery phases.

Sweat rate has been shown to affect lactate levels due to the dilution of lactate in sweat at higher sweat rates. A study explored the associations between exercise intensity (60%, 70%, and 80% of the age‐predicted maximum HR) and sweat lactate concentration as well as lactate excretion rate (LER), which is calculated by multiplying sweat lactate concentration with the sweat rate; a 90‐min treadmill walking session was employed for assessing the impact of exercise intensity on lactate levels in sweat.^[^ ^182^ ^]^ The study revealed decreased sweat lactate concentration and increased LER with increased exercise intensity. An inverse association was observed between sweat lactate concentration and sweat rate (r = −0.48), indicating that lactate concentration decreases with an increase in the sweat rate. By contrast, a strong positive correlation was observed between LER and sweat rate (r = 0.94), indicating that increased sweat production leads to the excretion of greater amounts of lactate via sweat. These observations suggest that increased exercise intensities indeed enhance the secretion of lactate in sweat, although this effect may be masked with uncorrected values for sweat lactate concentration due to the dilution effect brought on by increased sweat rates at higher exercise intensities (Figure 15d); the observed increase in lactate levels, therefore, may not accurately represent the true physiological response due to this dilution effect.

In previous studies, sweat lactate concentrations were adjusted by accounting for the changes in sweat rate to reveal a clear positive association between sweat lactate levels and exercise intensity.^[^ ^183^ ^]^ The correlation between sweat lactate threshold (LT_1_, the point at which lactate levels first rise with increasing exercise intensity) and ventilatory threshold (VT_1_) has been previously explored.^[^ ^184^ ^]^ This association was explored using a wearable lactate sensor, demonstrating how monitoring lactate levels can evaluate sensor performance during incremental exercise (Figure 15e). The LT_1_ in sweat closely aligns with the LT_1_ in blood as well as VT_1_. There is a sharp, continuous increase in sweat lactate concentration up to the point of exhaustion, followed by a decrease during recovery. The region where sweat is collected also affects lactate levels; the regions near active muscles exhibited concurrent increases in sweat and blood lactate levels, while areas near inactive muscles did not exhibit this pattern (Figure 15f).^[^ ^185^ ^]^

#### 4.4.2 Diagnosis of Cystic Fibrosis through Sweat Analysis

**Figure 16:**
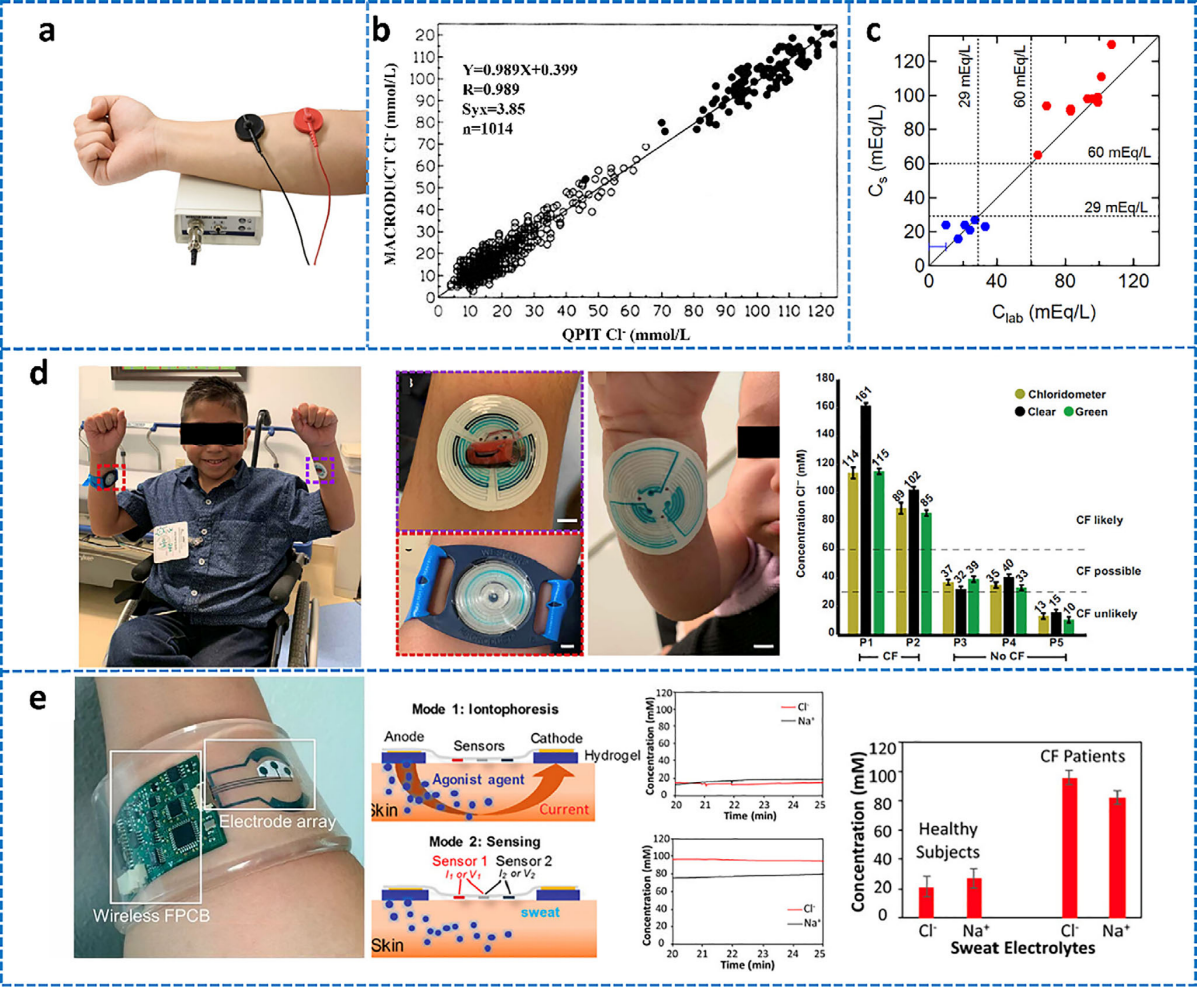
Wearable sensors for the diagnosis of cystic fibrosis. a) A commercial sweat sensor device for the diagnosis of cystic fibrosis. b) Correlation between the concentrations of Cl^−^ ions obtained in sweat from patients with cystic fibrosis (closed circles) and non‐afflicted individuals (open circles) using the Macroduct and QPIT systems. c) Correlation between sweat Cl^−^ levels in patients with cystic fibrosis (red circles) and non‐afflicted individuals (blue circles) obtained with a wearable sensor and laboratory‐based measurements. d) A soft epidermal microfluidic device for in situ colorimetry‐based quantification of Cl^−^ ions in sweat. Scale bars, 5 mm. e) An integrated single battery–powered wearable device for localized stimulation of sweat secretion and in situ analysis of sweat Cl^−^ levels. Reproduced with permission.^[^ ^74^ ^]^ Copyright 2023 ACS.

In addition to sports physiology, sweat rates and electrolyte levels have been regularly monitored for the diagnosis of cystic fibrosis, a life‐threatening genetic disorder caused by malfunctions in the cystic fibrosis transmembrane conductance regulator (CFTR) membrane channel, which affects sweat glands and leads to abnormal salt levels in sweat.^[^ ^186^ ^]^ Any interference in electrolyte movement within the reabsorptive channel of the sweat gland can cause significant salt loss via sweat. Since the discovery of the impermeability of sweat glands of patients with cystic fibrosis to Cl^−^ ions, the measurement of these (Cl^−^) ions in iontophoresis‐induced sweat has become the gold standard for the diagnosis of the disease, particularly for newborns.^[^ ^187^ ^]^ The Wesco Macroduct Sweat Testing System, as shown in **Figure** **16**a, is widely utilized in many diagnostic centers to detect cystic fibrosis.^[^ ^188^ ^]^ Unlike the conventional, time‐consuming quantitative pilocarpine iontophoresis test (QPIT), the application of the Macroduct system eliminates the need for weighing sweat and minimizing its evaporation, thereby simplifying the process and increasing its efficiency for the diagnosis of cystic fibrosis. The concentrations of Cl^−^ ions measured using the Wesco Macroduct system are comparable to those obtained with the conventional QPIT system, and clinically significant differences have not been observed. The Wesco Macroduct system exhibits an excellent ability to employ sweat chloride levels to reliably distinguish patients with cystic fibrosis from nonafflicted individuals (Figure 16b).^[^ ^188^ ^]^

Real‐time measurements of sweat Cl^−^ levels obtained using a wearable sensor with an integrated salt bridge were compared with results obtained using standard laboratory methods.^[^ ^189^ ^]^ Constant readings for sweat Cl^−^ levels (with SD of <1 mEq/L over a 5 min duration) were achieved within 15 min of sweat induction using the Macroduct system. The sweat volumes with real‐time detection (13.1 ± 11.4 µL) were often below the minimum threshold required for laboratory‐based testing. However, the results obtained using the wearable sensor demonstrated excellent correlation with lab results (r = 0.97), indicating the sensor's high accuracy for real‐time measurements of Cl^−^ levels (Figure 16c). To overcome challenges associated with sweat collection from infants with sensitive skin, the design was enhanced to improve the efficacy of sweat collection. A gentle, skin‐friendly microfluidic device was developed to directly collect sweat from the skin and conduct in situ colorimetry‐based Cl^−^ ions quantification, as shown in Figure 16d.^[^ ^190^ ^]^ The device effectively minimizes sweat loss and exhibits similar precision to containing technologies upon testing on patients with cystic fibrosis and non‐afflicted individuals across various age groups. Additionally, it replaces the complex process of sweat activation and extraction associated with the Macroduct system with a single, battery–powered wearable device that offers localized stimulation of sweat secretion and in situ analysis of Cl^−^ levels.^[^ ^108^ ^]^ The device features an array of electrodes for sweat induction as well as sensing, in conjunction with an FPCB, which allows for wireless data transmission, as shown in Figure 16e.

#### 4.4.3 Monitoring of Diet and Nutrition

Monitoring healthy diets is critical for managing various health conditions, including metabolic syndrome, diabetes, and cardiovascular diseases.^[^ ^191^, ^192^ ^]^ The management of chronic diseases involves multiple aspects; however, monitoring nutrition and making appropriate dietary interventions remain essential aspects, given that diet is a modifiable risk factor for various chronic conditions.^[^ ^193^ ^]^ The digitization of food frequency questionnaires and food diaries has enhanced their ease of use in online surveys and applications for tracking daily food consumption; however, the issues with these techniques include biases in portion estimation and misreporting.^[^ ^194^, ^195^ ^]^ Wearable sweat sensors have the ability to monitor nutritional data in a noninvasive, continuous manner; this facilitates the tracking of appropriate diets and provides guidance, which is expected to prove particularly useful for managing chronic conditions such as diabetes and cardiovascular diseases.

Wearable sweat sensors have been employed to detect various types of nutrients, including metabolites, vitamins, minerals, and amino acids. Among these, sweat glucose has been one of the most thoroughly studied nutrients, largely due to its crucial role in diabetes management. The increased awareness and employment of monitoring techniques for blood glucose have greatly advanced diabetes management. The current methods for measuring blood glucose levels involve taking small blood samples from finger pricks at specific intervals using a portable glucose meter. Monitoring glucose levels using sweat is a noninvasive method for continuously detecting and tracking serum glucose levels. The observed association between glucose levels in sweat and blood under in vitro conditions and the need for a noninvasive method that allows continuous glucose monitoring has driven recent advancements in wearable sweat glucose sensors. This emerging technology holds promise for real‐time glucose tracking more conveniently, especially for individuals managing diabetes.^[^ ^69^ ^]^ Earlier studies on wearable devices for sweat glucose monitoring focused on tracking changes in sweat glucose levels and their association with blood glucose levels.^[^ ^194^ ^]^

#### 4.4.4 Monitoring Stress and Mental Health

Stress has been considered a modern epidemic that impacts over a third of the population worldwide. During the coronavirus disease 2019 (COVID‐19) pandemic, the prevalence of anxiety and depression increased by 25%.^[^ ^196^ ^]^ At an individual level, ongoing mental health disorders increase the risk of developing cardiovascular and other diseases.^[^ ^197^ ^]^ On a larger scale, these issues impact social stability and place significant medical burdens on society.^[^ ^198^ ^]^ Real‐time evaluation of stress and ongoing monitoring in a preventive capacity enables early diagnosis and prompt initiation of treatment. Devices such as fitness trackers and smartwatches equipped with photoplethysmogram or electrocardiogram capabilities are poised to revolutionize healthcare, marking the shift toward more consumer‐focused models.^[^ ^199^ ^]^ Consumer healthcare devices presently detect stress in real‐time by measuring physiological indicators such as HR; however, limitations in the sensitivity and specificity of these devices pose challenges for the accurate quantification of stress levels. The emergence of wearable sensors capable of detecting biochemical markers in sweat in a noninvasive manner has presented a more precise approach for constantly monitoring and quantifying stress in real time.^[^ ^200^ ^]^

#### 4.4.5 Therapeutic Drug Monitoring

**Figure 17:**
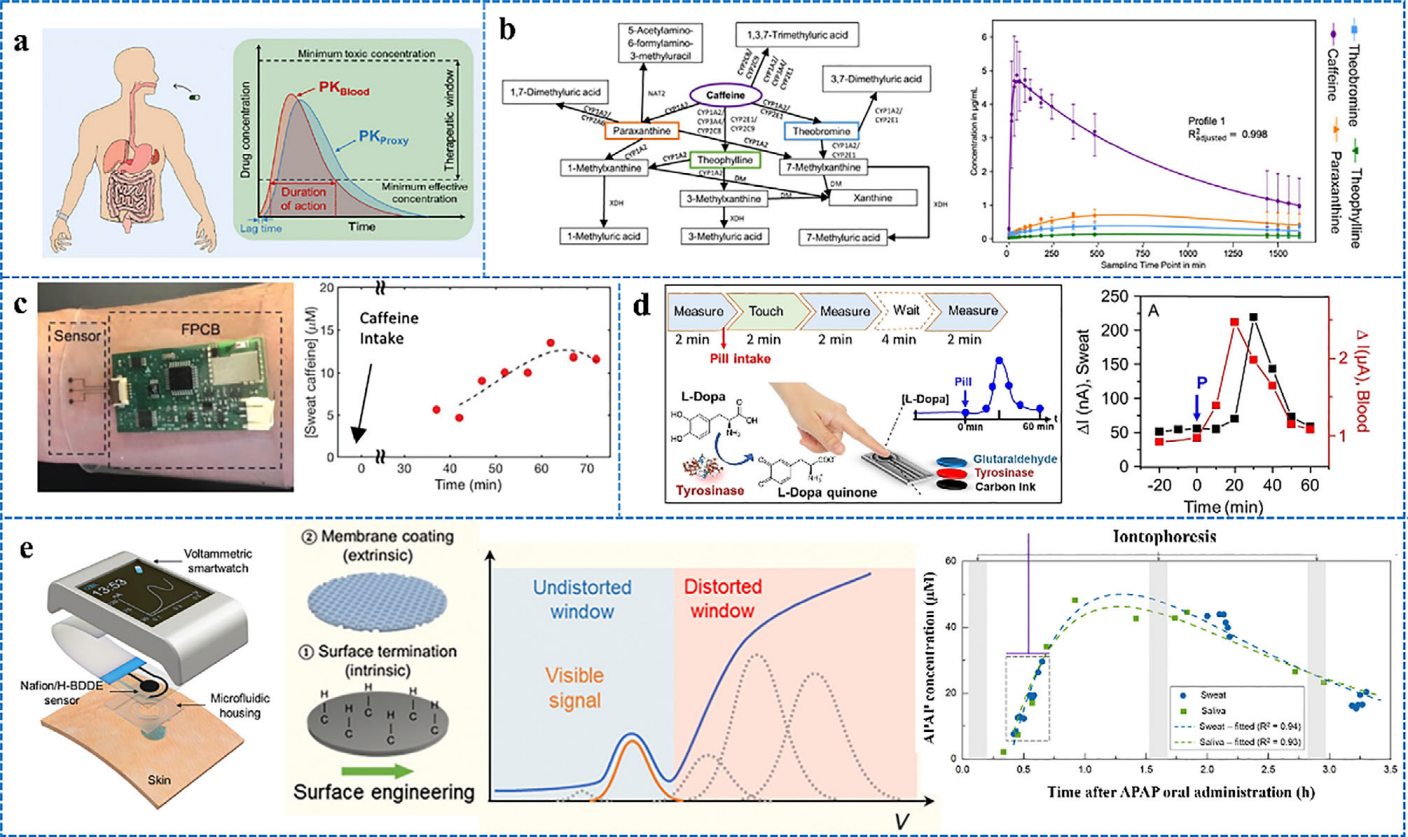
Wearable technologies for monitoring therapeutic drug levels. a) Illustration of the therapeutic range derived from drug pharmacokinetics and pharmacodynamics in blood and alternative fluids such as sweat. b) Identification of caffeine metabolites in fingertip sweat, with associated metabolic pathways revealing time‐dependent metabolic activity. c) Wearable electrochemical sensor designed for detecting caffeine, a methylxanthine compound, in real time. d) Time‐course tracking of levodopa levels in natural sweat using a fingertip‐mounted microfluidic sensor. e) Monitoring of acetaminophen absorption and clearance through sweat using a smartwatch‐integrated sensing system. Reproduced with permission.^[^ ^74^ ^]^ Copyright 2023 ACS.

Therapeutic drug monitoring (TDM) involves measuring the concentration of a drug or its associated biomarkers in body fluids to optimize drug dosage. This practice aids in maintaining drug levels within a specific therapeutic range, ensuring efficacy while minimizing side effects. TDM allows for personalized adjustments to treatment regimens for individual patients based on their unique responses to the medication.^[^ ^201^ ^]^ The development of clinical pharmacokinetics (PK) and pharmacodynamics (PD) has allowed the establishment of therapeutic ranges for drugs; this helps in optimizing drug efficacy while minimizing toxicity, ensuring safer and more effective treatments (**Figure** **17**a). The pharmacological effects of certain drugs, such as anti‐hypertensives, can be easily predicted via common measures involving the measurement of blood pressure. However, other medications present more challenges for the same. Drugs with steep dose‐response curves (theophylline),^[^ ^202^ ^]^ narrow therapeutic windows (lithium),^[^ ^203^ ^]^ or unpredictable association of dose–blood concentrations (phenytoin)^[^ ^204^ ^]^ require close monitoring and dynamic dosing for optimizing their therapeutic efficacy and reducing the risk of adverse effects. Conventional techniques for measuring drug levels in the bloodstream, including chromatographic methods (such as high‐performance liquid chromatography [HPLC]^[^ ^205^ ^]^ or liquid chromatography‐tandem mass spectrometry [LC‐MS/MS])^[^ ^206^ ^]^ and immunoassays,^[^ ^207^ ^]^ are widely utilized. Despite attempts to improve their efficacy and reduce costs, these methods continue to depend on invasive blood sampling and testing at centralized labs, which makes them less convenient for use in frequent monitoring.

A recent study on fingertip sweat profiling demonstrated that sweat is a reliable sample that can be easily collected at frequent intervals in a noninvasive manner by untrained personnel for metabolic phenotyping.^[^ ^208^ ^]^ The research utilized LC‐MS to identify different catabolic products of caffeine from the fingertip sweat samples, highlighting its potential for noninvasive metabolic analysis, as shown in Figure 17b. Additionally, a dynamic metabolic simulation network was employed to counter the impact of sweat volume in a time‐course study on caffeine metabolism. This model allowed for the estimation of sweat rate and provided insights into the patterns of caffeine metabolism by monitoring the levels of paraxanthine, theobromine, and theophylline, the three primary caffeine breakdown products, in each subject.

Research on sweat‐based monitoring of drug metabolism using wearable biosensors has hitherto been chiefly focused on electroactive drugs. An example includes the development of a wearable platform with an electrochemical differential pulse voltammetry (DPV) sensing module designed for detecting the electroactive methylxanthine drug, caffeine, as shown in Figure 17c.^[^ ^209^ ^]^ The study employed several dosages and time intervals post‐consumption to evaluate the differences in caffeine concentration in sweat; a peak in sweat caffeine was observed 60 min post‐consumption due to its absorption into the bloodstream, followed by a decrease in the levels owing to caffeine metabolism. The same team also developed a wearable electrochemical sweat band that utilized tyrosinase to detect levodopa and improve the management of Parkinson's disease.^[^ ^156^ ^]^ The long‐term use of levodopa is known to cause side effects such as motor fluctuations, dyskinesia, and dystonia. Monitoring the PK of levodopa is expected to facilitate dosage optimization for reducing these side effects during disease progression. Following the consumption of fava beans, the real‐time metabolic profiles of levodopa were analyzed using iontophoresis‐ and exercise‐induced sweat. Another study employed touch‐based detection methods for exploring personalized PK tracking by measuring levodopa concentrations in fingertip sweat and capillary blood samples following the oral intake of the drug (Figure 17d).^[^ ^210^ ^]^

The voltammetry‐based approach offers label‐free detection of electroactive drugs in biofluids; however, the electrochemical signals associated with the target can be disrupted by biofouling and affected by the presence of other electroactive species in complex biofluids, which can distort the redox measurements. A boron‐doped diamond electrode with a hydrogen‐terminated surface was generated to enhance the detection of acetaminophen and minimize the impact of other substances. This modification aids in separating the effects of tryptophan and tyrosine. Additionally, the electrode was coated with a negatively charged Nafion membrane to prevent interference from negatively charged uric acid. This selective modification allows for a more accurate detection of acetaminophen.^[^ ^211^ ^]^ The boron‐doped diamond electrode and a data processing framework capable of extracting redox peaks were incorporated into a special smartwatch. This integration allowed for real‐time drug monitoring, ensuring minute‐level precision for drug readouts, as shown in Figure 17e.

#### 4.4.6 Management of Chronic Diseases

Chronic diseases are the leading cause of death and disability, accounting for over two‐thirds of all deaths in the USA. The increasing incidence of these diseases worldwide, particularly attributable to an aging population, underscores the considerable need for healthcare options that are more cost‐effective and allow self‐management.^[^ ^212^ ^]^ Wearable sweat sensors enable long‐term, continuous, and noninvasive monitoring of physiological data and show great promise for managing chronic diseases and predicting clinical outcomes. The use of wearable sweat sensors for the management of chronic diseases has been investigated to a limited extent, with existing research chiefly focusing on the treatment of a few chronic disorders such as metabolic syndrome and diabetes; these have been discussed in the preceding sections.

The low concentration of relevant biomarkers presents one of the main challenges for the monitoring of chronic diseases. While the concentrations of biomarkers such as glucose and uric acid (for the conditions diabetes and metabolic syndrome, respectively) are typically higher than the inflammatory or cardiac biomarkers, such as cytokines and C‐reactive protein (CRP), are typically very low in blood and likely to be even lower in sweat. In addition, the available data on quantitative analysis of proteins and cytokines in sweat is considerably less than that on smaller molecules.^[^ ^213^, ^214^ ^]^ The absence of a reliable and effective method of sweat collection complicates the comparison of results across various studies. A soft, skin‐mounted microfluidic patch has been recently developed for the efficient collection of sweat (induced upon passive heating) to measure cytokine levels.^[^ ^215^ ^]^ Evaluation of the collected sweat samples from 10 individuals revealed that the levels of cytokines in sweat were not significantly affected by the sweating rate. Therefore, the cytokine levels in sweat may directly correlate with those in plasma, indicating that the noninvasive monitoring of sweat cytokines may provide insights into inflammatory responses. Recent advancements in wearable non‐faradaic impedance sensors have facilitated the detection of various inflammatory markers, hormones, and neurotransmitters, including the interleukins IL‐6, IL‐8, and IL‐10, TNF‐α, interferon‐gamma (IFN‐γ), and CRP, in natural sweat.^[^ ^216^ ^]^

### 4.5 Bioaffinity‐Based Sweat Sensors

The detection of disease‐related biomarkers in sweat can be challenging owing to their extremely low (nanomolar or lower) concentrations; the identification of trace amounts of targets such as proteins and hormones using ion‐selective and enzyme‐based sensors is fraught with challenges. This underscores the need for developing highly sensitive bioaffinity‐based sensors that can specifically target a variety of disease‐related biomarkers; this represents a crucial and much‐needed advancement for wearable sweat biosensors designed for the noninvasive monitoring of health and disease.^[^ ^217^ ^]^ Importantly, several of the bioaffinity‐based sweat sensors hitherto developed have been designed for single‐use, POCT applications and are incapable of continuous in situ monitoring. Bioaffinity‐based sensors typically contain a bioreceptor layer that identifies specific molecules and a signal transducer that converts the interaction between the receptor and target into a detectable signal. The bioreceptors commonly employed to develop wearable sweat biosensors include antibodies, receptors, nucleic acids, and biomimetic materials such as molecularly imprinted polymers (MIPs).^[^ ^218^ ^]^

#### 4.5.1 Antibody‐Based Sweat Sensors

**Figure 18:**
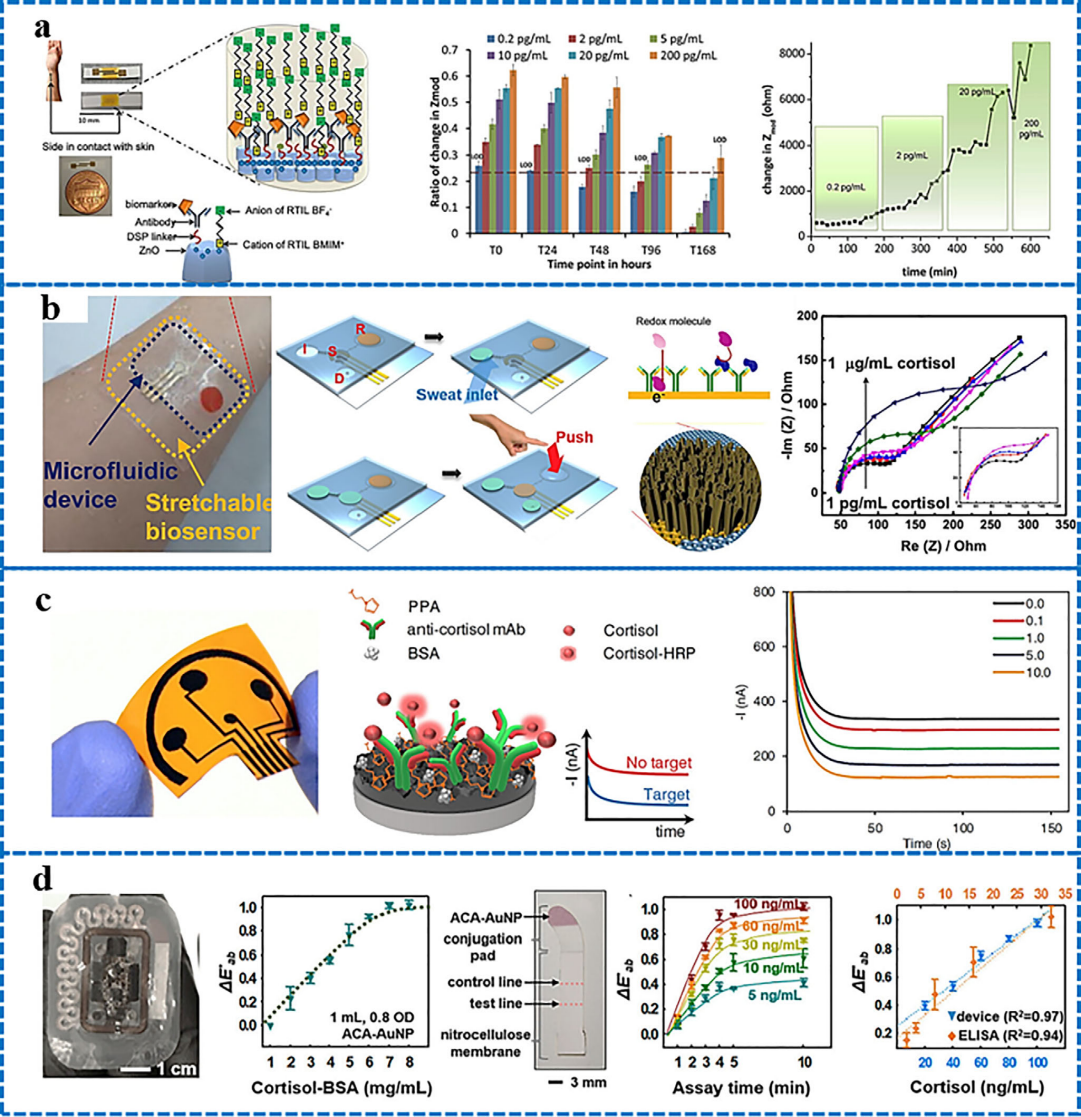
Wearable immunosensing platforms for sweat‐based cortisol monitoring. a) A non‐faradaic impedance‐based sensor enhanced with room‐temperature ionic liquid (RTIL) to improve measurement stability for cortisol detection in sweat. b) A microfluidic faradaic impedance device where detection is initiated by manually delivering redox agents from a reagent chamber to the sensing zone. c) An amperometric sensor for cortisol integrated on a flexible, laser‐patterned graphene surface. d) A colorimetric cortisol assay embedded in a soft microfluidic device, utilizing a lateral flow strip for visual detection. Reproduced with permission.^[^ ^74^ ^]^ Copyright 2023 ACS.

Antibodies are frequently employed as bioreceptors in bioaffinity‐based sensors owing to their strong affinity and specificity for target molecules, as well as their versatility and easy commercial availability. Antibody‐based sensors are of two types: labeled sensors, which employ electrochemical or optical tracers, and label‐free immunosensors. With the label‐free sensor, the binding of an antigen to the antibody is directly converted into electrical or optical signals.^[^ ^219^ ^]^ Non‐faradaic electrochemical impedance spectroscopy (EIS) is the most widely employed approach for label‐free sweat immunosensors. An EIS‐based non‐faradaic sensor was developed to detect ethyl glucuronide (EtG), an ethanol metabolite, and evaluated using human sweat samples spiked with the compound.^[^ ^220^ ^]^ The impedance was measured between two coplanar electrodes coated with either gold or zinc oxide (ZnO) and functionalized with monoclonal anti‐EtG antibodies. A detection range of 0.001 to 100 µg L^−1^ was obtained on both glass and PI substrates, with the ZnO electrodes exhibiting superior sensitivity to the gold electrodes. A similar approach was employed for developing a non‐faradaic EIS sensor using room‐temperature ionic liquid (RTIL) and nanoporous ZnO electrodes on flexible polymer membranes. This setup enhanced the stability of bioreceptors such as antibodies for the detection of IL‐6 and cortisol in human sweat samples, as shown in **Figure** **18**a.^[^ ^221^ ^]^ Clear signals were obtained with IL‐6 concentrations above the detection threshold after 168 h of storage and during continuous monitoring for 10 h. The detection range of the sensors for IL‐6 in spiked human sweat was found to be 0.2 to 200 pg mL^−1^. Furthermore, a printed two‐electrode system functionalized with anti‐CRP or anti‐interleukin‐1β (IL‐1β) antibodies was designed for nonfaradaic EIS‐based detection of the corresponding biomarkers.^[^ ^222^ ^]^ The device can detect cortisol, CRP, and IL‐1β, and has been modified with appropriate antibodies to allow the identification of other biomarkers such as IL‐6, IL‐8, IL‐10, TNF‐α, IL‐31, IFN‐γ, dehydroepiandrosterone, and neuropeptide Y (NPY) in eccrine sweat samples.^[^ ^216^ ^]^ Faradaic EIS immunosensors require redox species, making their application on the skin more challenging compared to that of non‐faradaic EIS sensors. This challenge has been overcome by the use of a stretchable microfluidic module, which delivers a preloaded redox mediator solution (such as potassium ferricyanide) to the antibody‐immobilized 3D‐nanostructured gold working electrode while simultaneously removing unbound cortisol and sweat, as shown in Figure 18b.^[^ ^223^ ^]^ The charge transfer resistance (Rct) increases at high cortisol concentrations (ranging from 1 pg/mL to 1 µg mL^−1^) in the presence of a redox mediator. A sensor utilizing ATi_3_C_2_Tx MXene‐integrated laser‐induced graphene (LIG) was developed for noninvasive POCT for cortisol. The incorporation of MXene enhances the sensitivity of the LIG electrode following its transfer onto PDMS. The Rct of the sensor was found to increase by ≈1000 Ω with an increase in cortisol levels from 10 pM to 100 nM.^[^ ^224^ ^]^

Immunosensors are often paired with direct signal‐generating labels for the detection of antigens, with the use of signal‐generating labels enhancing the detection signals and improving analytical sensitivity.^[^ ^225^ ^]^ Enzymes such as peroxidase, alkaline phosphatase, and luciferase, fluorescent markers such as fluorescein, rhodamine, and Cy5, as well as redox species such as methylene blue, ferrocene, and thionine are commonly used as labels in immunosensors. However, the need for label addition and washing steps limits the practical on‐skin application of labeled immunosensors. For example, a flexible, wireless sweat cortisol immunosensor was developed using ethyl (dimethylamino propyl) carbodiimide/*N*‐hydroxy succinimide (EDC/NHS) for antibody immobilization and electropolymerization of a carboxyl‐rich pyrrole derivative on laser‐engraved graphene electrodes (Figure 18c).^[^ ^226^ ^]^ The cortisol in sweat competes with horseradish peroxidase (HRP), which is labeled cortisol for binding to the immobilized antibody. This competition creates an inverse correlation between sweat cortisol concentration and the cathodic current generated by the enzymatic reduction of hydrogen peroxide (H_2_O_2_) in the presence of the mediator hydroquinone. The sensor was specifically developed for POCT, but necessitates additional cleaning and substrate additional steps that must be performed off‐body at a bench. Additionally, sweat biomarker detection has also been accomplished using HRP‐labeled antigens as optical tags. Various drug antibodies and drug‐HRP conjugates, including those of methadone, methamphetamine, amphetamine, and tetrahydrocannabinol, have been integrated into labeled competitive immunosensors on capillary arrays.^[^ ^227^ ^]^ The drugs were detected in artificial sweat using a chemiluminescent substrate and subsequent imaging with a CMOS camera. However, the integration of the capillary array and bulky CMOS camera with epidermal systems poses challenges due to the rigid structure of the components. This issue was addressed by integrating a flexible microfluidic system designed for skin interfacing with a lateral flow immunoassay for collecting and analyzing cortisol in sweat (Figure 18d).^[^ ^228^ ^]^

#### 4.5.2 MIP‐Based Sweat Sensors

MIPs are biomimetic receptors with binding affinities resulting from the self‐assembly of monomers around a template via covalent or noncovalent interactions, followed by polymerization to develop a mold‐like shell.^[^ ^229^ ^]^ The elimination of patterns from the polymer creates specific binding sites that enable selective recognition of target molecules. MIPs offer a cost‐effective, mass‐producible, and durable alternative to conventional bioreceptors such as antibodies, enzymes, and aptamers. Since MIPs often lack inherent signaling or catalytic capabilities, the development of signaling mechanisms that respond to the interaction between MIPs and their templates is crucial for the successful design of MIP‐based sensors.

In electrochemical MIP‐based sensors, recognition events typically cause changes in the dielectric properties at the electrode interface, and signals are detected in the presence of electroactive species. For example, a flexible electrochemical platform was developed to detect urea in sweat using potassium ferricyanide as a redox mediator.^[^ ^230^ ^]^ The binding of urea to the recognition sites on urea‐imprinted PEDOT within a CNT network and a gold nanotube (AuNT) structure obstructed electron transfer at the potassium‐ferricyanide probe. This interaction caused a detectable change in the DPV signal. The MIP sensor was found to exhibit a highly linear response and selectivity at physiologically significant urea concentrations. The same research group developed a flexible electrochemiluminescence (ECL)–based sensor by imprinting urea and lactate onto Ru (II)‐polyethylenimine (PEI)@SiO_2_ immobilized on AuNTs networks.^[^ ^231^ ^]^ The porous MIP membrane aided electron transfer for the electrochemical oxidation of Ru (II)‐PEI@SiO_2_, leading to ECL emission. However, these electron transfer routes are gradually blocked when the pores, which function as binding sites, are filled with target molecules, leading to a decrease in ECL signals. The ECL platform successfully enabled the sampling and detection of sweat urea and lactate and exhibited excellent on‐skin stability. Additionally, a lactate sensor for sweat samples employing MIP‐coated silver nanowires (AgNWs) on a screen‐printed electrode was also evaluated. The filling of imprinted cavities on the MIP with lactate molecules decreased the oxidation current at the AgNWs, indicating successful detection.^[^ ^232^ ^]^

#### 4.5.3 Nucleic Acid–Based Sweat Sensors

A novel class of bioreceptors known as aptamers was generated via the in vitro selection of single‐stranded nucleotides that exhibited binding affinities for specific targets. These aptamers have been developed to target a wide range of substances, including metal ions, small molecules, proteins, and even whole cells, based on their unique 3D structures, which often involve stem or loop formations.^[^ ^233^ ^]^ Aptamers present several advantages over antibodies, including easy and cost‐effective production, minimal variability between batches, non‐immunogenicity, simplicity of functionalization with various groups, and enhanced stability.^[^ ^234^, ^235^ ^]^ These unique features make aptamers well‐suited for various sensing technologies, particularly in POCT applications.

**Figure 19:**
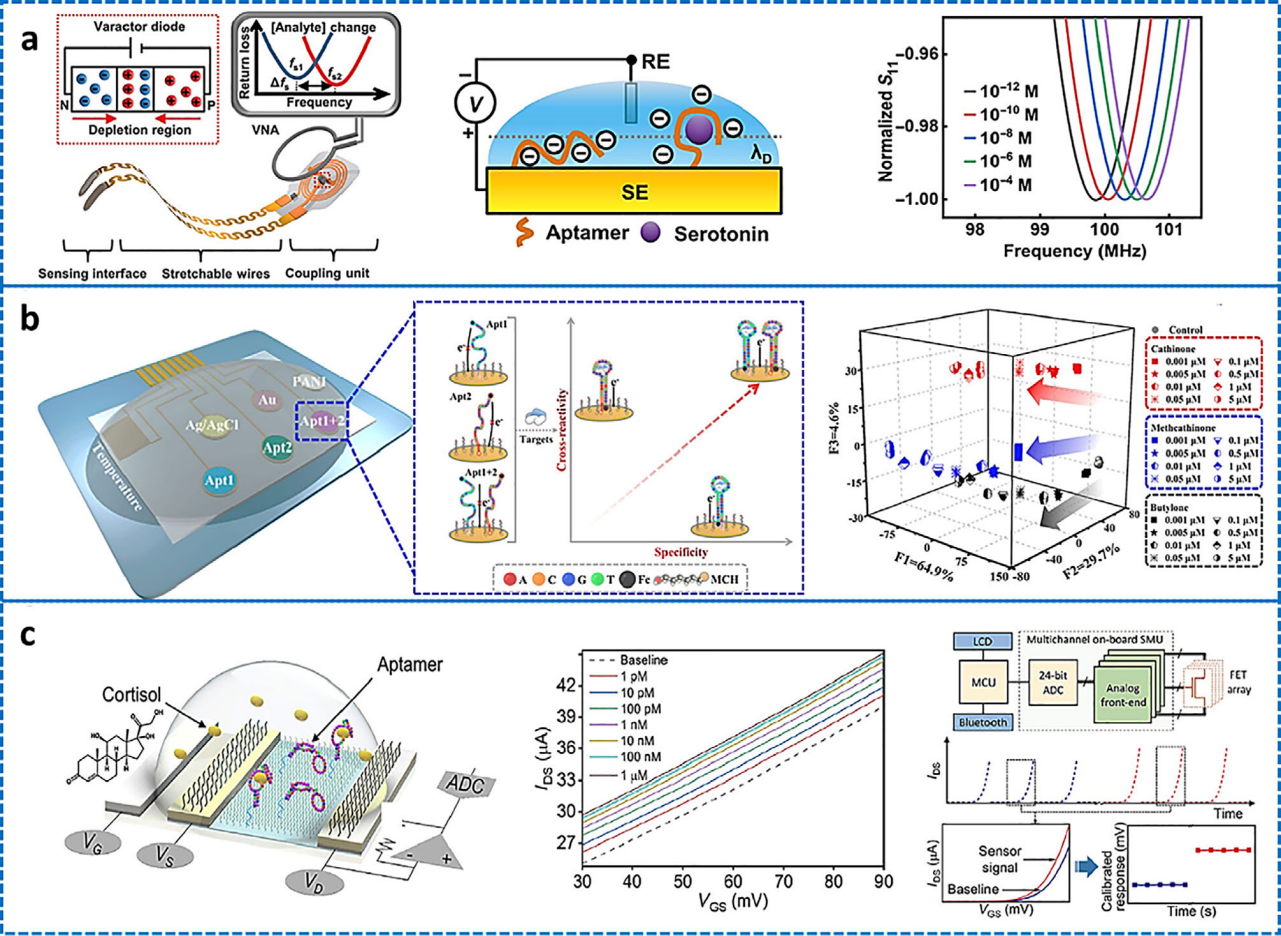
Wearable aptamer‐based sensors for biomolecular detection. a) A wireless aptasensor, inspired by a tuning circuit and fabricated on a gold electrode, enables the monitoring of serotonin levels in biofluids. b) A dual‐methylene‐labeled aptamer array designed for multiplexed detection of commonly abused drugs in synthetic sweat samples. c) A field‐effect transistor (FET) aptasensor utilizing a thin indium oxide film (In_2_O_3_) for sensitive cortisol detection in sweat. Reproduced with permission.^[^ ^74^ ^]^ Copyright 2023 ACS.

As an example, cortisol aptamers were modified at the 5′‐terminus with a thiol group and attached to ZnO nanoporous electrodes, which have been previously employed in non‐faradaic EIS‐based cortisol immunosensors.^[^ ^236^ ^]^ Instead of impedance determination, chronoamperometry was conducted by applying a potential step of 0.35 V (over the range 0.35 to −0.35 V) for 60 s. The resulting changes in steady‐state current reflected variations in the capacitance of the non‐faradaic double layer with an increase in cortisol concentrations. The same research group developed a platform for the simultaneous detection of cortisol and NPY using aptasensors on porous gold electrodes.^[^ ^237^ ^]^ Non‐faradaic EIS was employed for observing the dose responses of both targets to obtain detection ranges of 1–256 ng mL^−1^ and 1–256 pg mL^−1^ for cortisol and NPY, respectively. Furthermore, a tuning‐circuit design–inspired wireless serotonin aptasensor that employed the principles of non‐faradaic impedance was developed using gold electrodes, as shown in **Figure** **19**a.^[^ ^238^ ^]^ The versatility of chemical modifications on aptamers has allowed the development of various tracer‐labeled sensing technologies. For example, cortisol aptamers were functionalized with a thiol group at one end and redox label (methylene blue) at the other, followed by immobilization on gold electrodes for cortisol detection (Figure 19b).^[^ ^239^ ^]^ The binding of aptamers with the target results in conformational changes in the former due to stretching, which reduces the distance between the redox species and the electrode surface. This proximity enhances the electron transfer rate, resulting in an increase in the peak current, which can be monitored using square‐wave voltammetry (SWV). A similar strategy was applied for designing an integrated aptasensor array for drug detection.^[^ ^239^ ^]^ FETs represent another highly used transducer system that has been paired with aptamers for detecting biomolecules in sweat. A recently discovered cortisol aptamer was immobilized onto a flexible, thin In_2_O_3_‐based FET for this purpose, as shown in Figure 19c.^[^ ^240^ ^]^ The binding of aptamers with the target and the ensuing changes in the conformation of the former result in a reorganization of their negatively charged framework. This shift alters the surface charge of the FET, resulting in measurable changes in the gate voltage and source–drain current, allowing for quantitative detection of the target. An integrated device with an on‐board multichannel source measurement unit was designed for on‐body applications. In a similar vein, cortisol aptamers were immobilized onto electrospun conducting polyacrylonitrile (PAN) nanofibers that had been coated with carboxylated PEDOT, forming a liquid‐ion gated FET system on PET.

## 5 Is Sweat a Suitable Biofluid for Medical and Clinical Diagnostics Applications?

The use of biofluids for non‐invasive diagnostic applications in personalized and preventive healthcare has recently gained significant interest. The accessibility and ability of biofluids, such as sweat and ISF, to potentially provide critical and physiologically relevant data make them prominent candidates among available biofluids. Sweat is a readily available biofluid that offers localized information about hydration, electrolytes, and stress markers. However, its composition is highly variable and influenced by external and individual‐specific factors, limiting its reliability for systemic diagnostic applications. By contrast, the composition of ISF closely mirrors that of blood plasma, and the biofluid functions as an intermediary between capillaries and cells. This makes ISF a robust medium for the analysis of systemic biomarkers, including glucose, proteins, and electrolytes. Recent advances in wearable technologies, particularly in microneedle‐based systems, have enabled painless, virtually non‐invasive, and efficient extraction of ISF by overcoming challenges associated with conventional sampling methods.

**Figure 20:**
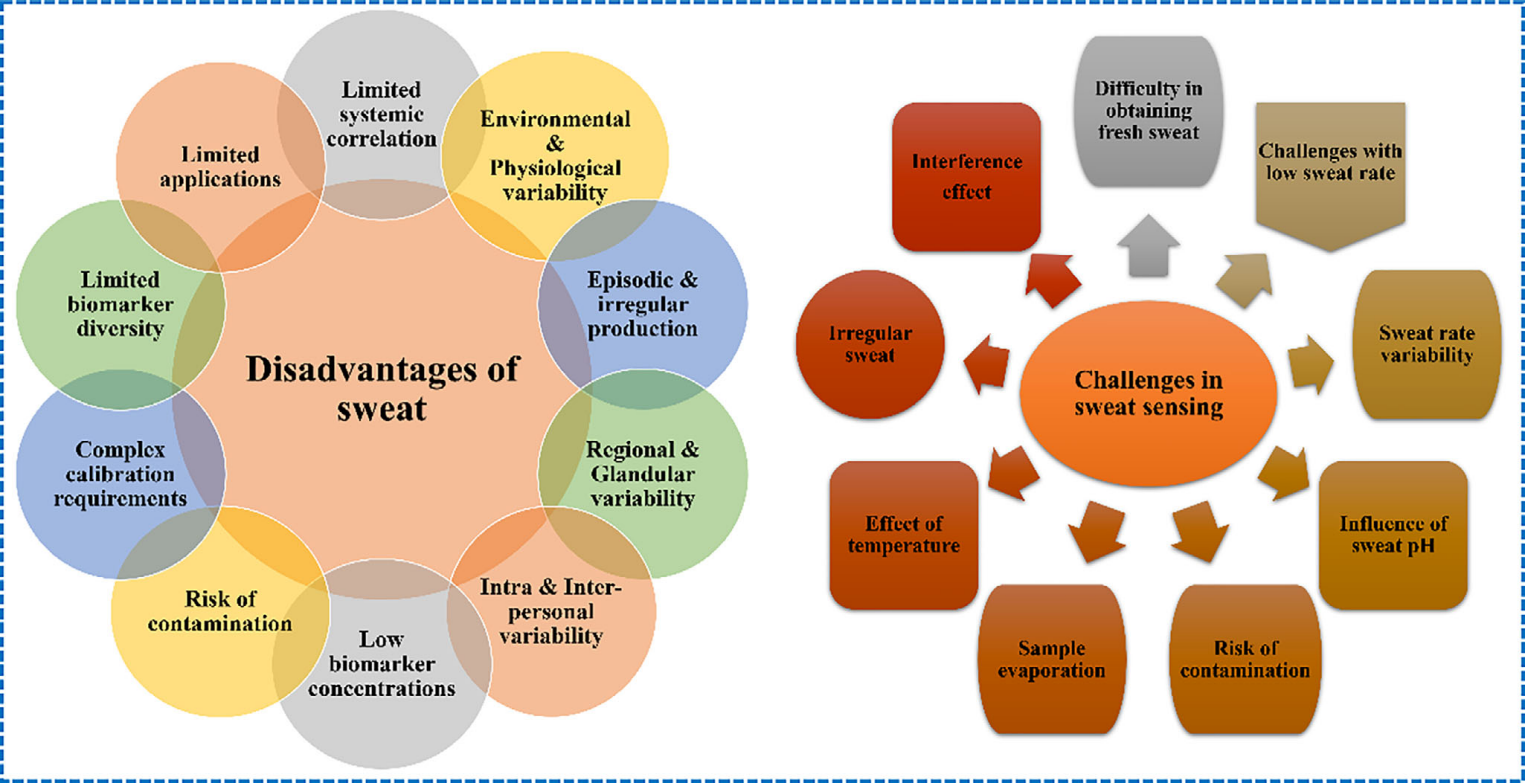
Disadvantages of sweat and challenges of sweat‐based sensing approaches.

Still, despite the non‐invasive nature of current sweat‐based diagnostics platforms, several challenges limit their reliability and effectiveness, particularly for systemic and clinical applications. These disadvantages are clearly outlined in **Figure** **20**.

### 5.1 Limited Systemic Correlation

- Levels of sweat biomarkers such as glucose and lactate do not consistently reflect blood plasma levels.
- The diffusion of analytes into sweat is influenced by complex physiological processes, leading to weaker correlations of analyte concentrations in sweat with systemic conditions such as diabetes or metabolic disorders.

### 5.2 Environmental and Physiological Variability

- External factors such as temperature, humidity, and physical activity significantly affect sweat composition.
- Physiological states such as hydration status or dehydration alter sweat production, impacting biomarker levels.

### 5.3 Episodic and Irregular Production

- Sweat production is not continuous and often depends on stimuli (exercise) or pharmacological agents (pilocarpine).
- Lack of constant production limits the applicability of sweat for real‐time, continuous health monitoring.

### 5.4 Regional and Glandular Variability

- Sweat composition varies across different body regions due to differences in gland density and function.
  ◦ **Palms and soles**: Sweat glands primarily excrete water and electrolytes.
  ◦ **Axillary region**: Sweat glands additionally excrete proteins and lipids.
- Site‐specific variability complicates the development of standardized wearable devices.

### 5.5 Intra‐ and Inter‐Personal Variability

- Intrapersonal: Daily schedule, diet, and physical activity cause fluctuations in sweat composition.
- Interpersonal: Age, gender, fitness level, and genetics lead to significant differences.

### 5.6 Low Biomarker Concentrations

- The concentrations of biomarkers such as glucose, proteins, and metabolites are much lower in sweat than in plasma or ISF.
- High sweat rates lead to further dilution of biomarkers and reduce the accuracy of diagnosis.

### 5.7 Risk of Contamination

- Sweat collected on the skin surface is vulnerable to contamination with environmental particles, skin secretions, cosmetics, and personal care products.
- Such contamination can interfere with sensor readings, leading to unreliable results.

### 5.8 Complex Calibration Requirements

- The effects of sweat rate, environmental conditions, and regional variations on sweat composition/biomarker levels necessitate advanced calibration algorithms to ensure accuracy of measurement, increasing the complexity of wearable devices and potential for errors during usage.

### 5.9 Limited Biomarker Diversity

- Sweat contains a narrow range of biomarkers that primarily include electrolytes (Na^+^ and K^+^ ions), small molecules (lactate and urea), and stress hormones (cortisol).
- The absence of key systemic markers such as large proteins or nucleic acids restricts the application of sweat to diagnose complex conditions such as cancer or autoimmune diseases.

### 5.10 Limited Applications in Acutely and Critically Ill Patients

- Sweat production may be reduced or absent in patients experiencing acute dehydration, fever, or shock.
- This inconsistency makes sweat unsuitable for real‐time monitoring in critical care settings where precision and reliability are crucial.

Based on the aforementioned discussions, as well as in Sections 3 and 4, although sweat‐based diagnostics may potentially offer accessibility and truly non‐invasive sampling, their clinical utility is hindered by variability in sweat composition, low systemic correlation for most biomarkers, irregular sweat production, body site‐specific differences, and susceptibility to environmental and physiological influences, all of which are compounded by low analyte concentrations, dilution effects under high sweat rates, contamination risks, and the need for complex calibration algorithms. As a result, sweat is most valuable for localized and non‐clinical monitoring applications such as hydration status, electrolyte loss, and stress marker assessment, rather than for disease monitoring and diagnosis. In contrast, ISF, due to its biochemical similarity to blood plasma and its biofluid role as the intermediary between capillaries and tissue cells, offers a composition that more accurately reflects systemic biomarker levels, including glucose, proteins, and electrolytes, making it highly suitable for clinical diagnostics. Recent advances in minimally invasive indwell microneedle‐based platforms have enabled efficient, painless ISF extraction, overcoming the limitations of conventional sampling methods, and supporting their integration into wearable devices for real‐time, reliable, and personalized health monitoring, thereby positioning ISF as a superior biofluid for precision diagnostics and preventive healthcare compared to sweat.^[^ ^19^ ^]^

## 6 Challenges in Sweat‐Based Sensing Approaches

Despite considerable advancements in wearable sweat‐sensing technology, numerous issues continue to be associated with the use of these sensors, as shown in Figure 20. The incorporation of precise details is crucial due to differences in the biological cycle as well as physio‐psychological parameters between individuals, as explained in Section 3.1. In addition, the development of wearable sweat sensors involves focusing on various issues, such as inconsistency of sweat production, contamination of sweat samples, and evaporation. Additionally, sweat rate varies between individuals in accordance with several internal and environmental variables, as mentioned in Section 3.2.

Sweating is an unpredictable phenomenon, with each individual exhibiting different rates of sweat secretion that can also vary across several body areas (see Section 3.1.1). The sweat rates change according to physical activity and fluctuate over the range 0.02–20 nL min^−1^ gland^−1^. The eccrine sweat glands produce very little sweat in certain regions, limiting sample volumes and making the accurate analyte profile measurements a challenging task. Glucose in sweat is ≈100 times more diluted than in ISF or blood plasma (Table 3), and accurate measurements of such low concentrations present challenges. This can be addressed by employing highly selective and ultrasensitive sensors to detect even minor fluctuations in glucose levels. Advanced microelectrodes enhanced with appropriate nanomaterials or electrocatalysts can help improve detection platforms' sensitivity and effectiveness for measuring small glucose concentrations. The key factors that influence sweat secretion rates include heat stress, hydration status, and physical activity, as mentioned in Section 3.3. Previous studies have shown that an increase in sweat rate leads to higher concentrations of certain ions, such as Na⁺ and Cl^−^ in sweat; however, the decrease in time for the accumulation of larger molecules at higher sweat rates lowers their concentration in sweat. These circumstances affect the detection of target biomarkers and need to be accounted for during measurements. Furthermore, the evaporation of sweat during sensing can alter the concentration of analytes, leading to inaccurate results. This may be addressed by accelerating the detection process and minimizing exposure to the environmental conditions of the sweat sample. Additionally, sweat is often contaminated with exogenous chemicals such as cosmetic products or environmental pollutants, which can skew the results. This may be resolved by designing the system to minimize the direct contact of the sweat sample with skin (see Section 3.6). Additionally, fresh sweat frequently mixes with previously secreted sweat as it moves to the sensing membrane, leading to an averaging of biomarker profiles rather than real‐time measurements and potentially inaccurate results. The use of microfluidic channels addresses this issue by facilitating the transport of sweat in real‐time. Additionally, challenges such as interference from fluid components, cross‐reactivity, and signal disruption plague biofluid‐sensing systems. Interference from fluid components may be reduced by focusing on the sensor design and properties of the specific material during the fabrication of wearable sweat sensors. Various composite nanomaterials based on metal oxides have demonstrated excellent sensing capabilities (sensitivity, specificity, etc.). Previous studies have shown that the sensitivity of these metal oxides can be considerably enhanced by doping with rare‐earth ions. For example, Nurdan et al. incorporated dysprosium, a rare‐earth element, into ZnO/CuO films, improving their sensing performance and making the material suitable for real‐time monitoring of hydration status.^[^ ^241^ ^]^

Moreover, sweat rate variability is a significant factor in precisely interpreting sweat‐based biomarker measurements. It directly affects biomarker dilution (analyte concentration) due to dynamic dilution effects. The rate of sweat production determines the fluid's transit time through the eccrine duct, which in turn influences the degree of solute reabsorption and concentration changes before collection. At higher sweat rates, often caused by exercise, heat stress, or pharmacological stimulation, the rapid secretion shortens the time the sweat spends in the duct. This reduction limits the opportunity for selective electrolyte reabsorption, resulting in lower measured concentrations of biomarkers such as sodium, chloride, lactate, and various metabolites. On the other hand, during slow sweat production, prolonged transit time can lead to excessive reabsorption or localized concentration effects, producing artificially elevated biomarker readings. This sweat rate‐dependent dilution and concentration phenomenon disrupts the linear relationship between sweat and systemic analyte levels, which limits the clinical applicability of raw concentration data. Without compensatory strategies, such as real‐time sweat rate monitoring, volumetric normalization, or advanced calibration algorithms. These fluctuations introduce significant variability between individuals and within the same individual over time. This variability can compromise the sensitivity and specificity of diagnosis. Therefore, accounting for sweat rate variability is essential for the development of robust and clinically reliable sweat biosensing platforms.^[^ ^242^ ^]^ Therefore, exploring ISF as a biofluid effectively addresses the abovementioned concerns, ensuring more accurate predictions and monitoring of health conditions.

## 7 Characteristics of Interstitial Fluid (ISF)

**Figure 21:**
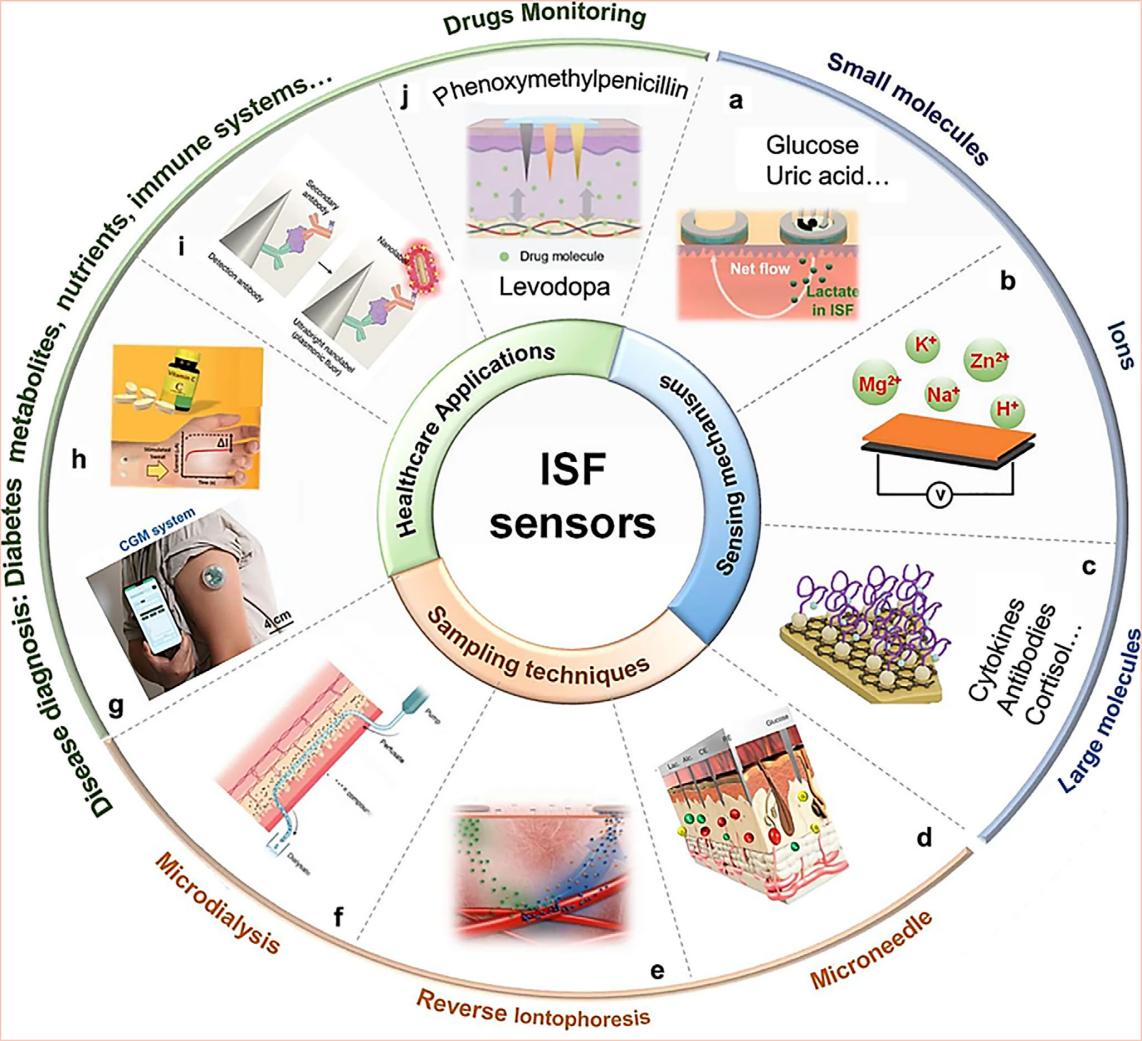
Overview of ISF‐based wearable biosensors for healthcare applications. a–c) Different sensing mechanisms for small, medium, and large molecules, d–f) ISF extraction sampling techniques, g–j) Real‐time healthcare applications to monitor the ISF‐based biomarkers. (a) Small molecule sensing mechanism (Glucose, Uric acid, etc.), (b) Ion‐sensing mechanisms (Na+, K+, Mg+, etc.). (c) Large molecule sensing mechanisms (Cytokines, antibodies, cortisol, etc.), (d) Microneedle‐based sampling extraction methods to access ISF to detect biomarkers (solid, hollow, porous, hydrogel, etc.). (e) A reverse Iontophoresis‐based sampling method to withdraw ISF without penetrating the skin stratum corneum. (f) Microdialysis mechanism for ISF extraction to identify the biomarkers. (g) Continuous glucose monitoring (CGM) by accessing the ISF‐based microneedle patch for real‐time diabetes patient management. (h) Nutrient monitoring using ISF for real‐time applications. (i) Cytokine and protein measurements by microneedle ISF technique. (j) Drug monitoring using microneedle‐based technologies. Reproduced with permission.^[^ ^40^ ^]^ Copyright 2024 Nature.

ISF is formed in extracellular spaces via direct diffusion from arteries, which makes it highly valuable for healthcare and biomedical applications, as shown in **Figure** **21**. ISF can be easily accessed via the skin, and its composition closely mirrors that of blood; additionally, it contains a range of biomarkers that can be detected using ISF‐based wearable biosensors.^[^ ^243^ ^]^ This enables the provision of health data critical for both clinicians and patients.^[^ ^40^, ^244^ ^]^ Blood continues to be the most commonly employed biofluid for diagnostic purposes and is often regarded as the gold standard for the detection of a wide range of biomarkers; however, sample collection is invasive, requiring the use of hypodermic needles and professional expertise to ensure safe extraction. By contrast, the abundance, low bio‐interference, and strong clinical relevance of ISF add to its value for use in diagnostic applications. To derive maximum benefits from the application of ISF‐based sensors in the healthcare sector, studies have extensively focused on developing methods for obtaining information from ISF reliably and simply, with minimal invasiveness. This includes the development of microneedle techniques (Figure 21d–f), such as reverse iontophoresis (RI)^[^ ^245^ ^]^ and microdialysis^[^ ^246^ ^]^ for sampling or accessing ISF, and the measurement of biomarkers in ISF, such as metabolic compounds and drugs, for the diagnosis and management of diseases (Figure 21g–j).^[^ ^247^ ^]^

ISF proves stronger clinical relevance for quantitative biomarker assessment than sweat due to its close physiological alignment with blood composition and reduced susceptibility to external variability. As ISF is in direct equilibrium with the capillary network, its biomolecule concentrations, such as glucose, electrolytes, and other small metabolites, closely reflect blood plasma levels (see Table 3), with predictable diffusion kinetics, enabling accurate calibration against standard clinical assays. This biochemical stability supports reliable, continuous, and point‐of‐care monitoring with minimal temporal lag. In contrast, sweat‐based biomolecule composition is highly variable, influenced by factors such as sweat rate, gland type, environmental conditions, skin contamination, and regional secretion heterogeneity. These all hinder reproducibility and quantitative accuracy without complex normalization.^[^ ^248^ ^]^ ISF's stable analyte profile (see Table 3) and lower environmental interference make it a more robust and clinically valid biofluid for quantitative diagnostics, enhancing its suitability for regulatory acceptance and clinical deployment. A comparative analysis of metabolites, hormones, and electrolytes in sweat, ISF, and blood shows distinct concentration profiles influenced by factors such as molecular weight, membrane permeability, and transport mechanisms. Among these fluids, metabolite concentrations are generally highest in blood and ISF, which reflects their critical roles in maintaining systemic homeostasis. In contrast, sweat usually contains more diluted substances due to glandular filtration and extracellular exchange processes. Compared with other biofluids, the detailed biomolecule concentrations in ISF are tabulated in Table 3.

## 8 Mechanisms of ISF Extraction and Collection

ISF is a valuable source for detecting unique biomarkers due to clotting factors' absence. It offers important biochemical data such as the concentrations of electrolytes, proteins, peptides, and metal ions. Obtaining an understanding of the movement of ISF within the human body is crucial for its collection and application in ISF‐based sensors; the mechanism governing the transport of fluid and solutes through tissues was first described in Starling's hypothesis.^[^ ^249^ ^]^ The theory proposes that fluid is transferred from capillaries to the surrounding tissue space at a vessel's arterial end and reabsorbed at the venous end. The properties of ISF are those of an incompressible Newtonian fluid, and its viscosity is influenced by factors such as pressure, temperature, and the presence of exogenous chemical compounds.

The dermis includes various types of cells, such as fibroblasts, macrophages, adipocytes, mast cells, Schwann cells, and stem cells. Fibroblasts are the primary type of cells within the dermis. The mast cells are multifunctional immune cells with several functions and are mostly found in connective tissues and around the capillaries.^[^ ^250^, ^251^ ^]^ The growth factors released by mast cells facilitate the proliferation and migration of fibroblasts. The mast cells also facilitate collagen synthesis within the surrounding extracellular matrix and play a crucial role in mediating inflammatory responses and boosting immune functions in the dermis.^[^ ^252^ ^]^

The movement of ISF in the extracellular space can be simulated by considering mast cells, which have a thin layer on their surface called the brinkman boundary layer. The interstitial space is filled with collagen fibrils that act as porous media, allowing for the flow of ISF. The activation of mast cells releases chemical mediators from their granules into the extracellular matrix, which triggers various biological responses.^[^ ^253^ ^]^ The flow characteristics of ISF can be effectively described using the Brinkman and continuity equations.^[^ ^254^ ^]^

**Figure 22:**
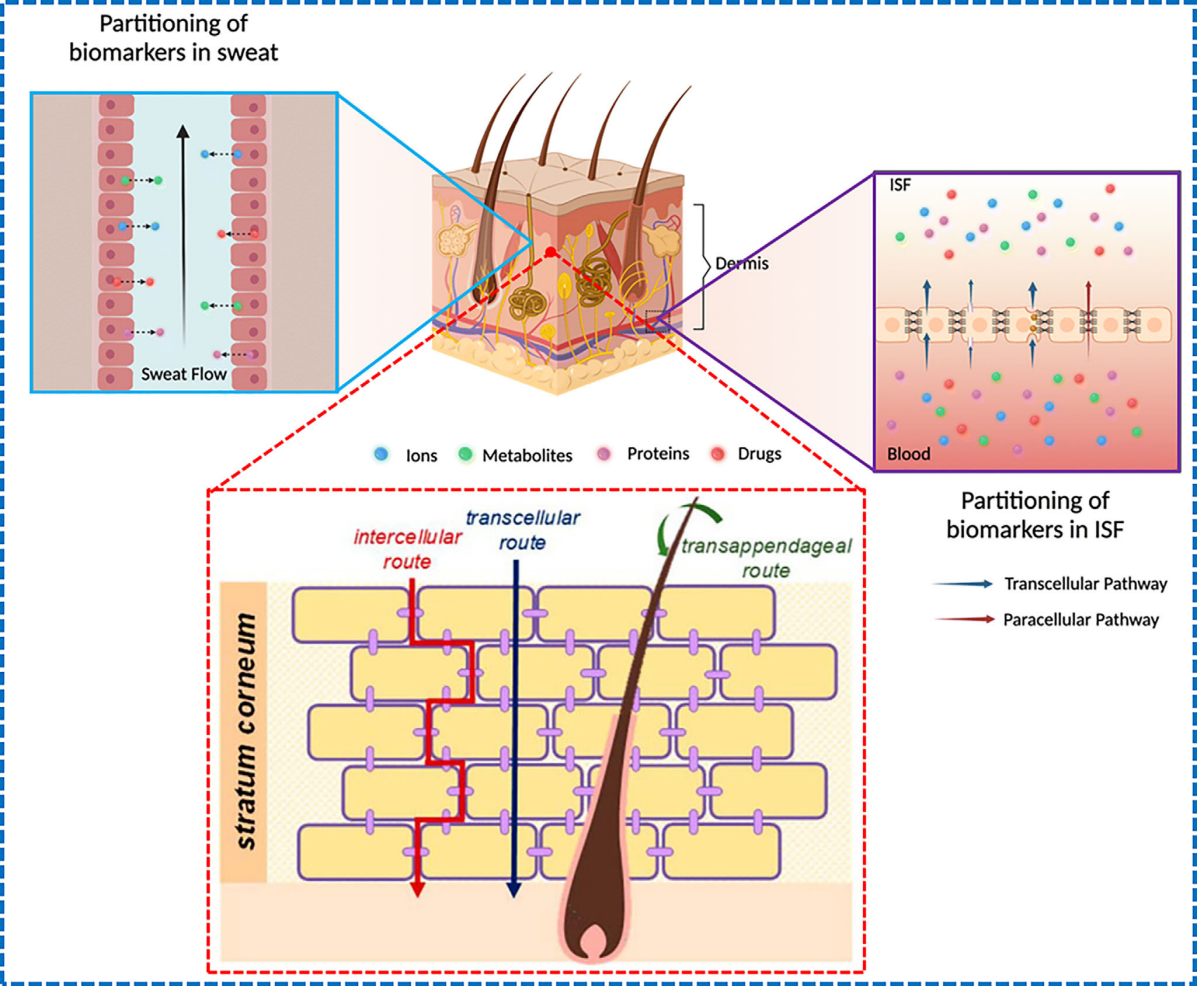
A schematic representation of the human skin and pathways of sweat and ISF, partitioning of biomarkers in sweat and ISF. Inset: represents the three possible routes for the permeation of fluids. These pathways can be employed for ISF sampling/extraction. Reproduced with permission.^[^ ^19^ ^]^ Copyright 2023 RSC.

A few possible approaches for the collection/extraction and analysis of ISF are presented in Figure 21. A schematic representation of these pathways in a skin model is illustrated in **Figure** **22**. The transcellular pathway involves passing through the cells to extract or directly analyze ISF;^[^ ^255^ ^]^ An example includes the use of needles for injections, where the needle passes through alternating hydrophilic and lipophilic layers to reach the dermal region. By contrast, the transcellular route involves navigating the space between cells, which is composed of cholesterol, ceramides, and free fatty acids. An option involves the use of appendageal pathways such as hair follicles and glands; however, this route is typically disregarded for ISF collection due to the small size and limited distribution of these structures in human skin.

A few studies have demonstrated that the permeation and extraction of fluids via the appendageal pathway is more efficient than that obtained in areas without appendages. This is particularly relevant for transdermal drug delivery, as hair follicles act as natural reservoirs located within the epidermal layer.^[^ ^256^ ^]^ Therefore, there is potential to exploit this natural pathway for the extraction and analysis of ISF for subsequent research. However, pinpointing the precise location of hair follicles in different individuals remains difficult. In short, the various methods (indirect/direct) for extracting and monitoring ISF include suction blistering, iontophoresis, microdialysis, sonophoretic extraction, and microneedle array patches.^[^ ^145^, ^257^ ^]^

### 8.1 Suction Blister Technique for ISF Extraction

The suction blister technique is a pivotal dermatological procedure that employs controlled negative pressure to induce blister formation, facilitating the collection of ISF samples. Recognized for its minimal invasiveness, it preserves the deeper skin strata, enabling a detailed analysis of immunological responses and cutaneous physiology. Its application is widespread in both clinical research and experimental dermatology as it offers a refined approach for investigating epidermal dynamics with reduced risk of scarring and complications. Kistala et al. used the suction blister technique to investigate the mechanisms underlying blister formation. This technique facilitates the separation of the epidermal layer from the dermis in human skin for analysis. The suction blister model was subsequently utilized to extract ISF from the skin. This technique requires the application of negative pressure (ranging from 100 to 200 mmHg) along with elevated temperatures for extended periods (60 to 90 min) on the skin. The pressure leads to the formation of a blister between the dermis and epidermis, which is gradually filled with dermal ISF. The suction blister technique is commonly employed for investigating the wound‐healing properties of epidermis and treatments for skin diseases. However, this method is associated with risks of significant injury, infections, and bleeding, often necessitating recovery for weeks. Additionally, fluid absorption through suction blisters is unfavorable to precise quantitative measurements and requires larger sample volumes.^[^ ^258^ ^]^ Yu et al. introduced a method for extracting ISF using a micro‐vacuum generator integrated with a microfluidic system.^[^ ^259^ ^]^ The porosity of porcine skin was enhanced via low‐frequency ultrasound treatment prior to ISF extraction. The microfluidic system was equipped with a micro pneumatic valve for regulating flow within the micro‐chamber, and a flow sensor was employed to measure the volume of extracted ISF. A brief comparison of invasiveness, limitations, reliability, sample volume, etc., of ISF sampling techniques is formulated in **Table** **4**.

**Table 4:** Comparison of ISF sampling techniques about invasiveness, limitations, reliability, and sample volume.^[^ ^17^ ^]^

S.No	ISF sampling technique	Invasiveness	Reliability	Limitations	Sample required	Anesthesia	Time‐consuming
1	Microneedle Extraction	Low‐penetrating stratum corneum (≤700 µm) with minimal pain	High for small molecules; good blood correlation	Limited sample volume; easy patch replacement after hours	Few µL	Not required	Low (minutes)
2	Reverse Iontophoresis	Very low‐ mild current extracts ISF	Moderate; affected by skin properties	Low efficiency for large molecules; signal delay; possible irritation	<5 µL/hr	Not required	Moderate (requires device run time)
3	Suction blister fluid	Moderate–High ‐blister formation via suction	Good for proteins/ metabolites	Pain; unsuitable for frequent use	50‐200 µL	Often required	High (>30 min to form blister)
4	Hydrogel‐based extraction	Low‐passive absorption through skin	Moderate; slow uptake	Low analyte concentration; not real‐time	Few µL over hours	Not required	High (hours)
5	Sonophoresis	Low‐Moderate ultrasound enhances permeability	Good for small molecules	Requires an ultrasound device; heating risk; skin‐type variability	Few‐tens µL	Not required	Low‐Moderate (minutes)
6	Microdialysis	Moderate–High ‐probe with membrane inserted into dermis/subcutis	Very high; continuous and quantitative	Invasive; perfusion pump needed; local tissue reaction possible	0.3‐2 µL/min	Local optional	High (setup + monitoring)
7	Thermal ablation	Moderate‐micro‐heaters/lasers remove stratum corneum	High for rapid sampling	Energy source needed; possible skin irritation	Few‐tens µL	Not required	Low‐Moderate (minutes)
8	Open flow microperfusion (OFM)	Moderate‐catheter in dermis	Very high; continuous sampling possible	More invasive; trained operator needed	10‐50 µL/min	Local optional	Moderate‐High (setup + monitoring)

### 8.2 Iontophoresis‐Based Methods for ISF Extraction

The iontophoresis‐based method of ISF extraction involves the application of an electric current to the epidermal layer of the skin to enhance the circulation of ISF. This charge generates an electroosmotic flow of solvent from the anode to the cathode, facilitating the movement of ISF across the skin.^[^ ^260^ ^]^ A commercially accessible device known as GlucoWatch employs iontophoresis to monitor glucose levels using integrated biosensors continuously. However, these studies have shown that prolonged use of the device leads to skin irritation; additionally, the system was associated with a higher false‐positive rate, resulting in its eventual withdrawal from the market. By contrast, Kim et al. developed a needle‐free device based on reverse iontophoresis (RI) and compared its effectiveness with that of conventional continuous glucose monitoring (CGM) devices that involved the use of needles.^[^ ^261^ ^]^ The RI‐based device simultaneously analyzed sweat samples at an anode using glucose oxidase biosensors while extracting and analyzing ISF at a cathode using alcohol oxidase biosensors within a wearable epidermal platform. The device's performance with respect to monitoring changes of alcohol levels in sweat and glucose levels in ISF in human subjects was assessed following the consumption of food and alcohol. The results indicated reliable estimation of glucose levels in the ISF, although with a delay of a few minutes; however, repeated use of the system resulted in skin injuries. Additionally, Kim et al. presented a screen‐printed electrochemical tattoo patch pathway that employed iontophoresis‐based extraction of ISF.^[^ ^18^ ^]^ These tattoo patches, designed with a specific design, contained flexible electronic devices and functioned as a glucose sensor; they effectively detected real‐time changes in glucose levels following food and alcohol consumption. However, the study did not evaluate the long‐term effects of iontophoresis‐based extraction.

### 8.3 Sonophoresis‐Based Methods of ISF Extraction

Sonophoresis involves the application of low‐frequency ultrasound to skin, generating cavitation bubbles that enhance skin permeability; coupling the technique with vacuum pressure leads to a considerable increase in skin permeability and allows the noninvasive extraction of ISF within 5 to 15 min. Additionally, the enhanced skin porosity obtained with ultrasound pretreatment can be maintained for up to 42 h when the treated area is covered.^[^ ^262^ ^]^ Nevertheless, this method can only be applied to extract ISF from the epidermis and other superficial regions of the skin.^[^ ^263^ ^]^ Pu et al. developed CGM devices featuring three electrodes integrated into a microfluidic chip.^[^ ^264^ ^]^ Ultrasound was applied to enhance skin permeability, and vacuum pressure was employed to extract up to 1 µL of ISF. The working electrode, coated with a composite of graphene and gold nanoparticles, was activated to detect glucose levels. The inclusion of this nanocomposite structure greatly enhanced the sensitivity of the device.

### 8.4 Microdialysis‐Based Methods of ISF Extraction

Microdialysis is a well‐established invasive technique for long‐term continuous sampling of ISF, especially for monitoring glucose levels.^[^ ^265^ ^]^ This method involves the use of an implantable probe, typically constructed from a hollow semipermeable fiber.^[^ ^266^ ^]^ A solution (termed perfusate solution) is circulated at a low flow rate of 0.5–5 µL min^−1^ to generate a concentration gradient within the dermal layer. Microdialysis relies on the passive diffusion of analytes across a semipermeable membrane, allowing for molecular weight analysis without altering protein concentrations.^[^ ^267^ ^]^ However, maintaining probe sensitivity requires fine adjustments and regular calibration. The procedure is intricate and often involves using external pumps to provide the necessary flow of the perfusate solution.

### 8.5 Use of Microneedle‐based Devices for Direct ISF Probing and Analysis

**Figure 23:**
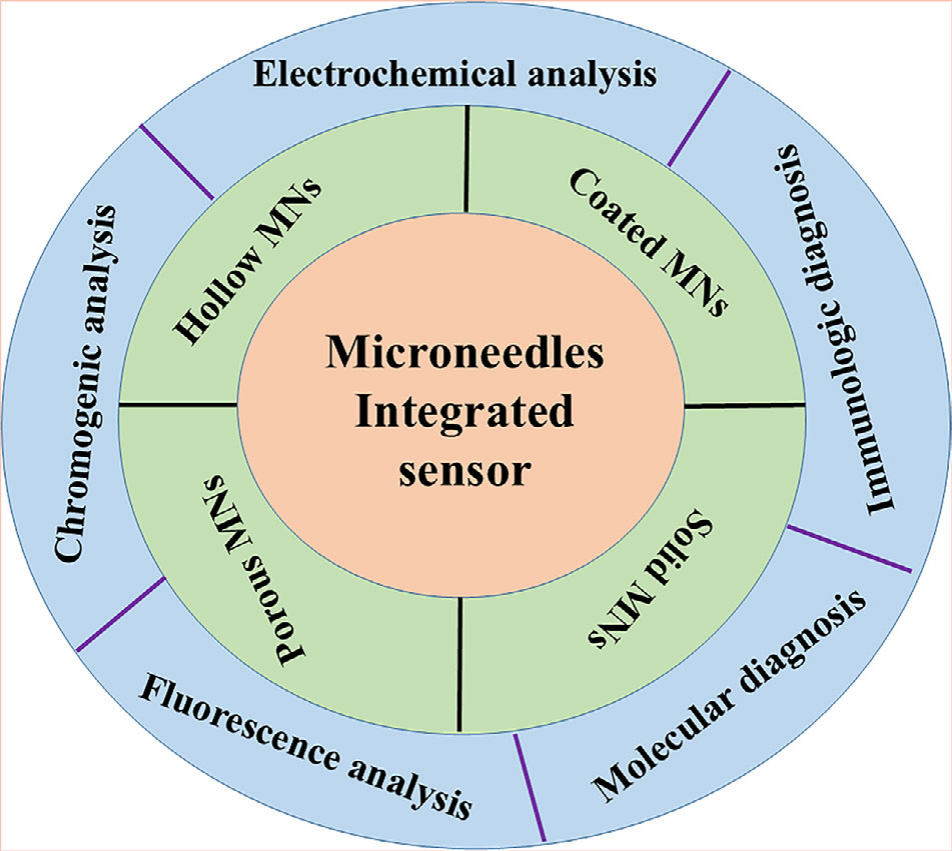
Schematic representation of a typical microneedle (MN)‐based sensor designed to extract skin interstitial fluids and conduct metabolic analysis. Reproduced with permission.^[^ ^273^ ^]^ Copyright 2023 MDPI.

Microneedles ranging in length from 300 to 1500 µm can penetrate the dermal layer of skin to allow the extraction of ISF. Microneedles of different designs, including hollow, porous, solid, and hydrogel‐based microneedles, have been employed for efficient ISF extraction, as shown in **Figure** **23**. Several aspects need to be considered when microneedles are employed for ISF extraction; these include the geometry of the microneedles (length, diameter, size, and shape), the selection of biocompatible materials, and the array's configuration. Microneedles can be employed as probes for biosensing applications and for fluid collection.^[^ ^268^ ^]^ Solid microneedles are usually employed in conjunction with electrodes or biosensors to detect ISF. These electrodes are fabricated using conductive materials such as gold, platinum, or silver, while the biosensing layer relies on enzymatic reactions to identify biomarkers such as glucose, proteins, and ions. By contrast, hollow and porous microneedles rely on capillary action for ISF extraction; however, the fabrication of hollow microneedles is more complex and expensive compared to that of the solid and porous types. Porous or hollow microneedles can be filled with solutions to increase ionic strength, creating an osmotic pressure gradient that drives the flow of ISF through the micropores for as long as the gradient persists.

Wang et al. utilized solid glass microneedles of length 700–1500 µm for achieving skin penetration.^[^ ^269^ ^]^ ISF was eliminated from the newly generated pores using vacuum pressure, enabling the collection of 1–10 µL of ISF within 2–10 min to evaluate glucose levels. Mukerjee et al. were the first to employ hollow microneedles (length 250–350 µm) fabricated from single‐crystal silicon as well as micromachining techniques for ISF extraction.^[^ ^270^ ^]^ The collected ISF was directed to the back of the microfluidic system, where a commercially available glucose strip was employed for measuring glucose levels. However, the diffusion of ISF through hollow channels to reach the sensor compartments caused a measurement delay, affecting the response time.^[^ ^271^ ^]^

Porous microneedles can be designed with integrated sensors for immediate analysis of ISF or for merely obtaining ISF samples via diffusion. Hydrogel‐based microneedles typically dissolve within the skin, allowing for drug diffusion upon contact with the ISF. However, this method can be slow, and the recovery of target biomarkers from the ISF often necessitates bulky equipment. This challenge arises from the ability of the hydrogel to retain a considerable amount of water within its structure. A recent study by Sheidaei et al. revealed that hydrogel‐based microneedles fabricated from a blend of polyvinyl alcohol and chitosan (PVA/CS) patches can mitigate these drawbacks.^[^ ^272^ ^]^ The PVA/CS composite microneedles exhibit rigidity when dry due to their phase transition characteristics, enabling easy skin penetration. Additionally, the thermal degradation behavior of PVA facilitates the straightforward recovery of target biomarkers from the microneedle arrays.

## 9 Types of Microneedles and Working Principle

Microneedle‐based sampling techniques employ arrays of microneedles to achieve skin penetration and create channels for the extraction of ISF. Depending on their composition and morphology, microneedles can be categorized as solid, hollow, porous, or hydrogel‐based.

### 9.1 Solid Microneedles

**Figure 24:**
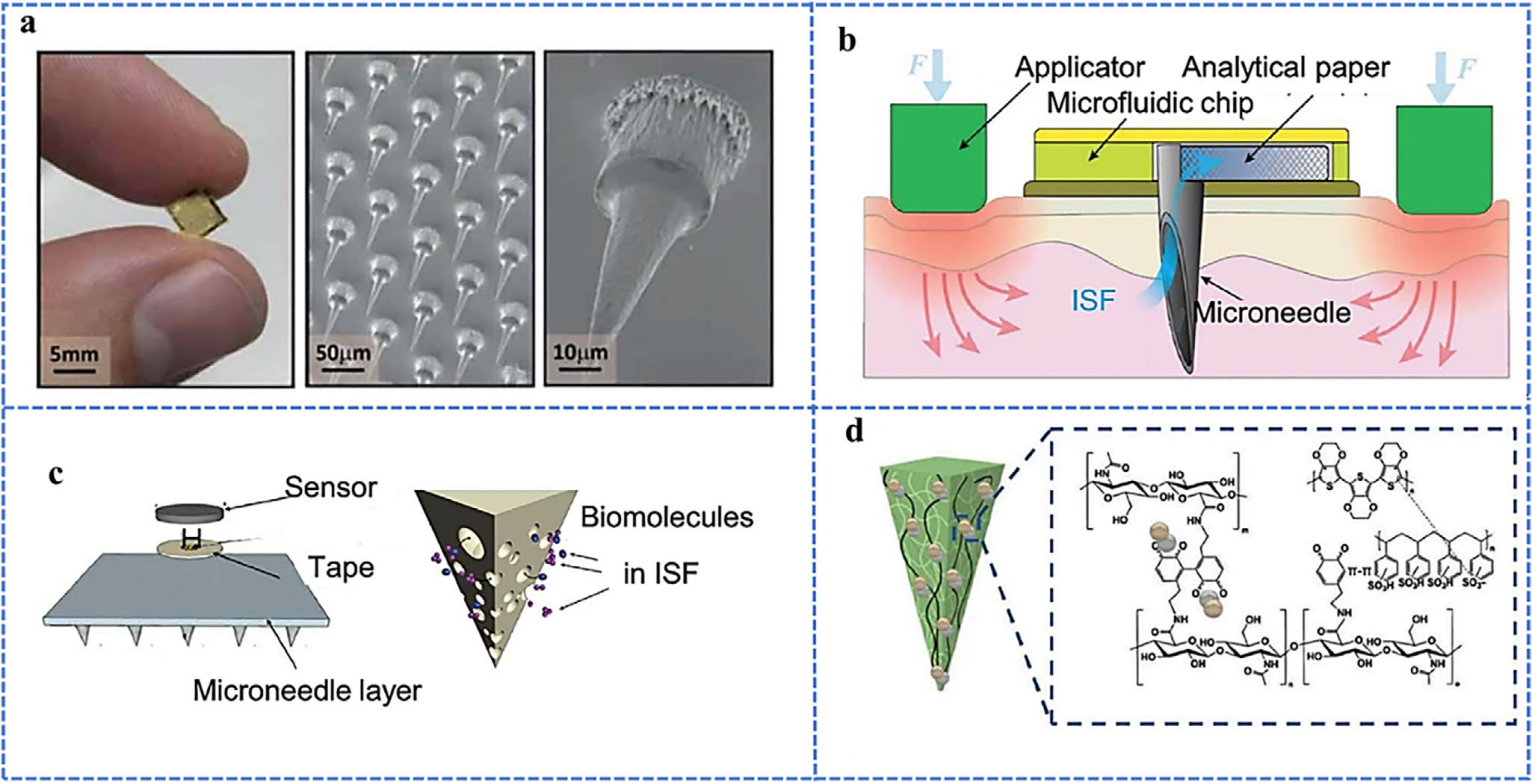
ISF sampling techniques. a) Functionalized solid microneedle patch for in vivo detection of biomarkers. b) Hollow microneedle patch for ISF collection that enables the detection of multiple analytes. c) Paper‐based porous microneedle patch for prediabetes screening test. d) Conductive hydrogel‐based microneedle array for real‐time monitoring of glucose levels. Reproduced with permission.^[^ ^43^ ^]^ Copyright 2024 Nature.

Solid microneedles are usually fabricated in the shape of a pyramid or cone with sharp edges. They are easier to fabricate, provide greater mechanical strength, and are more economical compared to the other types of microneedles.^[^ ^274^ ^]^ The application and subsequent withdrawal of microneedles on the skin leads to the formation of microchannels that allow for ISF sampling or extraction. The flow of ISF into these channels depends on osmotic pressure and is driven by a concentration gradient. Sensors positioned near the microchannels can directly analyze the components of ISF.^[^ ^275^ ^]^ Negative pressure–driven convection, which generates a pressure difference between the dermal layer and the skin surface, can also be employed to enhance the flow of ISF into the microchannels formed by microneedles for further analysis. Additionally, RI has been shown to aid the flow of ISF into the micropores for subsequent examination.^[^ ^42^ ^]^ Furthermore, solid microneedles fabricated to contain functional materials can be inserted into the dermal layer for in situ detection of biomarkers.^[^ ^247^ ^]^ In this case, the edges of the microneedle are coated with electrode materials and then functionalized with sensor elements to allow the detection of specific biomarkers and conversion of the generated signals for further analysis. As these microneedles directly function as sensors, they can be easily integrated with other electronic devices and data collection systems to monitor the levels of biomarkers. Corrie et al. were among the first to introduce the idea of functionalized microneedle patches for the detection of specific biomarkers, as shown in **Figure** **24**a.^[^ ^276^ ^]^ This method has been designed to identify clinically relevant biomarkers such as glucose, drugs, and numerous other biomarkers.^[^ ^247^, ^277^, ^278^ ^]^ While it allows for the selection of target biomarkers via the alteration of microneedles, an extra step generally needs to be included to elute the captured biomarkers from the microneedles for subsequent in vitro analysis.

### 9.2 Hollow Microneedles

Solid and hollow microneedles are usually fabricated from the same materials and have similar morphological characteristics. The hollow core of these microneedles creates microchannels for the extraction of ISF; these microchannels also serve as a pathway for drug delivery. This technique employs capillary action to collect ISF from the dermis, generating rapid convection within the capillaries and creating a concentration gradient that facilitates the extraction of ISF from the dermal layer for further analysis.^[^ ^279^ ^]^ The integration of a microneedle patch compatible with clinical translation was proposed by Ribet et al. for extracting ISF, as shown in Figure 24b.^[^ ^280^ ^]^ This method allows for the simultaneous monitoring of various analytes, including small molecules, antibodies, and proteins. The incorporation of functional materials into the lumen allows for the precise identification of biomarkers within the dermis. As an example, Gout et al. developed a microneedle array containing microneedles coated with different carbon pastes, facilitating both non‐enzymatic and enzymatic detection of levodopa.^[^ ^281^ ^]^ The development of sensing systems that employ hollow microneedles has been the focus of several studies.^[^ ^282^, ^283^ ^]^ However, a major challenge with the use of hollow microneedles is their tendency to get clogged, given that the insertion procedure involves severing dermal tissue.^[^ ^284^ ^]^

### 9.3 Porous Microneedles

Porous microneedles are distinguishable by an abundance of capillary channels and are commonly fabricated from materials such as polymers, metals, and inorganic compounds.^[^ ^285^ ^]^ In conjunction with iontophoresis‐based systems, these microneedles can facilitate the extraction of ISF from the dermis to the sensing chamber of the device for further analysis. As an example, Kusama et al. combined iontophoresis with ion‐conductive porous microneedles enhanced with charged hydrogels to improve ISF extraction efficiency significantly.^[^ ^286^ ^]^ Besides their utilization in conjunction with iontophoresis, the porous microneedles can facilitate the rapid extraction of ISF via capillary‐driven channels for diagnostic purposes. For instance, Lee et al. developed a system that combined porous microneedles with a glucose sensor; the system performed exceedingly well for sample extraction as well as glucose measurement, as illustrated in Figure 24c.^[^ ^287^ ^]^ Moreover, the microporous shape of porous microneedle electrodes offers a higher specific surface area, which, in turn, provides a greater number of active sites for the detection of targets.^[^ ^288^ ^]^

### 9.4 Hydrogel‐Based Microneedles

Hydrogel‐based microneedles represent an innovative type of microneedles that remain stiff in dry environments but swell upon penetrating the dermis. These microneedles can be fabricated entirely from hydrogel or merely coated with it.^[^ ^289^, ^290^, ^291^ ^]^ Hydrogel‐based microneedles present several distinct advantages compared to conventional microneedles. First, the stiffness of hydrogel‐based microneedles can be easily controlled by adjusting the polymer crosslinking ratio, which is more challenging than conventional microneedles.^[^ ^292^ ^]^ Second, the hydrogel‐based microneedles absorb greater quantities of ISF from the skin due to their swelling properties, leading to a larger loading capacity concomitant with a notable increase in volume during penetration.^[^ ^289^ ^]^ Hydrogel‐based microneedles are particularly conducive to the integration of functional materials that impart specialized capabilities. For example, Nejad et al. developed a CGM system utilizing hydrogel‐based microneedles embedded with conductive polymers and nanoparticles, which generate conductive networks that serve as working electrodes (Figure 24d).^[^ ^289^ ^]^ Mandal et al. described a hydrogel‐based microneedle system embedded with adjuvants and antigens for tracking skin‐resident immune responses. Additionally, the incorporation of osmolytes into hydrogel‐based microneedles has been shown to increase their collection efficiency by promoting osmosis‐driven flow.^[^ ^293^ ^]^ Moreover, hydrogel‐based microneedles can act as reservoirs, although further steps are required to retrieve the collected ISF from the microneedles.^[^ ^294^ ^]^

## 10 Integration of Microfluidics with Microneedle‐Based Arrays for Simultaneous Sampling and Sensing Applications

The integration of a microneedle array patch with a microfluidic system is an effective approach for wearable sensing, medical, and agricultural applications.^[^ ^295^, ^296^, ^297^ ^]^ This method allows for noninvasive extraction of fluids from the skin in conjunction with an appropriate pushing force.^[^ ^298^ ^]^ However, consistent and accurate ISF measurements necessitate addressing challenges such as ensuring analysis with low volumes of the collected sample, minimizing sample dilution, and preventing contamination during the process of collection and analysis.^[^ ^299^, ^300^ ^]^ Electrochemical sensing can be integrated with a microfluidic chip by directing fluids through the microchannel using a microneedle array.^[^ ^301^ ^]^ However, various factors influence the efficiency of the microneedle array, including the material used for fabrication as well as the height, tip radius, diameter, shape, and density of the array.^[^ ^302^ ^]^ Additionally, mechanical and biological optimizations are essential for ensuring the effective functioning of the developed microneedles on human skin.

Takeuchi et al. illustrated the extraction of ISF using porous microneedles fabricated using hyaluronic acid (HA) coated PDMS. The array consisted of 21 microneedles, each measuring 1000 µm in length.^[^ ^268^ ^]^ The microneedle array exhibited the ability to penetrate an agarose gel‐based phantom and allow the extraction of ISF for further analysis. A continuous flow of ISF was established within the device via capillary action, which was enhanced by manual compression. A detection of changes in glucose concentration required a minimum flow rate of 1–2 µL min^−1^. Samant et al. successfully collected more than 1 µL of ISF from pig cadaver skin and living human subjects within a duration of 20 min using microneedle patches. The patches contained solid, porous, and hollow microneedle arrays, which penetrated the outer layers of skin. The study indicated that hydrogel‐based porous microneedles supported efficient ISF extraction via diffusion, whereas capillary or osmotic driving forces enhanced the extraction efficiency of hollow microneedles.

**Figure 25:**
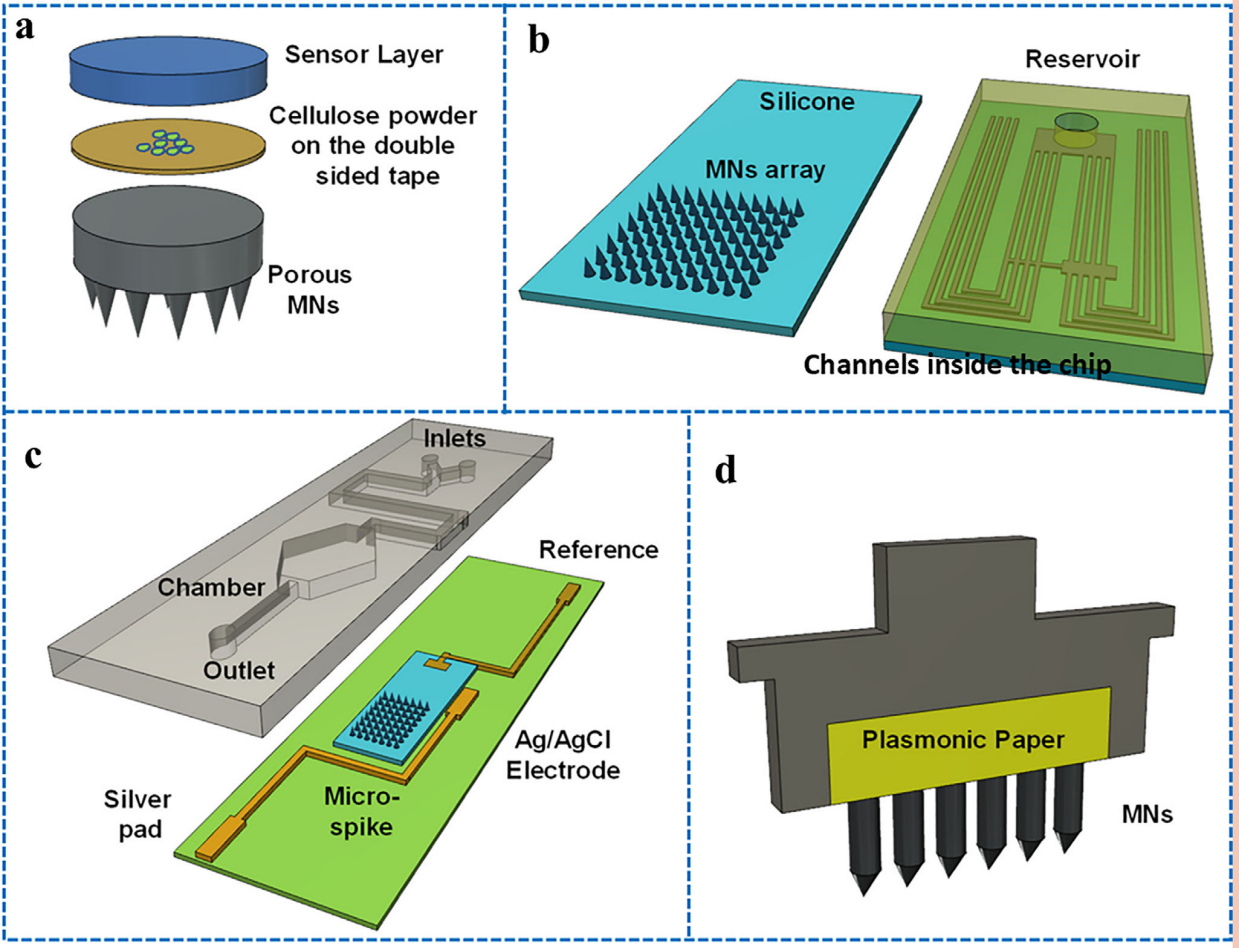
Integration of microneedle arrays with the microfluidic system. a) A porous microneedle array fabricated from PDMS, connected to paper‐ and colorimetry‐based glucose sensors. b) A hollow microneedle array connected to a set of microchannels with a common reservoir. c) A solid microneedle array (microspike) coated with an enzyme layer and connected to a microfluidic system for further investigations of substrates of clinical relevance in ISF. d) A microneedle array attached to plasmonic paper. The ISF absorbed onto the paper is further investigated using SERS. Reproduced with permission.^[^ ^308^ ^]^ Copyright 2021 MDPI.

Ribet et al. described a sampling system consisting of a single stainless‐steel microneedle that allowed the successful collection of ISF (1 µL) from the human forearm.^[^ ^303^ ^]^ The microneedles were integrated with a microfluidic chip and a paper matrix to store the collected biofluid. Off‐chip analysis of the collected biofluid was conducted using LC‐MS/MS. Lee et al. designed a porous microneedle array fabricated from PDMS using mold‐casting and salt‐leaching techniques, as shown in **Figure** **25**a. A microneedle array consisting of 21 needles measuring 800 µm in length was incorporated into a paper‐ and colorimetry‐based enzymatic glucose sensor via a microfluidic channel. The fluid was drawn from an agarose gel, and the color of the filter paper varied according to the concentration of glucose in the sample.^[^ ^304^ ^]^

Mukerjee et al. reported the development of a hollow microneedle array, with the heights of microneedles ranging from 250 to 350 µm; this was connected to a microchannel system (Figure 25b), which featured a shared reservoir for the collection of ISF. The microneedles effectively penetrated the epithelial layer containing live cells to access ISF, with capillary action facilitating the fluid flow. Glucose levels in ISF were validated via in situ measurements.^[^ ^270^ ^]^

Trzebinski et al. designed a solid microneedle array, referred to as microspikes, to detect substrates of clinical relevance, such as glucose and lactose, in ISF, as shown in Figure 25c. This array was coated with an enzyme layer consisting of glucose oxidase or lactose oxidase over a gold layer, which enhanced the sensitivity of the device by increasing the signal‐to‐noise ratio. The microspike system was integrated with a microfluidic chip to monitor biomolecule movement.^[^ ^305^ ^]^

Kolluru et al. designed an array of microneedles to extract ISF from the dermal layer of rats.^[^ ^306^ ^]^ The microneedle array was affixed to a thin strip of filter paper, which absorbed the extracted ISF for subsequent analysis. Each fabricated microneedle of length 650 µm facilitated the successful extraction of more than 2 µL of ISF within a minute. However, multiple applications of the array were required to enhance ISF extraction, which poses challenges for human applications. This was addressed by subsequent improvements in the design achieved via the integration of plasmonic paper, as illustrated in Figure 25d.^[^ ^307^ ^]^ The filter paper was functionalized with polystyrene sulfonate (PSS)–coated gold nanorods. The ISF absorbed by the paper was analyzed using surface‐enhanced Raman spectroscopy (SERS). This method was successfully applied for quantifying and evaluating the pharmacokinetic profiles of rhodamine 6G from ISF extracted from the dermal layer of rats.

The detection of biomarkers is essential for the early diagnosis of life‐threatening diseases; however, the conventional methods of detection involve invasive, time‐consuming, and expensive blood tests. Heifler et al.^[^ ^56^ ^]^ highlighted the growing strain on medical facilities due to an aging population, modern lifestyle diseases, and pandemics. To address these challenges, the healthcare industry has shifted focus to the development of home‐based and POCT medical devices. Diabetes, as a chronic condition that poses serious mortality risks and socio‐economic consequences, is of considerable concern and necessitates stringent glucose regulation to prevent complications. However, the current glucose management systems are fraught with challenges such as the lack of sensor reliability and the unavailability of integrated systems. Moreover, a minimally invasive system for CGM that uses a chemically modified SiNW‐FET nanosensor array integrated with microneedle elements; this system enables the direct, uninterrupted, and highly specific measurement of ISF‐based metabolites, effectively avoiding interference from other substances. The platform is designed with a unique 2D–3D architecture fabricated on a single silicon‐on‐insulator (SOI) chip, which allows for selective sensing of multiple metabolites. The system was successfully employed for CGM in vivo trials with healthy subjects. Additionally, the integration of microinjection needles allowed for both glucose monitoring and insulin delivery in mice, highlighting the system's potential as a cost‐effective and multifunctional wearable device for real‐time tracking of biomarkers and synchronized drug delivery.

**Figure 26:**
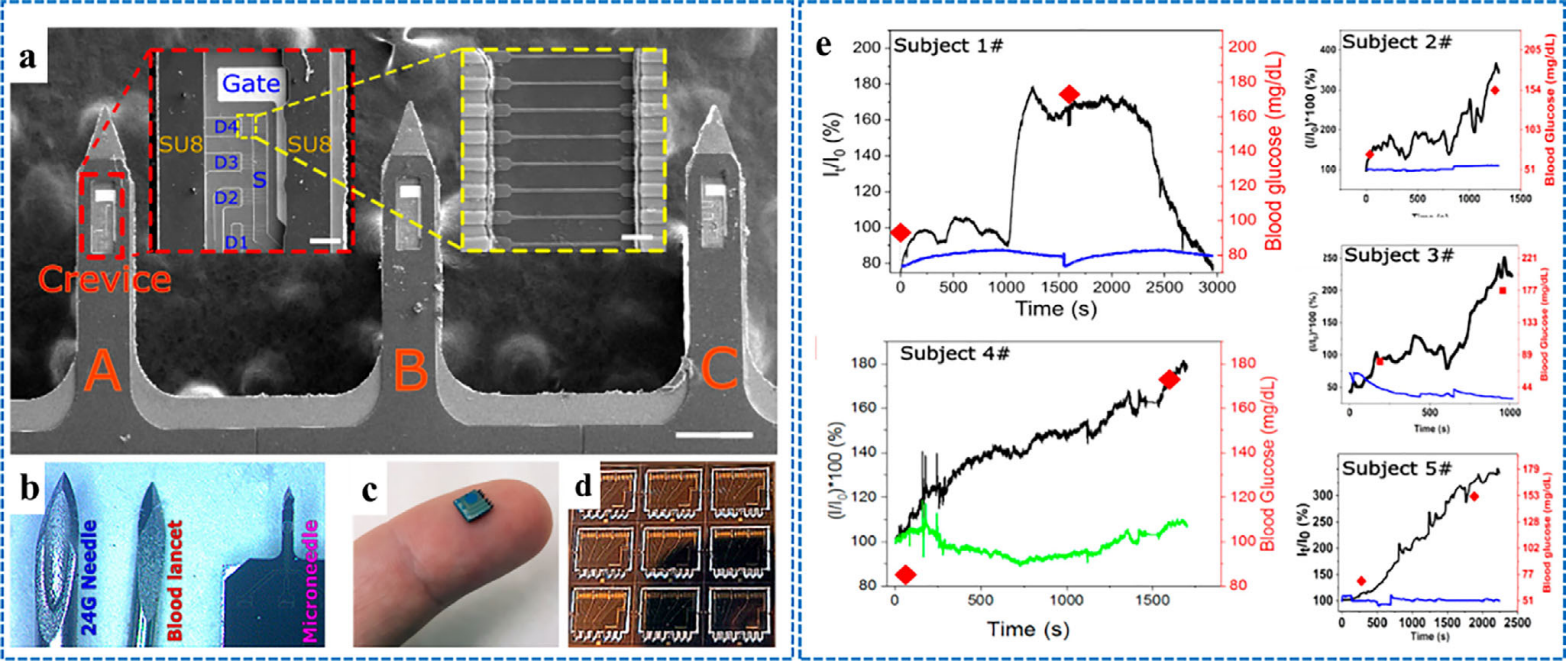
SiNW array‐integrated microneedle sensor platform for in vivo metabolite monitoring and oral glucose tolerance testing (OGTT). a) Fabricated microneedle with SiNW, b) Dimension comparison between our microneedle, commercially used injection needles, and blood lancet. c) The microneedle chip on a finger. d) Die batch image of a six‐microneedle architecture after full separation by micromachining. e) Sensor performance analysis. Reproduced with permission.^[^ ^56^ ^]^ Copyright 2021 American Chemical Society.

A robust vertical silicon microneedle was designed to incorporate an array of electrical nanosensors at its tip for continuous transdermal monitoring. As illustrated in **Figure** **26**a, the structure of the platform includes the following: a) a depth‐controlled, sharp silicon microneedle, b) a protective polymer layer with a crevice that exposes the sensing and gating regions, and c) an independent SiNW‐FET sensor array integrated into each microneedle. The detection fissure shielded by SU8, four SiNW‐FET devices (D1–4), a gold gate, and Ti/Pd source–drain connections for passivation are depicted in the inset with a red dashed border in Figure 26a. The SiNW array device, with eight nanowires of 125‐nm width and 50‐nm height between connections, is shown in the inset with a yellow dashed border. The Si/metal contact improvement is attributable to the larger area associated with the SiNWs. A multianalyte sensing device is made possible by employing three distinct needles. The redundant sensors of the platform enable self‐calibration via the use of enzyme‐free microneedle components; consequently, any changes arising from nonspecific reactions are considered a baseline component. As illustrated in Figure 26b,c, the fabricated microneedles are considerably smaller than the commercially available needles and lancets typically employed for insulin injections and blood sampling. This smaller size makes the microneedles minimally invasive and easier to insert into the skin compared to standard needles and lancets used in commercial applications. Additionally, batches with highly controllable and reproducible electrical properties can be produced (Figure 26d), which correlate with the number of wires fabricated and the SOI wafer properties. A comparison of the results obtained with CGM and the oral glucose tolerance test is featured in Figure 26e.

**Figure 27:**
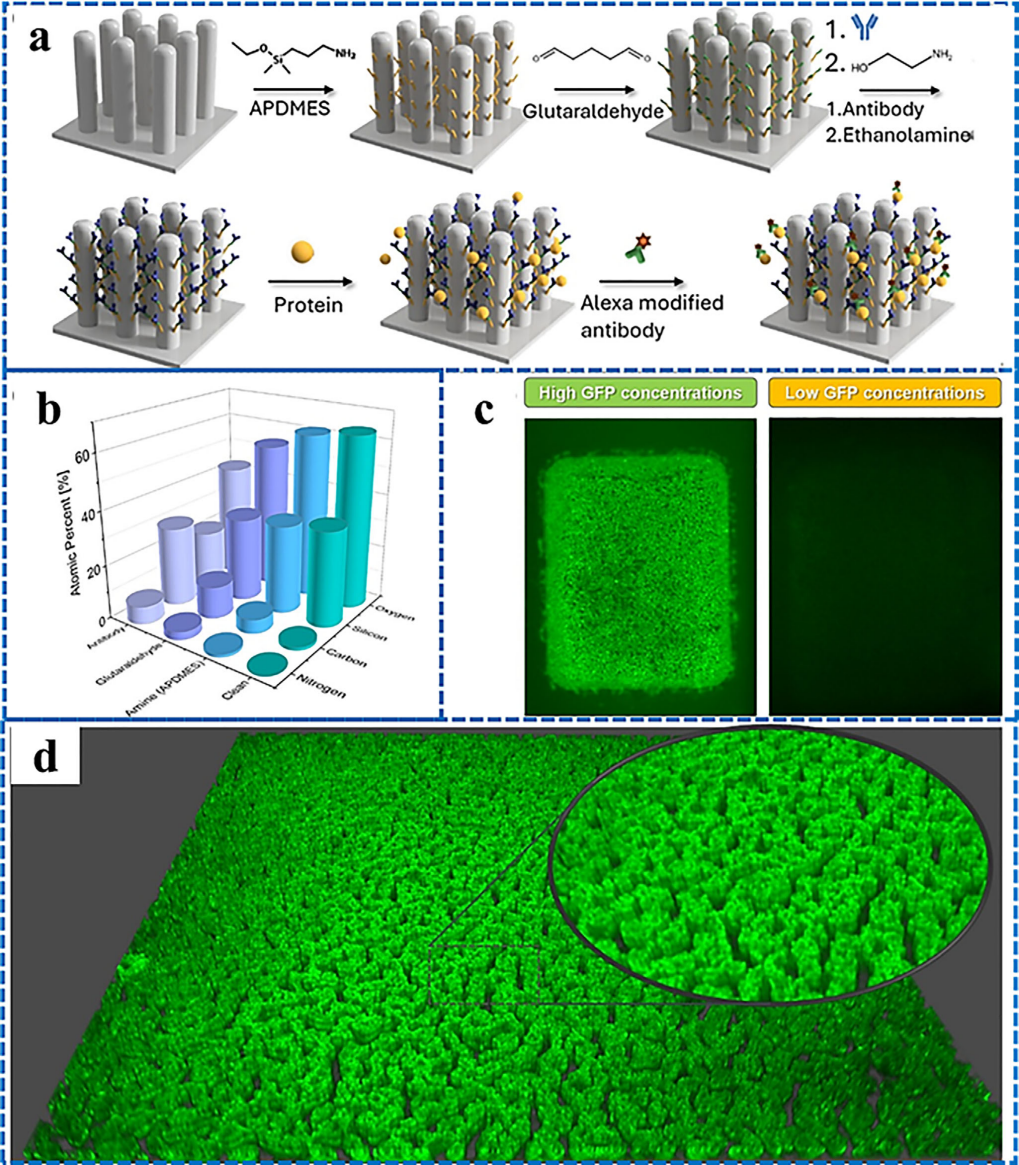
Surface modification process. a) Schematic illustration of the chemical modification process and fluorescent labeling. b) Results of XPS analysis at four different stages of surface modification. c) Fluorescence microscopy images showing concentration‐dependent fluorescence intensities resulting from the binding of GFP to the surface modified with anti‐GFP antibody. d) 3D‐Fluorescence microscopy imaging (obtained using Z‐stacking) of the nanopillars sensing area. The fluorescence resulting from GFP binding across the pillar's entire height (8 µm) is shown. Reproduced with permission.^[^ ^58^ ^]^ Copyright 2024 American Chemical Society.

Patolsky et al.^[^ ^58^ ^]^ developed a minimal‐invasive diagnostic platform featuring microneedles embedded with vertically‐aligned nanopillar arrays. This innovative platform allows for the direct optical measurement of biomarkers in capillary blood. It enables fast, highly sensitive, and multiplexed detection of protein biomarkers based on antibody–antigen interactions, thus eliminating the need for blood extraction. It exhibited remarkable sensitivity (low pM) and specificity in preliminary in vitro and in vivo experiments, highlighting its potential as an effective POCT solution. The results demonstrate that the sensing microneedles do not impact cell viability or the overall health of the experimental mice. Additionally, immuno‐inflammatory responses were not observed in the skin of mice following pricking. The excellent biocompatibility of the SiNP‐embedded microneedles highlights their strong potential for future clinical applications. The accurate and direct detection of bioanalytes from capillary blood relies on the interface between the sample and the sensing region of the SiNP array on the device. Given that most proteins in the human body are non‐fluorescent, the device's functionality was initially demonstrated by modifying the surface with anti‐green fluorescent protein (anti‐GFP) antibody (**Figure** **27**a). The chemical modification of the SiNP surface facilitates the transdermal monitoring of various biomarkers and proteins. Figure 27b shows the X‐ray photoelectron spectroscopy (XPS) analysis results of the fabricated SiNPs following modification. Figure 27c illustrates the fluorescence microscopy images obtained using different contrasts because of variations in the fluorescence intensity. Figure 27d presents a 3D image (obtained via Z‐stacking) illustrating the complete modification of the surface of the pillars, captured using a fluorescence microscope following the binding of GFP.

## 11 Diagnostic Applications of Microneedle‐Based ISF Biosensors

### 11.1 Immunodiagnostic Biosensors with Integrated Microneedles

Immunodiagnostic sensors integrated with microneedles are designed to detect humoral and cellular immune responses by employing microneedle‐based extraction in conjunction with immunological techniques for sample analysis.^[^ ^280^, ^309^ ^]^ These sensors can detect infections caused by pathogenic microorganisms, tumors, or autoimmune diseases. Typically, antigens or antibodies are employed for detecting the corresponding target antibody or antigen based on the specific antigen–antibody interaction. Different methods of signal enhancement, including fluorescence, chemiluminescence, and colorimetric chemical reactions, are often employed.^[^ ^310^, ^311^ ^]^ Currently, microneedles are primarily used for extracting or transferring ISF for clinical analysis. These microneedles have the potential to be converted into efficient immunodiagnostic sensors that allow in situ immune‐detection directly within local tissues.^[^ ^312^ ^]^

Various microneedles have been designed to exploit antigen–antibody binding to allow the identification of specific protein markers in skin‐derived ISF. These microneedles are extracted from the skin and incubated with enzyme‐labeled antibodies for a few minutes. This process is followed by the detection and quantification of target protein markers using fluorescence microscopy or colorimetric assays involving enzyme–substrate reactions. For instance, Zhang et al. introduced innovatively designed immunodiagnostic microneedles integrated with photonic crystal (PhC) barcodes; the probe‐modified PhC was enriched with specific antibodies for the targeted detection of markers.^[^ ^309^ ^]^

**Figure 28:**
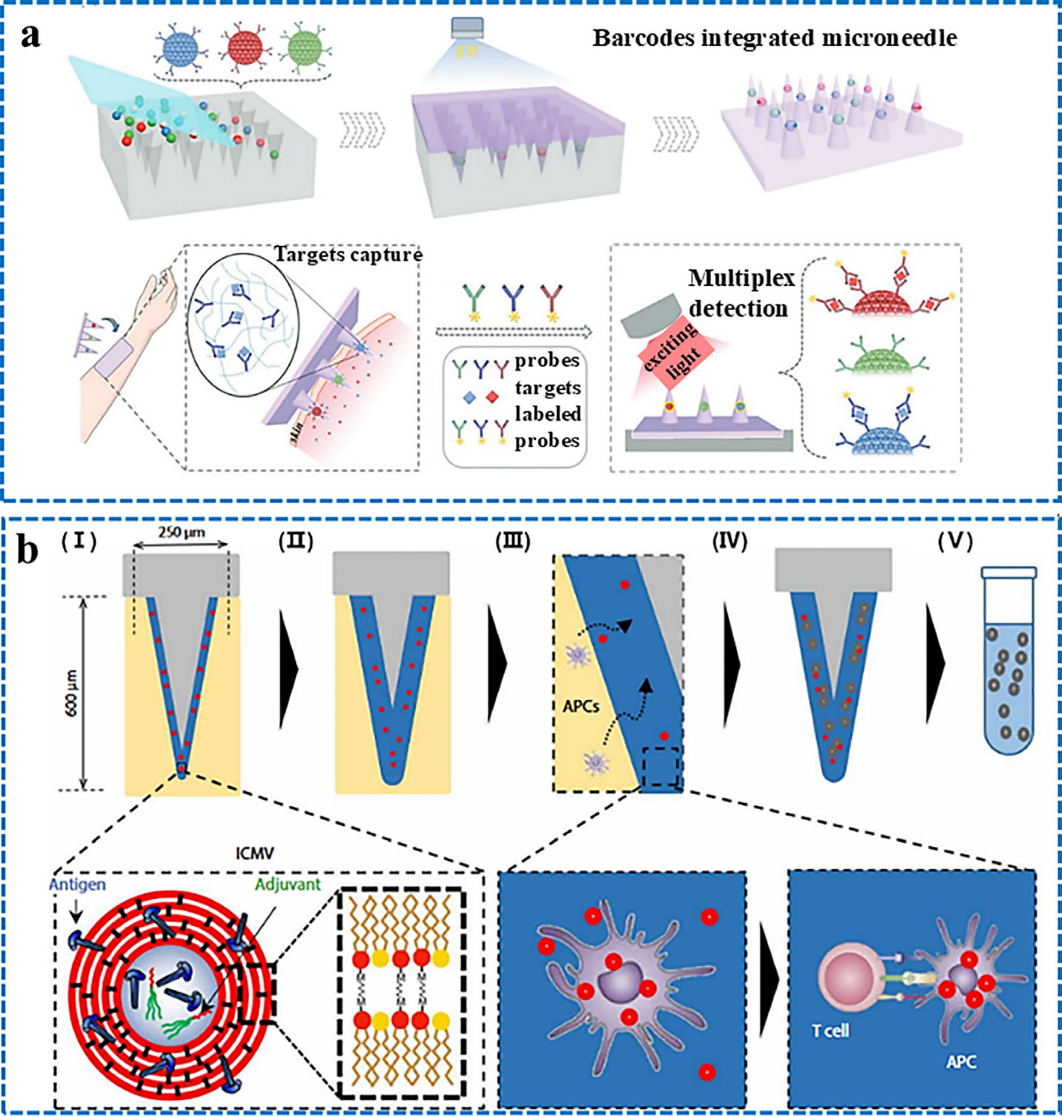
Construction of immunodiagnostic microneedle‐integrated sensors. a) Schematic illustration of the fabrication and application of immunodiagnostic microneedles for the detection of markers in ISF. b) Schematic representation of the structure and mechanism of APC‐enriching microneedles. I) Application of a microneedle coated with a layer of dried hydrogen onto the skin. II) Swelling of the hydrogel layer with ISF. III) Immune‐cell infiltration into the hydrogen layer. IV) Removal of the microneedle array from the skin. V) Removal of cross‐linked hydrogel layer and release of cells, as well as ISF for analysis. Reproduced with permission.^[^ ^273^ ^]^ Copyright 2023 MDPI.

The antigens can be easily identified by observing the reflective colors of the photonic crystal barcodes, with corresponding fluorescence intensities revealing the relative quantities of the target biomarkers. These microneedles facilitated the simultaneous detection of biomarkers in ISF (IL‐1β, TNF‐α, and IL‐6) in a mouse model of sepsis, as illustrated in **Figure** **28**. This microneedle device demonstrated that integrating various antigen–antibody combinations with microneedles holds significant potential for monitoring and screening disease markers, as shown in Figure 28a. Conventional blood draws frequently fail to support the effective monitoring of various types of cells in local tissues. The number of antigen‐specific T‐cell populations in the bloodstream is typically low, but it is of significant clinical importance, particularly during vaccination or infection. Consequently, the in situ detection of T‐cells using immunodiagnostic microneedles is crucial for assessing local immune responses. Mandal et al. developed a microneedle array that is loaded with adjuvant and specific antigens to attract antigen‐presenting cells from the skin, allowing for subsequent analysis of cell phenotypes and functions without altering the immune status of the surrounding tissues.^[^ ^291^ ^]^

### 11.2 Microneedle‐Integrated Biosensors for Molecular Diagnostics

Microneedle‐integrated sensors for molecular diagnostics present an innovative approach for the in situ detection, analysis, and diagnosis of genetic materials within tissues by applying microneedle technology in conjunction with molecular biology techniques.^[^ ^56^, ^313^ ^]^ These sensors can identify aberrant changes in molecules such as DNA and RNA, providing crucial evidence for the early diagnosis, prevention, and treatment of various diseases.

**Figure 29:**
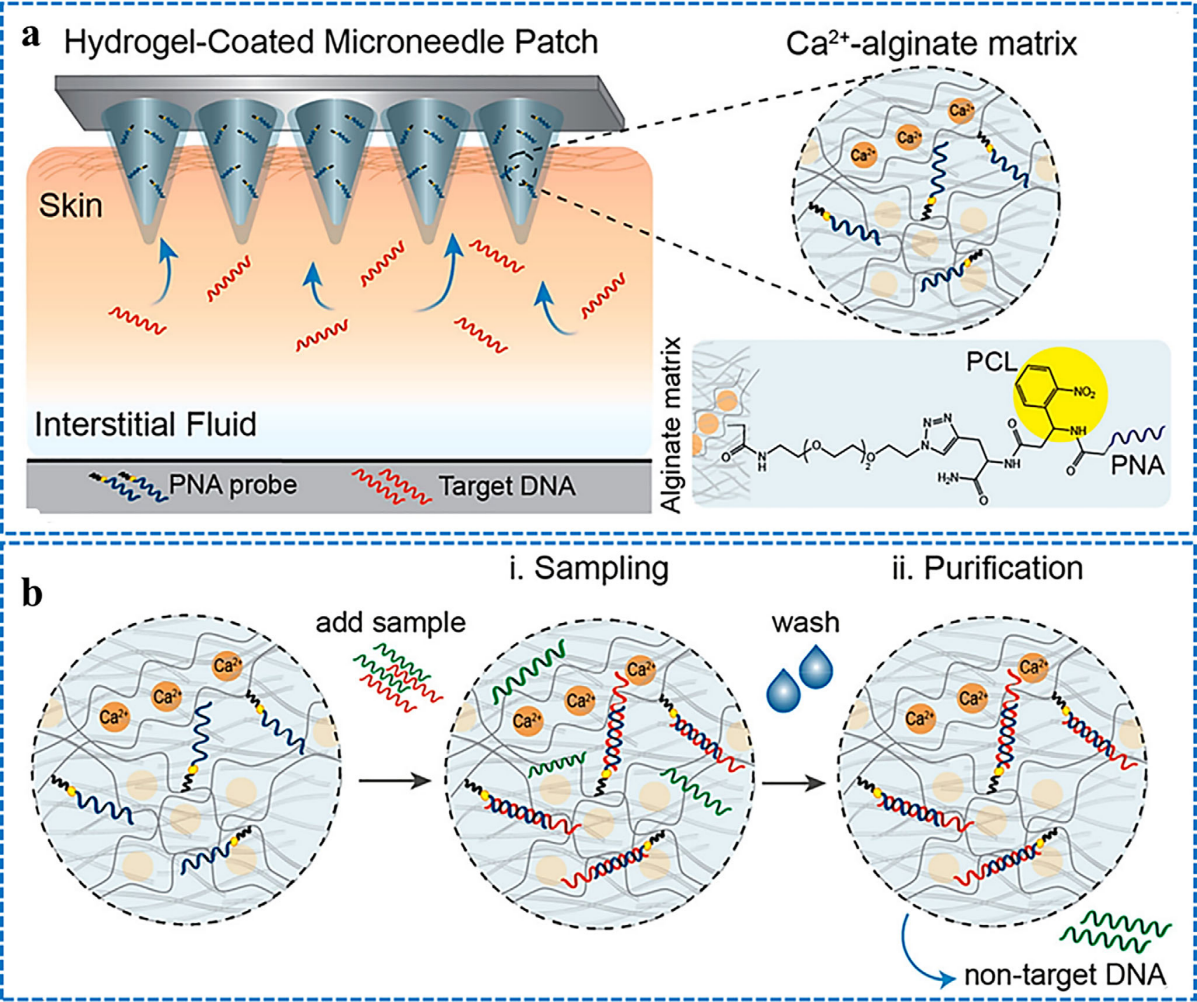
Construction of microneedle‐integrated sensors for molecular diagnostics. a) Schematic illustration of microneedles functionalized with SA‐PCL‐PNA coatings for detecting target DNA in ISF obtained from the skin. b) A generic protocol for the sampling and purification of target DNA and the removal of nontarget sequences. Circles represent a magnified view of the SA hydrogel coating on microneedle patches. Reproduced with permission.^[^ ^273^ ^]^ Copyright 2023 MDPI.

The in situ detection of microRNAs (miRNAs) in tissues has emerged as a promising approach for clinical monitoring and localized molecular diagnostics. For instance, Yang et al. developed a methacrylated HA (MeHA)–based microneedle patch integrated with an innovative DNA hydrogel technique, enabling the quick enrichment of miRNAs for a higher efficiency of detection.^[^ ^314^ ^]^ The accumulation of sufficient quantities of miRNA in the MeHA/DNA‐MNs patch resulted in the initiation of a series of DNA displacement reactions, which resulted in the generation of a detectable fluorescence signal. This mechanism of signal amplification, characterized by cascade endpoint–mediated DNA displacement, enables the monitoring of miRNAs at concentrations as low as 241.56 pM, reflecting the high sensitivity of the approach. In another example, Sulaiman et al. developed microneedle arrays coated with sodium alginate (SA)‐peptide nucleic acid (PNA) hydrogel, facilitating the simultaneous in situ detection of specific miRNA markers in ISF collected from the skin, as illustrated in **Figure** **29**a.^[^ ^315^ ^]^ Compared to conventional oligonucleotides, the PNAs exhibit enhanced affinity and specificity during hybridization with complementary DNA or RNA sequences. As illustrated in Figure 29b, the application of the microneedle patch for extracting DNA or RNA from a solution facilitates the selective hybridization of the PNA probe with target sequences and allows for the removal of nonspecific molecules from the hydrogel matrix via washing. These microneedles demonstrate rapid sampling kinetics and a high capacity of 3.25 µL/min, facilitating the simultaneous detection and quantification of target nucleic acids within the microneedle or via the use of a light‐activated hydrogel solution. Furthermore, Yang et al. proposed a microneedle patch that boasts of graphene biointerfaces triggered by clustered regularly interspaced short palindromic repeats (CRISPR) and their associated protein (Cas‐9) for continuous in vivo monitoring of DNA.^[^ ^316^ ^]^

The wearable equipment described above facilitated instant monitoring of the Epstein–Barr virus and cell‐free DNA associated with kidney transplantation and demonstrated impressive stability for 10 days under in vivo conditions. This microneedle‐integrated molecular diagnostic sensor shows significant promise for continuous, long‐term tracking of cell‐free DNA and may serve as a valuable tool for the early detection of diseases and postoperative monitoring.

The commonly employed ISF‐based sensors and their applications are presented in **Table** **5**. Minimally invasive approaches, such as wearable ISF‐based sensors, have been successfully employed to detect biologically significant markers. The strong correlation between analytes in blood and ISF suggests that ISF can be employed to measure the levels of more complex analytes in future applications. However, the development of noninvasive biosensors for monitoring critical analytes in ISF remains challenging, given that the current methods of ISF extraction using RI remain limited by low flow rates and a lack of uniformity. These challenges are expected to be addressed in the future. With advancements in existing techniques, wearable ISF‐based sensors are likely to witness broader applications in the future.

**Table 5:** Commonly employed ISF‐based sensors and their applications.

S.No	Biofluid	Platform	Biosensor	Analyte	Limit of detection	Reference
1	ISF	MN	Electrochemical	β‐hydroxybutyrate	Lac: 1‐–10 mM HB: 0.0‐–1.0 mM Glucose:1‐–10 mM	[317]
2	ISF	MN	Electrochemical	Lactate and glucose	0.1–‐10 mM	[318]
3	ISF	MN	Electrochemical	Alcohol	0–‐80 mM	[319]
4	ISF	MN	Field‐effect transistor	Sodium	2.78 × 10^−6^ M	[320]
5	ISF	Tattoo	Electrochemical	Glucose (in ISF)	Glucose: 0‐–160 µM	[18]
6	ISF	Tattoo	Electrochemical	Glucose (in ISF)	0.06 µM	[321]
7	ISF	MN	SERS	H_2_O_2_	1 µM	[119]
8	ISF	MN	Electrochemical	Catecholamine	100 nM	[322]
9	ISF	MN	Electrochemical	Levodopa	100 nM	[323]
10	ISF	MN	Optical	miRNA	miRNA‐141: 14 pM miRNA‐155: 6 pM	[124]
11	ISF	Microelectrodes	Electrochemical	Glucose	0–‐32 mM	[324]
12	ISF	MN patch	Electrochemical	Glucose	3–‐18 mM	[125]
13	ISF	MN	Electrochemical	Apomorphine	0.6‐–0.75 µM	[143]
14	ISF	MN array	Electrochemical	Glucose, lactate, and alcohol	Glucose: 0‐–4 0 mM Lactate: 0‐–28 mM Alcohol: 0‐–100 mM	[146]
15	ISF	High‐density polymeric MN array–based (PMNA) sensing patch	Electrochemical	pH	‐	[123]

MN, Microneedle; SERS, Surface‐Enhanced Raman Spectroscopy

### 11.3 Biomarker Stability in ISF During Extraction and Storage

The stability of biomarkers within ISF is crucial for ensuring analytical accuracy and clinical utility. This stability is significantly influenced by both the extraction methodology and the subsequent storage protocols. ISF comprises a wide variety of biomolecules, including metabolites, electrolytes, peptides, proteins, and nucleic acids, each with unique susceptibilities to enzymatic degradation, oxidation, adsorption to collection surfaces, and thermal instability. During the extraction process, physical and chemical stresses, such as shear forces, exposure to ambient temperatures, and prolonged contact with endogenous enzymes, can accelerate degradation. This is particularly true for sensitive targets like cytokines or low‐abundance peptides. To minimize these effects, it is essential to use rapid and minimally invasive collection methods, such as optimized minimal invasive microneedle arrays with low volume channels, and to isolate the sample from the environment immediately. In some cases, incorporating stabilizing additives, such as protease or RNase inhibitors, can help preserve biomarker integrity. Post‐collection storage is equally important for maintaining the fidelity of the biomarkers. Immediate cooling to 4°C for short‐term handling is recommended, followed by snap‐freezing in liquid nitrogen for long‐term storage at −80°C.^[^ ^59^ ^]^ These measures help suppress enzymatic and oxidative degradation. For highly unstable analytes, aliquoting samples to avoid repeated freeze‐thaw cycles and using inert, low‐binding storage vials can further enhance stability.^[^ ^325^ ^]^ Additionally, the stability of biomarkers is analyte‐specific; small metabolites and electrolytes often remain stable for long periods under refrigeration, while proteins, nucleic acids, and volatile compounds require stricter controls over temperature and pH.^[^ ^273^, ^326^ ^]^

Given this variability, implementing standardized pre‐analytical protocols that clearly define extraction duration, environmental conditions, stabilization strategies, and storage timelines is essential for ensuring reproducibility across studies and laboratories. Such rigorous control over both extraction and storage stages allows ISF‐based assays to produce clinically reliable and comparable results, addressing one of the key challenges in minimally invasive diagnostics.

## 12 ISF‐Based Wearable Biosensors for Minimal‐Invasive Biomedical Applications

ISF holds promise for minimally invasive healthcare applications owing to the strong correlation between biomarker levels in ISF and blood. Wearable ISF‐based biosensors enable real‐time, continuous monitoring of critical health parameters without the need for frequent blood sampling. ISF contains important biomarkers such as glucose, lactate, and electrolytes, and monitoring their levels is essential for managing conditions such as diabetes, dehydration, and metabolic disorders. These sensors typically employ microneedles or RI to painlessly access ISF, ensuring patient comfort.

### 12.1 Monitoring of Glucose Levels

**Figure 30:**
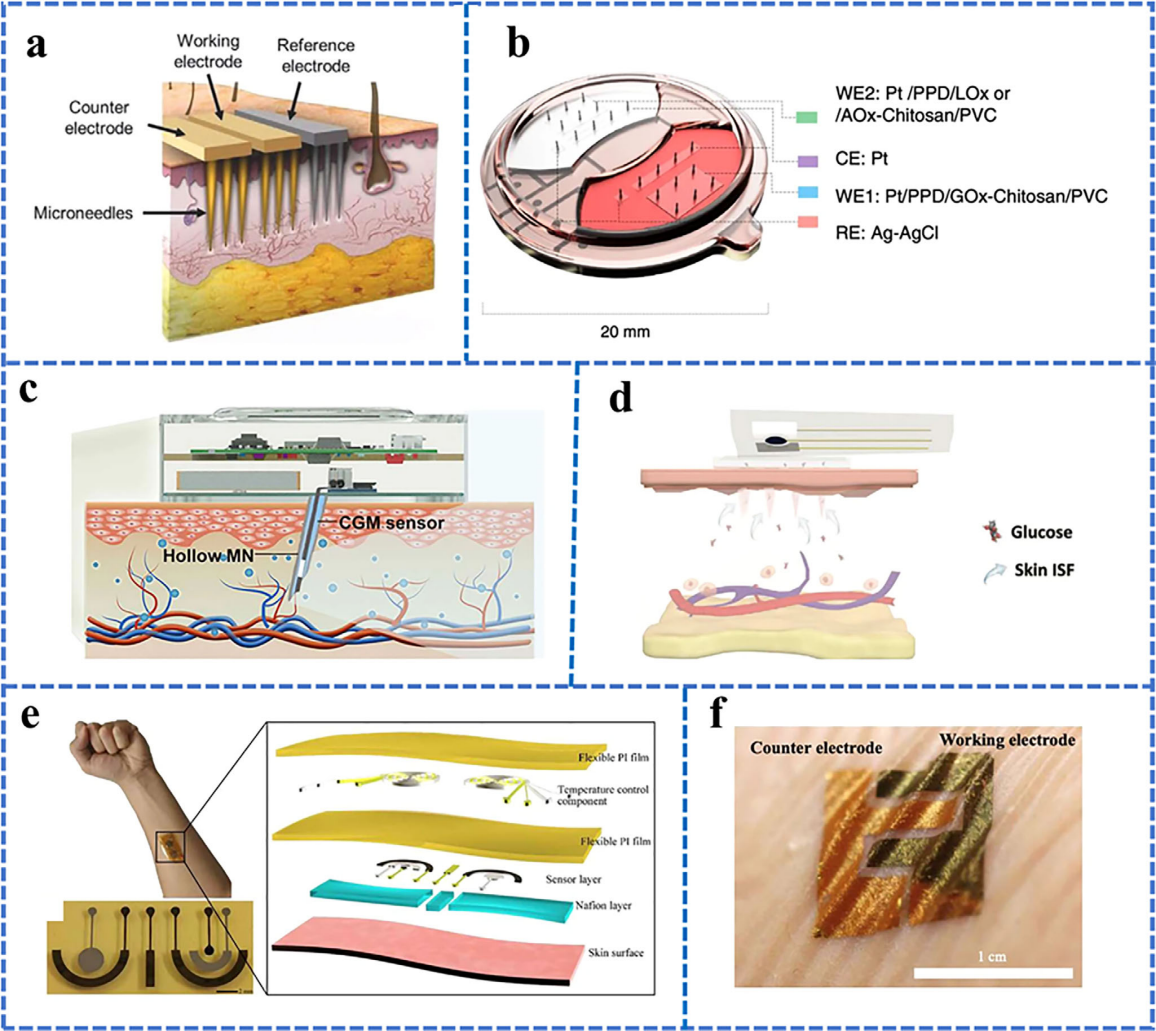
ISF‐based glucose sensors. a) Illustration of a high‐density microneedle‐based glucose sensor. b) Illustration of PMMA‐based electrochemical sensors with integrated microneedle systems. c) Schematic illustration of a PET‐based sensor for the continuous monitoring of glucose levels for diabetes management. d) Schematic illustration of a swellable microneedle‐based glucose sensor. e,f) Optical and layer‐by‐layer illustration of the RI‐based glucose sensor patch. Reproduced with permission.^[^ ^43^ ^]^ Copyright 2024 Nature.

The use of various ISF sampling techniques in combination with different sensors has led to the development of multiple platforms capable of real‐time monitoring of biomolecules. Monitoring the levels of glucose has received considerable attention by the healthcare community, as it can aid in the management of diabetes, one of the most common chronic conditions, and the implementation of measures to prevent disease progression. Various RI‐ and microneedle‐based platforms have hitherto been developed for assessing glucose levels in a minimally invasive manner. Many of these systems employ glucose oxidase–based amperometry, where changes in glucose concentration correlate with fluctuations in the produced current. For example, Dervisevic et al. introduced a high‐density silicon microneedle array that effectively detected increasing glucose levels in mice, demonstrating its capability to penetrate the skin barrier and facilitate the analysis of ISF (**Figure** **30**a).^[^ ^327^ ^]^ Additionally, Tehrani et al. developed a wearable device the size of a coin, which combined PMMA‐based sensors for glucose, lactate, and alcohol with units for signal capture and wireless transmission, as illustrated in Figure 30b.^[^ ^247^ ^]^ Pilot studies in humans have demonstrated that the sensors can effectively monitor glucose level fluctuations, including those triggered by the consumption of metals and snacks, for over 6 h. The results closely align with those of the standard finger‐prick test, achieving a mean absolute relative difference of 8.83%. Yang et al. employed similar techniques for developing a glucose sensor using PET. This sensor features hollow microneedles that allow easy skin insertion, as shown in Figure 30c.^[^ ^280^ ^]^ The complete equipment includes a wireless transmission module, which provides a viable alternative to established CGM systems such as Freestyle Libre from Abbott. Additionally, swellable microneedles facilitate the transport of sampled ISF to the sensing chamber for subsequent electrochemical analysis, as shown in Figure 30d.^[^ ^286^, ^293^ ^]^ Moreover, the incorporation of glucose‐responsive materials during the fabrication of needle tips allows for the detection of colorimetric, chemiluminescent, or fluorescent signals for the semi‐quantitative assessment of glucose levels. These techniques can also be adapted for the detection of other small molecules, including uric acid and vitamin C.^[^ ^328^, ^329^, ^330^ ^]^

Although microneedles provide an effective and minimally invasive system for the transdermal monitoring of ISF, achieving consistent penetration depth and maintaining sensor stability continues to present challenges. By contrast, RI‐based patch sensors utilize a planar arrangement of functional materials, where the rate and volume of ISF sampling can be modulated by adjusting the applied current density. These patches are typically designed with two electrodes that function as a source of direct current (DC). Various RI‐based platforms with integrated sensors have hitherto been established. For example, Pu et al. designed an entirely inkjet‐printed patch (Figure 30e) that features a Na^+^ ion–based electrode amenable to differential calibration as well as a thermal activation unit, effectively mitigating interference due to variations in sweat secretion and temperature.^[^ ^331^ ^]^ To enhance skin conformity and sensitivity, Chen et al. employed transfer printing methods to develop an ultrathin nanotextured device measuring less than 50 µm, which shows excellent sensitivity and is capable of detecting glucose at micromolar concentrations (Figure 30f).^[^ ^332^ ^]^ However, the necessity for applying DC for a few minutes prior to each measurement leads to inherent constraints on the sampling rate. This limitation has been addressed by combining microneedle systems with RI to expedite ISF collection and enhance the consistency of the sampled ISF. Cheng et al. introduced a glucose monitoring device that included a touch‐actuated RI‐based microneedle platform, which increased the rate of ISF extraction by ≈1.6 times.^[^ ^333^ ^]^

Non‐enzymatic glucose sensors have attracted considerable research interest due to their potential to address certain limitations of enzyme‐based systems, such as enzyme denaturation, limited shelf life, and dependency on oxygen or artificial mediators. These platforms promise enhanced chemical robustness and temperature tolerance by utilizing direct electrocatalytic oxidation of glucose with noble metals (Pt, Au, Pd) or transition metal/metal oxide nanostructures (Ni, Cu). However, despite these advantages, several fundamental limitations have hindered their clinical adoption.^[^ ^334^ ^]^

Selectivity remains a significant challenge for non‐enzymatic sensors, primarily due to their susceptibility to interference from various electroactive substances commonly found in physiological fluids, such as ascorbic acid, uric acid, dopamine, lactate, and other sugars, which can lead to overlapping oxidation signals. Unlike enzyme‐based systems that exhibit high molecular recognition specificity for glucose, non‐enzymatic platforms lack this intrinsic selectivity, increasing the risk of inaccurate readings. Additionally, surface fouling and stability issues considerably impact long‐term performance, as the adsorption of proteins, lipids, and biofilm components onto the electrode surface can obstruct active catalytic sites, reducing sensitivity over time. Metal oxide catalysts may also experience gradual morphological or chemical changes under physiological conditions, including dissolution, passivation, or instability of the oxide layer, ultimately shortening their operational lifespan. Many non‐enzymatic sensors demonstrate optimal catalytic activity only in alkaline media, which is incompatible with the near‐neutral pH found in biological fluids like blood or interstitial fluid, necessitating sensitivity and reaction kinetics trade‐offs. Furthermore, higher operational potentials are typically required to facilitate direct glucose oxidation, increasing susceptibility to electrochemical noise and heightening interference from other redox‐active biomolecules.^[^ ^335^ ^]^ Combining these challenges are batch‐to‐batch variability in nanomaterial synthesis, scalability concerns, and the absence of standardized manufacturing protocols, which collectively hinder the transition of these sensors from laboratory prototypes to clinically approved devices.^[^ ^336^ ^]^

In contrast, enzyme‐based glucose sensors, particularly those utilizing glucose oxidase, continue to dominate the commercial market due to their unmatched specificity, reproducibility, and proven performance in real‐world continuous glucose monitoring (CGM) systems. While non‐enzymatic sensors show promise as a research avenue, significant advancements in selectivity engineering, anti‐fouling surface treatments, and catalysis under physiological conditions are necessary before they can realistically replace enzyme‐based platforms in clinical diagnostics.^[^ ^334^ ^]^

**Figure 31:**
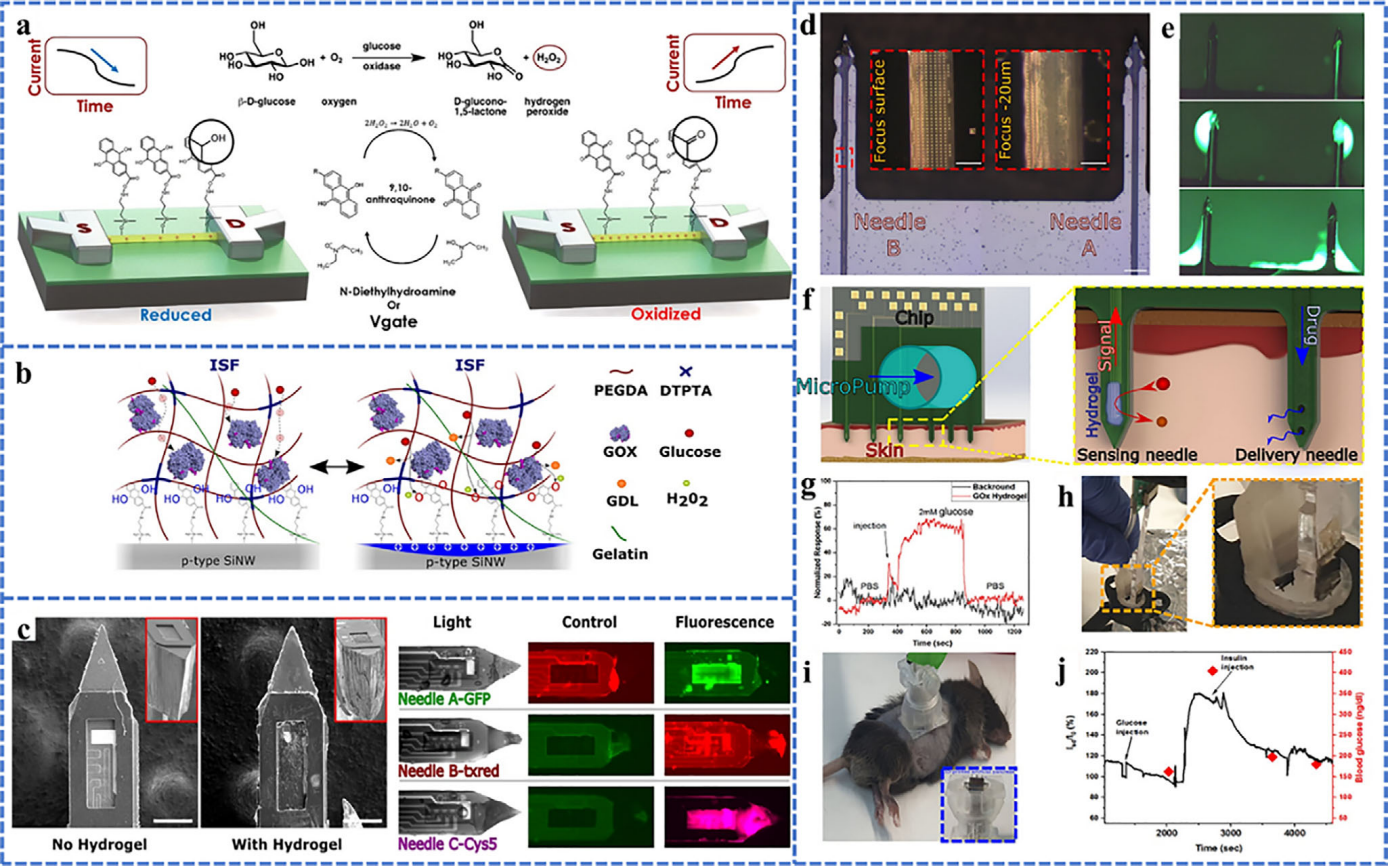
Integrated microneedle‐based biosensing and drug delivery system utilizing AQ‐modified SiNW‐FET and hydrogel coatings. a) Reaction diagram of AQ surface‐modified SiNW‐FET in the presence of metabolite and its oxidizing enzyme. b) Schematic of the hydrogel structure and glucose reaction mechanism applied to a SiNW device. c) SEM images of a Si microneedle with or without applied hydrogel (scale bar: 100 µm). The red contour inset shows a tilted image of the needles. The hydrogel was applied to microneedles set on the same die, where different fluorescent species were added to the hydrogel of each microneedle. d) Microscope image of two microneedles with a 5 mm long microchannel (scale bar: 20 µm). Red dashed insets show the array of cavities forming the channel (scale bar: 20 µm). e) Images of fluorescent solution ejection through two separate microfluidic microneedles. The top caption was taken just before the fluid release from the edge of the microneedle. At the bottom, the solution is withdrawn by the microneedles. f) Illustration of the clinic‐on‐a‐needle platform. The integrated chip, connected to a bundled electrical monitoring system and micropump/reservoir device, penetrates the skin and is immobilized. g) In vitro simultaneous sensing and injection experiment results. h) Both delivery microneedle and sensor microneedle elements in a tube during the experiment. i) Sedated C57BL/6 mouse with a 3D‐printed artificial pancreas patch holding the microneedle chip in place during a continuous glucose measurement. Blue dashed contour inset shows an inverted look at the microneedles passing through the patch apparatus. j) In vivo mouse glucose level measurements after 16 h of fasting. Reproduced with permission.^[^ ^56^ ^]^ Copyright 2021 American Chemical Society.

Patolsky et al. developed an innovative microneedle‐ and IFS‐based transdermal and versatile sensor for multiplex and continuous monitoring of biomarkers.^[^ ^56^ ^]^ This study describes the modification of the fabrication method to enable the on‐chip integration of microinjection needle components, which are ideally suited for employment as a drug delivery system, as shown in **Figure** **31**. This single‐chip multifunctional platform directly monitors clinically relevant biomarkers in ISF while simultaneously facilitating synchronized transdermal drug delivery. Figure 31a,b shows the illustrations of the chemically active SiNW‐FET biosensor modified with AQ and hydrogel and the integration of microneedles with an embedded microfluidic system for drug delivery, respectively.

### 12.2 Chloride (Cl−) Testing for Cystic Fibrosis

ISF‐based assays present a promising alternative to sweat chloride testing for cystic fibrosis (CF) by addressing several inherent limitations that compromise the accuracy and reliability of traditional sweat‐based diagnostics. While sweat chloride concentration is the current gold standard for CF screening and diagnosis, the method is highly susceptible to variability from sweat rate fluctuations, insufficient sample volumes, particularly in newborns or individuals with low sweat output, evaporation, and contamination from skin surface residues. These factors can lead to technically inadequate samples or inconclusive test results, necessitating repeat testing and delaying clinical decision‐making.^[^ ^337^ ^]^ Indeed, ISF offers a stable and consistent electrolyte composition, closely resembling that of blood plasma, and is less affected by external factors. It can be accessed through minimally invasive methods like microneedle arrays, allowing continuous or on‐demand sampling without external stimulation. This approach avoids the physiological variability of sweat and provides more reliable chloride measurements. Because ISF is regulated under homeostatic conditions, it may better reflect systemic electrolyte imbalances, making it a promising diagnostic fluid for cystic fibrosis (CF). Integrating ISF‐based platforms with wearable or point‐of‐care sensors could significantly enhance the sensitivity and reliability of chloride detection in CF screening, particularly for early‐life diagnostics. However, further clinical validation and regulatory approval are needed to confirm ISF assays as a definitive alternative in diagnostics.^[^ ^43^, ^338^ ^]^

### 12.3 Non‐Invasive Drug Monitoring

Numerous studies on noninvasive drug monitoring have focused on recreational and therapeutic drugs, particularly on illegal and addictive drugs. Various therapeutic dosages of levodopa, vancomycin, theophylline, and others have improved therapeutic outcomes.^[^ ^339^ ^]^ Additionally, the abuse of recreational drugs, including opioids and morphine, poses a significant threat to public health and social stability. Consequently, substantial efforts have been made to fabricate technologies allowing real‐time drug monitoring. These innovations aim to personalize drug dosages, improve therapeutic efficacy, and prevent drug misuse.

**Figure 32:**
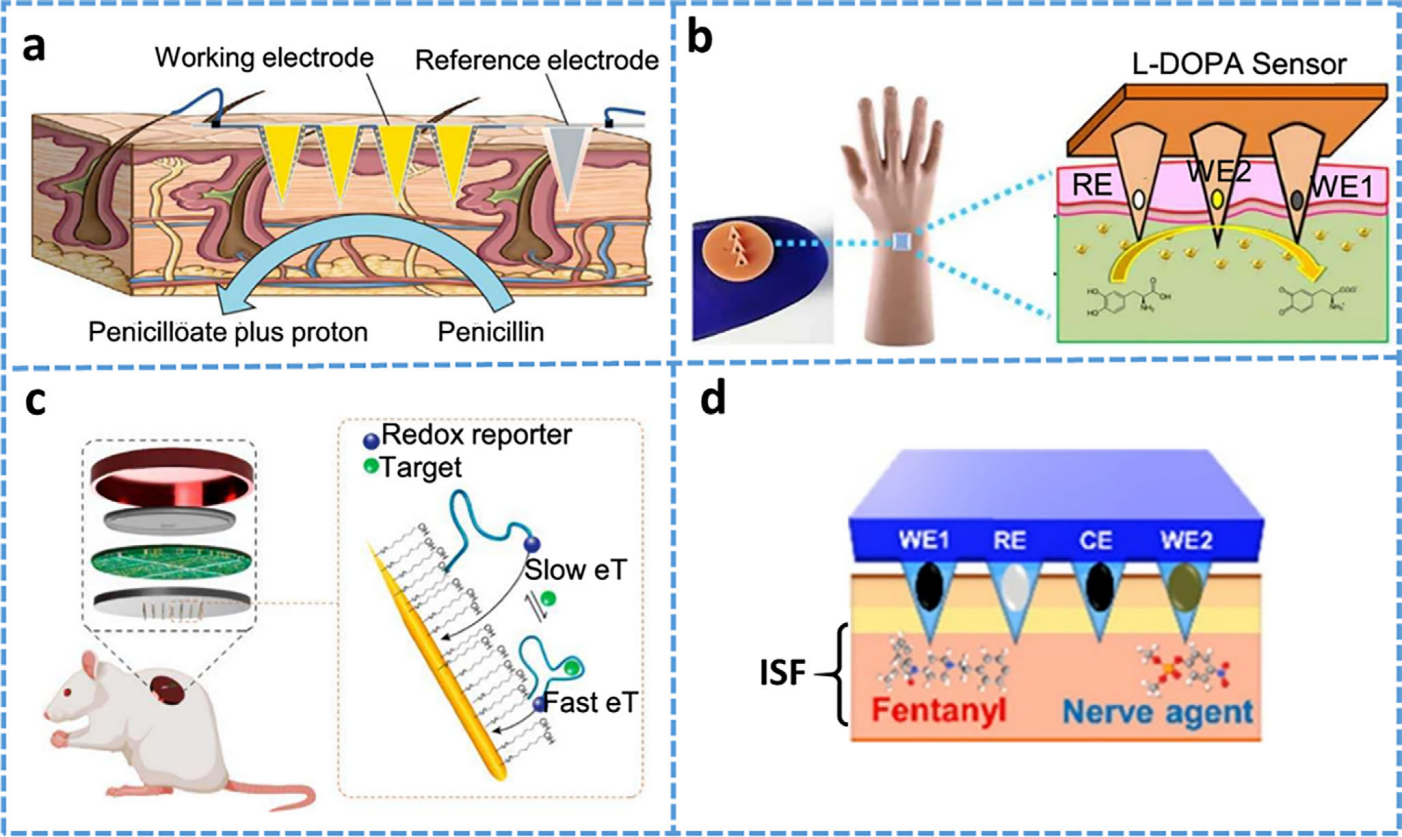
Sensors for ISF‐based drug monitoring. a) Microneedle‐based sensor for monitoring phenoxymethylpenicillin levels. b) Dual‐sensing platform for the estimation of levodopa levels. c) Microneedle aptamer‐based sensor for the estimation of multiple targets. d) Microneedle‐based sensing platform for the measurement of fentanyl and organophosphates. Reproduced with permission.^[^ ^43^ ^]^ Copyright 2024 Nature.

The use of ISF‐based sensors for TDM offers advantages over conventional blood‐based methods, as it allows for the selective measurement of the bioavailable, unbound form of drugs. Unlike blood‐based detection, which frequently measures the concentrations of both bound and unbound forms of drugs, the ISF‐based sensors provide a more precise estimation of the concentration of active drugs in the body.^[^ ^340^ ^]^ In a pioneering study, Rawson et al. reported the design of an ISF sensor‐based microneedle aimed at monitoring the drug phenoxymethylpenicillin in a healthy individual, as shown in **Figure** **32**a; the device will be evaluated in a first‐in‐human trial.^[^ ^341^ ^]^ This sensor incorporates a working electrode coated with iridium oxide, designed to detect variations in pH, along with a hydrogel layer containing β‐lactamase, which catalyzes the conversion of diffused phenoxymethylpenicillin to penicillate and a proton; an increase in the proton concentration results in a concomitant decrease in pH. The open‐circuit potential output of the sensor correlates with phenoxymethylpenicillin concentration, and the results obtained were comparable with those obtained using microdialysis. Goud et al. combined tyrosinase‐based enzymatic amperometry with non‐enzymatic voltammetry to enable dual sensing of levodopa. This approach helps ensure the measured concentration's accuracy and reliability, as shown in Figure 32b. This sensor platform provides efficient real‐time feedback for the analysis of levodopa, enhancing the quality of available data for diagnostic purposes. However, a detection strategy that is reliant on the enzymatic activities of oxidases or reductases limits the scope of detectable target molecules. By contrast, aptamer‐based sensors allow for a significant expansion in the range of detectable targets. For example, a microneedle aptamer‐based sensor for the detection of cancer medications and antibiotics, as illustrated in Figure 32c. They employed aptamers containing single‐stranded nucleic acid sequences, which were selected using the systematic evolution of ligands by exponential enrichment (SELEX) method. The sensor was employed for monitoring therapeutic drugs such as irinotecan, doxorubicin, and tobramycin under both in vitro and in vivo conditions and found to demonstrate high sensitivity in real time as well as alignment with PK models. These aptamers were designed to exhibit high‐affinity interactions with a broad range of target molecules, allowing for the simultaneous measurement of multiple targets using a single platform.

In addition to TDM, the continuous tracking of recreational drugs has received considerable attention for the prevention of drug abuse. For instance, Mishra et al. developed a microneedle‐based ISF sensor capable of concurrently detecting fentanyl and organophosphates, as shown in Figure 32d.^[^ ^278^ ^]^ The sensor is equipped with two working electrodes specifically designed for the independent detection of fentanyl and organophosphates. This configuration enables the distinction between an overdose of opioids and poisoning with nerve agents, as both conditions can present similar symptoms.

### 12.4 Monitoring of Hormone and Protein Levels in ISF

The small size of hormones allows them to pass through the walls of capillaries into the ISF. Specifically, steroid hormones such as cortisol are frequently associated with albumin and several protein carriers.^[^ ^342^ ^]^ As a result, the focus of hormone monitoring has shifted from measuring the total concentrations to that of the unbound, bioavailable hormone fractions. ISF‐based sensors typically detect these unbound fractions to reflect the levels of active hormones more accurately. Cortisol is a common hormone that regulates the immune system and functions as a significant indicator of stress response; the detection of cortisol has garnered considerable interest from the healthcare community. For example, Venugopal et al. reported the development of a device that employed EIS to allow monitoring of cortisol levels in real time. In addition, this was the first study to elucidate the association between cortisol concentrations in ISF and plasma.^[^ ^343^ ^]^

**Figure 33:**
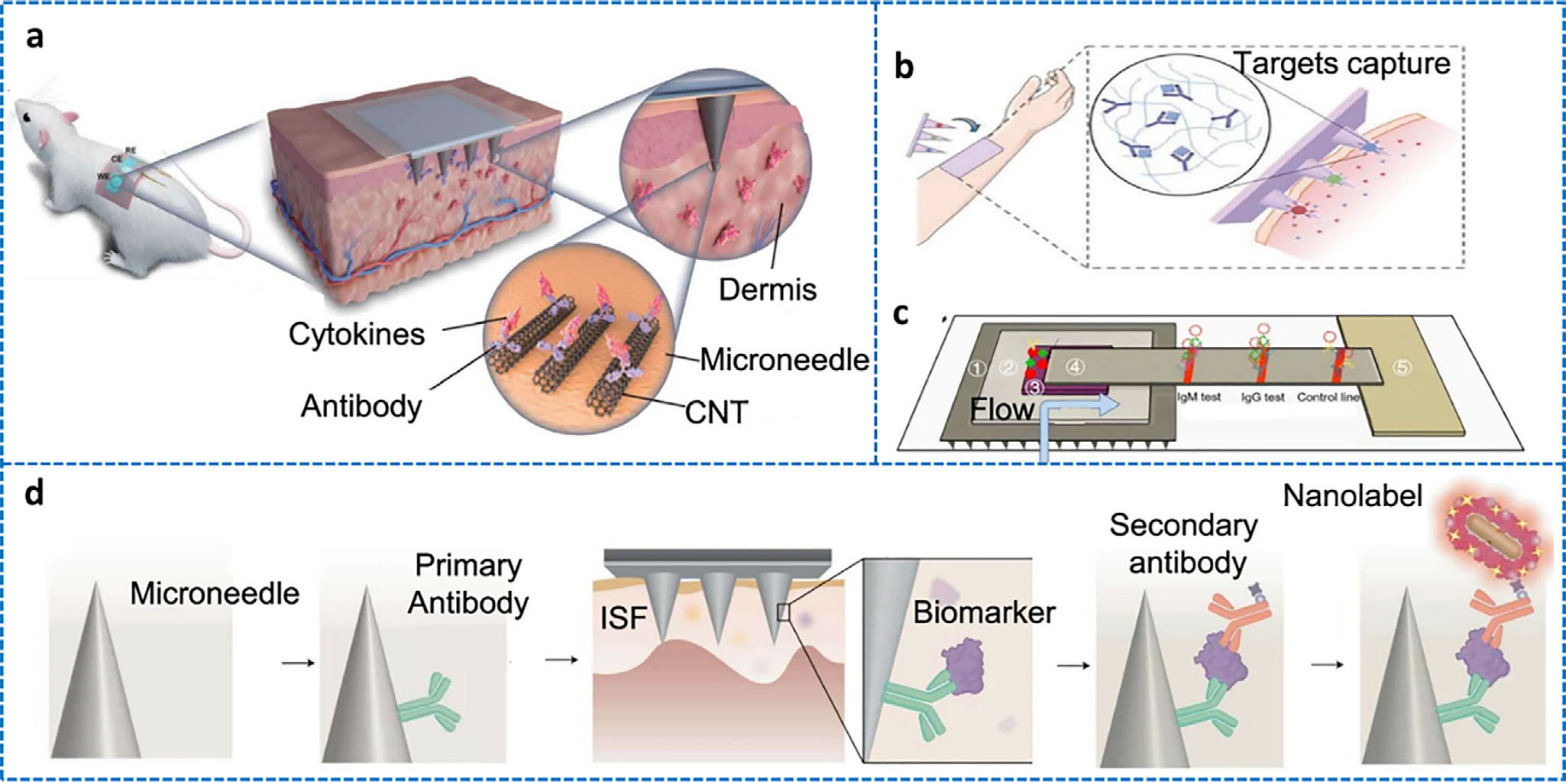
ISF‐based sensors for monitoring macromolecules. a) Platform for the capture and measurement of proteins in ISF. b) Encoded microneedle arrays for optical detection of multiple cytokines. c) Porous microneedle patch for swift detection of anti‐SARS‐CoV‐2 IgM/IgG antibodies. d) Microneedle patch for the detection of cytokines and antibodies. Reproduced with permission.^[^ ^43^ ^]^ Copyright 2024 Nature.

Cytokines are small proteins that play a crucial role in controlling the activities and functions of immune and blood cells. The monitoring of cytokine levels in ISF can provide valuable insights into immune responses. “Cytokine storm” is the term used to describe the rapid release of several types of cytokines at the onset of an immune response.^[^ ^344^ ^]^ Recent studies have reported the development of an innovative strategy for monitoring cytokines, which provides detailed information to facilitate the rapid diagnosis and treatment of inflammation. For example, Xu et al. developed the first real‐time platform designed to capture and quantify proteins in ISF, as shown in **Figure** **33**a.^[^ ^345^ ^]^ Functionalized carbon nanotubes were incorporated into microneedles for the collection of cytokines via the use of specific antibodies. Additionally, this platform enables real‐time electrochemical analysis of cytokines by integrating capture with detection based on electrochemical methods, circumventing the need for the removal of microneedles to allow measurements of cytokine levels. Optical techniques are widely employed for the detection of cytokines owing to the simplicity of observation. For example, Zhang et al. developed an advanced approach for the simultaneous detection of multiple cytokines by integrating PhC barcodes with microneedles, as illustrated in Figure 33b.^[^ ^309^ ^]^ The penetration of skin by the microneedles is followed by the rapid adhesion of cytokines to the barcode‐equipped microneedle tips. The addition of fluorescent probes for the generation of immunocomplexes allowed the determination of the relative amounts of cytokines based on the fluorescence intensity of the barcodes. Moreover, the cytokines may also be distinguished based on differences in the corresponding reflection peaks.

Antibodies are important proteins produced by the immune system in response to antigens. The measurement of antibody levels provides definitive insights, which are vital for the early diagnosis of diseases. The concentrations of antibodies in ISF are typically 15–25% of the corresponding levels in blood.^[^ ^86^ ^]^ Bao et al. developed a diagnostic system featuring a porous microneedle patch in conjunction with immune‐chromatography, enabling the rapid detection of IgM/IgG antibodies specific for severe acute respiratory syndrome coronavirus 2 (anti‐SARS‐CoV‐2), as shown in Figure 33c.^[^ ^310^ ^]^ The study involved the collection of ISF using a biodegradable porous microneedle patch. The collected ISF then flowed to a paper‐based sensor combined with a colloidal gold immunoassay for analysis. The results were visualized by observing the color bands on the sensor. Wang et al. monitored both cytokines and antibodies utilizing a microneedle patch, as shown in Figure 33d.^[^ ^346^ ^]^

### 12.5 Monitoring of Other Metabolic Biomarkers in ISF

**Figure 34:**
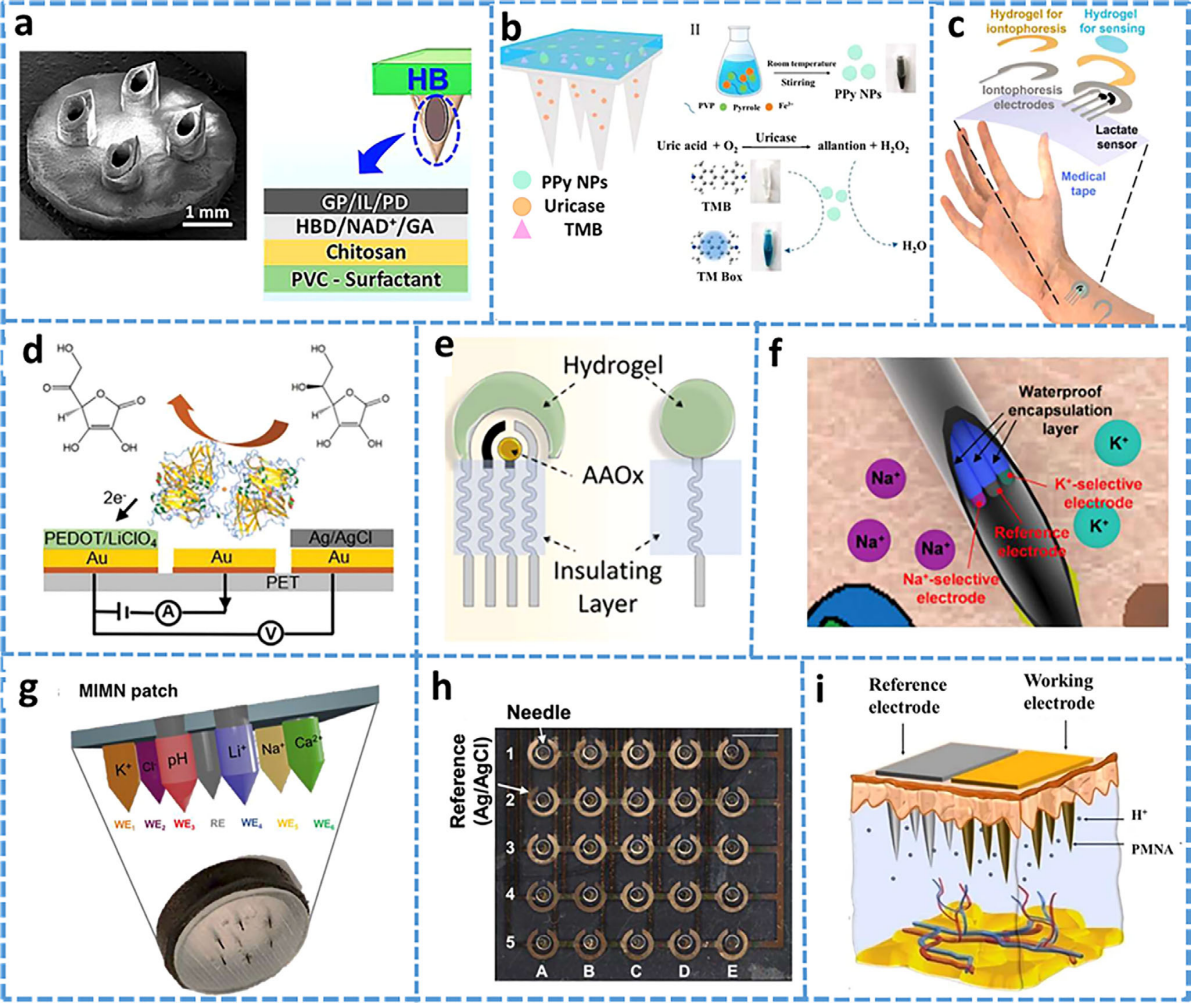
ISF‐based sensors for the detection of other small molecules and biomarkers. a) SEM image and layer‐by‐layer illustration of a sensor for ketone bodies. b) Colorimetry‐based uric acid sensor and mechanism of detection. c) Schematic illustration of a wearable lactate sensor for the quantification of lactate levels in ISF. d) Schematic illustration of the structure and sensing mechanism of a wearable ascorbic acid sensor. e) Schematic illustration of a stretchable sensor for vitamin C. f) Configuration of a sensor for Na^+^ and K^+^ ions integrated with a hollow microneedle. g) Optical image and schematic illustration of a microneedle‐based sensor for the detection of multiple ions. h) Conformable pH sensor array enabling the generation of a pH map. i) Schematic illustration of a high‐density microneedle‐based pH sensor. Reproduced with permission.^[^ ^43^ ^]^ Copyright 2024 Nature.

The aforementioned techniques can also be utilized to detect different metabolic biomarkers, which aid in diagnosing and monitoring conditions such as gout, extreme diabetic ketoacidosis, and phenylketonuria. For example, ketone bodies in ISF have been effectively measured using hollow microneedles equipped with sensing materials within their cavities, as shown in **Figure** **34**a.^[^ ^317^, ^347^, ^348^, ^349^ ^]^ A system utilizing nicotinamide adenine dinucleotide (NAD⁺) and β‐hydroxybutyrate dehydrogenase was employed for the detection of ketone body precursors; stable and continuous monitoring of their levels can be achieved using this method. Additionally, the crystallization of the biomarker uric acid, a byproduct of purine metabolism, in joints causes gout. Zhang et al. developed a colorimetry‐based microneedle sensor for the detection of uric acid, which utilizes uricase and 3,3′,5,5′‐tetramethylbenzidine (TMB; Figure 34b).^[^ ^350^ ^]^ H_2_O_2_ is produced as a result of the reaction between uricase and uric acid, and reacts with TMB to cause a color change; the linear detection range for uric acid using this approach was found to be 200–1000 µM. Although this sensor was effectively tested for uric acid on porcine skin, the lack of in vivo data diminishes the impact of the study. The detection of lactate in sweat has been the focus of previous studies, as aforementioned in the text; however, lactate levels have also been estimated in ISF. The lactate concentration in ISF is considered more reflective of serum levels compared to that in sweat, owing to the dilution effect associated with sweat samples.^[^ ^351^ ^]^ Sensors have been developed to monitor high lactate levels in ISF, which reflect anaerobic metabolism and muscle fatigue. These sensors utilize sampling methods and detection principles similar to those of glucose sensors. Both microneedle‐ and RI‐based techniques, as illustrated in Figure 34c, have been employed for ISF extraction, and comparable sensing mechanisms to those of glucose sensors, but involving the enzyme lactate oxidase have been used for detection.^[^ ^352^, ^353^ ^]^ While in vitro studies have revealed the potential of these sensors for the dynamic monitoring of lactate levels, comprehensive evaluations of accuracy in human subjects remain in the early stages, much like those for glucose monitoring.

Unlike metabolic biomarkers that exhibit strong correlations with diseases, nutrient biomarkers can be employed to prevent and manage nutritional imbalances. For instance, the common nutrient vitamin C can serve as a biomarker for monitoring whether the daily nutrient requirements are met. Instead of the conventional methods of estimating vitamin C levels based on its oxidation, Sempionatto et al. developed a stretchable RI patch (Figure 34e) that incorporated ascorbate oxidase, enabling continuous measurements of vitamin C with high specificity.^[^ ^354^ ^]^ Studies on human subjects following the administration of various doses of vitamin C (in the form of tablets) validated the ability of the sensors to detect changes in vitamin C levels within the body in real time. The approach of Zhao et al. was similar to that illustrated in Figure 34d and involved comparable recognition mechanisms, but after enhancing the sensor with a wireless setup, enabling long‐term monitoring for over 24 h.^[^ ^355^ ^]^

### 12.6 Monitoring pH and Electrolyte Concentrations

Electrolytes, which chiefly comprise ions such as Cl^−^, zinc (Zn^2+^), K^+^, and Na^+^, have vital functions in our bodies, including the maintenance of a stable chemical microenvironment in cells and tissues, the determination and maintenance of membrane potentials of cells, and roles as biomarkers for certain diseases. Both K^+^ and Na^+^ ions are important biomarkers, the levels of which correlate with the overall levels of electrolytes in the body and are associated with conditions such as hypertension and cardiovascular disease. This has prompted the development of devices that enable accurate and continuous monitoring of these ions. However, the electrical stimulation needed for RI‐based approaches can affect the composition of ISF through a process known as electroosmosis,^[^ ^40^ ^]^ leading to an accumulation of these ions near the cathode. Therefore, microneedle‐based techniques are frequently employed to measure the levels of Na^+^ and K^+^ in ISF. For example, a hollow microneedle‐based device (Figure 34f) has been equipped with electrodes sensitive to both K^+^ and Na^+^ ions, simultaneously measuring both ions.^[^ ^356^ ^]^ A porous microneedle paired with a flat, screen‐printed Na^+^‐sensing electrode was evaluated in preliminary human trials to validate its effectiveness as a monitoring device.^[^ ^357^ ^]^ Additionally, a solid microneedle‐based sensor array (Figure 34g) was introduced for in vivo applications and found to be capable of measuring the levels of various ions such as Na^+^, K^+^, Cl^−^, Li^+^, and Ca^2+^, demonstrating the feasibility of its application for the comprehensive monitoring of electrolyte levels.^[^ ^358^ ^]^

The pH of biofluids is a critical indicator of human health, as deviations in the pH of biofluids, particularly in specific body areas such as the limbs, often signal underlying health issues. ISF is derived directly from circulating blood and provides a more accurate representation of systemic changes in biomarkers compared to sweat, which is produced by the eccrine glands. Furthermore, ISF is a more reliable medium than sweat for the measurement of pH, as it is less prone to interference from chemicals in the epidermal layer as well as bacterial byproducts. Several pH sensors have been developed for this purpose. For example, Lee et al. developed a flexible microneedle array designed for determining localized pH in various regions of the limbs, as illustrated in Figure 34h.^[^ ^359^ ^]^ A pH map covering 25 distinct regions was effectively generated using this device, revealing pH variations in areas impacted by peripheral artery disease. Additionally, Dervisevic et al. developed a high‐density microneedle array, achieving an enhanced sensitivity of 62.9 mV per pH unit across an expanded detection surface, as illustrated in Figure 34i.^[^ ^360^ ^]^

### 12.7 Biocompatibility of Microneedle‐Based Sensing Platforms

**Figure 35:**
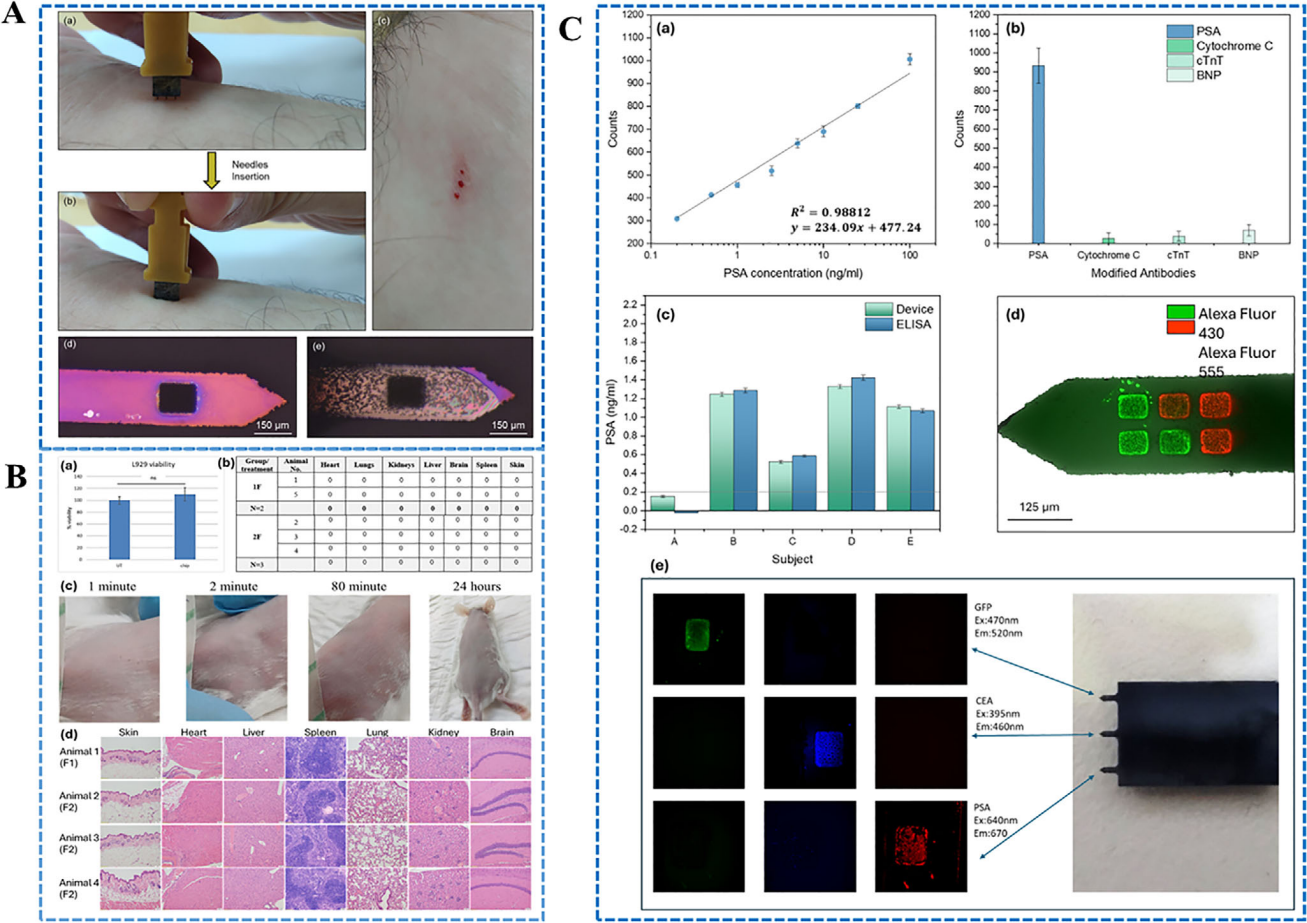
A) Visual imaging of the insertion of the microneedle‐based device into the skin. a) The initial pricking step of the microneedle device is connected to a 3D printed holder. b) Fully inserted microneedle device inside a volunteer's arm. c) Three puncture holes for three needles on the device, with visible blood droplets as a result of the capillary network rupture during pricking. d) Optical microscope image of the microneedle with the silica protective window before contact with the blood droplet. e) Optical microscope image of the microneedle after contact with the blood droplet, showing red blood cells on the needle surface. B) In vivo and in vitro biocompatibility of the device. a) Effect of the microneedles on L929 cells viability. The left and right columns show the results of the untreated and treated cells, respectively. b) Summary of the semiquantitative analysis of the histological findings, using a scoring method of five grades (0‐–4), for the severity of the pathological changes, c) Test subjects’ images directly after, 2 min, 80 min, and 24 h after pricking. d) Histological photographs of H&E‐stained organs. C) Sensitivity of the microneedle‐based device to PSA in capillary blood evaluated via in vivo intradermal measurements. Reproduced with permission.^[^ ^58^ ^]^ Copyright 2024 American Chemical Society. a) Linear response curve to increasing PSA concentrations in spiked bovine serum samples (*N* =10). b) Specificity measurements, each modified with PSA antibody and introduced to different protein solutions: Cytochrome C, BNP, cTnT, and PSA. The results clearly show that only the specific PSA protein biomarker leads to a high fluorescent response, while the nonspecific proteins show a negligible response. c) In vivo measurements in five human volunteers for quantification of capillary blood PSA concentration using the SiNPs device (cyan bars, *N* = 3), in comparison to venous blood‐based ELISA measurements (blue bars). d) Fluorescence image of the multiplex detection of two different fluorophores on the multiple‐sensing area device. e) Fluorescence image of multiplex detection with three different antigens on the same chip. Reproduced with permission.^[^ ^58^ ^]^ Copyright 2024 American Chemical Society.

The process of inserting the microneedles into the skin is illustrated in **Figure** **35**a. As shown in Figure 35b, the application of microneedle sensors did not compromise cell viability or negatively impact the health of mice. Additionally, immune or inflammatory reactions were not observed in the region where the microneedles were applied. The impressive biocompatibility of the silicon nanoparticle–embedded microneedles indicate their significant potential for future clinical applications. Prior to experiments involving in vivo intradermal measurements of biomarkers in capillary blood, the sensitivity of the microneedle‐based device to the protein biomarker PSA was assessed using in vitro experiments, as shown in Figure 35c.

## 13 Challenges of ISF‐Based Sensing Platforms and Potential Solutions

ISF‐based detectors hold considerable promise for the diagnosis and management of various diseases as well as drug monitoring by enabling the detection of critical biomarkers and pharmaceuticals with minimal or no invasiveness. These advancements can greatly enhance personalized medicine and support POCT; however, certain challenges remain to be addressed, including the long‐term durability of the ISF‐based sensors and their ability to allow continuous monitoring over extended durations. Current reports indicate that the sensors for small molecules, including glucose and lactate, can be effectively maintained for over 24 h. A notable example is glucose sensors based on the highly selective and durable enzyme glucose oxidase. Commercially available sensors such as G6 and G4 from Dexcom, Freestyle Libre from Abbott, DEXCOM G5, and MiniMed Guardian from Medtronic are designed to function effectively for over a week. Nevertheless, only a few highly specific and reliable enzymes have been engineered or identified for specific analytes. Bioaffinity‐based sensing is a technique employed to capture target molecules using specific binding agents such as aptamers, antibodies, or synthetic receptors. However, the detachment of the targets from these binding agents can be particularly challenging, especially for sensors designed for high target selectivity and sensitivity. The accumulation of bound targets on the sensing surface can lead to decreased effectiveness of the device and inaccuracies in measurements. Various strategies have been proposed for addressing this issue, including the application of external stimuli for regenerating the sensing element and the development of mechanisms for releasing the bound targets.^[^ ^361^ ^]^ Additionally, the achievement of high accuracy and reliability represents another challenge with the ISF‐based sensors. Issues in real‐time monitoring applications, such as the buildup of analytes and byproducts, as well as biofouling on the sensing surfaces, can limit target recognition and device response. The application of biocompatible outer coatings, such as zwitterionic membranes or hydrogels, can significantly minimize biofouling and reduce interferences due to the immune response, although this approach necessitates meticulous molecular design.^[^ ^287^, ^362^, ^363^ ^]^ Monitoring biomarkers without the use of devices that support in situ detection involves frequent, continuous, and extensive extraction or transportation of ISF to facilities that allow in vitro analysis, which can lead to localized immune responses on the skin and repelling effects. This process may also impact the composition, including the presence and levels of various components of ISF, resulting in an inaccurate correlation between biomarker levels in ISF and blood and, ultimately, inaccurate measurements. This may be addressed by developing systems that regulate the volume of extracted ISF as well as the timing and frequency of extraction. A regulation of these factors is expected to help minimize the chances of triggering an immune response or affecting target concentrations, thereby ensuring the accuracy of measurements.

The impact of mechanical strain associated with movement on the accuracy, stability, and durability of portable ISF‐based sensors represents another challenge related to the use of these devices. Although the versatile, portable device offers convenience and supports easy diagnostics, it also subjects the sensors to substantial mechanical stress during regular human activity. Frequent and significant strains can cause the detachment of the sensing elements from the skin and physical damage to the sensor element. The development of a flexible substrate designed to be insensitive to mechanical strain can help alleviate this issue by reducing interference at the sensor interface.^[^ ^364^, ^365^ ^]^ However, the development of the flexible sensor for commercialization purposes is hampered by the presence of rigid–flexible interfaces and the poor compatibility of silicon‐based materials with the widely utilized flexible substrates.

The development of devices that allow long‐term monitoring of biomarkers with reliability and accuracy, particularly that of larger biomolecules such as cytokines, cortisol, and proteins, should form the focus of future research. Advances in ISF‐based sensing technologies have the potential to revolutionize personalized diagnostics and treatment, particularly for the management of immune‐related diseases. There is a significant need for biochemical and physical sensing membranes of greater durability than the currently available ones, which should be capable of withstanding the complexities of biological environments and human activity demands. In other words, the development of membranes that are resistant to biofouling and capable of enduring physical strain remains a necessity. Additionally, further in vivo research into the detected concentrations of biomarkers in ISF and their corresponding concentrations in blood is expected to increase confidence in the use of ISF for biosensing applications. Recently, a few studies have shown that there is an analyte‐specific lag‐time between blood and ISF, although ISF has a plasma‐like composition.^[^ ^366^ ^]^ In diabetes management, continuous glucose monitors (CGMs) such as the Dexcom G7 and FreeStyle Libre typically exhibit a delay of 5 to 10 min for ISF glucose levels compared to blood glucose levels, particularly during rapid changes such as after meals or in cases of hypoglycemia.^[^ ^367^ ^]^ In athletes, studies on electrolyte monitoring reveal that sodium and potassium levels in ISF achieve equilibrium with blood levels within minutes, making them reliable indicators of hydration status, though they may be less responsive during acute electrolyte disturbances. Conversely, inflammatory cytokines have much longer lag times. For instance, interleukin‐6 (IL‐6) and tumor necrosis factor‐alpha (TNF‐α) in ISF have been reported to rise 20 to 40 min after elevations in plasma levels during exercise or trauma‐induced inflammation. Additionally, pharmacokinetic studies on drugs like vancomycin and methotrexate show that ISF concentrations generally reflect plasma levels, but with a temporal offset of 10 to 20 min, depending on the drug's size and lipophilicity. Overall, these findings highlight that while ISF can accurately reflect systemic biochemical levels, it is important to consider the specific lag times for different analytes.^[^ ^368^ ^]^ However, short lag times are typically acceptable for monitoring chronic diseases, while longer delays may limit the use of ISF in acute diagnostic situations.

The true clinical impact of wearable ISF biosensors will be fully realized when their continuous, high‐resolution data streams are seamlessly integrated with AI‐driven health platforms. In such systems, raw biomarker measurements, like glucose, electrolytes, metabolites, and inflammatory markers, can be transmitted in real time to secure cloud‐based or on‐device machine learning algorithms.^[^ ^369^ ^]^ These algorithms can process and interpret dynamic trends, filter out noise, and correlate fluctuations in biomarkers with individual patient profiles, lifestyle factors, and additional physiological data gathered from complementary wearable sensors, such as heart rate, activity levels, and sleep patterns. This integration enables real‐time analytics for the early detection of deviations from personal baselines, the prediction of adverse health events, and dynamic risk stratification. Furthermore, AI‐driven personalization allows diagnostics to move beyond static, population‐based thresholds by tailoring clinical decision‐making to an individual's unique biomarker profile. In a closed‐loop system, such platforms could trigger automated therapeutic responses, like adjusting insulin dosing for diabetes management, or generate actionable alerts for patients and healthcare providers.^[^ ^370^ ^]^ Thus, integrating ISF‐derived biomarker data with intelligent analytics frameworks represents a transformative step toward proactive, precision health management, effectively bridging wearable diagnostics with personalized medicine.^[^ ^371^ ^]^

## 14 Clinical Trials of Commercially‐Available Diagnostic Platforms

Despite significant research on the development of microneedle sensors, the availability of commercially viable microneedle devices in the market remains limited. Microneedle sensors have been predominantly commercialized for CGM, with notable examples being G6 from Dexcom, Freestyle Libre two from Abbott, and Guardian from Medtronic. These devices offer real‐time monitoring of glucose levels in patients with diabetes, providing a convenient alternative to the conventional finger‐pricking methods for blood sampling. Despite the commercial success of CGM devices, their high cost and limited operational lifespan have hindered the widespread adoption of microneedle‐based technologies. The development of cost‐effective and scalable methods for the manufacture of microneedles holds significant potential for enhancing the practical application of these sensors. The successful clinical diagnostic platforms are described in **Table** **6**, including clinical settings that require FDA or CE approval and face challenges such as cost, reliability, and user acceptance.

**Table 6:** Successful clinical diagnostic platforms: applications, device type, regulatory status, and translational challenges.

S.No	Platform	Diagnostic application	Device/Technology type	Regulatory status (FDA/CE/Other)	Translational challenges	Remarks
1	Dexcom G7, Abbott FreeStyle Libre, Senseonics Eversense E3	Diabetes: continuous glucose monitoring	Wearable sensor (transdermal patch/ implantable sensor)	FDA: Dexcom (510(k)), Libre (510(k)), Eversense (PMA); CE‐marked	High device/consumable cost; adhesive reactions; training burden; reimbursement dependence	Widely adopted in diabetes care; Medicare expansion increased access
2	Abbott ID NOW	Infectious diseases (COVID‐19, Strep, Flu)	Portable molecular diagnostic platform (POC NAAT)	FDA: EUA, 510(k); CLIA‐waived; CE‐marked	Reliability in asymptomatic users; supply constraints; per‐test cost	Rapid decentralized infectious disease testing
3	Abbott i‐STAT Alinity	Blood gases, electrolytes, troponin	Handheld cartridge‐based chemistry analyzer	FDA: 510(k); CLIA‐waived/moderate complexity; CE‐marked	Cartridge cost, operator training, LIS/EHR integration	Standard in ED/ICU bedside testing
4	Cologuard® (Exact Sciences)	Colorectal cancer screening	Home stool collection + lab‐based DNA/FIT analysis	FDA: PMA; CMS reimbursed; CE‐marked	Cost vs. FIT; follow‐up compliance; user acceptance	Increased colorectal screening rates
5	Guardant360 CDx, FoundationOne Liquid CDx	Oncology (liquid biopsy for tumor genotyping)	Blood‐based NGS assay (liquid biopsy)	FDA: PMA‐approved CDx; CMS coverage for select cancers; CE‐IVD	High assay cost, payer reimbursement, and need for utility data	Widely used in precision oncology
6	Minuteful Kidney™	Chronic kidney disease (urine ACR)	Smartphone‐based imaging of the dipstick test	FDA: 510(k); CLIA‐waived; CE‐marked	Smartphone variability; patient compliance; provider integration	First FDA‐cleared smartphone‐based lab test
7	IDx‐DR	Diabetic retinopathy screening	AI‐based autonomous software diagnostic	FDA: De Novo clearance; CE‐marked; CMS coverage	Generalizability; workflow integration; liability	First FDA‐cleared autonomous AI diagnostic
8	Apple Watch ECG App / Omron HeartGuide	Atrial fibrillation detection & blood pressure	Consumer wearables (smartwatch/cuffless wrist BP)	Apple ECG: FDA De Novo; CE‐marked; Omron: FDA 510(k), CE‐marked	False positives; physician skepticism; consumer over‐reliance	Strong consumer adoption; shifted digital health acceptance
9	Multi‐Cancer Early Detection (MCED, e.g., Galleri®)	Pan‐cancer blood‐based screening	Liquid biopsy NGS assay (LDT in U.S.)	U.S.: CLIA‐regulated LDT; not FDA‐approved; EU: investigational	High cost, uncertain utility, lack of reimbursement	Large‐scale trials ongoing
10	Microneedle / ISF‐sensing wearables (e.g., glucose, lactate, ketones)	Continuous monitoring of metabolites/drugs	Microneedle patches & minimally invasive biosensors	FDA: limited clearance (glucose only); CE: prototypes CE‐marked; trials ongoing	Biocompatibility; reproducibility; patient comfort; regulatory hurdles	Strong pipeline, not yet mainstream beyond glucose
11	Cuffless optical BP monitors (Aktiia, Hilo)	Continuous non‐invasive blood pressure monitoring	Optical sensor‐based wearable	EU: CE Class IIa (MDR); FDA: OTC clearance (2025, U.S. launch 2026)	Accuracy across physiology/skin tones; clinical trust; guideline acceptance	Potential paradigm shift in hypertension management

The efficacy and safety of microneedle‐based biosensors for the diagnosis of different diseases are presently being evaluated in numerous clinical trials.^[^ ^372^ ^]^ A thorough literature review was performed utilizing the keywords “microneedle” and “biosensor” across various databases, including the National Center for Biotechnology Information, National Library of Medicine (ClinicalTrials.gov), European Union Clinical Trials Register, International Standard Randomized Controlled Trial Number Registry, International Clinical Trials Registry Platform of the WHO, Australian New Zealand Clinical Trials Registry, Chinese Clinical Trial Registry, Japan Pharmaceutical Information Center Clinical Trials Information, Korean Clinical Trial Registry, and the Clinical Trials Registry–India (CTRI).^[^ ^62^ ^]^ The collective demonstration of clinical potential of microneedle‐based sensors in various applications, as shown in **Table** **7**, includes monitoring medication adherence during the treatment of opioid use disorder and disease progression in conditions such as Parkinson's disease and diabetes. The integration of microneedles with advanced electrochemical and molecular technologies is useful for the continuous monitoring of a variety of analytes and has the potential to improve patient care and therapeutic management greatly. Additionally, comparative studies based on the data from clinical trials indicate that the safety and efficacy of microneedle‐based systems are superior to those of conventional monitoring techniques. The potential of applying microneedles in pediatric care is particularly evident, as comfort and ease of use are crucial for enhancing healthcare accessibility and reducing anxiety in children. Despite the encouraging results from clinical trials, a widespread clinical application of microneedle‐based systems and ensuring their efficacy in labs with diverse operating conditions necessitates further research and development. The full potential of microneedle‐based biosensors for enhancing patient outcomes in healthcare across various fields of medicine may be realized by resolving technical challenges and prioritizing the safety and reliability of these devices.

**Table 7:** An overview of clinical trials on microneedle‐based biosensors.^[^ ^62^ ^]^

S.No	Clinical trial	Condition/Disease	Biomarker	Number of subjects
1	KCT0008534 NCT05922176	Allergic rhinitis	RNA	30
2	NCT05998876	Opioid use disorder	Methadone	45
3	NCT05546229	Opioid use disorder	Methadone Buprenorphine	11
4	NCT04735627	Parkinson's disease	Levodopa	20
5	NCT02682056	Glucose	Pediatric diabetes	15
6	NCT04238611	Lactate	Anaerobic thresholds	11
7	NCT01908530	Glucose	Type‐1 diabetes	26
8	NCT03847610	Beta‐lactam Antibiotics	Antimicrobial resistance	11

## 15 Recent Research on Sweat Analysis to Identify Clinically Relevant Biomarkers

Recently, Lee et al. (2025) introduced significant advancements in wearable sweat sensing technology through the development of a bioinspired 3D MIN patch that can capture and analyze ultralow volumes (≥75 nL) of sweat within just 45 seconds.^[^ ^373^ ^]^ While this illustrates impressive technical achievements in fluid handling and near‐infrared optical sensing of metabolites such as vitamins B2, B6, B9, and cortisol, it also reveals several fundamental limitations of using sweat as a diagnostic biofluid compared to ISF. The detection limits reported (1.74–2270 nM) for target analytes are close to the lower physiological ranges found in sweat, which may restrict the clinical utility for low‐abundance biomarkers that are more easily detected in ISF. Furthermore, the study's emphasis on sedentary sweat collection fails to address well‐documented challenges related to sweat variability, including flow rate dependence, dilution effects, and contamination risks from skin surface impurities. These issues are substantially mitigated in ISF‐based sensing approaches. In addition, the technical complexity of the 3D MIN system, which requires specialized fabrication and optical readout instrumentation, contrasts sharply with emerging minimally invasive ISF sampling technologies that offer simpler designs and continuous monitoring capabilities. Although this study successfully demonstrates sweat biomarker detection, its findings inadvertently underscore the key advantages of ISF: more stable analyte concentrations, direct correlation with blood levels, and access to a broader spectrum of clinically relevant biomarkers, including proteins and cytokines that are often absent or present only in trace amounts in sweat.

Arwani et al. (2024) introduce an innovative wearable platform that utilizes a stretchable ionic‐electronic bilayer hydrogel (ICH‐ECH) for in situ detection of solid‐state epidermal biomarkers (SEBs) such as cholesterol and lactate, thereby eliminating the need for biofluid extraction.^[^ ^374^ ^]^ The authors position SEBs as a promising alternative to conventional sweat‐based monitoring, emphasizing their strong correlation with serum levels (Pearson's r = 0.89–0.95) and the advantages of avoiding the inherent limitations of sweat, including variable secretion rates, dilution, and contamination. However, the methodology inadvertently highlights the comparative weaknesses of sweat as a diagnostic medium. A notable limitation is the hydrogel's operational stability, which is constrained to ≈4 h due to evaporation. Its performance also significantly deteriorates under active perspiration conditions, exhibiting a 38.9% signal drift. Furthermore, while SEBs remove the need for sweat induction, their detection remains dependent on passive diffusion through the stratum corneum, potentially introducing latency and variability akin to the kinetic delays observed with sweat. These limitations underscore unresolved challenges in epidermal biomarker sensing and indirectly confirm the advantages of interstitial fluid (ISF), which typically provides more stable analyte concentrations and reduced susceptibility to environmental influences. A comprehensive clinical quantitative performance comparison of ISF vs. sweat as diagnostic biofluids is tabulated in **Table** **8**.

**Table 8:** A comprehensive clinical quantitative performance comparison of ISF vs. sweat as diagnostic biofluids.

S.No	Biomarker	Biofluid	Reported correlation vs Blood	Quantitative accuracy/Clinical metrics	Lag/Variability	Clinical relevance and notes
1	Glucose	ISF	R^2^ = 0.85‐0.95 in CGM clinical trials	MARD = 9‐13%, >90% in Clarke Error Grid Zone A/B (meets FDA/ISO standards)	≈2–5 min lag	FDA‐ and CE‐approved ISF‐CGM devices (Dexcom G7, Libre 3). Considered clinically validated.
2		Sweat	R^2^ typically 0.3‐0.6, inconsistent	No FDA‐approved sweat glucose device; high intra‐/inter‐individual variability	Highly variable; affected by sweating rate, gland heterogeneity, contamination, skin microbiome	Promising for non‐invasive monitoring, but not yet clinically reliable for systemic glucose.
3	Sodium (Na⁺)	ISF	Deviation ±5‐10% vs plasma	ICC >0.85 in pilot studies	Minor lag (minutes)	Stable compared to sweat; feasible for hydration and electrolyte monitoring.
4		Sweat	Highly variable; deviation often >±20%	Values are strongly influenced by sweat rate and environmental factors	High temporal and inter‐individual variability	Widely used in the sweat chloride test for cystic fibrosis, but systemic reliability for Na⁺ is poor.
5	Potassium (K⁺)	ISF	Deviation ±5‐10% vs plasma	ICC >0.80	Small lag, physiologically stable	Promising for cardiac/renal monitoring; under early validation.
6		Sweat	Very weak correlation; often inconsistent	No reproducible quantitative agreement	Highly variable; easily contaminated	Limited diagnostic value; poor systemic correlation.
7	Chloride (Cl⁻)	ISF	<10% deviation vs plasma	High reproducibility	Minor lag	Potential alternative for cystic fibrosis diagnostics.
8		Sweat	Established diagnostic biomarker	“Sweat chloride test” >60 mmol/L diagnostic for CF	Influenced by age, hydration, and sweat rate	FDA‐approved and the gold standard for CF diagnosis, but it is not suitable for other systemic markers.
9	Lactate	ISF	R^2^ > 0.85 with plasma (athletes & ICU)	<10% deviation; clinically relevant	≈10 min lag reported	Useful in sepsis, hypoxia, and sports physiology.
10		Sweat	Correlation modest (R^2^ ≈0.4‐0.6)	Large variability; dependent on exercise intensity, gland metabolism	Unstable; affected by local tissue metabolism	Limited systemic diagnostic reliability; more useful for sports/fitness monitoring.
11	Cytokines (IL‐6, TNF‐α)	ISF	Detectable at pg/mL levels, comparable to ELISA	LOD for IL‐6 ≈0.3 pg/mL	Limited lag; detection feasible	Early human feasibility; promising for inflammation and infection monitoring.
12		Sweat	Sparse data; low, unstable concentrations	Often below assay detection thresholds	Rapid degradation, instability	Not yet viable for clinical cytokine monitoring.

## 16 Comparative Analysis of the Biofluids: ISF, Sweat, Saliva, Tears, and Urine

The following comparative analysis evaluates the strengths and limitations of the biofluids ISF, sweat, saliva, tears, and urine as sources of biomarkers for medical and clinical applications, as shown in **Table** **9**. Factors such as systemic correlation, variability, sampling methods, and the behavior of these biofluids across different demographics have been evaluated.

**Table 9:** Comparative analysis of body biofluids for medical and clinical applications.

Comparative analysis of the strengths and limitations of biofluids evaluated using different criteria						
S. No	Criterion	ISF	Sweat	Saliva	Tears	Urine
1	Systemic Correlation based on biomarker levels in blood plasma	Strong correlation with blood plasma; biomarkers such as glucose, proteins, and electrolytes accurately reflect systemic conditions.	Partial correlation with systemic physiology; biomarkers such as glucose and lactate can show deviations from corresponding levels in plasma due to diffusion dynamics and external influences.	Moderate correlation with systemic health includes hormones, enzymes, and infection markers.	Shows correlation with systemic markers such as glucose and electrolytes, but is primarily useful for monitoring ocular conditions.	Strong correlation with systemic conditions; renal and metabolic biomarkers such as creatinine and urea accurately reflect systemic conditions.
2	Sampling and Accessibility	Minimally invasive: extraction involves the use of microneedles, ensuring controlled and consistent sampling for continuous monitoring.	Noninvasive: Easily accessible from the skin surface but dependent on sweat production, which may require stimulation using techniques such as iontophoresis.	Noninvasive: collected using swabs or saliva‐collection devices.	Noninvasive: collected via the use of absorbent pads or microfluidic systems.	Noninvasive: easily collected but requires sterile handling for accurate analysis.
3	Biomarker Density	High; closely reflects plasma composition, containing glucose, cytokines, proteins, and drugs, though concentrations can lag behind blood.	Low–moderate; contains electrolytes (Na⁺, Cl⁻, K⁺), lactate, metabolites, but generally lower biomarker concentration than systemic fluids.	Moderate; includes nucleic acids, hormones (cortisol), proteins, pathogens; reflects both local and systemic changes.	Moderate; rich in ocular proteins, cytokines, electrolytes, and stress‐related molecules; niche but informative.	High metabolites, hormones, proteins, and excreted drugs are abundant in urine, often at higher concentrations than in plasma.
4	Sample Volume & Collection	Small volumes; collected via microneedles or reverse iontophoresis; minimally invasive	Collection is non‐invasive, but sweat rate is highly variable and requires stimulation (exercise, iontophoresis).	Easy, stress‐free collection; sufficient volumes obtainable non‐invasively.	Small volumes require capillary tubes, micro‐sponges, or Schirmer strips; minimally invasive.	Large volumes readily available; non‐invasive and repeatable.
5	Biomarker Concentrations	Concentrations of biomarkers are close to those in plasma, simplifying detection and improving reliability for clinical use.	Lower concentrations of systemic biomarkers, such as glucose and lactate, compared to those in plasma; dilution effects complicate detection.	Moderate concentrations of enzymes, hormones, and specific metabolites.	Moderate concentrations of markers such as lactoferrin, glucose, and electrolytes.	High concentrations of metabolic and renal biomarkers enable ease of detection.
6	Biomarker Range	A broad range of biomarkers, including glucose, proteins, cytokines, hormones, and nucleic acids, is suitable for complex diagnostics.	Limited diversity; focus on electrolytes, lactate, cortisol, and hydration biomarkers.	Moderate range; focus on oral health and systemic conditions.	Limited range; focus on electrolytes and proteins related to ocular health.	Broad range; includes toxins, metabolites, electrolytes, and proteins.
7	Contamination Risks	Moderate; contamination from skin flora or improper handling can alter composition.	High skin surface contaminants, lotions, and environmental exposure easily interfere with sample quality.	Moderate; susceptible to oral microbiota, food debris, and variable pH.	Moderate; potential contamination from ocular surface debris or environmental exposure.	Low‐moderate risk of bacterial growth during storage if not processed rapidly.
8	Environmental Factors, Including Temperature and Humidity	Minimally influenced by environmental conditions, composition depends on physiological processes, ensuring biofluid stability.	Highly sensitive to temperature, humidity, and physical activity, causing variations in analyte concentrations.	Sensitive to changes in pH, contamination, and degradation of saliva.	Sensitive to evaporation, irritation, and environmental contamination.	Minimal environmental influence; stable with proper handling and storage.
9	Region‐based Variability (Forehead, hands, and neck)	Minimal regional variability due to uniform composition reduces the need for region‐based adjustments in diagnostics.	Significant variability across body regions, such as palms, soles, and axilla, requires site‐specific calibration for accuracy.	Minimal variability; uniformly secreted across salivary glands.	Minimal variability; consistent across tear glands.	No regional variability; reflects systemic kidney and metabolic functions.
10	Intra‐ and Inter‐Person Variability	Low variability: ISF biomarkers are regulated by homeostasis, ensuring consistency within and across individuals.	High variability in sweat rate and composition due to age, gender, fitness level, and hydration status.	Moderate variability due to hydration status, diet, and individual health conditions.	Varies with hydration, diet, health, and emotional conditions.	Moderate variability due to hydration status, diet, and renal functioning.
11	Gender‐based Differences	Minimal gender‐based differences; physiological and hormonal conditions primarily influence levels of systemic biomarkers.	Sweat rates and composition differ between the genders. Males typically produce higher sweat volumes, leading to analyte dilution, whereas females may exhibit higher concentrations of biomarkers due to lower sweat rates.	Salivary hormones such as cortisol and enzyme levels may exhibit gender‐based differences influenced by hormonal cycles.	Varies due to hormonal factors, particularly in females during menstruation or menopause.	Minimal gender‐based differences; biomarkers primarily reflect systemic metabolic functions.
12	Challenges in Gender‐based Calibration	Minimal calibration differences between genders; ISF biomarker levels are independent of sex‐specific differences in volume.	Higher sweat volumes in males necessitate different calibration thresholds for devices. Lower sweat volumes in females may lead to false‐positive results if thresholds are not adjusted.	Salivary hormones such as cortisol and enzyme levels may require gender‐specific adjustments due to hormonal cycles.	Minor gender‐based differences in tear production are linked to hormonal influences, particularly in females.	Minimal gender‐based calibration challenges due to systemic consistency.
13	Athletic‐Training Considerations	ISF provides consistent data on systemic biomarkers during physical exertion, unaffected by changes in sweat rate or environmental conditions.	Sweat variability during intense training complicates systemic diagnostic applications; monitoring of hydration status is more reliable.	Limited use in athletics; can monitor hydration status and stress‐related biomarkers.	Useful for hydration‐related diagnostics in athletes, but limited applications for monitoring systemic health.	Not suitable for real‐time monitoring during athletic training but reflects the cumulative effects of activity and hydration status.
14	Athletic Performance and Training	ISF provides reliable data on systemic biomarkers such as glucose as well as electrolyte balance during and after physical activity, unaffected by changes in sweat rate.	Sweat volume increases during athletic training, resulting in dilution of markers such as Na^+^ ions and glucose. Electrolyte loss is significant but variable among individuals.	Limited athletic applications; used for monitoring hydration status and stress‐related markers.	Useful for hydration‐related diagnostics in athletes but limited systemic diagnostic applications.	Unsuitable for real‐time monitoring in athletes but reflects the cumulative effects of activity and hydration status.
15	Applications for Critically Ill Patients	ISF offers reliable access to systemic biomarkers even in critically ill patients, enabling effective monitoring and intervention.	Sweat production is often reduced or erratic in critically ill patients, limiting its utility for monitoring systemic health markers.	Saliva is moderately reliable but prone to contamination and degradation in acute‐care settings.	Tear diagnostics have limited use in critical care due to their localized applications and lower systemic correlation.	Reliable for monitoring metabolic and renal health in critically ill patients.
16	Applications for Acutely Ill Patients	ISF remains accessible and stable even in patients with acute conditions, making it a highly reliable medium for monitoring critical biomarkers such as electrolytes and glucose.	Sweat production may be limited or erratic in acute conditions such as fever, dehydration, or shock, complicating diagnostic applications and limiting accuracy.	Moderately useful; prone to contamination and degradation in acute care.	Limited applicability in acute‐care; primarily localized diagnostics.	Highly reliable for assessing metabolic and renal health in acute conditions.
17	Temporal Stability	Continuously present in interstitial spaces, offering reliable real‐time monitoring for systemic biomarkers.	Episodic generation dependent on activity or stimulation; lacks consistency for continuous monitoring of biomarkers.	Saliva is degraded quickly and prone to contamination, limiting its temporal stability.	Episodic production, evaporation, and secretion variability reduce temporal stability.	Stable overtime reflects cumulative systemic biomarkers rather than real‐time changes.
18	Diagnostic Applications	Ideal for systemic diagnostic applications, including monitoring glucose levels in diabetes, inflammatory markers, and therapeutic drugs.	Effective for localized diagnostic applications such as monitoring hydration status, stress, and fitness level.	Suitable for evaluating oral health, hormones, and incidence of infection.	Best for ocular health diagnostics, including dry eye syndrome and conjunctivitis.	Well‐suited for evaluation of metabolic, renal, and hydration status.
19	Reliability and Consistency	Stable and reliable; minimally affected by transient conditions, making it suitable for critical clinical applications.	Susceptible to variations due to environmental and physiological factors; less reliable for clinical diagnostic applications.	Moderate reliability; prone to contamination and variability.	Moderate reliability: evaporation and irritants influence readings.	Highly reliable for systemic health assessments.
20	Device Integration	Emerging integration with wearable microneedle sensors; superior for precision diagnostics in chronic and critical conditions.	Widely used in commercially available wearable sensors for hydration, fitness, and stress, calibration issues occur.	Integration into sensors for monitoring oral health and metabolism; growing adoption in consumer healthcare devices.	Integration into sensors for monitoring ocular health; limited systemic applications.	Emergence of primarily laboratory‐based, portable analyzers for home and field use.
21	Ease of Use	Microneedle‐based extraction requires slight skin penetration but offers passive, continuous, and reliable data collection.	Easily collected but in a manner dependent on active stimulation or natural sweating, limiting its use in applications that require passive monitoring.	Simple, painless collection using swabs or spit samples; requires minimal expertise.	Noninvasive and simple collection; evaporation risks can complicate manual handling.	Easy collection; sterile techniques are essential for diagnostic accuracy.
22	Calibration Complexity	Minimal calibration is required due to stable composition and close correlation of biomarker concentrations with those in blood plasma, reducing error potential.	Requires complex algorithms to address variability and dilution effects, increasing the potential for measurement errors.	Moderate; sensitive to degradation and contamination of saliva.	Moderate; evaporation and secretion variability require calibration adjustments.	Low; biomarker stability minimizes the need for complex calibration.
23	Cost and Scalability	Initial costs for microneedle‐based systems are higher, but ongoing technological advancements have improved scalability.	Low‐cost sweat sensors are scalable for wellness and fitness applications but pose challenges for clinical use.	Moderate cost; scalable for diagnostic applications for oral health and infection.	Moderate cost; scalable primarily for ocular health diagnostics.	Low cost and highly scalable; well‐established in clinical workflows.
24	Challenges Associated with Wearable Design	Demands biocompatible microneedle arrays and fluidics but offers consistent analyte capture and systemic accuracy.	Requires sweat stimulation techniques and sophisticated surface integration for consistent collection.	Easily integrated into sensors for oral monitoring; potential for portable applications.	Requires specialized designs to prevent evaporation and contamination.	Limited wearable potential; remains primarily lab‐based.
25	Clinical Validation	Strong potential for clinical validation due to the alignment of biomarker levels in ISF with those in plasma; ideal for monitoring chronic disease and critical clinical care applications.	Limited validation for systemic diagnostics; sweat‐based devices are primarily used for monitoring fitness and wellness.	Moderate validation; focus on monitoring oral health and salivary hormones.	Validated for ocular health diagnostics but lacks systemic validation.	Extensive clinical validation; commonly used for diagnostics based on metabolism and renal health.
26	Technological Development	Rapidly advancing microneedle technology is revolutionizing ISF sampling for real‐time clinical diagnostics.	Established for monitoring fitness and hydration status; limited in systemic clinical diagnostics due to variability.	Moderately developing; integrated into devices for oral health diagnostics and limited systemic diagnostic applications.	Emerging for ocular health diagnostics; less developed for systemic applications.	Fully developed; widely used in clinical settings with emerging portable analyzers.
27	Clinical Applicability	Strong candidate for wearable monitoring devices; useful for glucose monitoring, drug pharmacokinetics, and inflammatory markers.	Limited systemic use; primarily applied for hydration, electrolyte balance, and cystic fibrosis testing (Cl⁻).	Applied in stress monitoring, infectious disease detection (HIV), and drug testing.	Used in ophthalmology (dry eye disease, diabetic retinopathy) and systemic stress marker evaluation.	Routine is used for renal function, pregnancy, drug screening, and metabolic disorders.
28	Advantages	Minimally invasive access to plasma‐equivalent biomarkers; potential for continuous, wearable diagnostics.	Easily accessible; ideal for wearable sensors; real‐time monitoring feasible.	Convenient, painless, stress‐free collection; good for population‐level screening.	Rich in eye‐related biomarkers; potential for early detection of systemic stress.	Large volumes, stable biomarker content; longitudinal sampling feasible.
29	Limitations	Low sample yield, lag in biomarker equilibration with blood, and limited standardized clinical assays.	High inter‐ and intra‐individual variability; influenced by environmental factors (temperature, humidity, hydration).	Lower biomarker density than blood or ISF; strong influence of oral hygiene and circadian cycles.	Limited sample volume; technically difficult collection; low yield.	May not reflect dynamic systemic physiology due to delayed clearance.
30	Maturity Level	Medium–High; rapidly growing in wearable diagnostics and continuous monitoring.	Medium: experimental stage, limited clinical deployment.	Medium–High; FDA‐approved kits exist (hormone assays, viral tests).	Medium: research stage, growing translational interest.	High; well‐established and routinely applied in clinical diagnostics.

## 17 Challenges and Future Directions in Clinical Diagnostics

The rapid advancements in biofluid‐based diagnostics offer opportunities for minimally invasive, point‐of‐care, and continuous health monitoring. However, significant challenges still exist across various biofluids. Identifying and categorizing these challenges is crucial for transitioning these technologies from proof‐of‐concept devices to clinically validated diagnostic tools.

### 17.1 Standardization and Validation

The composition of biofluids is variable and influenced by various factors, as clearly discussed in the earlier section. For example, electrolyte concentrations in sweat can change with temperature and hydration, while saliva biomarkers are affected by oral microbiota and food intake. The variability in interstitial fluid composition is impacted by skin permeability and collection depth.^[^ ^375^ ^]^ The absence of standardized protocols for sampling, storage, and processing hampers data reproducibility across laboratories. Emerging technologies, such as microneedle‐based extraction, hydrogel sweat patches, and microfluidic sensors, show promise but require validation against blood‐based standards. Multi‐center clinical studies are also needed to establish baseline ranges, determine correction factors for physiological variability, and ensure compliance with regulatory standards. Standardization is essential for improving comparability between studies and facilitating regulatory approval.^[^ ^376^, ^377^ ^]^

### 17.2 Multiplexing Strategies

Most current diagnostic devices target single analytes, such as glucose in ISF, cortisol in saliva, and chloride ions in sweat for cystic fibrosis. However, diseases are rarely defined by just one biomarker, making multiplexed detection essential for clinical accuracy. Emerging platforms like lab‐on‐chip (LOC), nanostructured biosensors, and laser‐induced multiplex graphene electrodes now allow for the parallel detection of proteins, nucleic acids, and metabolites from tiny volumes of biofluids.^[^ ^378^, ^379^ ^]^

Examples include:

1. Blood assays are increasingly utilizing microfluidic immunoarrays for cancer biomarker detection panels.
2. Minimal‐invasive Microneedle arrays designed for interstitial fluid, combined with nanomaterial sensors, allow for simultaneous monitoring of glucose, lactate, and cytokines.
3. Sweat patches are being developed with multiplexed electrochemical sensors capable of detecting electrolytes and metabolites.
4. Contact lenses equipped with embedded tear sensors can simultaneously monitor glucose levels and stress biomarkers.

Future efforts should focus on enhancing signal‐to‐noise ratios in low‐concentration fluids, such as sweat and tears, while developing multiplexed point‐of‐care diagnostics that better reflect the complexity of diseases.^[^ ^380^ ^]^

### 17.3 Integration into Clinical Workflow

The transition from benchtop prototypes to clinical adoption represents one of the most significant bottlenecks in healthcare. Blood remains the dominant biofluid because of its deep integration into existing healthcare infrastructure. In contrast, emerging biofluids leave physicians uncertain about their systemic relevance, assay reliability, and regulatory acceptance.^[^ ^381^ ^]^

To overcome these challenges, technologies must demonstrate clinical utility, cost‐effectiveness, and compatibility with existing workflows. Examples include:

1. ISF microneedle devices and wearable sweat biosensors that require robust clinical trial data to convince practitioners of their reliability and interpretability.
2. Saliva‐based assays, which are already integrated into oral swab kits used for infectious disease testing (HIV, SARS‐CoV‐2).
3. Urine dipstick tests, which are fully embedded in hospital workflows for assessing renal and metabolic diseases.
4. Tear‐based diagnostics, which remain largely niche and need to expand beyond ophthalmology into broader systemic health monitoring.

Integration requires compatibility with electronic health record (EHR) systems, AI‐driven analytics, and telemedicine platforms. This compatibility ensures that diagnostics utilizing biofluids can deliver actionable insights without disrupting existing care models.^[^ ^382^, ^383^, ^384^ ^]^

### 17.4 Longitudinal Monitoring Potential

Unlike traditional blood tests that provide static data at a single point in time, non‐invasive biofluids such as interstitial fluid (ISF), sweat, saliva, and tears present unique opportunities for continuous and longitudinal monitoring.^[^ ^16^, ^24^ ^]^ Various wearable technologies, ranging from minimal‐invasive microneedle patches to smart contact lenses and epidermal sweat sensors, are being developed to capture real‐time biomarker dynamics. This continuous stream of data has the potential to transform healthcare by enabling more personalized and timely medical interventions.^[^ ^385^ ^]^

Examples include:

1. Early disease detection, such as identifying pre‐diabetic glucose dysregulation from ISF.
2. Dynamic treatment monitoring, including tracking chemotherapy drug levels in ISF and hormonal cycles from saliva.
3. Personalized precision medicine, like observing electrolyte fluctuations in athletes via sweat.

Several challenges persist, including sensor calibration, biofouling, drift, power consumption, and user compliance, all of which hinder long‐term deployment. Integrating AI/ML models for trend analysis, anomaly detection, and predictive health outcomes will be essential for deriving meaningful clinical insights from continuous biofluid management.^[^ ^386^ ^]^

## 18 Technologies Innovation and Integration

Future of biomedical diagnostics by focusing on wearable and mobile devices, smartphone‐based diagnostics, the integration of artificial intelligence and machine learning (AI/ML), and miniaturized sensors. Together, these complementary technologies represent a significant shift from centralized, laboratory‐dependent testing to continuous, decentralized, and patient‐centered monitoring systems.

### 18.1 Wearable and Mobile Diagnostics

Wearable devices, such as microneedle patches, sweat analyzers, and flexible electronic skin patches, allow for minimally invasive or non‐invasive access to relevant physiological biofluids (ISF, sweat, saliva, tears, etc.). By enabling continuous and longitudinal tracking of biomarkers, these devices address the episodic nature of conventional testing and offer insights into dynamic physiological changes. Mobile diagnostics, especially when combined with wireless data transmission and cloud connectivity, can be integrated into broader healthcare ecosystems, supporting remote monitoring and telemedicine.^[^ ^16^, ^24^, ^387^ ^]^

### 18.2 Smartphone‐Based Diagnostics

Smartphone‐based diagnostics enhance accessibility by transforming mobile phones into portable diagnostic hubs. Built‐in sensors and custom‐designed attachments, such as lens‐free microscopes, microfluidic chips, or electrochemical sensing units, facilitate the detection and quantification of a wide range of biomarkers at the point of care. The widespread use of smartphones allows for scalable deployment even in resource‐limited settings, democratizing access to diagnostics while reducing costs. Additionally, smartphones serve as real‐time interfaces for signal processing, visualization, and patient feedback, while also enabling secure data transmission to healthcare professionals.^[^ ^388^ ^]^

### 18.3 Integration of Artificial Intelligence and Machine Learning (AI/ML)

Nowadays, AI and ML play a crucial role in enhancing the diagnostic reliability of wearable and smartphone‐based systems. These algorithms help correct for motion artifacts, environmental fluctuations, and inter‐patient variability, improving data accuracy. In addition to data refinement, AI/ML models can identify subtle, disease‐specific patterns across continuous data streams, enabling predictive diagnostics, for instance, forecasting glucose levels, detecting arrhythmia, or monitoring stress‐related cortisol dynamics. When integrated with cloud‐based platforms, AI/ML provides adaptive, personalized insights and supports early intervention strategies. Importantly, these capabilities allow diagnostics to evolve from descriptive measurements to predictive and prescriptive healthcare solutions.^[^ ^389^, ^390^, ^391^, ^392^, ^393^ ^]^

However, recent studies in cancer diagnosis emphasize the importance of protecting sensitive patient data, while still enabling effective AI analysis. Malin and Goodman et al. outlined several strategies to mitigate privacy risks without compromising data utility.^[^ ^394^, ^395^ ^]^ These include consent frameworks, privacy risk assessments, cryptographic methods for secure querying, and game‐theoretic approaches for data sharing. Moreover, it is crucial to address algorithmic bias by using diverse datasets and involving experts, as this helps prevent the worsening of existing health disparities. As for power constraints, large AI models require significant computational resources and energy, which can impose pressure on current infrastructure and raise sustainability concerns. Research indicates that training advanced AI models can consume energy equivalent to that of entire countries, highlighting the urgent need for more efficient algorithms and the adoption of renewable energy sources. In brief, addressing these privacy and power considerations is essential for ensuring the responsible, equitable, and sustainable deployment of AI in healthcare and other fields.^[^ ^396^, ^397^ ^]^

### 18.4 Miniaturized Sensors

Recent advances in microfabrication, nanostructured functional materials, and microfluidics have led to the development of highly miniaturized sensors that offer exceptional sensitivity and specificity. These sensors can operate with microliter to nanoliter‐scale sample volumes and can be seamlessly integrated into wearable patches, smartphone‐based accessories, or implantable systems. Their multiplexing capabilities allow for the simultaneous detection of multiple biomarkers, while flexible and biocompatible substrates ensure reliable operation under real‐world conditions, including motion and environmental variability. This miniaturization enhances portability and facilitates rapid, low‐power, and real‐time analysis, making these sensors indispensable for continuous monitoring platforms.^[^ ^398^, ^399^, ^400^ ^]^

Overall, the combination of wearable/mobile devices, smartphone‐based diagnostics, AI/ML integration, and miniaturized sensors creates a convergent diagnostic ecosystem. This integration enables continuous physiological mapping, intelligent data interpretation, and patient‐centered healthcare solutions. Importantly, these platforms align with the goals of precision and preventive medicine, transitioning diagnostics from reactive, clinic‐centered approaches to proactive, decentralized, and globally scalable healthcare delivery.

## 19 Conclusion and Future Perspectives

The use of ISF in minimal‐invasive diagnostic applications presents clear advantages over that of other available biofluids (sweat and saliva) in non‐invasive diagnostic applications, owing to the close correlation of biomarker levels in ISF with those in blood plasma (see Table 3). ISF is composed of a rich array of systemic biomarkers such as glucose, proteins, electrolytes, and cytokines, enabling comprehensive health monitoring. While sweat has served as an accessible and practical biofluid for monitoring hydration status and stress, its susceptibility to environmental and physiological variations makes it less reliable for applications requiring evaluations of systemic biomarkers. The integration of microneedle technology has unlocked the full potential of various ISF‐based techniques by providing painless and efficient method of ISF extraction and enabling continuous, real‐time monitoring by ISF‐based wearable sensors. These advancements in ISF‐based diagnostic devices pave the way for the transformation of healthcare, particularly in areas such as the management of chronic diseases, monitoring fitness levels, and optimizing therapeutic drugs. Furthermore, the use of ISF offers opportunities for dual‐function devices that combine diagnostics with targeted drug delivery, enhancing the precision and effectiveness of personalized medicine.

Despite these advances, key challenges remain in ISF‐based diagnostics, including standardizing measurements of ISF‐based biomarkers, addressing interindividual variability, and developing cost‐effective production processes essential for scalability. Clinical validation via rigorous clinical trials and streamlined regulatory pathways is also critical for establishing ISF‐based diagnostics as a reliable alternative to more invasive approaches.

### 19.1 Future Perspectives

The use of ISF‐based diagnostics in conjunction with artificial intelligence and digital health platforms holds remarkable potential for redefining personalized healthcare. ISF‐based biosensors enable real‐time, continuous monitoring and timely interventions, empowering patients as well as healthcare providers and facilitating better outcomes. With ongoing advancements in wearable technology and microneedle systems, ISF‐based devices are poised to lead the next generation of noninvasive, accurate, and accessible diagnostic solutions. The strategic future perspectives for diagnostics on ISF and their expected clinical relevance are tabulated in **Table** **10**.

**Table 10:** Strategic future perspectives for diagnostics based on ISF and their expected clinical relevance.

S.No	Prioritization	Future perspective	Key benefit	Expected clinical relevance
1	Advancement in Microneedle‐based ISF Extraction	Refinement of hydrogel‐based and hollow microneedles	Increased ISF yield while preserving biocompatibility and user comfort	Reliable, repeatable sampling for both clinical and wearable applications
2		Development of biodegradable microneedles for single‐use	Eliminates risk of reuse and simplifies disposal	Enhanced safety and scalability for population screening and diagnostic
3		Integration of self‐healing materials	Maintains functionality despite micro‐damage	Extended device lifespan and reduced replacement frequency
4		Adoption of advanced manufacturing (3D bioprinting)	High‐precision, reproducible microneedle fabrication	Large‐scale, consistent quality production
5	Expansion of Biomarker Detection in ISF	Comprehensive biomarker panels (Proteins, cytokines, nucleic acids, drugs)	Broader diagnostic capability beyond glucose	Application in neurodegenerative, oncological, metabolic, and psychiatric disorders
6		Real‐time monitoring of less‐studied biomarkers (Neuropeptides, inflammatory mediators)	Novel targets for continuous monitoring	Early detection and intervention in inflammation and neurological disease
7	Hybrid Biofluid Monitoring Platforms	Integrated sweat–ISF devices	Simultaneous systemic and localized biomarker assessment	Complete health profiling for sports performance, chronic disease management, and workplace health monitoring.
8	Standardization and Calibration	Establishment of plasma–ISF correlation standards	Defined biomarker reference ranges and conversion models	Improved clinical interpretability and standard‐of‐care integration
9		Advanced calibration protocols	Accounts for inter‐ and intra‐individual variability	Accurate and reproducible results across diverse populations
10	Material Innovation for Wearable Devices	Use of lightweight, durable, biocompatible materials (PI, PDMS, TPEs)	Enhanced comfort and long‐term skin compatibility	Increased user compliance and continuous monitoring
11		Integration of flexible/stretchable electronics	Maintained performance during dynamic activity	Expanded usability in active and occupational settings
12	Clinical Validation and Regulatory Pathways	Rigorous clinical validation trials	High‐quality evidence against gold‐standard blood tests	Strengthened regulatory submissions and clinical adoption
13		Regulatory framework adaptation for microneedle diagnostics	Streamlined approval processes	Accelerated time‐to‐market
14	Digital Health Integration	Cloud‐based and AI‐driven analytics	Predictive health insights from continuous data streams	Real‐time alerts, risk prediction, and personalized health management
15		Closed‐loop therapeutic integration (CGM + insulin delivery)	Automated link between diagnosis and treatment	Improved outcomes in chronic disease management
16	Therapeutic–Diagnostic Synergy (Theranostics)	Dual‐function devices for ISF monitoring and drug delivery	Simultaneous diagnosis and therapy	Personalized, on‐demand intervention
17	Scalability and Accessibility	Scalable, cost‐optimized manufacturing	Affordable production for diverse markets	Expanded access, including resource‐limited settings
18		Collaboration between academia, industry, and regulators	Alignment of innovation with compliance and feasibility	Reduced barriers to clinical translation
19	Complementary Use of Sweat and ISF	Dual‐biofluid analysis approach	Leverages the strengths of each biofluid	Versatile applications across multiple health contexts
20	Digital Health and AI/ML Integration	Cloud‐based and AI‐driven analytics	Predictive health insights from continuous data streams	Real‐time alerts, risk prediction, and personalized health management
21		AI/ML‐enabled data processing	Automated noise reduction, lag‐time correction, and multi‐parametric analysis	Enhanced accuracy, improved trend detection, and adaptive therapy recommendations
22		Closed‐loop therapeutic integration (CGM + insulin delivery)	Automated link between diagnosis and treatment	Improved outcomes in chronic disease management

ISF‐based devices, supported by revolutions in the minimally invasive microneedle‐based technology and wearable biosensors, are poised to redefine the landscape of minimally invasive diagnostic applications. Addressing current challenges associated with standardization, device integration, and accessibility of ISF‐based monitoring technologies can enable them to surpass the technologies based on conventional methods, showcasing the transformative potential of the former in personalized and preventive healthcare. With the ability of ISF to mirror systemic biomarker profiles and support therapeutic interventions, ISF‐based devices represent the future of noninvasive diagnostics, empowering patients and healthcare providers alike to achieve better patient outcomes.

## Conflict of Interest

The authors declared that they do not have any conflicts of interest.

## References

[1] M. Mascini , S. Tombelli , Biomarkers 2008, 13, 637. 19061054 10.1080/13547500802645905

[2] A. J. Bandodkar , W. J. Jeang , R. Ghaffari , J. A. Rogers , Annu. Rev. Anal. Chem. 2019, 12, 1. 10.1146/annurev-anchem-061318-114910 30786214

[3] S. K. Vashist , P. B. Luppa , L. Y. Yeo , A. Ozcan , J. H. T. Luong , Trends Biotechnol. 2015, 33, 692. 26463722 10.1016/j.tibtech.2015.09.001

[4] I. H. Mahardika , S. Naorungroj , W. Khamcharoen , S. Kin , N. Rodthongkum , O. Chailapakul , K. Shin , Adv. NanoBiomed Res. 2023, 3, 2300058.

[5] C. P. Price , Br. Med. J. 2001, 322, 1285.11375233

[6] C. Dincer , R. Bruch , A. Kling , P. S. Dittrich , G. A. Urban , Trends Biotechnol. 2017, 35, 728. 28456344 10.1016/j.tibtech.2017.03.013 PMC5538621

[7] Grand View Research , Protein Supplements Market Size, Share & Trends Analysis Report, Grand View Research, San Francisco, CA 2022.

[8] L. Laursen , Nat. Med. 2012, 18, 1156. 22869167 10.1038/nm0812-1156

[9] L. Lipani , B. G. R. Dupont , F. Doungmene , F. Marken , R. M. Tyrrell , R. H. Guy , A. Ilie , Nat. Nanotechnol. 2018, 13, 504. 29632401 10.1038/s41565-018-0112-4

[10] S. Saba Raoof , M. A. S. Durai , Contrast Media Mol. Imaging 2022, 1, 4822235. 10.1155/2022/4822235 PMC9536991 36247859

[11] D. W. Kim , M. Eala , G. Lee , M. B. Lam , N. Martin , B. Nakfoor , A. Dicker , Transl. Radiat. Oncol. Academic Press 2023, 551.

[12] J. L. J. M. Müskens , R. B. Kool , S. A. van Dulmen , G. P. Westert , BMJ Qual. Saf. 2022, 31, 54. 10.1136/bmjqs-2020-012576 PMC8685650 33972387

[13] H. C. Ates , A. Brunauer , F. von Stetten , G. A. Urban , F. Güder , A. Merkoçi , S. M. Früh , C. Dincer , Adv. Funct. Mater. 2021, 31, 2010388.

[14] L. Stendelyte , M. Malinauskas , D. E. Grinkeviciute , L. Jankauskaite , Diagnostics 2023, 13, 1929. 37296781 10.3390/diagnostics13111929 PMC10252322

[15] T. W. Pittman , D. B. Decsi , C. Punyadeera , C. S. Henry , Theranostics 2023, 13, 1091. 36793864 10.7150/thno.78872 PMC9925318

[16] J. Xu , Y. Fang , J. Chen , Biosensors 2021, 11, 245. 34436047 10.3390/bios11080245 PMC8391966

[17] K. M. Saifullah , Z. Faraji Rad , Adv. Mater. Interfaces 2023, 10, 2201763.

[18] J. Kim , J. R. Sempionatto , S. Imani , M. C. Hartel , A. Barfidokht , G. Tang , A. S. Campbell , P. P. Mercier , J. Wang , Adv. Sci. 2018, 5, 1800880. 10.1002/advs.201800880 PMC6193173 30356971

[19] A. Veronica , Y. Li , Y. Li , I. M. Hsing , H. Y. Y. Nyein , Sen. Diagn. 2023, 2, 1335.

[20] L. Qiao , M. R. Benzigar , J. A. Subramony , N. H. Lovell , G. Liu , ACS Appl. Mater. Interfaces 2020, 12, 34337. 32579332 10.1021/acsami.0c07614

[21] S. Emaminejad , S. Pilehvar , A. J. Wilhelm , A. J. Wilhelm , K. King , 2018, 10639, 231.

[22] Y. Song , D. Mukasa , H. Zhang , W. Gao , Accounts Mater. Res. 2021, 2, 184.

[23] N. Davis , J. Heikenfeld , C. Milla , A. Javey , Nat. Biotechnol. 2024, 42, 860. 38212492 10.1038/s41587-023-02059-1

[24] F. Gao , C. Liu , L. Zhang , T. Liu , Z. Wang , Z. Song , H. Cai , Z. Fang , J. Chen , J. Wang , M. Han , J. Wang , K. Lin , R. Wang , M. Li , Q. Mei , X. Ma , S. Liang , G. Gou , N. Xue , Microsystems Nanoeng 2023, 9, 1(2023). 10.1038/s41378-022-00443-6 PMC9805458 36597511

[25] J. Kim , A. S. Campbell , B. E. F. de Ávila , J. Wang , Nat. Biotechnol. 2019, 37, 389. 30804534 10.1038/s41587-019-0045-y PMC8183422

[26] Y. Yang , W. Gao , Chem. Soc. Rev. 2019, 48, 1465. 29611861 10.1039/c7cs00730b

[27] H. Yu , J. Sun , Nanotechnol. Precis. Eng. 2020, 3, 126. 10.1016/j.npe.2019.12.001 PMC8006565 33786424

[28] P. Kumar , S. Gupta , B. C. Das , Transl. Oncol. 2024, 40, 101827. 38042138 10.1016/j.tranon.2023.101827 PMC10701368

[29] C. Z. Zhang , X. Q. Cheng , J. Y. Li , P. Zhang , P. Yi , X. Xu , X. D. Zhou , Int. J. Oral Sci. 2016, 8, 133. 27585820 10.1038/ijos.2016.38 PMC5113094

[30] Y. H. Lee , D. T. Wong , Am. J. Dent. 2009, 22, 241.19824562

[31] U. Subbiah , H. V. Subbiah , K. Sumathi , S. S. Lalitha , J. Angiother. 2021, 5, 2152.

[32] S. Kumari , M. Samara , R. Ampadi Ramachandran , S. Gosh , H. George , R. Wang , R. P. Pesavento , M. T. Mathew , Biomed. Mater. Devices 2024, 2, 121. 10.1007/s44174-023-00090-z PMC10243891 37363139

[33] P. Dongiovanni , M. Meroni , S. Casati , R. Goldoni , D. V. Thomaz , N. S. Kehr , D. Galimberti , M. Del Fabbro , G. M. Tartaglia , Int. J. Oral Sci. 2023, 15, 27. 37386003 10.1038/s41368-023-00231-6 PMC10310701

[34] P. Ravishankar , A. Daily , Appl. Sci. 2022, 12, 2884.

[35] G. Backiyalakshmi , U. Snekhalatha , A. L. Salvador , Anal. Biochem. 2024, 692, 115578. 38801938 10.1016/j.ab.2024.115578

[36] N. Balhara , M. Devi , A. Balda , M. Phour , A. Giri , URINE 2023, 5, 40.

[37] I. Sarosiek , R. Schicho , P. Blandon , M. Bashashati , World J. Gastrointest. Oncol. 2016, 8, 459. 27190585 10.4251/wjgo.v8.i5.459 PMC4865713

[38] D. Li , L. Yan , F. Lin , X. Yuan , X. Yang , X. Yang , L. Wei , Y. Yang , Y. Lu , J. Gastric Cancer 2022, 22, 306. 36316107 10.5230/jgc.2022.22.e28 PMC9633929

[39] C. Zhang , W. Leng , C. Sun , T. Lu , Z. Chen , X. Men , Y. Wang , G. Wang , B. Zhen , J. Qin , EBioMedicine 2018, 30, 120. 29576497 10.1016/j.ebiom.2018.03.009 PMC5952250

[40] M. Friedel , I. A. P. Thompson , G. Kasting , R. Polsky , D. Cunningham , H. T. Soh , J. Heikenfeld , Nat. Biomed. Eng. 2023, 7, 1541. 36658344 10.1038/s41551-022-00998-9

[41] A. Oharazawa , G. Maimaituxun , K. Watanabe , T. Nishiyasu , N. Fujii , J. Dermatol. Sci. 2024, 114, 141. 38740531 10.1016/j.jdermsci.2024.04.001

[42] P. P. Samant , M. M. Niedzwiecki , N. Raviele , V. Tran , J. Mena‐Lapaix , D. I. Walker , E. I. Felner , D. P. Jones , G. W. Miller , M. R. Prausnitz , Sci. Transl. Med. 2020, 12, eaaw0285. 33239384 10.1126/scitranslmed.aaw0285 PMC7871333

[43] Z. Wu , Z. Qiao , S. Chen , S. Fan , Y. Liu , J. Qi , C. T. Lim , Commun. Mater. 2024, 5, 33.

[44] J. Madden , C. O'Mahony , M. Thompson , A. O'Riordan , P. Galvin , Sens. Bio‐Sensing Res. 2020, 29, 100348.

[45] R. Amarnani , P. Shende , Biomed. Microdevices 2022, 24, 4. 10.1007/s10544-021-00604-w PMC8651504 34878589

[46] M. R. Babu , S. Vishwas , R. Khursheed , V. Harish , A. B. Sravani , F. Khan , B. Alotaibi , A. Binshaya , J. Disouza , P. S. Kumbhar , V. Patravale , G. Gupta , R. Loebenberg , M. F. Arshad , A. Patel , S. Patel , K. Dua , S. K. Singh , Drug Deliv. Transl. Res. 2024, 14, 1393. 38036849 10.1007/s13346-023-01475-9

[47] M. Garg , N. Jain , S. Kaul , V. K. Rai , U. Nagaich , Microchim. Acta 2023, 190, 301. 10.1007/s00604-023-05859-z 37464230

[48] J. Halder , S. Gupta , R. Kumari , G. D. Gupta , V. K. Rai , J. Pharm. Innov. 2021, 16, 558. 32837607 10.1007/s12247-020-09460-2 PMC7276250

[49] K. Aich , T. Singh , S. Dang , Drug Deliv. Transl. Res. 2022, 12, 1556. 34564827 10.1007/s13346-021-01056-8

[50] S. H. Bariya , M. C. Gohel , T. A. Mehta , O. P. Sharma , J. Pharm. Pharmacol. 2012, 64, 11. 22150668 10.1111/j.2042-7158.2011.01369.x

[51] R. Parhi , J. Drug Deliv. Sci. Technol. 2022, 75, 103639.

[52] H. Sun , Y. Zheng , G. Shi , H. Haick , M. Zhang , Small 2023, 19, 2207539. 10.1002/smll.202207539 36950771

[53] P. Dardano , I. Rea , L. De Stefano , Curr. Opin. Electrochem. 2019, 17, 121.

[54] D. Kulkarni , D. Gadade , N. Chapaitkar , S. Shelke , S. Pekamwar , R. Aher , A. Ahire , M. Avhale , R. Badgule , R. Bansode , B. Bobade , Sci. Pharm. 2023, 91, 27.

[55] L. Wang , Y. Wang , X. Wu , P. Wang , X. Luo , S. Lv , Microchim. Acta 2024, 191, 88.

[56] O. Heifler , E. Borberg , N. Harpak , M. Zverzhinetsky , V. Krivitsky , I. Gabriel , V. Fourman , D. Sherman , F. Patolsky , ACS Nano 2021, 15, 12019. 34157222 10.1021/acsnano.1c03310 PMC8397432

[57] Q. Chen , Y. Zhao , Y. Liu , Chinese Chem. Lett. 2021, 32, 2305.

[58] A. Raz , H. Gubi , A. Cohen , F. Patolsky , ACS Nano 2024, 18, 30848. 39463189 10.1021/acsnano.4c11612 PMC11544710

[59] S. Ma , J. Li , L. Pei , N. Feng , Y. Zhang , J. Pharm. Anal. 2023, 13, 111. 36908860 10.1016/j.jpha.2022.12.004 PMC9999301

[60] A. Himawan , L. K. Vora , A. D. Permana , S. Sudir , A. R. Nurdin , R. Nislawati , R. Hasyim , C. J. Scott , R. F. Donnelly , Adv. Healthcare Mater. 2023, 12, 2202066. 10.1002/adhm.202202066 PMC11468661 36414019

[61] L. Li , Y. Zhou , C. Sun , Z. Zhou , J. Zhang , Y. Xu , X. Xiao , H. Deng , Y. Zhong , G. Li , Acta Biomater. 2024, 175, 199. 38160859 10.1016/j.actbio.2023.12.044

[62] Y. Hu , E. Chatzilakou , Z. Pan , G. Traverso , A. K. Yetisen , Adv. Sci. 2024, 11, 86. 10.1002/advs.202306560 PMC10966570 38225744

[63] K. Wilke , A. Martin , L. Terstegen , S. S. Biel , Int. J. Cosmet. Sci. 2007, 29, 169. 18489347 10.1111/j.1467-2494.2007.00387.x

[64] P. Klaka , S. Grüdl , B. Banowski , M. Giesen , A. Sättler , P. Proksch , T. Welss , T. Förster , PLoS One 2017, 12, 0182752. 10.1371/journal.pone.0182752 PMC5552089 28796813

[65] D. P. Elpa , H. Y. Chiu , S. P. Wu , P. L. Urban , Trends Endocrinol. Metab. 2021, 32, 66. 33353809 10.1016/j.tem.2020.11.009

[66] W. B. SHELLEY , H. J. HURLEY , J. Invest. Dermatol. 1953, 20, 285. 13052978 10.1038/jid.1953.35

[67] K. Sato , R. Leidal , F. Sato , Am. J. Physiol. ‐ Regul. Integr. Comp. Physiol. 1987, 252, R181.

[68] L. B. Baker , Sport. Med. 2017, 47, 111.

[69] J. Moyer , D. Wilson , I. Finkelshtein , B. Wong , R. Potts , Diabetes Technol. Ther. 2012, 14, 398. 22376082 10.1089/dia.2011.0262

[70] V. A. LeGrys , J. Pediatr. 1996, 129, 892. 8969732 10.1016/s0022-3476(96)70034-3

[71] H. Y. Y. Nyein , W. Gao , Z. Shahpar , S. Emaminejad , S. Challa , K. Chen , H. M. Fahad , L.i‐C. Tai , H. Ota , R. W. Davis , A. Javey , ACS Nano 2016, 10, 7216. 27380446 10.1021/acsnano.6b04005

[72] K. Sato , W. H. Kang , K. Saga , K. T. Sato , J. Am. Acad. Dermatol. 1989, 20, 537. 2654204 10.1016/s0190-9622(89)70063-3

[73] Z. Sonner , E. Wilder , J. Heikenfeld , G. Kasting , F. Beyette , D. Swaile , F. Sherman , J. Joyce , J. Hagen , N. Kelley‐Loughnane , R. Naik , Biomicrofluidics 2015, 9, 031301. 26045728 10.1063/1.4921039 PMC4433483

[74] J. Min , J. Tu , C. Xu , H. Lukas , S. Shin , Y. Yang , S. A. Solomon , D. Mukasa , W. Gao , Chem. Rev. 2023, 123, 5049. 36971504 10.1021/acs.chemrev.2c00823 PMC10406569

[75] Kuno Y. Variation in secretory activity of human sweat glands. Institute of Physiology, Imperial University, Kyoto, Japan, 1938, 5971.

[76] N. A. S. Taylor , C. A. Machado‐Moreira , Extrem. Physiol. Med. 2013, 2, 4. 23849497 10.1186/2046-7648-2-4 PMC3710196

[77] P. A. Low , Prim. Auton. Nerv. Syst. Second Ed. 2004, 124.

[78] O. Bar‐Or , L. I. Magnusson , E. R. Buskirk , Hum. Biol. 1968, 40, 235. 5664189

[79] F. Sato , M. Owen , R. Matthes , K. Sato , C. V. Gisolfi , J. Appl. Physiol. 1990, 69, 232. 2203723 10.1152/jappl.1990.69.1.232

[80] Sato K. , Am. J. Physiol. 1987, 252, 181. 10.1152/ajpregu.1987.252.1.R181 3544873

[81] L. B. Baker , Temperature 2019, 6, 211. 10.1080/23328940.2019.1632145 PMC6773238 31608304

[82] S. Jadoon , S. Karim , M. R. Akram , A. Kalsoom Khan , M. A. Zia , A. R. Siddiqi , G. Murtaza , Int. J. Anal. Chem. 2015, 2, 164974. 10.1155/2015/164974 PMC4369929 25838824

[83] F. Criscuolo , I. Ny Hanitra , S. Aiassa , I. Taurino , N. Oliva , S. Carrara , G. De Micheli , Sensors Actuators, B Chem 2021, 328, 129017.

[84] M. M. Raiszadeh , M. M. Ross , P. S. Russo , M. A. Schaepper , W. Zhou , J. Deng , D. Ng , A. Dickson , C. Dickson , M. Strom , C. Osorio , T. Soeprono , J. D. Wulfkuhle , E. F. Petricoin , L. A. Liotta , W. M. Kirsch , J. Proteome Res. 2012, 11, 2127. 22256890 10.1021/pr2007957 PMC3703649

[85] P. A. Low , Clin. Neurophysiol. 2004, 115, 1506. 15203051 10.1016/j.clinph.2004.01.023

[86] J. Heikenfeld , A. Jajack , B. Feldman , S. W. Granger , S. Gaitonde , G. Begtrup , B. A. Katchman , Nat. Biotechnol. 2019, 37, 407. 30804536 10.1038/s41587-019-0040-3

[87] S. R. Tripathi , E. Miyata , P. Ben Ishai , K. Kawase , Sci. Rep. 2015, 5, 9071. 25766116 10.1038/srep09071 PMC4357862

[88] C. P. Lu , L. Polak , A. S. Rocha , H. A. Pasolli , S. C. Chen , N. Sharma , C. Blanpain , E. Fuchs , Cell 2012, 150, 136. 22770217 10.1016/j.cell.2012.04.045 PMC3423199

[89] J. Diao , J. Liu , S. Wang , M. Chang , X. Wang , B. Guo , Q. Yu , F. Yan , Y. Su , Y. Wang , Cell Death Dis. 2019, 10, 238. 30858357 10.1038/s41419-019-1485-5 PMC6411741

[90] N. Liu , S. Huang , B. Yao , J. Xie , X. Wu , X. Fu , Sci. Rep. 2016, 6, 34410. 27694985 10.1038/srep34410 PMC5046070

[91] C. Y. Cui , D. Schlessinger , Exp. Dermatol. 2015, 24, 644. 26014472 10.1111/exd.12773 PMC5508982

[92] D. L. Bovell , Exp. Dermatol. 2018, 27, 544. 29626846 10.1111/exd.13556

[93] K. Sato , F. Sato , Am. J. Physiol. ‐ Cell Physiol. 1981, 10, C113.

[94] P. M. Quinton , Physiology 2007, 22, 212. 17557942 10.1152/physiol.00041.2006

[95] C. Mihályi , I. Iordanov , B. Töröcsik , L. Csanády , Proc. Natl. Acad. Sci. USA 2020, 117, 21740. 32817533 10.1073/pnas.2007910117 PMC7474675

[96] D. B. Salinas , Y. H. Peng , B. Horwich , C. P. Wee , E. Frisbee , J. M. Maarek , Pediatr. Res. 2020, 87, 137. 31344706 10.1038/s41390-019-0503-8 PMC6962560

[97] Z. Sonner , E. Wilder , T. Gaillard , G. Kasting , J. Heikenfeld , Lab Chip 2017, 17, 2550. 28675233 10.1039/c7lc00364a

[98] B. Riedl , M. Nischik , F. Birklein , B. Neundörfer , H. O. Handwerker , J. Auton. Nerv. Syst. 1998, 69, 83. 9696262 10.1016/s0165-1838(98)00016-2

[99] P. A. Low , 1993, 655.

[100] P. A. Low , T. L. Opfer‐Gehrking , M. Kihara , Clin. Auton. Res. 1992, 2, 29. 1638102 10.1007/BF01824208

[101] M. Ohmi , M. Tanigawa , Y. Wada , M. Haruna , Ski. Res. Technol. 2012, 18, 378. 10.1111/j.1600-0846.2011.00580.x 22092881

[102] T. Ohashi , N. Gerrett , S. Shinkawa , T. Sato , R. Miyake , N. Kondo , S. Mitsuzawa , Anal. Chem. 2020, 92, 15534. 33169984 10.1021/acs.analchem.0c03466

[103] M. J. Buono , K. D. Ball , F. W. Kolkhorst , J. Appl. Physiol. 2007, 103, 990. 17600161 10.1152/japplphysiol.00015.2007

[104] P. Simmers , S. K. Li , G. Kasting , J. Heikenfeld , J. Dermatol. Sci. 2018, 89, 40. 29128285 10.1016/j.jdermsci.2017.10.013

[105] M. Shibasaki , C. G. Crandall , J. Appl. Physiol. 2001, 90, 757. 11181580 10.1152/jappl.2001.90.3.757

[106] K. Sato , R. L. Dobson , J. Invest. Dermatol. 1970, 54, 443. 5446389 10.1111/1523-1747.ep12259272

[107] M. Bariya , H. Y. Y. Nyein , A. Javey , Nat. Electron. 2018, 1, 160.

[108] S. Emaminejad , W. Gao , E. Wu , Z. A. Davies , H. Yin Yin Nyein , S. Challa , S. P. Ryan , H. M. Fahad , K. Chen , Z. Shahpar , S. Talebi , C. Milla , A. Javey , R. W. Davis , Proc. Natl. Acad. Sci. USA 2017, 114, 4625. 28416667 10.1073/pnas.1701740114 PMC5422810

[109] M. Zech , M. Benesch , J. Hepp , S. Polifka , B. Glaser , Isotopes Environ. Health Stud. 2019, 55, 394. 31257926 10.1080/10256016.2019.1635125

[110] Y. L. Chen , W. H. Kuan , C. L. Liu , Int. J. Environ. Res. Public Health 2020, 17, 3377.32408694

[111] S. Lin , B.o Wang , Y. Zhao , R. Shih , X. Cheng , W. Yu , H. Hojaiji , H. Lin , C. Hoffman , D. Ly , J. Tan , Y.u Chen , D. Di Carlo , C. Milla , S. Emaminejad , ACS Sens. 2020, 5, 93. 31786928 10.1021/acssensors.9b01727

[112] S. L. Souza , G. Graça , A. Oliva , Ski. Res. Technol. 2018, 24, 187. 10.1111/srt.12412 29131416

[113] M. Bariya , L. Li , R. Ghattamaneni , C. H. Ahn , H. Y. Y. Nyein , L. C. Tai , A. Javey , Sci. Adv. 2020, 6, eabb8308. 32923646 10.1126/sciadv.abb8308 PMC7455190

[114] P. H. Lin , S. C. Sheu , C. W. Chen , S. C. Huang , B. R. Li , Talanta 2022, 241, 123187. 35030501 10.1016/j.talanta.2021.123187

[115] D. Vairo , L. Bruzzese , M. Marlinge , L. Fuster , N. Adjriou , N. Kipson , P. Brunet , J. Cautela , Y. Jammes , G. Mottola , S. Burtey , J. Ruf , R. Guieu , E. Fenouillet , Sci. Rep. 2017, 7, 11801. 28924220 10.1038/s41598-017-12211-y PMC5603548

[116] J. Kim , I. Jeerapan , S. Imani , T. N. Cho , A. Bandodkar , S. Cinti , P. P. Mercier , J. Wang , ACS Sens. 2016, 1, 1011.

[117] S. Li , K. Hart , N. Norton , C. A. Ryan , L. Guglani , M. R. Prausnitz , Bioeng. Transl. Med. 2021, 6, e10222. 34589599 10.1002/btm2.10222 PMC8459588

[118] P. Simmers , Y. Yuan , Z. Sonner , J. Heikenfeld , Biomicrofluidics 2018, 12, 034101. 30867858 10.1063/1.5023396 PMC6404941

[119] M. C. Brothers , M. DeBrosse , C. C. Grigsby , R. R. Naik , S. M. Hussain , J. Heikenfeld , S. S. Kim , Acc. Chem. Res. 2019, 52, 297. 30688433 10.1021/acs.accounts.8b00555

[120] M. Wang , Y. Yang , J. Min , Y.u Song , J. Tu , D. Mukasa , C. Ye , C. Xu , N. Heflin , J. S. McCune , T. K. Hsiai , Z. Li , W. Gao , Nat. Biomed. Eng. 2022, 6, 1225. 35970928 10.1038/s41551-022-00916-z PMC10432133

[121] C. C. S. Gomez , F. A. L. Marson , M. F. Servidoni , A. F. Ribeiro , M. Â. G. O. Ribeiro , V. A. L. Gama , E. T. Costa , J. D. Ribeiro , F. U. Vieira Junior , BMC Pulm. Med. 2018, 18, 153. 30217179 10.1186/s12890-018-0696-3 PMC6137935

[122] B. Yang , X. Jiang , X. Fang , J. Kong , Lab Chip 2021, 21, 4285. 34672310 10.1039/d1lc00438g

[123] L. B. Baker , C. T. Ungaro , B. C. Sopeña , R. P. Nuccio , A. J. Reimel , J. M. Carter , J. R. Stofan , K. A. Barnes , J. Appl. Physiol. 2018, 124, 1304. 29420145 10.1152/japplphysiol.00867.2017

[124] M. M. Rutherford , A. P. Akerman , S. R. Notley , R. D. Meade , M. D. Schmidt , G. P. Kenny , Exp. Physiol. 2021, 322, 1. 10.1152/ajpregu.00161.2021 34786980

[125] Y. Chen , C. Zhang , L. Lu , X. Zheng , S. Chang , Sci. Rep. 2022. 10.1038/s41598-022-04974-w PMC8770470 35046487

[126] E. H. Wissler , Human Temperature Control: A Quantitative Approach, Springer Heidelberg, Germany 2018.

[127] S. Powers , E. Howley , Theory and application to fitness and performence, McGraw Hill LLC 2018.

[128] K. Katić , R. Li , W. Zeiler , Build. Environ. 2016, 106, 286.

[129] D. Gagnon , C. G. Crandall , Handb Clin Neurol 2018, 156, 211. 30454591 10.1016/B978-0-444-63912-7.00013-8

[130] D. Gagnon , G. P. Kenny , J. Physiol. 2012, 590, 5963. 23045336 10.1113/jphysiol.2012.240739 PMC3530110

[131] Y. Lu , Y. Fujita , S. Honda , S. Yang , Y. Xuan , K. Xu , T. Arie , S. Akita , K. Takei , Adv. Healthcare Mater. 2021, 10, 2021. 10.1002/adhm.202100103 33955182

[132] H. Y. Y. Nyein , M. Bariya , B. Tran , C. H. Ahn , B. J. Brown , W. Ji , N. Davis , A. Javey , Nat. Commun. 2021, 12, 1823. 33758197 10.1038/s41467-021-22109-z PMC7987967

[133] C. J. Smith , G. Havenith , Eur. J. Appl. Physiol. 2011, 111, 1391. 21153660 10.1007/s00421-010-1744-8

[134] C. J. Smith , G. Havenith , Med. Sci. Sports Exerc. 2012, 44, 2350. 22811031 10.1249/MSS.0b013e318267b0c4

[135] N. A. Coull , A. M. West , S. G. Hodder , Eur J Appl Physiol. 2021, 121, 109. 32990756 10.1007/s00421-020-04503-5 PMC7815578

[136] M. S. Ummah , Sustain. 2019, 11, 1.

[137] N. A. Coull , A. M. West , S. G. Hodder , P. Wheeler , G. Havenith , Eur. J. Appl. Physiol. 2021, 121, 109. 32990756 10.1007/s00421-020-04503-5 PMC7815578

[138] J. ZHOU , D. MEN , X. E. ZHANG , Chinese J. Anal. Chem. 2022, 50, 87.

[139] Y. Qiao , L. Qiao , Z. Chen , B. Liu , L. Gao , L. Zhang , Chemosensors 2022, 10, 273.

[140] M. Zhang , S. Guo , D. Weller , Y. Hao , X. Wang , C. Ding , K. Chai , B. Zou , R. Liu , J. Nanobiotechnol. 2019, 17, 42. 10.1186/s12951-019-0480-4 PMC6434865 30914060

[141] L. R. Fairservice , T. Bomersback , C. Bjornson , G. Bendiak , Pediatr. Pulmonol. 2018.

[142] C. E. Dziedzic , M. L. Ross , G. J. Slater , L. M. Burke , Int. J. Sports Physiol. Perform. 2014, 9, 832. 24436351 10.1123/ijspp.2013-0480

[143] W. Gao , H. Y. Y. Nyein , Z. Shahpar , H. M. Fahad , K. Chen , S. Emaminejad , Y. Gao , L.i‐C. Tai , H. Ota , E. Wu , J. Bullock , Y. Zeng , D.‐H. Lien , A. Javey , ACS Sens. 2016, 1, 866.

[144] W. Gao , S. Emaminejad , H. Y. Y. Nyein , S. Challa , K. Chen , A. Peck , H. M. Fahad , H. Ota , H. Shiraki , D. Kiriya , D.‐H. Lien , G. A. Brooks , R. W. Davis , A. Javey , Nature 2016, 529, 509. 26819044 10.1038/nature16521 PMC4996079

[145] H. Lee , C. Song , Y. S. Hong , M. S. Kim , H. R. Cho , T. Kang , K. Shin , S. H. Choi , T. Hyeon , D. H. Kim , Sci. Adv. 2017, 3, e1601314. 28345030 10.1126/sciadv.1601314 PMC5342654

[146] Y. Song , J. Min , Y. Yu , H. Wang , Y. Yang , H. Zhang , W. Gao , Sci. Adv 2020, 6, 9842. 10.1126/sciadv.aay9842 PMC7527225 32998888

[147] J. Xu , Y. Zou , A. Nashalian , J. Chen , Front. Chem. 2020, 8, 577327. 33330365 10.3389/fchem.2020.577327 PMC7717947

[148] Z. Peng , J. Song , Y. Gao , J. Liu , C. Lee , G. Chen , Z. Wang , J. Chen , M. K. H. Leung , Nano Energy 2021, 85, 106021.

[149] Y. Zou , V. Raveendran , J. Chen , Nano Energy 2020, 77, 105303.

[150] Y. Zhou , W. Deng , J. Xu , J. Chen , Cell Reports Phys. Sci. 2020, 1, 100142.

[151] W. Deng , Y. Zhou , X. Zhao , S. Zhang , Y. Zou , J. Xu , M. H. Yeh , H. Guo , J. Chen , ACS Nano 2020, 14, 9050. 32627531 10.1021/acsnano.0c04113

[152] E. Bakker , E. Pretsch , TrAC ‐ Trends Anal. Chem. 2005, 24, 199. 10.1016/j.trac.2005.01.003 PMC1482835 16642205

[153] M. J. Patterson , S. D. R. Galloway , M. A. Nimmo , Exp. Physiol. 2000, 85, 869. 11187982 10.1111/j.1469-445x.2000.02058.x

[154] L. Manjakkal , L. Yin , A. Nathan , J. Wang , R. Dahiya , Adv. Mater. 2021, 2, 2100899. 10.1002/adma.202100899 PMC11481680 34247412

[155] E. V. Karpova , E. V. Shcherbacheva , A. A. Galushin , D. V. Vokhmyanina , E. E. Karyakina , A. A. Karyakin , Anal. Chem. 2019, 91, 3778. 30773009 10.1021/acs.analchem.8b05928

[156] L.i‐C. Tai , T. S. Liaw , Y. Lin , H. Y. Y. Nyein , M. Bariya , W. Ji , M. Hettick , C. Zhao , J. Zhao , L. Hou , Z. Yuan , Z. Fan , A. Javey , Nano Lett. 2019, 19, 6346. 31381353 10.1021/acs.nanolett.9b02478

[157] S. Paprocki , M. Qassem , P. A. Kyriacou , Sensors 2022, 22, 6819. 36146167 10.3390/s22186819 PMC9501510

[158] D. Kim , J. Lee , M. K. Park , S. H. Ko , Commun. Mater. 2024, 5, 41.

[159] B. Y. Kim , H. B. Lee , N. E. Lee , Sensors Actuators, B Chem 2019, 283, 312.

[160] D. M. Cate , J. A. Adkins , J. Mettakoonpitak , C. S. Henry , Anal. Chem. 2015, 87, 19. 25375292 10.1021/ac503968p

[161] M. M. Calabretta , M. Zangheri , D. Calabria , A. Lopreside , L. Montali , E. Marchegiani , I. Trozzi , M. Guardigli , M. Mirasoli , E. Michelini , Sensors 2021, 21, 4309. 34202483 10.3390/s21134309 PMC8271422

[162] R. Ghosh , S. Gopalakrishnan , R. Savitha , T. Renganathan , S. Pushpavanam , Sci. Rep. 2019, 9, 7896. 31133720 10.1038/s41598-019-44455-1 PMC6536539

[163] G. Zhu , X. Yin , D. Jin , B. Zhang , Y. Gu , Y. An , TrAC ‐ Trends Anal. Chem. 2019, 111, 100.

[164] S. Zhang , J. Zeng , C. Wang , L. Feng , Z. Song , W. Zhao , Q. Wang , C. Liu , Front. Bioeng. Biotechnol. 2021, 9, 75. 10.3389/fbioe.2021.774210 PMC8692794 34957071

[165] Q. Cao , B. Liang , T. Tu , J. Wei , L. Fang , X. Ye , RSC Adv. 2019, 9, 5674. 35515907 10.1039/c8ra09157a PMC9060762

[166] L. Fiore , V. Mazzaracchio , A. Serani , G. Fabiani , L. Fabiani , G. Volpe , D. Moscone , G. M. Bianco , C. Occhiuzzi , G. Marrocco , F. Arduini , Sensors Actuators B Chem 2023, 379, 133258.

[167] A. Vaquer , E. Barón , R. De La Rica , ACS Sens. 2022, 7, 488. 35172102 10.1021/acssensors.1c02244

[168] T. Someya , Z. Bao , G. G. Malliaras , Nature 2016, 540, 379. 27974769 10.1038/nature21004

[169] J. Park , J. R. Sempionatto , J. Kim , Y. Jeong , J. Gu , J. Wang , I. Park , ACS Sens. 2020, 5, 1363. 32105060 10.1021/acssensors.0c00078

[170] G. Xu , C. Cheng , Z. Liu , W. Yuan , X. Wu , Y. Lu , S. S. Low , J. Liu , L. Zhu , D. Ji , Adv. Mater. Technol. 2019, 4, 59.

[171] D. R. Seshadri , R. T. Li , J. E. Voos , J. R. Rowbottom , C. M. Alfes , C. A. Zorman , C. K. Drummond , npj Digit. Med. 2019, 2, 72. 31341957 10.1038/s41746-019-0150-9 PMC6646404

[172] R. T. Li , S. R. Kling , M. J. Salata , S. A. Cupp , J. Sheehan , J. E. Voos , Sports Health 2016, 8, 74. 26733594 10.1177/1941738115616917 PMC4702159

[173] K. S. Seiler , G. Ø. Kjerland , Scand. J. Med. Sci. Sport. 2006, 16, 49. 10.1111/j.1600-0838.2004.00418.x 16430681

[174] T. J. Gabbett , Br. J. Sports Med. 2016, 50, 273. 26758673 10.1136/bjsports-2015-095788 PMC4789704

[175] S. J. Strath , A. M. Swartz , J. Bassett , W. L. O'Brien , G. A. King , B. E. Ainsworth , Med. Sci. Sports Exerc. 2000, 32, S465. 10993416 10.1097/00005768-200009001-00005

[176] J. Esteve‐Lanao , C. Foster , S. Seiler , A. Lucia , J. Strength Cond. Res. 2007, 21, 943. 17685689 10.1519/R-19725.1

[177] T. Hew‐Butler , M. H. Rosner , S. Fowkes‐Godek , J. P. Dugas , M. D. Hoffman , D. P. Lewis , R. J. Maughan , K. C. Miller , S. J. Montain , N. J. Rehrer , W. O. Roberts , I. R. Rogers , A. J. Siegel , K. J. Stuempfle , J. M. Winger , J. G. Verbalis , Br. J. Sports Med. 2015, 49, 1432. 26227507 10.1136/bjsports-2015-095004

[178] L. B. Baker , J. R. Stofan , A. A. Hamilton , C. A. Horswill , J. Appl. Physiol. 2009, 107, 887. 19541738 10.1152/japplphysiol.00197.2009

[179] A. Koh , D. Kang , Y. Xue , S. Lee , R. M. Pielak , J. Kim , T. Hwang , S. Min , A. Banks , P. Bastien , M. C. Manco , L. Wang , K. R. Ammann , K.‐I.n Jang , P. Won , S. Han , R. Ghaffari , U. Paik , M. J. Slepian , G. Balooch , Y. Huang , J. A. Rogers , Sci. Transl. Med. 2016, 8, 366ra165. 10.1126/scitranslmed.aaf2593 PMC5429097 27881826

[180] J. T. Reeder , J. Choi , Y. Xue , P. Gutruf , J. Hanson , M. Liu , T. Ray , A. J. Bandodkar , R. Avila , W. Xia , S. Krishnan , S. Xu , K. Barnes , M. Pahnke , R. Ghaffari , Y. Huang , J. A. Rogers , Sci. Adv. 2019, 5, 6356. 10.1126/sciadv.aau6356 PMC6357724 30746456

[181] K. Kwon , J. U.k Kim , Y. Deng , S. R. Krishnan , J. Choi , H. Jang , K. Lee , C.‐J.u Su , I. Yoo , Y. Wu , L. Lipschultz , J.‐H. Kim , T. S. Chung , D. Wu , Y. Park , T.‐I. Kim , R. Ghaffari , S. Lee , Y. Huang , J. A. Rogers , Nat. Electron. 2021, 4, 302.

[182] M. J. Buono , N. V. L. Lee , P. W. Miller , J. Physiol. Sci. 2010, 60, 103. 20013328 10.1007/s12576-009-0073-3 PMC10717057

[183] K. Van Hoovels , X. Xuan , M. Cuartero , M. Gijssel , M. Swarén , G. A. Crespo , ACS Sens. 2021, 6, 3496. 34549938 10.1021/acssensors.1c01403 PMC8546758

[184] Y. Seki , D. Nakashima , Y. Shiraishi , T. Ryuzaki , H. Ikura , K. Miura , M. Suzuki , T. Watanabe , T. Nagura , M. Matsumato , M. Nakamura , K. Sato , K. Fukuda , Y. Katsumata , Sci. Rep. 2021, 11, 4929. 33654133 10.1038/s41598-021-84381-9 PMC7925537

[185] E. V. Karpova , A. I. Laptev , E. A. Andreev , E. E. Karyakina , A. A. Karyakin , ChemElectroChem 2020, 7, 191.

[186] G. R. Cutting , Annu. Rev. Genomics Hum. Genet. 2005, 6, 237. 16124861 10.1146/annurev.genom.6.080604.162254

[187] P. M. Q , Nature 1983, 301, 421. 6823316 10.1038/301421a0

[188] K. B. Hammond , N. L. Turcios , L. E. Gibson , J. Pediatr. 1994, 124, 255. 8301433 10.1016/s0022-3476(94)70314-0

[189] D. H. Choi , A. Thaxton , I. cheol Jeong , K. Kim , P. R. Sosnay , G. R. Cutting , P. C. Searson , J. Cyst. Fibros. 2018, 17, 35. 10.1016/j.jcf.2018.03.005 29580829

[190] T. R. Ray , M. Ivanovic , P. M. Curtis , D. Franklin , K. Guventurk , W. J. Jeang , J. Chafetz , H. Gaertner , G. Young , S. Rebollo , 2022, 13, eabd8109. 10.1126/scitranslmed.abd8109 PMC8351625 33790027

[191] G. Riccardi , A. A. Rivellese , Br. J. Nutr. 2000, 83, S143. 10889805 10.1017/s0007114500001082

[192] F. B. Hu , Diabetes Care 2011, 34, 1249. 21617109 10.2337/dc11-0442 PMC3114340

[193] E. K. Amine , N. H. Baba , M. Belhadj , M. Deurenberg‐Yap , A. Djazayery , T. Forrestre , D. A. Galuska , S. Herman , W. P. T. James , J. R. M'Buyamba Kabangu , M. B. Katan , T. J. Key , S. Kumanyika , P. J. Mann , P. J. Moynihan , A. O. Musaiger , G. W. Olwit , J. Petkeviciene , A. M. Prentice , K. S. Reddy , A. Schatzkin , J. C. Seidell , A. P. Simopoulos , S. Srianujata , N. P. Steyn , B. Swinburn , R. Uauy , M. Wahlqvist , W. Zhao‐Su , N. Yoshiike , et al., World Heal. Organ. ‐ Tech. Rep. Ser. 2002, 916, 1.

[194] K. L. Rennie , A. Coward , S. A. Jebb , Br. J. Nutr. 2007, 97, 1169. 17433123 10.1017/S0007114507433086

[195] R. J. Stubbs , L. M. O'Reilly , S. Whybrow , Z. Fuller , A. M. Johnstone , M. B. E. Livingstone , P. Ritz , G. W. Horgan , Br. J. Nutr. 2014, 111, 2032. 24635904 10.1017/S0007114514000154

[196] M. L. Wainberg , P. Scorza , J. M. Shultz , L. Helpman , J. J. Mootz , K. A. Johnson , Y. Neria , J. M. E. Bradford , M. A. Oquendo , M. R. Arbuckle , Curr. Psychiatry Rep. 2017, 19, 28. 28425023 10.1007/s11920-017-0780-z PMC5553319

[197] A. Steptoe , M. Kivimäki , Nat. Rev. Cardiol. 2012, 9, 360. 22473079 10.1038/nrcardio.2012.45

[198] L. Kesner , J. Horáček , Front. Psychiatry 2022, 12, 68640. 10.3389/fpsyt.2021.809239 PMC8785246 35082704

[199] K. L. Hsiao , C. C. Chen , Telemat. Informatics 2018, 35, 103.

[200] S. M. Coulon , C. M. Monroe , D. S. West , Am. J. Prev. Med. 2016, 51, 95. 26993534 10.1016/j.amepre.2016.01.026

[201] J. S. Kang , M. H. Lee , Korean J. Intern. Med. 2009, 24, 1. 19270478 10.3904/kjim.2009.24.1.24 PMC2687659

[202] R. I. Ogilvie , Clin. Pharmacokinet. 1978, 3, 267. 354635 10.2165/00003088-197803040-00002

[203] A. Amdisen , Clin. Pharmacokinet. 1977, 2, 73. 324690 10.2165/00003088-197702020-00001

[204] A. Richens , Clin. Pharmacokinet. 1979, 4, 153. 383353 10.2165/00003088-197904030-00001

[205] Z. K. Shihabi , J. Liq. Chromatogr. 1988, 11, 1579.

[206] J. E. Adaway , B. G. Keevil , J. Chromatogr. B Anal. Technol. Biomed. Life Sci. 2012, 883, 33. 10.1016/j.jchromb.2011.09.041 21992751

[207] M. Carlier , V. Stove , S. C. Wallis , J. J. De Waele , A. G. Verstraete , J. Lipman , J. A. Roberts , Int. J. Antimicrob. Agents 2015, 46, 367. 26271599 10.1016/j.ijantimicag.2015.06.016

[208] J. Brunmair , M. Gotsmy , L. Niederstaetter , B. Neuditschko , A. Bileck , A. Slany , M. L. Feuerstein , C. Langbauer , L. Janker , J. Zanghellini , S. M. Meier‐Menches , C. Gerner , Nat. Commun. 2021, 12, 5993. 34645808 10.1038/s41467-021-26245-4 PMC8514494

[209] L.i‐C. Tai , W. Gao , M. Chao , M. Bariya , Q. P. Ngo , Z. Shahpar , H. Y. Y. Nyein , H. Park , J. Sun , Y. Jung , E. Wu , H. M. Fahad , D.‐H. Lien , H. Ota , G. Cho , A. Javey , Adv. Mater. 2018, 30, 1707442. 10.1002/adma.201707442 29663538

[210] J. M. Moon , H. Teymourian , E. De la Paz , J. R. Sempionatto , K. Mahato , T. Sonsa‐ard , N. Huang , K. Longardner , I. Litvan , J. Wang , Angew. Chemie ‐ Int Ed. 2021, 60, 19074. 10.1002/anie.202106674 PMC8373796 34145703

[211] S. Lin , W. Yu , B.o Wang , Y. Zhao , K.e En , J. Zhu , X. Cheng , C. Zhou , H. Lin , Z. Wang , H. Hojaiji , C. Yeung , C. Milla , R. W. Davis , S. Emaminejad , Proc. Natl. Acad. Sci. USA 2020, 117, 19017. 32719130 10.1073/pnas.2009979117 PMC7431025

[212] W. Raghupathi , V. Raghupathi , Int. J. Environ. Res. Public Health 2018, 15, 431. 29494555 10.3390/ijerph15030431 PMC5876976

[213] Y. Yu , I. Prassas , C. M. J. Muytjens , E. P. Diamandis , J. Proteomics 2017, 155, 40. 28095327 10.1016/j.jprot.2017.01.005

[214] B. A. Katchman , M. Zhu , J. Blain Christen , K. S. Anderson , Proteomics ‐ Clin. Appl. 2018, 12, 25. 10.1002/prca.201800010 PMC6282813 29882373

[215] A. J. Aranyosi , J. B. Model , M. Z. Zhang , S. P. Lee , A. Leech , W. Li , M. S. Seib , S. Chen , N. Reny , J. Wallace , M. H. Shin , A. J. Bandodkar , J. Choi , A. S. Paller , J. A. Rogers , S. Xu , R. Ghaffari , J. Invest. Dermatol. 2021, 141, 433. 32561424 10.1016/j.jid.2020.05.107

[216] B. Jagannath , K. C. Lin , M. Pali , D. Sankhala , S. Muthukumar , S. Prasad , Bioeng. Transl. Med. 2021, 6, e10220. 34589597 10.1002/btm2.10220 PMC8459593

[217] J. Tu , R. M. Torrente‐Rodríguez , M. Wang , W. Gao , Adv. Funct. Mater. 2020, 30, 2004312.

[218] M. A. Morales , J. M. Halpern , Bioconjug. Chem. 2018, 29, 3231. 30216055 10.1021/acs.bioconjchem.8b00592 PMC6416154

[219] M. Vestergaard , K. Kerman , E. Tamiya , Sensors 2007, 7, 3442. 28903304 10.3390/s7123442 PMC3841905

[220] A. Panneer Selvam , S. Muthukumar , V. Kamakoti , S. Prasad , Sci. Rep. 2016, 6, 256. 10.1038/srep23111 PMC4800395 26996103

[221] R. D. Munje , S. Muthukumar , B. Jagannath , S. Prasad , Sci. Rep. 2017, 7, 658. 28512341 10.1038/s41598-017-02133-0 PMC5434046

[222] B. Jagannath , K. C. Lin , M. Pali , D. Sankhala , S. Muthukumar , S. Prasad , Inflamm. Bowel Dis. 2020, 26, 1533. 32720974 10.1093/ibd/izaa191

[223] H. B. Lee , M. Meeseepong , T. Q. Trung , B. Y. Kim , N. E. Lee , Biosens. Bioelectron. 2020, 156, 112133. 32174559 10.1016/j.bios.2020.112133

[224] J. S. Nah , S. C. Barman , M. A. Zahed , M. Sharifuzzaman , H. Yoon , C. Park , S. Yoon , S. Zhang , J. Y. Park , Sensors Actuators, B Chem 2021, 329, 129206.

[225] J. D. R. Thomas , M. Aizawa , I. J. Higgins , W. J. Albery , 1987, 121.

[226] R. M. Torrente‐Rodríguez , J. Tu , Y. Yang , J. Min , M. Wang , Y.u Song , Y. Yu , C. Xu , C. Ye , W. W. IsHak , W. Gao , Matter 2020, 2, 921. 32266329 10.1016/j.matt.2020.01.021 PMC7138219

[227] W. Xue , X. Tan , M. K. Khaing Oo , G. Kulkarni , M. A. Ilgen , X. Fan , Analyst 2020, 145, 1346. 31967116 10.1039/c9an02498k

[228] S. Kim , B. Lee , J. T. Reeder , S. H. Seo , S.‐U.k Lee , A. Hourlier‐Fargette , J. Shin , Y. Sekine , H. Jeong , Y. S. Oh , A. J. Aranyosi , S. P. Lee , J. B. Model , G. Lee , M.‐H.o Seo , S. S. Kwak , S. Jo , G. Park , S. Han , I. Park , H.‐I.l Jung , R. Ghaffari , J. Koo , P. V. Braun , J. A. Rogers , Proc. Natl. Acad. Sci. USA 2020, 117, 27906. 33106394 10.1073/pnas.2012700117 PMC7668081

[229] G. Selvolini , G. Marrazza , Sensors (Switzerland) 2017, 17, 718. 10.3390/s17040718 PMC5421678 28353669

[230] Y. L. Liu , R. Liu , Y. Qin , Q. F. Qiu , Z. Chen , S. B. Cheng , W. H. Huang , Anal. Chem. 2018, 90, 13081. 30272442 10.1021/acs.analchem.8b04223

[231] M. M. Chen , S. B. Cheng , K. Ji , J. Gao , Y. L. Liu , W. Wen , X. Zhang , S. Wang , W. H. Huang , Chem. Sci. 2019, 10, 6295. 31341582 10.1039/c9sc01937e PMC6598512

[232] W. Tang , L. Yin , J. R. Sempionatto , J. M. Moon , H. Teymourian , J. Wang , Adv. Mater. 2021, 33, 2008465. 10.1002/adma.202008465 33786887

[233] J. Zheng , R. Yang , M. Shi , C. Wu , X. Fang , Y. Li , J. Li , W. Tan , Chem. Soc. Rev. 2015, 44, 3036. 25777303 10.1039/c5cs00020c PMC4431697

[234] S. Song , L. Wang , J. Li , C. Fan , J. Zhao , TrAC ‐ Trends Anal. Chem. 2008, 27, 108.

[235] D. Monroe , B. A. Sullenger , C. P. Rusconi , E. Scardino , J. Layzer , G. A. Pitoc , T. L. Ortel , Nature 2002, 419, 90. 12214238 10.1038/nature00963

[236] M. Pali , B. Jagannath , K. C. Lin , S. Upasham , D. Sankhalab , S. Upashama , S. Muthukumar , S. Prasad , Electrochim. Acta 2021, 390, 138834.

[237] N. K. M. Churcher , C. Greyling , S. Upasham , K. C. Lin , P. Rice , M. Pali , J. Spiro , S. Prasad , Biosens. Bioelectron. X 2022, 10, 100145.

[238] T. L. Liu , Y. Dong , S. Chen , J. Zhou , Z. Ma , J. Li , Sci. Adv. 2022, 8, 7049. 10.1126/sciadv.abo7049 PMC9258955 35857473

[239] S. E. Bo Wang , C. Zhao , Z. Wang , K.‐A.e Yang , X. Cheng , W. Liu , W. Yu , S. Lin , Y. Zhao , K. M. Cheung , H. Lin , H. Hojaiji , P. S. Weiss , M. N. Stojanović , A. Janet Tomiyama , A. M. Andrews , Sci. Adv. 2022, 8, abk0967. 10.1126/sciadv.abk0967 PMC8730602 34985954

[240] B. Wang , C. Zhao , Z. Wang , K. A. Yang , X. Cheng , W. Liu , W. Yu , S. Lin , Y. Zhao , K. M. Cheung , H. Lin , H. Hojaiji , P. S. Weiss , M. N. Stojanović , A. J. Tomiyama , A. M. Andrews , S. Emaminejad , Sci. Adv. 2022, 8, eabk0967. 34985954 10.1126/sciadv.abk0967 PMC8730602

[241] N. S. Kirik , B. Şahin , Micro Nanostruct. 2022, 167, 207290.

[242] S. Cinca‐Morros , S. Garcia‐Rey , J. Álvarez‐Herms , L. Basabe‐Desmonts , F. Benito‐Lopez , Anal. Chim. Acta 2024, 1327, 342988. 39266058 10.1016/j.aca.2024.342988

[243] N. Fogh‐Andersen , B. M. Altura , B. T. Altura , O. Siggaard‐Andersen , Clin. Chem. 1995, 41, 1522. 7586528

[244] Y. Kim , M. R. Prausnitz , Nat. Biomed. Eng. 2021, 5, 3. 33483708 10.1038/s41551-020-00679-5

[245] A. J. Bandodkar , W. Jia , C. Yard , X. Wang , J. Ramirez , J. Wang , Anal. Chem. 2015, 87, 394. 25496376 10.1021/ac504300n

[246] A. M. Nightingale , C. L. Leong , R. A. Burnish , S. ul Hassan , Y. Zhang , G. F. Clough , M. G. Boutelle , D. Voegeli , X. Niu , Nat. Commun. 2019, 10, 2741. 31227695 10.1038/s41467-019-10401-y PMC6588579

[247] F. Tehrani , H. Teymourian , B. Wuerstle , J. Kavner , R. Patel , A. Furmidge , R. Aghavali , H. Hosseini‐Toudeshki , C. Brown , F. Zhang , K. Mahato , Z. Li , A. Barfidokht , L.u Yin , P. Warren , N. Huang , Z. Patel , P. P. Mercier , J. Wang , Nat. Biomed. Eng. 2022, 6, 1214. 35534575 10.1038/s41551-022-00887-1

[248] K. Kuruvinashetti , A. Komeili , A. Sanati Nezhad , Lab Chip 2025, 25, 3879. 40678926 10.1039/d5lc00536a

[249] S. R. Chary , R. K. Jain , Proc. Natl. Acad. Sci. USA 1989, 86, 5385. 2748592 10.1073/pnas.86.14.5385 PMC297627

[250] D. Zhang , G. Ding , X. Shen , W. Yao , Z. Zhang , Y. Zhang , J. Lin , Q. Gu , Explor. J. Sci. Heal. 2008, 4, 170. 10.1016/j.explore.2008.02.002 18466847

[251] A. S. Janssens , R. Heide , J. C. D. Hollander , P. G. M. Mulder , B. Tank , A. P. Oranje , J. Clin. Pathol. 2005, 58, 285. 15735162 10.1136/jcp.2004.017210 PMC1770584

[252] T. A. Wilgus , B. C. Wulff , Adv. Wound Care 2014, 3, 356. 10.1089/wound.2013.0457 PMC3985512 24757590

[253] W. Yao , Y. Li , G. Ding , Altern. Med. 2012, 2012, 853516. 10.1155/2012/853516 PMC3534246 23365601

[254] R. Y. Tsay , S. Weinbaum , J. Fluid Mech. 1991, 226, 125.

[255] H.‐H. Hsu , K. Schimek , U. Marx , R. Pörtner , Biomater. Regen. Med. 2018, 132, 56412.

[256] F. Knorr , J. Lademann , A. Patzelt , W. Sterry , U. Blume‐Peytavi , A. Vogt , Eur. J. Pharm. Biopharm. 2009, 71, 173. 19041720 10.1016/j.ejpb.2008.11.001

[257] H. Lee , T. K. Choi , Y. B. Lee , H. R. Cho , R. Ghaffari , L. Wang , H. J. Choi , T. D. Chung , N. Lu , T. Hyeon , S. H. Choi , D.‐H. Kim , Nat. Nanotechnol. 2016, 11, 566. 26999482 10.1038/nnano.2016.38

[258] M. M. Niedzwiecki , P. Samant , D. I. Walker , V. Tran , D. P. Jones , M. R. Prausnitz , G. W. Miller , Anal. Chem. 2018, 90, 3786. 29425024 10.1021/acs.analchem.7b04073 PMC5863097

[259] H. Yu , D. Li , R. C. Roberts , K. Xu , N. C. Tien , J. Microelectromechanical Syst. 2012, 21, 917.

[260] R. Zhao , C. Wang , F. Lu , L. Du , Z. Fang , X. Guo , J. T. Liu , C. J. Chen , Z. Zhao , Sensors 2018, 18, 1431. 29734708 10.3390/s18051431 PMC5982095

[261] J. Kim , A. S. Campbell , J. Wang , Talanta 2018, 177, 163. 29108571 10.1016/j.talanta.2017.08.077

[262] S. K. Li , A. H. Ghanem , K. D. Peck , W. I. Higuchi , J. Pharm. Sci. 1998, 87, 40. 9452966 10.1021/js970189l

[263] J. H. Chang , N. C. Hogan , I. W. Hunter , J. Controlled Release 2015, 211, 37. 10.1016/j.jconrel.2015.05.264 25979330

[264] Z. Pu , C. Zou , R. Wang , X. Lai , H. Yu , K. Xu , D. Li , Biomicrofluidics 2016, 10, 011910. 26958097 10.1063/1.4942437 PMC4769273

[265] Y. Liu , B. Huang , Y. Yao , 2012 IEEE Int. Conf. Mechatronics Autom. 2012, 1, 647.

[266] J. D. Ulrich , J. M. Burchett , J. L. Restivo , D. R. Schuler , P. B. Verghese , T. E. Mahan , G. E. Landreth , J. M. Castellano , H. Jiang , J. R. Cirrito , D. M. Holtzman , Mol. Neurodegener. 2013, 8, 3. 23601557 10.1186/1750-1326-8-13 PMC3640999

[267] H. Wiig , M. A. Swartz , Physiol. Rev. 2012, 92, 1005. 22811424 10.1152/physrev.00037.2011

[268] K. Takeuchi , N. Takama , B. Kim , K. Sharma , P. Ruther , O. Paul , 2018 Japan ICSJ 2018, 85.

[269] P. M. Wang , M. Cornwell , M. R. Prausnitz , Diabetes Technol. Ther. 2005, 7, 131. 15738711 10.1089/dia.2005.7.131

[270] E. V. Mukerjee , S. D. Collins , R. R. Isseroff , R. L. Smith , Sensors Actuators, A Phys 2004, 114, 267.

[271] L. Ventrelli , L. Marsilio Strambini , G. Barillaro , Adv. Healthcare Mater. 2015, 4, 2606. 10.1002/adhm.201500450 26439100

[272] Z. Sheidaei , P. Akbarzadeh , N. Kashaninejad , J. Sci. Adv. Mater. Devices 2020, 5, 295.

[273] J. Yang , R. Luo , L. Yang , X. Wang , Y. Huang , Int. J. Mol. Sci. 2023, 24, 9882. 37373027 10.3390/ijms24129882 PMC10298030

[274] F. K. Aldawood , A. Andar , S. Desai , Polymer (Guildf) 2021, 13, 2815.

[275] J. Yang , S. Zheng , D. Ma , T. Zhang , X. Huang , S. Huang , H. J. Chen , J. Wang , L. Jiang , X. Xie , Sci. Adv. 2022, 8, eabo6900. 36516258 10.1126/sciadv.abo6900 PMC9750147

[276] S. R. Corrie , G. J. P. Fernando , M. L. Crichton , M. E. G. Brunck , C. D. Anderson , M. A. F. Kendall , Lab Chip 2010, 10, 2655. 20820632 10.1039/c0lc00068j

[277] Y. Liu , Q. Yu , X. Luo , L. Yang , Y. Cui , Microsystems Nanoeng 2021, 7, 75. 10.1038/s41378-021-00302-w PMC8481261 34631143

[278] Y. Wu , F. Tehrani , H. Teymourian , J. Mack , A. Shaver , M. Reynoso , J. Kavner , N. Huang , A. Furmidge , A. Duvvuri , Y. Nie , L. M. Laffel , F. J. Doyle , M.‐E. Patti , E. Dassau , J. Wang , N. Arroyo‐Currás , Anal. Chem. 2022, 94, 8335. 35653647 10.1021/acs.analchem.2c00829 PMC9202557

[279] P. P. Samant , M. R. Prausnitz , Proc. Natl. Acad. Sci. USA 2018, 115, 4583. 29666252 10.1073/pnas.1716772115 PMC5939066

[280] F. Ribet , A. Bendes , C. Fredolini , M. Dobielewski , M. Böttcher , O. Beck , J. M. Schwenk , G. Stemme , N. Roxhed , Adv. Healthcare Mater. 2023, 12, e2202564. 10.1002/adhm.202202564 PMC11468663 36748807

[281] K. Y. Goud , C. Moonla , R. K. Mishra , C. Yu , R. Narayan , I. Litvan , J. Wang , ACS Sens. 2019, 4, 2196. 31403773 10.1021/acssensors.9b01127

[282] R. K. Mishra , A. M. Vinu Mohan , F. Soto , R. Chrostowski , J. Wang , Analyst 2017, 142, 918. 28220163 10.1039/c6an02625g

[283] J. Yang , X. Gong , S. Chen , Y. Zheng , L. Peng , B. Liu , Z. Chen , X. Xie , C. Yi , L. Jiang , ACS Sens. 2023, 8, 1241. 36821704 10.1021/acssensors.2c02635

[284] G. S. Liu , Y. Kong , Y. Wang , Y. Luo , X. Fan , X. Xie , B. R. Yang , M. X. Wu , Biomaterials 2020, 232, 119740. 31918227 10.1016/j.biomaterials.2019.119740 PMC7432994

[285] G. Gao , L. Zhang , Z. Li , S. Ma , F. Ma , ACS Biomater. Sci. Eng. 2023, 9, 85. 36475572 10.1021/acsbiomaterials.2c01123

[286] S. Kusama , K. Sato , Y. Matsui , N. Kimura , H. Abe , S. Yoshida , M. Nishizawa , Nat. Commun. 2021, 12, 658. 33510169 10.1038/s41467-021-20948-4 PMC7843990

[287] H. Lee , G. Bonfante , Y. Sasaki , N. Takama , T. Minami , B. Kim , Med. Devices Sensors 2020, 3, e10109.

[288] H. Kai , A. Kumatani , JPhys Energy 2021, 3, 024006.

[289] P. GhavamiNejad , A. GhavamiNejad , H. Zheng , K. Dhingra , M. Samarikhalaj , M. Poudineh , Adv. Healthcare Mater. 2023, 12, e2202362. 10.1002/adhm.202202362 36183355

[290] X. Zhang , Y. Wang , J. Chi , Y. Zhao , Research 2020, 2020, 7462915. 33623910 10.34133/2020/7462915 PMC7877383

[291] A. Mandal , A. V. Boopathy , L. K. W. Lam , K. D. Moynihan , M. E. Welch , N. R. Bennett , M. E. Turvey , N. Thai , J. H. Van , J. C. Love , P. T. Hammond , D. J. Irvine , Sci. Transl. Med. 2018, 10, 56. 10.1126/scitranslmed.aar2227 PMC11972007 30429353

[292] R. F. Donnelly , T. R. R. Singh , M. J. Garland , K. Migalska , R. Majithiya , C. M. McCrudden , P. L. Kole , T. M. T. Mahmood , H. O. McCarthy , A. D. Woolfson , Adv. Funct. Mater. 2012, 22, 4879. 23606824 10.1002/adfm.201200864 PMC3627464

[293] M. Zheng , Z. Wang , H. Chang , L. Wang , S. W. T. Chew , D. C. S. Lio , M. Cui , L. Liu , B. C. K. Tee , C. Xu , Adv. Healthcare Mater. 2020, 9, e1901683. 10.1002/adhm.201901683 32351042

[294] H. Chang , M. Zheng , X. Yu , A. Than , R. Z. Seeni , R. Kang , J. Tian , D. P. Khanh , L. Liu , P. Chen , C. Xu , Adv. Mater. 2017, 29, 36. 10.1002/adma.201702243 28714117

[295] K. Haider , 2025, 1, 13.

[296] L. Zhou , N. Liang , Y. Zheng , X. Sun , J. He , B. Huang , Appl. Mater. Today 2025, 46, 102869.

[297] N. Rabiee , J. Mater. Chem. B 2025, 13, 5264. 40264330 10.1039/d5tb00251f

[298] S. N. Patil , S. N. Jain , S. N. Patil , Y. V. Bhise , Drug Dev. Ind. Pharm. 2025, 51, 1149. 40708230 10.1080/03639045.2025.2529437

[299] E. Larrañeta , J. Moore , E. M. Vicente‐Pérez , P. González‐Vázquez , R. Lutton , A. D. Woolfson , R. F. Donnelly , Int. J. Pharm. 2014, 472, 65. 24877757 10.1016/j.ijpharm.2014.05.042 PMC4111867

[300] A. Munaz , M. J. A. Shiddiky , N. T. Nguyen , Biomicrofluidics 2018, 12, 031501. 29983837 10.1063/1.5035388 PMC6013300

[301] S. Wan , Y. Wang , X. Li , J. Qiu , J. Liu , B. Gao , Anal. Chem. 2025, 97, 12467. 40493246 10.1021/acs.analchem.5c01003

[302] F. Ribet , M. Dobielewski , M. Böttcher , O. Beck , G. Stemme , N. Roxhed , Sens. Bio‐Sensing Res. 2020, 9, 83.

[303] S. Wang , J. M. Tarbell , Arterioscler. Thromb. Vasc. Biol. 2000, 20, 2220. 11031207 10.1161/01.atv.20.10.2220

[304] K. Nagamine , J. Kubota , H. Kai , Y. Ono , M. Nishizawa , Biomed. Microdevices 2017, 19, 68. 28776235 10.1007/s10544-017-0207-y

[305] A. Martín , J. Kim , J. F. Kurniawan , J. R. Sempionatto , J. R. Moreto , G. Tang , A. S. Campbell , A. Shin , M. Y. Lee , X. Liu , X. Liu , J. Wang , ACS Sens. 2017, 2, 1860. 29152973 10.1021/acssensors.7b00729

[306] C. Kolluru , M. Williams , J. Chae , M. R. Prausnitz , Adv. Healthcare Mater. 2019, 8, 1801262. 10.1002/adhm.201801262 PMC6394862 30609270

[307] C. Kolluru , R. Gupta , Q. Jiang , M. Williams , H. Gholami Derami , S. Cao , R. K. Noel , S. Singamaneni , M. R. Prausnitz , ACS Sens. 2019, 4, 1569. 31070358 10.1021/acssensors.9b00258 PMC6679599

[308] N. Kashaninejad , A. Munaz , H. Moghadas , S. Yadav , M. Umer , N. T. Nguyen , Chemosensors 2021, 9, 83.

[309] X. Zhang , G. Chen , F. Bian , L. Cai , Y. Zhao , Adv. Mater. 2019, 31, 256. 10.1002/adma.201902825 31271485

[310] L. Bao , J. Park , B. Qin , B. Kim , Sci. Rep. 2022, 12, 16. 35778408 10.1038/s41598-022-14725-6 PMC9249772

[311] X. Jiang , P. B. Lillehoj , Microsyst. Nanoeng. 2020, 6, 351. 10.1038/s41378-020-00206-1 PMC7605440 33194222

[312] C. Kolluru , M. Williams , J. S. Yeh , R. K. Noel , J. Knaack , M. R. Prausnitz , Biomed. Microdevices 2019, 21, 14. 30725230 10.1007/s10544-019-0363-3 PMC6533066

[313] N. Harpak , E. Borberg , A. Raz , F. Patolsky , ACS Nano 2022, 16, 13800. 36006419 10.1021/acsnano.2c01793 PMC9527802

[314] Q. Yang , Y. Wang , T. Liu , C. Wu , J. Li , J. Cheng , W. Wei , F. Yang , L. Zhou , Y. Zhang , S. Yang , H. Dong , ACS Nano 2022, 16, 18366. 36326107 10.1021/acsnano.2c06261

[315] D. Al Sulaiman , J. Y. H. Chang , N. R. Bennett , H. Topouzi , C. A. Higgins , D. J. Irvine , S. Ladame , ACS Nano 2019, 13, 9620. 31411871 10.1021/acsnano.9b04783 PMC6746174

[316] E. R. Kim , C. Joe , R. J. Mitchell , M. B. Gu , Trends Biotechnol. 2023, 41, 374. 36567185 10.1016/j.tibtech.2022.12.005

[317] H. Teymourian , C. Moonla , F. Tehrani , E. Vargas , R. Aghavali , A. Barfidokht , T. Tangkuaram , P. P. Mercier , E. Dassau , J. Wang , Anal. Chem. 2020, 92, 2291. 31874029 10.1021/acs.analchem.9b05109

[318] P. Bollella , S. Sharma , A. E. G. Cass , F. Tasca , R. Antiochia , Catalysts 2019, 9, 580.

[319] A. M. V. Mohan , J. R. Windmiller , R. K. Mishra , J. Wang , Biosens. Bioelectron. 2017, 91, 574. 28088750 10.1016/j.bios.2017.01.016 PMC5323319

[320] Y. Zheng , R. Omar , R. Zhang , N. Tang , M. Khatib , Q. Xu , Y. Milyutin , W. Saliba , Y. Y. Broza , W. Wu , M. Yuan , H. Haick , Adv. Mater. 2022, 34, 2108607. 10.1002/adma.202108607 34918409

[321] Y. Yao , J. Chen , Y. Guo , T. Lv , Z. Chen , N. Li , S. Cao , B. Chen , T. Chen , Biosens. Bioelectron. 2021, 179, 113078. 33607417 10.1016/j.bios.2021.113078

[322] C. Tortolini , A. E. G. Cass , R. Pofi , A. Lenzi , R. Antiochia , Microchim. Acta 2022, 189, 180. 10.1007/s00604-022-05260-2 PMC8989844 35391571

[323] S. Park , Y. Jae , E. Kostal , V. Matylitskaya , S. Partel , W. Ryu , Biosens. Bioelectron. 2023, 220, 114912. 36413912 10.1016/j.bios.2022.114912

[324] L. Lei , C. Zhao , X. Zhu , S. Yuan , X. Dong , Y. Zuo , H. Liu , Electroanalysis 2022, 34, 415.

[325] K. M. Saifullah , A. Mushtaq , P. Azarikhah , P. D. Prewett , G. J. Davies , Z. Faraji Rad , Microsystems Nanoeng. 2025, 11, 1. 10.1038/s41378-024-00850-x PMC11706973 39774609

[326] S. Kim , M. S. Lee , H. S. Yang , J. H. Jung , Sci. Rep. 2021, 11, 14018. 34234204 10.1038/s41598-021-93235-3 PMC8263571

[327] M. Dervisevic , M. Alba , L. Yan , M. Senel , T. R. Gengenbach , B. Prieto‐Simon , N. H. Voelcker , Adv. Funct. Mater. 2022, 32, 56.

[328] M. Sang , M. Cho , S. Lim , I.n S. Min , Y. Han , C. Lee , J. Shin , K. Yoon , W.‐H. Yeo , T. Lee , S. M. Won , Y. Jung , Y. J. Heo , K.i J. Yu , Sci. Adv. 2023, 9, eadh1765. 37256939 10.1126/sciadv.adh1765 PMC10413647

[329] R. He , H. Liu , T. Fang , Y. Niu , H. Zhang , F. Han , B. Gao , F. Li , F. Xu , Adv. Sci. 2021, 8, e2103030. 10.1002/advs.202103030 PMC8693053 34719884

[330] J. H. Han , C. R. Kim , C. H. Min , M. J. Kim , S. N. Kim , H. B. Ji , S. Bin Yoon , C. Lee , Y. B. Choy , Biosens. Bioelectron. 2023, 238, 115571. 37562343 10.1016/j.bios.2023.115571

[331] Z. Pu , X. Zhang , H. Yu , J. Tu , H. Chen , Y. Liu , X. Su , R. Wang , L. Zhang , D. Li , Sci. Adv. 2021, 7, 26. 10.1126/sciadv.abd0199 PMC7840141 33571117

[332] Y. Chen , S. Lu , S. Zhang , Y. Li , Z. Qu , Y. Chen , B. Lu , X. Wang , X. Feng , Sci. Adv. 2017, 3, 1. 10.1126/sciadv.1701629 PMC5738229 29279864

[333] Y. Cheng , X. Gong , J. Yang , G. Zheng , Y. Zheng , Y. Li , Y. Xu , G. Nie , X.i Xie , M. Chen , C. Yi , L. Jiang , Biosens. Bioelectron. 2022, 203, 114026. 35114468 10.1016/j.bios.2022.114026

[334] S. S. Nemati , G. Dehghan , S. Rashtbari , T. N. Tan , A. Khataee , Microchem. J. 2023, 193, 109038.

[335] M. Govindaraj , A. Srivastava , M. K. Muthukumaran , P. C. Tsai , Y. C. Lin , B. K. Raja , J. Rajendran , V. K. Ponnusamy , J. A. Selvi , Int. J. Biol. Macromol. 2023, 253, 126680. 37673151 10.1016/j.ijbiomac.2023.126680

[336] S. A. Pullano , M. Greco , M. G. Bianco , D. Foti , A. Brunetti , A. S. Fiorillo , Theranostics 2022, 12, 493. 34976197 10.7150/thno.64035 PMC8692922

[337] W. Di , H. A. Clark , Anal. Methods 2020, 12, 1441. 32226484 10.1039/C9AY02717C PMC7100910

[338] R. Smith , S. Collins , J. Duy , T. Minogue , 2018, 104921, 1049102.

[339] H. Teymourian , M. Parrilla , J. R. Sempionatto , N. F. Montiel , A. Barfidokht , R. Van Echelpoel , K. De Wael , J. Wang , ACS Sens. 2020, 5, 2679. 32822166 10.1021/acssensors.0c01318

[340] M. A. Zeitlinger , H. Derendorf , J. W. Mouton , O. Cars , W. A. Craig , D. Andes , U. Theuretzbacher , Antimicrob. Agents Chemother. 2011, 55, 3067. 21537013 10.1128/AAC.01433-10 PMC3122431

[341] T. M. Rawson , S. A. N. Gowers , D. M. E. Freeman , R. C. Wilson , S. Sharma , M. Gilchrist , A. MacGowan , A. Lovering , M. Bayliss , M. Kyriakides , P. Georgiou , A. E. G. Cass , D. O'Hare , A. H. Holmes , Lancet Digit. Heal. 2019, 1, 335. 10.1016/S2589-7500(19)30131-1 33323208

[342] R. Bhake , G. M. Russell , Y. Kershaw , K. Stevens , F. Zaccardi , V. E. C. Warburton , A. C. E. Linthorst , S. L. Lightman , J. Clin. Endocrinol. Metab. 2020, 105, 1749. 10.1210/clinem/dgz002 31529059

[343] M. Venugopal , S. K. Arya , G. Chornokur , S. Bhansali , Sensors Actuators, A Phys 2011, 172, 154. 10.1016/j.sna.2011.04.028 PMC3234992 22163154

[344] V. A. Ryabkova , L. P. Churilov , Y. Shoenfeld , Clin. Immunol. 2021, 223, 108652. 33333256 10.1016/j.clim.2020.108652 PMC7832378

[345] J. Xu , B. Yang , J. Kong , Y. Zhang , X. Fang , Adv. Healthcare Mater. 2023, 12, e2203133. 10.1002/adhm.202203133 36857411

[346] Z. Wang , J. Luan , A. Seth , L. Liu , M. You , P. Gupta , P. Rathi , Y. Wang , S. Cao , Q. Jiang , X. Zhang , R. Gupta , Q. Zhou , J. J. Morrissey , E. L. Scheller , J. S. Rudra , S. Singamaneni , Nat. Biomed. Eng. 2021, 5, 64. 33483710 10.1038/s41551-020-00672-y PMC8020465

[347] K. L. Rock , H. Kataoka , J. J. Lai , Nat. Rev. Rheumatol. 2013, 9, 13. 22945591 10.1038/nrrheum.2012.143 PMC3648987

[348] N. Blau , F. J. Van Spronsen , H. L. Levy , Lancet 2010, 376, 1417. 20971365 10.1016/S0140-6736(10)60961-0

[349] L. Laffel , Diabetes. Metab. Res. Rev. 1999, 15, 412. 10634967 10.1002/(sici)1520-7560(199911/12)15:6<412::aid-dmrr72>3.0.co;2-8

[350] P. Zhang , X. Wu , H. Xue , Y. Wang , X. Luo , L. Wang , Anal. Chim. Acta 2022, 1212, 339911. 35623785 10.1016/j.aca.2022.339911

[351] P. J. Derbyshire , H. Barr , F. Davis , S. P. J. Higson , J. Physiol. Sci. 2012, 62, 429. 22678934 10.1007/s12576-012-0213-z PMC10717375

[352] E. De la Paz , T. Saha , R. Del Caño , S. Seker , N. Kshirsagar , J. Wang , Talanta 2023, 254, 124122. 36459870 10.1016/j.talanta.2022.124122

[353] P. Bollella , S. Sharma , A. E. G. Cass , R. Antiochia , Biosens. Bioelectron. 2019, 123, 152. 30177422 10.1016/j.bios.2018.08.010

[354] J. R. Sempionatto , A. A. Khorshed , A. Ahmed , A. N. De Loyola e Silva , A. Barfidokht , L.u Yin , K. Y. Goud , M. A. Mohamed , E. Bailey , J. May , C. Aebischer , C. Chatelle , J. Wang , ACS Sens. 2020, 5, 1804. 32366089 10.1021/acssensors.0c00604

[355] J. Zhao , H. Y. Y. Nyein , L. Hou , Y. Lin , M. Bariya , C. H. Ahn , W. Ji , Z. Fan , A. Javey , Adv. Mater. 2021, 33, 2006444. 10.1002/adma.202006444 33225539

[356] H. Li , G. Wu , Z. Weng , H. Sun , R. Nistala , Y. Zhang , ACS Sens. 2021, 6, 2181. 34038108 10.1021/acssensors.0c02330

[357] D. D. Zhu , Y. R. Tan , L. W. Zheng , J. Z. Lao , J. Y. Liu , J. Yu , P. Chen , ACS Appl. Mater. Interfaces 2023, 15, 14146. 36916026 10.1021/acsami.3c00573

[358] Á. Molinero‐Fernández , A. Casanova , Q. Wang , M. Cuartero , G. A. Crespo , ACS Sens. 2023, 8, 158. 36475628 10.1021/acssensors.2c01907 PMC9887649

[359] W. Lee , S. H. Jeong , Y. W. Lim , H. Lee , J. Kang , H. Lee , I. Lee , H. S. Han , S. Kobayashi , M. Tanaka , B. S. Bae , Sci. Adv. 2021, 7, eabi6290. 34826244 10.1126/sciadv.abi6290 PMC8626065

[360] M. Dervisevic , E. Dervisevic , L. Esser , C. D. Easton , V. J. Cadarso , N. H. Voelcker , Biosens. Bioelectron. 2023, 222, 114955. 36462430 10.1016/j.bios.2022.114955

[361] X. Zhang , C. Song , K. Yang , W. Hong , Y. Lu , P. Yu , L. Mao , Anal. Chem. 2018, 90, 4968. 29570273 10.1021/acs.analchem.7b05442

[362] S. P. Nichols , A. Koh , W. L. Storm , J. H. Shin , M. H. Schoenfisch , Chem. Rev. 2013, 113, 2528. 23387395 10.1021/cr300387j PMC3624030

[363] X.i Xie , J. C. Doloff , V. Yesilyurt , A. Sadraei , J. J. McGarrigle , M. Omami , O. Veiseh , S. Farah , D. Isa , S. Ghani , I. Joshi , A. Vegas , J. Li , W. Wang , A. Bader , H. H. Tam , J. Tao , H.‐J. Chen , B. Yang , K. A. Williamson , J. Oberholzer , R. Langer , D. G. Anderson , Nat. Biomed. Eng. 2018, 2, 894. 30931173 10.1038/s41551-018-0273-3 PMC6436621

[364] Y. Zhao , B.o Wang , J. Tan , H. Yin , R. Huang , J. Zhu , S. Lin , Y. Zhou , D. Jelinek , Z. Sun , K. Youssef , L. Voisin , A. Horrillo , K. Zhang , B. M. Wu , H. A. Coller , D. C. Lu , Q. Pei , S. Emaminejad , Science 2022, 378, 1222. 36520906 10.1126/science.abn5142

[365] D. W. Kim , M. Kong , U. Jeong , Adv. Sci. 2021, 8, 22. 10.1002/advs.202004170 PMC8061377 33898192

[366] K. Rebrin , G. M. Steil , Diabetes Technol. Ther. 2000, 2, 461. 11467349 10.1089/15209150050194332

[367] T. Bailey , B. W. Bode , M. P. Christiansen , L. J. Klaff , S. Alva , Diabetes Technol. Ther. 2015, 17, 787. 26171659 10.1089/dia.2014.0378 PMC4649725

[368] M. P. Christiansen , S. K. Garg , R. Brazg , B. W. Bode , T. S. Bailey , R. H. Slover , A. Sullivan , S. Huang , J. Shin , S. W. Lee , F. R. Kaufman , Diabetes Technol. Ther. 2017, 19, 446. 28700272 10.1089/dia.2017.0087 PMC5567873

[369] D. K. Vo , K. T. L. Trinh , Biosensors 2024, 14, 560.39590019

[370] S. Shajari , K. Kuruvinashetti , A. Komeili , U. Sundararaj , Sensors 2023, 23, 9498. 38067871 10.3390/s23239498 PMC10708748

[371] K. Dong , L. Tianmei , F. Sheng , X. Peng , Fangzhi Xuebao/Journal Text. Res. 2024.

[372] J. Zhang , H. Li , L. Albakr , Y. Zhang , A. Lu , W. Chen , T. Shao , L. Zhu , H. Yuan , G. Yang , N. J. Wheate , L. Kang , C. Wu , J. Controlled Release 2023, 360, 687. 10.1016/j.jconrel.2023.07.023 37442203

[373] Y. S. Lee , S. Shin , G. R. Kang , S. Lee , D. W. Kim , S. Park , Y. Cho , D. Lim , S. H. Jeon , S. Y. Cho , Nat. Commun. 2025, 16, 289. 40188097 10.1038/s41467-025-58425-x PMC11972314

[374] R. T. Arwani , S. C. L.i Tan , A. Sundarapandi , W. P. Goh , Y. Liu , F. Y. Leong , W. Yang , X. T. Zheng , Y. Yu , C. Jiang , Y. C. Ang , L. Kong , S. L. Teo , P. Chen , X. Su , H. Li , Z. Liu , X. Chen , L.e Yang , Y. Liu , Nat. Mater. 2024, 23, 1115. 38867019 10.1038/s41563-024-01918-9

[375] D. S. Yang , R. Ghaffari , J. A. Rogers , Science 2023, 379, 760. 36821680 10.1126/science.abq5916

[376] C. Yáñez , G. DeMas‐Giménez , S. Royo , Sensors 2022, 22, 6836. 36146183 10.3390/s22186836 PMC9503462

[377] M. J. Baker , S. R. Hussain , L. Lovergne , V. Untereiner , C. Hughes , R. A. Lukaszewski , G. Thiéfin , G. D. Sockalingum , Chem. Soc. Rev. 2016, 45, 1803. 26612430 10.1039/c5cs00585j

[378] B. G. Rosa , O. E. Akingbade , X. Guo , L. Gonzalez‐Macia , M. A. Crone , L. P. Cameron , P. Freemont , K. L. Choy , F. Güder , E. Yeatman , Biosens. Bioelectron. 2022, 251, 116124. 10.1016/j.bios.2022.114050 35134685

[379] B. S. Munge , T. Stracensky , K. Gamez , D. DiBiase , J. F. Rusling , Electroanalysis 2016, 28, 2644. 28592919 10.1002/elan.201600183 PMC5459496

[380] S. Min , H. Geng , Y. He , T. Xu , Q. Liu , X. Zhang , Sen. Diagn. 2025, 4, 370.

[381] S. Staras , J. S. Tauscher , N. Rich , E. Samarah , L. A. Thompson , M. M. Vinson , M. J. Muszynski , E. A. Shenkman , JMIR mHealth uHealth 2021, 9, 18534. 10.2196/18534 PMC8047797 33626016

[382] F. M. Bowens , P. A. Frye , W. A. Jones , Perspect. Health Inf. Manag. 2010, 1, 7. PMC2966355 21063545

[383] J. L. Warner , S. K. Jain , M. A. Levy , Genome Med 2016, 8, 113. 27784327 10.1186/s13073-016-0371-3 PMC5081968

[384] D. D. Johnston , S. W. Vanderstoep , J. W. Creswell , K. Källander , J. K. Tibenderana , O. J. Akpogheneta , D. L. Strachan , Z. Hill , A. H. A. T. Asbroek , L. Conteh , Comput. Educ. 2018, 2, 253.

[385] E. Dolatabadi , Y. X. Zhi , A. J. Flint , A. Mansfield , A. Iaboni , B. Taati , Arch. Gerontol. Geriatr. 2019, 82, 200. 30831526 10.1016/j.archger.2019.02.004

[386] P. Yang , G. Wei , A. Liu , F. Huo , Z. Zhang , npj Flex. Electron. 2022, 6, 33.

[387] H. Liang , H. Ma , X. Duan , J. Yu , H. Wang , S. Li , M. Zhu , A. Chen , H. Zheng , Y. Zhang , Acta Chim. Sin. 2023, 81, 1402.

[388] D. L. Glasco , A. Sheelam , N. H. B. Ho , J. G. Bell , Anal. Chim. Acta 2023, 1273, 341546. 37423672 10.1016/j.aca.2023.341546

[389] D. Paulson , A. Johnson , Creative Commons (CC) BY license, 2025, e2339.

[390] S. N. A. B. M. Nashruddin , F. H. M. Salleh , R. M. Yunus , H. B. Zaman , Heliyon 2024, 10, 37964. 10.1016/j.heliyon.2024.e37964 PMC11425101 39328566

[391] E. U. Alum , Discov. Oncol. 2025, 16, 313. 40082367 10.1007/s12672-025-02064-7 PMC11906928

[392] M. Fazlali , M. Nasira , A. Moravej , Curr. Allergy Asthma Rep 2025, 25, 27. 40459653 10.1007/s11882-025-01207-8

[393] T. Wasilewski , W. Kamysz , J. Gębicki , Biosensors 2024, 14, 356. 39056632 10.3390/bios14070356 PMC11274923

[394] B. Malin , K. Goodman , Yearb. Med. Inform. 2018, 27, 55. 30157505 10.1055/s-0038-1641216 PMC6115244

[395] L. B. Elvas , J. C. Ferreira , M. S. Dias , L. B. Rosário , Systems 2023, 11, 435.

[396] C. E. Guerra , M. E. Fleury , L. P. Byatt , T. Lian , L. Pierce , Am. Soc. Clin. Oncol. Educ. B. 2022, 42, 127. 10.1200/EDBK_350565 35687825

[397] M. McGraw , Oncol. Times 2022, 44, 14.

[398] W. Zhang , R. Wang , F. Luo , P. Wang , Z. Lin , Chinese Chem. Lett. 2020, 31, 589.

[399] B. Gil , B. Li , A. Gao , G. Z. Yang , ACS Appl. Electron. Mater. 2020, 2, 2669. 32879913 10.1021/acsaelm.0c00538 PMC7450887

[400] Z. M. Rittersma , Sensors Actuators, A Phys 2002, 96, 196.

